# Global, regional and national intake of plant-based foods among youth in 185 countries (1990–2018): findings from the Global Dietary Database

**DOI:** 10.1136/bmjgh-2025-021543

**Published:** 2026-07-08

**Authors:** Sydney Yearley, Victoria Miller, Frederick Cudhea, Peilin Shi, Julia R Sharib, Laura Lara-Castor, Kofi Essel, Renata Micha, Jianyi Zhang, Dariush Mozaffarian, Global Dietary Database, Antonia Trichopoulou

**Affiliations:** 1 Food is Medicine Institute, Tufts University Friedman School of Nutrition Science and Policy, Boston, Massachusetts, USA; 2 Tufts University School of Medicine, Boston, Massachusetts, USA; 3 Population Health Research Institute, Department of Medicine, McMaster University, Hamilton, Ontario, Canada; 4 Institute for Health Metrics and Evaluation, University of Washington, Seattle, WA, USA; 5 George Washington University School of Medicine & Health Sciences, Washington DC, District of Columbia, USA; 6 Elevance Health, Indianapolis, Indiana, USA; 7 Department of Food Science and Nutrition, University of Thessaly, Volos, Thessalia Sterea Ellada, Greece; 8 Division of Cardiology, Tufts Medical Center, Boston, Massachusetts, USA; 9 Tufts University Friedman School of Nutrition Science and Policy, Boston, Massachusetts, USA

**Keywords:** Global Health, Child health, Nutrition, Public Health

## Abstract

**Introduction:**

Healthful plant-based foods can improve youth nutrition and contribute to global targets such as Sustainable Development Goal (SDG) 2 on ending malnutrition, SDG3 improving health and SDG12 promoting sustainable diets, yet global intakes remain unquantified.

**Methods:**

We analysed the Global Dietary Database, aggregating 1248 dietary surveys from 185 countries in a Bayesian hierarchical model adjusted for survey heterogeneity, temporal trends and data uncertainty. We assessed energy-adjusted consumption of fruits, non-starchy vegetables, starchy vegetables (excluding potatoes), beans/legumes and nuts/seeds among youth by age (0–19) globally in 2018; evaluated heterogeneity regionally, nationally and by sex, education and urbanicity; and assessed trends from 1990.

**Results:**

Globally, plant-based food intake was low, from 1.19 servings/day (95% uncertainty interval (UI) 1.16 to 1.35) in <1-year-olds to 3.55 servings/day (3.35 to 3.79) in 15–19-year-olds. South Asia had the lowest intakes across all ages; East/Southeast Asia had the highest intakes for several ages, driven by non-starchy vegetables. Intake increased with age in all regions except high-income countries, where <1-year-olds consumed the most (3.77 servings/day (95% UI 3.30 to 4.35)), mainly from fruit (2.43 (2.14 to 2.77)). Across most regions, females consumed more fruit and non-starchy vegetables; urban youth consumed more fruit and nuts/seeds and those from higher educated households had higher intakes of all plant-based foods except beans/legumes. Among the 25 most populous countries, lowest intakes of total plant-based foods were in Spain (1.35 (1.10 to 1.75)), Pakistan (1.43 (1.21 to 1.76)), and the UK (1.71 (1.50 to 2.01)); and highest in Vietnam (4.28 (3.29 to 5.78)), Congo (4.38 (3.49 to 5.95)) and Mexico (5.18 (4.75 to 5.65)). Compared to 1990, youth globally in 2018 consumed more non-starchy vegetables and nuts/seeds, less starchy vegetables, and higher total plant-based foods.

**Conclusion:**

Youth globally consume inadequate healthful plant-based foods, varying substantially by age, region and sociodemographic factors. These findings highlight gaps towards SDGs, underscoring the needs for targeted surveillance, policy interventions, and equity-focused strategies to improve youth dietary quality.

**WHAT IS ALREADY KNOWN ON THIS TOPIC**

- Prior global studies of children’s diets have assessed limited plant-based foods, animal-source foods, or overall dietary patterns. No prior global analysis has evaluated the intakes of multiple healthful plant-based foods across youth ages 0–19 years.

**WHAT THIS STUDY ADDS**

- This study provides comprehensive estimates of global, regional and national consumption of healthful plant-based foods among youth, including first-ever estimates by sex, household education, and urbanicity. It reports intakes of fruits, non-starchy vegetables, starchy vegetables (excluding potatoes), beans/legumes, nuts/seeds, and their total. Temporal trends are also assessed from 1990 to 2018, highlighting dietary changes among youth over nearly three decades.

**HOW THIS STUDY MIGHT AFFECT RESEARCH, PRACTICE OR POLICY**

- This first global analysis of healthful plant-based food consumption among youth offers critical insights for national and subnational surveillance, policy development, and public health interventions. The identified patterns and heterogeneity highlight opportunities for tailored approaches such as age-specific, context-specific or country-specific policies, culturally relevant dietary guidance and systems-level strategies targeting diets from early life to adolescence.

## Introduction

Poor diets in childhood produce major health burdens worldwide.^1^ The double burden of malnutrition includes undernutrition, afflicting 150 million children <age 5 with stunted growth and 43 million with wasting, linked to micronutrient deficiencies, cognitive delays and weakened immunity,^2 3^ and obesity, present in 425 million children <age 18 and increasing the risk of type 2 diabetes, hypertension and cardiovascular disease across the life course.^4^,^7^

Healthful plant-based foods like fruits, vegetables, beans/legumes and nuts/seeds are critical to addressing youth undernutrition. These foods are minimally processed, rich in essential nutrients and support a healthy microbiome, physical and mental development, body weight and cardiometabolic risk profile.^8^ In addition, such foods can partly replace industrial livestock, helping to lower greenhouse gas emissions, conserve freshwater and prevent deforestation.^9^,^12^ However, while the United Nations Sustainable Development Goals (SDGs) aim to eliminate all forms of malnutrition by 2030,^13^ progress has been slow, especially for nutrition-related targets.^14^ Promoting healthful plant-based foods can support these goals and improve youth health.

Dietary behaviours and health outcomes in youth are also influenced by key social determinants, like parental education,^15^ urban or rural residence, other markers of socioeconomic status,^16^ race^17^,^19^ and food security.^16 20^ Dietary intakes likely also vary by child sex and age. However, global data on differences in healthful plant-based food consumption among youth by these factors remain limited.^21^ Understanding these patterns is important for designing interventions that are equity-focused and tailored to at-risk populations with lowest intakes.

Prior studies of plant-based foods among youth globally have included narrow age ranges, for example, focusing on 1–5-year-olds^22^ or limited numbers of foods among 12–17-year-olds.^23^ Prior reports from the Global Dietary Database (GDD) have published more comprehensive global estimates for certain dietary factors among youth, including for sugar-sweetened beverages,^24^ animal-source foods^25^ and overall dietary patterns.^21^ To our knowledge, no global analysis has evaluated intake of multiple healthful plant-based foods across the 0–19 age range. Such data are essential to understanding nutritional challenges and opportunities for youth and for supporting planetary health.

To address these gaps, we had three specific aims: (1) examine consumption of five healthful plant-based foods among youth aged 0–19 years at global, regional and national levels; (2) assess differences by age, sex, household education and urbanicity; and (3) evaluate temporal trends from 1990 to 2018. These data-driven insights help inform targeted interventions and policies to address the double burden of malnutrition, improve child diet and health and achieve the SDGs.

## Methods

### Data sources

We used data from the GDD, an international initiative that provides standardised estimates of dietary intake across 185 countries based on individual-level dietary surveys. Detailed methods for survey selection and standardisation have been described elsewhere.^25^,^32^ The GDD includes 54 dietary factors, food groups, beverages, macronutrients and micronutrients. This analysis focused on five healthful plant-based foods among youth 0–19 years: fruits, non-starchy vegetables, starchy vegetables (excluding potatoes), beans/legumes and nuts/seeds (definitions in online supplemental table S1). Potatoes were excluded due to more limited evidence of their benefit for undernutrition and chronic disease prevention.^33^

### Dietary surveys

The GDD compiled individual-level dietary data organised from national representative samples that used validated dietary assessment methods, such as 24-hour dietary recalls or food frequency questionnaires.^28^ When national surveys were unavailable for any country, large subnational surveys, regional surveys or cohorts without evident selection or measurement bias were included. For any large countries lacking individual-level surveys, WHO STEPwise and household budget surveys were used. Surveys of special populations (eg, pregnant women or people with specific diseases) were excluded.

In total, GDD incorporated 1248 surveys across 185 countries and seven regions, covering an estimated 99% of the global population (online supplemental table S2).^25^ For this analysis, data were derived from 806 surveys for fruit (including a total of 5 209 894 participants), 792 for non-starchy vegetables (N=5 135 389), 380 for beans/legumes (N=3 189 559), 239 for starchy vegetables (N=1 677 068) and 267 for nuts/seeds (N=2 209 704) (online supplemental table S3).

### Data extraction

For each included survey, the GDD team used standardised protocols to extract information on survey characteristics and dietary metrics, including units, mean intake and SD.^25 29^ Data were jointly stratified by age (22 total age groups, which for youth included <1, 1–2, 3–4, 5–9, 10–14 and 15–19 years), sex, urban or rural residence and education level (<6, 6–12, >12 years of education, for youth defined as the highest level attained within the household).^29^ All extracted data were systematically assessed for consistency and verified for accuracy prior to modelling. To ensure cross-survey comparability, all surveys were evaluated using harmonised GDD definitions, units and energy-adjusted metrics. Most individual-level dietary data were directly re-analysed by the original survey investigators, with results provided to the central GDD team; for a subset of surveys, the central GDD team directly analysed the individual-level data shared by the original survey investigators. All intakes were energy-adjusted using standardised age-specific reference values: 700 kcal/day (age 0–0.9 years), 1000 (1–1.9), 1300 (2–5), 1700 (6–10 and ≥75) and 2000 (11–74). For youngest ages (<2 years), intake estimates were for complementary foods, beyond breast milk or formula.

### Data modelling and validation

To address missing data, heterogeneity across surveys and sampling uncertainty, the GDD applied a Bayesian nested hierarchical model, nesting countries within regions and regions within the globe. The model incorporated stratum-specific dietary intake data from each survey, jointly categorised by country, year, age, sex, education and urban/rural residence. The model incorporated country-level covariates and survey-level covariates, including survey type and food definition.

Country-year-specific covariates that were tested for inclusion into the model include data from the United Nations Food and Agricultural Organization (FAO) food balance sheets (1980–2018), Harvard Global Expanded Nutrient Supply (GENuS) (1980–2011), principal component analysis of FAO and GENuS data (2013), Euromonitor fat and oils sales data (1998–2018), World Bank Gross Domestic Product (1980–2018), World Bank unemployment rate (1980–2015), World Bank gini coefficient (1980–2015), World Bank poverty rate (1980–2015), Barro Lee years of schooling (1980–2010), World Bank precipitation (1982–2014), CIA Factbook latitude, CIA Factbook land area and CIA Factbook coastline ratio. Stepwise regression was used to identify three candidate models, each differing only in the set of country-level covariates included. These models were evaluated using fivefold cross-validation. We divide the data into five partitions, randomly binning each survey into a partition. Then, the models were trained on four partitions and tested on the fifth, rotating through all partitions. Model performance was assessed using expected log predictive density. Additional model validity was evaluated by comparing predicted versus observed intakes (excluding implausible estimates) and through visual inspection of global and national mean intakes using heat maps.^25^ Final covariates for each dietary factor are presented in the supplement. Overdispersion parameters in the Bayesian model adjusted for differences in survey quality, such as national representativeness or level of detailed stratification by age, sex or education. The final model estimated mean consumption in servings/day for 264 demographic strata, jointly defined by age (<1 to >95), sex, education (<6, 6–12, >12 years) and urban/rural residence, in each of 185 countries from 1990 to 2018. Stratum-specific intakes were pooled using population-weights for each stratum, calculated using data from the United Nations Population Division^34^ and additional stratification data on education and urbanicity from Barro and Lee^35^ and the United Nations.^36^

We fit each model in STAN^37 38^ using the RStan interface,^39^ employing the No-U-Turn Sampler,^40^ a variant of Hamiltonian Monte Carlo.^41^ Each model was run for 2000 iterations across four chains, with the first 1000 iterations of each chain discarded as burn-in. The remaining 4000 iterations (1000 per chain) were retained to characterise the posterior distributions of the model parameters.

Using these posterior samples, we generated posterior estimates of mean intake for each dietary factor by country-year and subgroup. For each iteration, we applied the sampled model parameters to calculate predicted values as a linear combination of covariates, including uncertainty propagation for countries or regions with no data by drawing from higher-level distributions (eg, region or global level). From these posterior predictive distributions, we calculated summary statistics, including the median and 95% uncertainty intervals (UIs), defined as the 2.5th and 97.5th percentiles. Further details on the methodology used to generate country-level estimates from the model parameters are provided in the supplemental text.

### Statistical analysis

Estimates for each healthful plant-based food and their total were calculated at global, regional, national and within-country subgroup levels, stratified by age, sex, urban/rural residence and education level of the head of household. Findings were evaluated for the GDD age categories (<1, 1–2, 3–4, 5–9, 10–14 and 15–19 years) and for age<2 and 2–19 years.

Intakes were evaluated as mean servings/day with 95% UIs, overall and stratified by subgroups. Spearman correlations were used to assess between-nation relationships among the five food groups (online supplemental tables S4–S9). Absolute differences and percentage differences in mean intakes between subgroups and over time were calculated using all 4000 posterior draws. While formal hypothesis testing is not appropriate for Bayesian analysis, differences were considered meaningful when 95% UIs did not include zero.

### Patient and public involvement

There was no patient or public involvement in the study; we used publicly available or privately stored data to conduct these analyses.

## Results

### Global consumption levels

**Figure 1:**
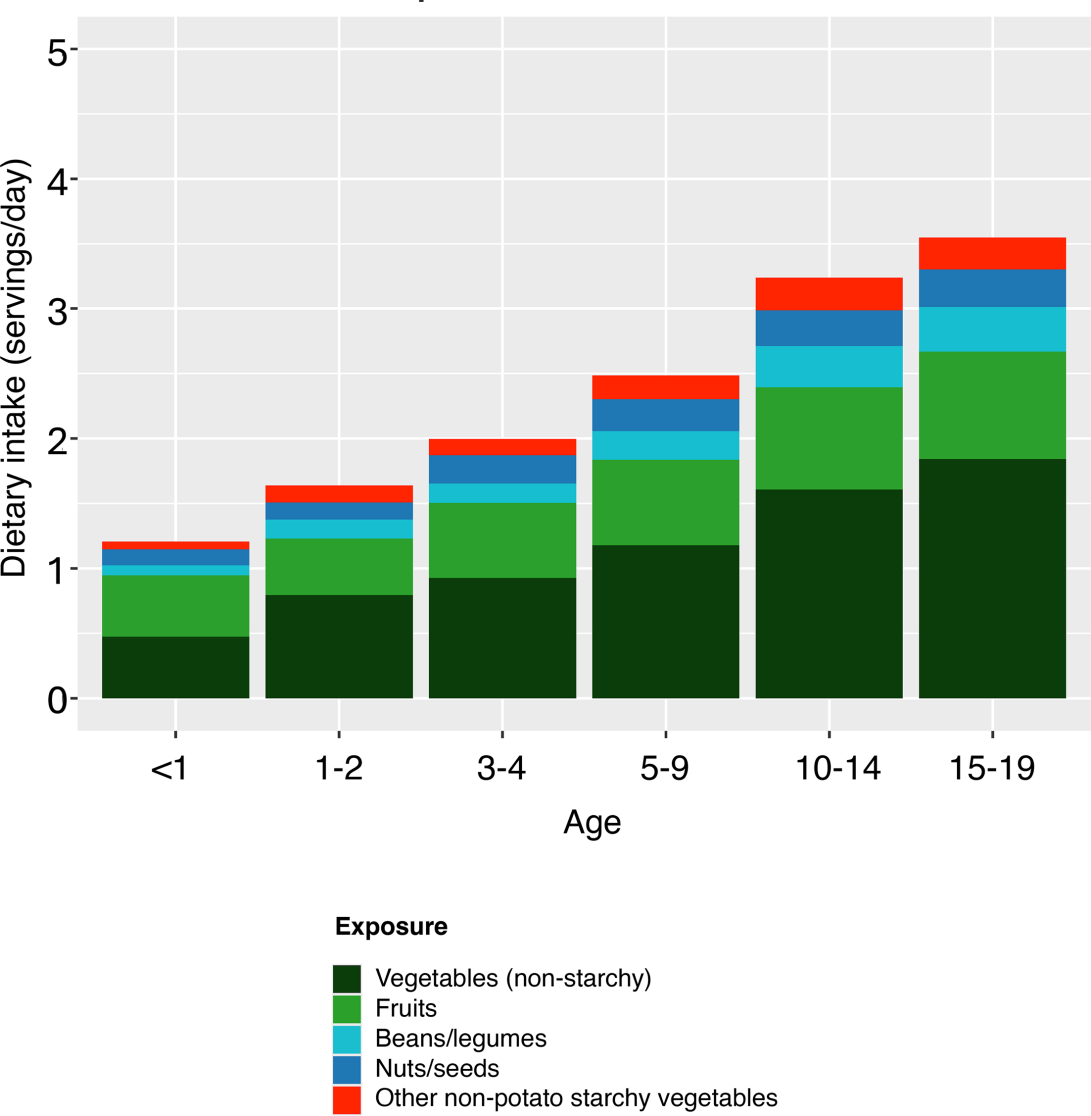
Global energy-adjusted mean intake of plant-based dietary foods by age in 2018. The filled bars represent the mean global consumption (servings/day) of fruits, non-starchy and other non-potato starchy vegetables, beans/legumes and nuts/seeds by age. One serving of fruit is equal to 49 g (6–11 months), 75 g (12–24 months) and 100 g (3–19 years). One serving of non-starchy vegetables is equal to 44 g (6–11 months), 50 g (12–24 months) and 100 g (3–19 years). One serving of other non-potato starchy vegetables is equal to 42 g (6–11 months), 47 g (12–24 months) and 160 g (3–19 years). One serving of beans/legumes is equal to 24 g (6–11 months), 32 g (12–24 months) and 100 g (3–19 years). One serving of nuts/seeds consumption is equal to 24 g (6–11 months), 32 g (12–24 months) and 28.35 g (3–19 years). All estimates are adjusted to age-specific daily energy intake levels as follows: 700 kcal/day for ages 0–0.9 years, 1000 kcal/day for ages 1–1.9 years, 1300 kcal/day for ages 2–5 years, 1700 kcal/day for ages 6–10 years, 2000 kcal/day for ages 11–74 years and 1700 kcal/day for ages >75 years.

In 2018, global total intake of healthful plant-based foods was 1.43 servings/day (95% UI 1.33 to 1.56) in children <2 years and 2.80 (2.65 to 2.99) in children aged 2–19 years (online supplemental tables S10 and S11). Non-starchy vegetables contributed the most in both age groups (0.64 servings/day (95% UI 0.61 to 0.68) and 1.38 (1.33 to 1.44), respectively). Adjusting for age-specific caloric needs, total intake increased nearly threefold from <1 year (1.19 (1.16 to 1.34)) to 15–19 years (3.55 (3.35 to 3.79)), with similar age-related gradients in each plant-based food category (figure 1, online supplemental table S12).

### Regional consumption levels

**Figure 2:**
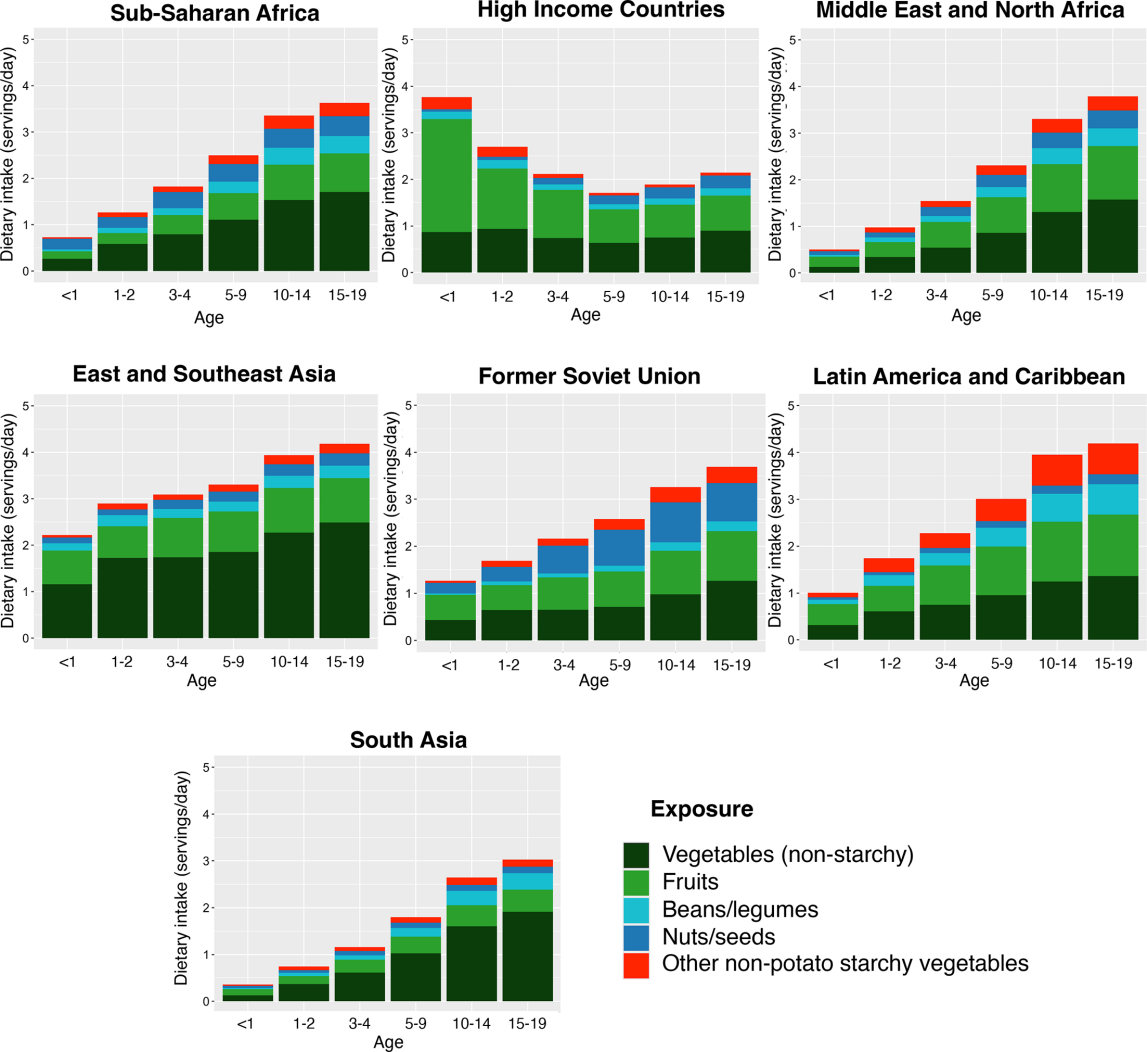
Regional energy-adjusted mean intake of plant-based dietary foods by age in 2018. The filled bars represent the mean consumption (servings/day) of fruits, non-starchy and other non-potato starchy vegetables, beans/legumes and nuts/seeds by region and age. One serving of fruit is equal to 49 g (6–11 months), 75 g (12–24 months) and 100 g (3–19 years). One serving of non-starchy vegetables is equal to 44 g (6–11 months), 50 g (12–24 months) and 100 g (3–19 years). One serving of other non-potato starchy vegetables is equal to 42 g (6–11 months), 47 g (12–24 months) and 160 g (3–19 years). One serving of beans/legumes is equal to 24 g (6–11 months), 32 g (12–24 months) and 100 g (3–19 years). One serving of nuts/seeds consumption is equal to 24 g (6–11 months), 32 g (12–24 months) and 28.35 g (3–19 years). All estimates are adjusted to age-specific daily energy intake levels as follows: 700 kcal/day for ages 0–0.9 years, 1000 kcal/day for ages 1–1.9 years, 1300 kcal/day for ages 2–5 years, 1700 kcal/day for ages 6–10 years, 2000 kcal/day for ages 11–74 years and 1700 kcal/day for ages >75 years.

Age-related trends in energy-adjusted consumption of healthful plant-based foods were generally consistent across world regions, with the notable exception of high-income countries (figure 2, online supplemental table S13). In this region, intake was highest in children <2 years (3.11 servings/day (95% UI 2.80 to 3.52)) and lower in children aged 2–19 years (1.89 (1.79 to 1.98)), the lowest intake for this age group across regions (online supplemental tables S10–S13). In high-income countries, total plant-based food intake peaked in <1-year-olds (3.77 (3.30 to 4.35)), declined in mid-childhood (5–9 years: 1.71 (1.62 to 1.82)), then modestly increased by late adolescence (15–19 years: 2.14 (2.05 to 2.26)) (online supplemental table S13). These trends were driven primarily by declines in fruit and starchy vegetable intake from <1 to 15–19 years, whereas non-starchy vegetables and beans/legumes remained stable, and nuts/seeds increased.

Across world regions, youth in South Asia had the lowest total plant-based food intake at age <2 years and (except for high-income countries) the second lowest intake at ages 2–19 years (online supplemental tables S10 and S11). Age-related trends in South Asia were primarily due to increased non-starchy vegetables and beans/legumes, while fruit and nuts/seeds remained low at all ages (online supplemental table S13). In comparison, youth in East/Southeast Asia had the highest total intake of healthful plant-based foods among world regions, driven by non-starchy vegetables which were high at the youngest age categories and increased across all ages, while fruit consumption was moderately high in early childhood but then declined.

Youth in Latin America/Caribbean had generally the highest starchy vegetable intake (0.66 (0.60 to 0.74) at 10–19 years), highest bean/legume intake after age 2 (0.65 (0.58 to 0.73) at 15–19 years) and highest fruit intake after age 5 (1.31 (1.24 to 1.39) at 15–19 years). Youth in the Former Soviet Union had the highest nuts/seeds intake across ages, peaking at age 10–14 (0.85 (0.65 to 1.08)). In contrast, intakes of fruit and non-starchy vegetables were more moderate, and beans/legumes low, relative to other regions. In the Middle East/North Africa, children <2 years had the second lowest intake of total plant-based foods, after South Asia, driven by the lowest intake of non-starchy vegetables of all regions (online supplemental table S10). By age 15–19, intakes of fruits and non-starchy vegetables increased and were more comparable to other regions (online supplemental table S13). Compared with other regions, youth in Sub-Saharan Africa had intermediate plant-based food consumption across ages (online supplemental tables S10 and S11), with the largest portion from non-starchy vegetables and, across ages, large age-related increases in fruit consumption (online supplemental table S13).

### National consumption levels

**Figure 3:**
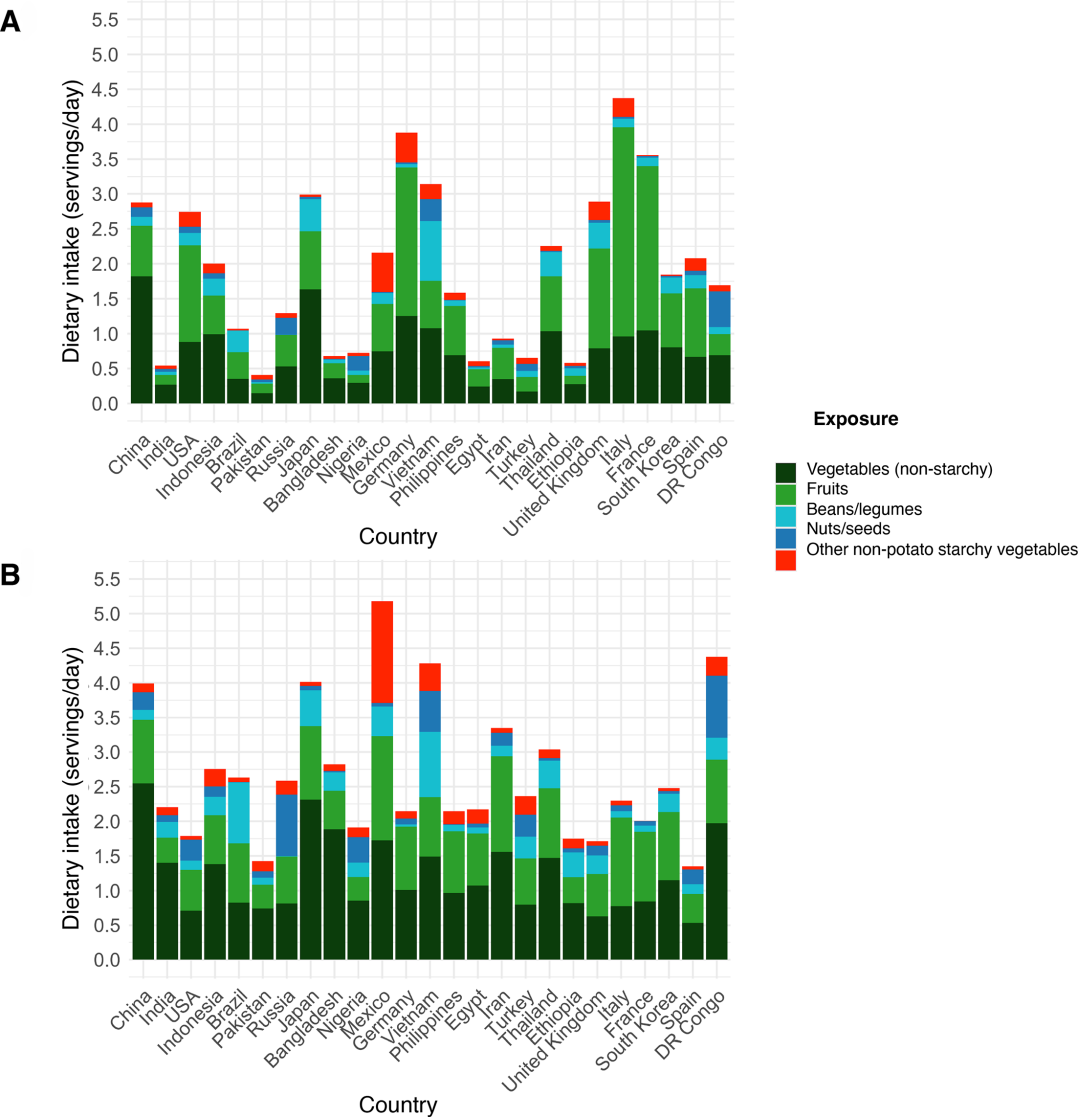
Energy-adjusted mean intake of plant-based dietary foods for (A) infants and toddlers (0–23 months) and (B) children and adolescents (2–19 years) for the 25 most populous countries in 2018. The filled bars represent the mean consumption (servings/day) of fruits, non-starchy and other non-potato starchy vegetables, beans/legumes and nuts/seeds by nation and age. One serving of fruit is equal to 49 g (6–11 months), 75 g (12–24 months) and 100 g (3–19 years). One serving of non-starchy vegetables is equal to 44 g (6–11 months), 50 g (12–24 months) and 100 g (3–19 years). One serving of other non-potato starchy vegetables is equal to 42 g (6–11 months), 47 g (12–24 months) and 160 g (3–19 years). One serving of beans/legumes is equal to 24 g (6–11 months), 32 g (12–24 months) and 100 g (3–19 years). One serving of nuts/seeds consumption is equal to 24 g (6–11 months), 32 g (12–24 months) and 28.35 g (3–19 years). All estimates are adjusted to age-specific daily energy intake levels as follows: 700 kcal/day for ages 0–0.9 years, 1000 kcal/day for ages 1–1.9 years, 1300 kcal/day for ages 2–5 years, 1700 kcal/day for ages 6–10 years, 2000 kcal/day for ages 11–74 years and 1700 kcal/day for ages >75 years.

Across the world’s 25 most populous countries, healthful plant-based food intake varied considerably (figures3  4, online supplemental tables S14 and S15; see onlinesupplemental figures S1S5 and online supplemental file 1 tables S6–S12 for all age categories across all 185 countries). At age <2, lowest total intakes were in Pakistan (0.41 servings/day (95% UI 0.34 to 0.55)), India (0.54 (0.49 to 0.61)) and Ethiopia (0.58 (0.51 to 0.66)), primarily due to low fruits and non-starchy vegetables. Intakes at age <2 years were highest in France (3.56 (3.1 to 4.08)), Germany (3.88 (3.39 to 4.44)) and Italy (4.38 (3.81 to 5.05)), with primary contributors being fruits and/or non-starchy vegetables.

**Figure 4:**
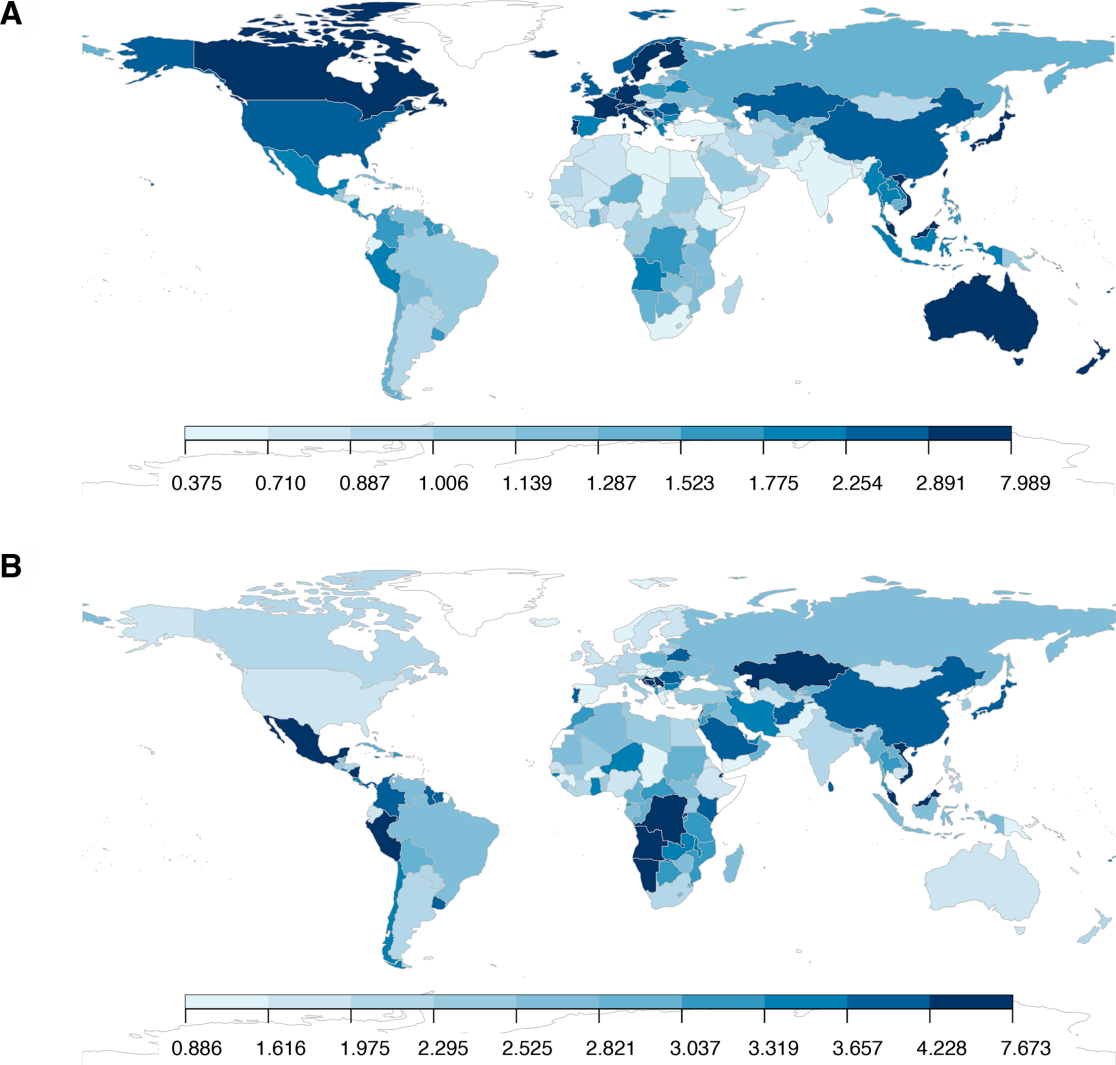
: Global energy adjusted decile ranked mean total intake of plant-based dietary foods in (A) infants and toddlers 0–23 months and (B) children and adolescents 2–19 years. The coloured countries are the decile ranked mean total consumption (servings/day) of fruits, non-starchy and other non-potato starchy vegetables, beans/legumes and nuts/seeds by country and by infants and toddlers (0–23 months) vs children and adolescents (2–19 years). Total consumption of plant-based foods is ranked into deciles, with light blue indicating the lowest decile of total consumption and dark blue indicating the highest decile of consumption. One serving of fruit is equal to 49 g (6–11 months), 75 g (12–24 months) and 100 g (3–19 years). One serving of non-starchy vegetables is equal to 44 g (6–11 months), 50 g (12–24 months) and 100 g (3–19 years). One serving of other non-potato starchy vegetables is equal to 42 g (6–11 months), 47 g (12–24 months) and 160 g (3–19 years). One serving of beans/legumes is equal to 24 g (6–11 months), 32 g (12–24 months) and 100 g (3–19 years). One serving of nuts/seeds consumption is equal to 24 g (6–11 months), 32 g (12–24 months) and 28.35 g (3–19 years). All estimates are adjusted to age-specific daily energy intake levels as follows: 700 kcal/day for ages 0–0.9 years, 1000 kcal/day for ages 1–1.9 years, 1300 kcal/day for ages 2–5 years, 1700 kcal/day for ages 6–10 years, 2000 kcal/day for ages 11–74 years and 1700 kcal/day for ages >75 years.

For youth aged 2–19 years, lowest intakes of total healthful plant-based foods were seen in Spain (1.35 (1.10 to 1.75)), Pakistan (1.43 (1.21 to 1.76)) and the UK (1.71 (1.50 to 2.01)); and highest in Vietnam (4.28 (3.29 to 5.78)), the Democratic Republic of the Congo (4.38 (3.49 to 5.95)) and Mexico (5.18 (4.75 to 5.65)). Higher intakes were primarily driven by fruits and non-starchy vegetables, as well as starchy vegetables in Mexico.

### Consumption levels by demographic subgroups

Total intake of healthful plant-based foods was modestly lower in boys versus girls globally (−0.18 servings/day (95% UI −0.32 to −0.05)), in high-income countries (−0.19 (-0.29 to −0.10)) and in East/Southeast Asia (−0.41 (–0.79 to −0.07)) (online supplemental figure S6, online supplemental table S2). This was because boys generally consumed less fruits and non-starchy vegetables than girls. Differences by sex in other plant-based foods were minor and inconsistent across regions. Percentage differences by sex are shown in online supplemental table S23.

**Figure 5:**
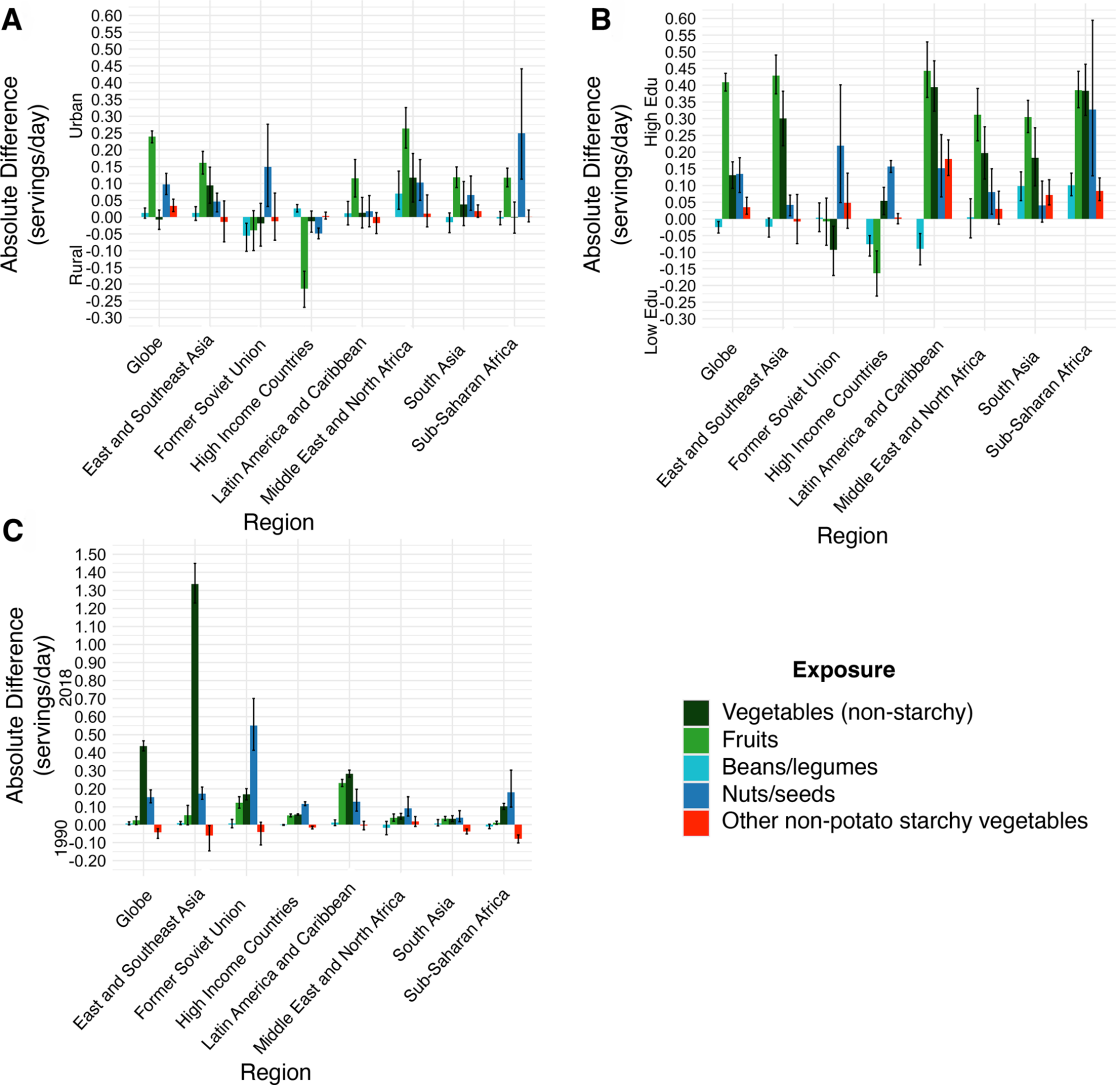
Mean absolute difference of (A) urban versus rural residence (2018), (B) high versus low education households (2018) and (C) 1990 versus 2018 in youth 0–19 years. The filled bars represent the absolute mean difference in servings/day in consumption of fruits, non-starchy and other non-potato starchy vegetables, beans/legumes and nuts/seeds, and the error bars the 95% uncertainty interval. Positive values indicate greater mean consumption in (A) urban versus rural households; (B) high (>12 years) versus low (<6 years) education households (middle education (>6–<12 years) excluded) and (C) 2018 versus 1990. One serving of fruit is equal to 49 g (6–11 months), 75 g (12–24 months) and 100 g (3–19 years). One serving of non-starchy vegetables is equal to 44 g (6–11 months), 50 g (12–24 months) and 100 g (3–19 years). One serving of other non-potato starchy vegetables is equal to 42 g (6–11 months), 47 g (12–24 months) and 160 g (3–19 years). One serving of beans/legumes is equal to 24 g (6–11 months), 32 g (12–24 months) and 100 g (3–19 years). One serving of nuts/seeds consumption is equal to 24 g (6–11 months), 32 g (12–24 months) and 28.35 g (3–19 years). All estimates are adjusted to age-specific daily energy intake levels as follows: 700 kcal/day for ages 0–0.9 years, 1000 kcal/day for ages 1–1.9 years, 1300 kcal/day for ages 2–5 years, 1700 kcal/day for ages 6–10 years, 2000 kcal/day for ages 11–74 years and 1700 kcal/day for ages >75 years.

Globally, children in urban households consumed more healthful plant-based foods than those in rural households (+0.37 servings/day (95% UI 0.27 to 0.49)), a pattern consistent across most regions except high-income countries, where intake was lower in urban areas (−0.24 (–0.36 to −0.12)) (figure 5, online supplemental table S24). Fruit consumption was similarly higher in urban youth globally (+0.24 (0.22 to 0.26)) and regionally, except again in high-income countries, where it was lower (−0.21 (–0.27 to −0.16)). Urban youth also consumed more nuts/seeds globally (+0.10 (0.07 to 0.13)) and in most regions. In contrast, urban–rural differences in youth intake were smaller for non-starchy vegetables, starchy vegetables and beans/legumes, without consistent regional patterns. Percentage differences are in online supplemental table S25.

Children in more educated households had higher healthful plant-based food consumption than in lower education households globally (+0.69 (0.52 to 0.84)) and in most regions (figure 5, online supplemental table S26). These differences reflect higher intakes of starchy vegetables (+0.04 (0.01 to 0.06)), non-starchy vegetables (+0.13 (0.09 to 0.17)), nuts/seeds (+0.13 (0.08 to 0.18)) and fruits (+0.41 (0.38 to 0.44)) in more educated households. These patterns were generally consistent across regions, except that more educated households had lower fruit intake in high-income countries (−0.16 (–0.23 to −0.10)) and lower non-starchy vegetable intake in the Former Soviet Union (−0.09 (–0.17 to −0.02)). Percentage differences are in online supplemental table S27.

### Trends over time

Total intake of healthful plant-based foods among youth increased from 1990 to 2018 globally and in all regions except South Asia (figure 5, online supplemental table S28). Most of this was due to increases in non-starchy vegetables (+0.44 servings/day (95% UI 0.41 to 0.47)) and nuts/seeds (+0.15 (0.12 to 0.19)). In contrast, intake of beans/legumes and fruit remained relatively stable, while intake of starchy vegetables declined globally and in most regions. Percentage differences are in online supplemental table S29.

## Discussion

This global, regional and national analysis of healthful plant-based food consumption among youth reveals important differences by food group, world region, age, sex, urbanicity and household education. To our knowledge, this is the first global report on these foods across youth, a critical yet understudied population. Healthful plant-based food consumption in youth is essential to achieving global nutrition and diet-related disease goals. The findings have implications for childhood nutrition and policy development, including for surveillance, nutrition assistance programmes^42^ and healthcare interventions.^43^ Our findings highlight major gaps, for example, in progress towards SDG targets on ending malnutrition (SDG2) and ensuring health (SDG3).

Consumption of total plant-based foods by youth remains low, averaging 2.80 servings/day overall and no more than 3.55 daily servings in any age group at a global level. Dietary shortfalls of these foods during childhood are linked to suboptimal growth, cognitive development and immune function^2 3^ as well as high blood pressure, dyslipidaemia, pre-diabetes,^44^ diabetes^45^ and obesity.^46^ Childhood dietary patterns also influence lifelong eating behaviours and chronic disease risk, reinforcing the need for interventions that support national and global objectives for child nutrition and health.^47^

We also identified substantial variation across regions and subgroups, underscoring the opportunities for context-specific interventions. In high-income countries, fruit and vegetable intake uniquely declined with age, consistent with prior reports from individual Western countries.^48 49^ This suggests that, despite higher purchasing power, other factors such as youth autonomy, food environments or cultural norms have a particular hold among older youth in this region. Many high-income nations have early-life nutrition programmes that include healthful plant-foods,^50^,^54^ which could explain higher intakes at youngest ages. In comparison, youth in East/Southeast Asia consumed more non-starchy vegetables, which may reflect cultural preferences and traditional market affordability^55^; yet, total healthful plant-food consumption remains below recommendations, indicating potential challenges around food system inefficiencies, production gaps, costs or educational and cultural barriers.^56 57^ We found that South Asia had very low intake of healthful plant-based foods, building on earlier findings evaluating fruits and vegetables in this region.^56^ These identified regional patterns inform future research and policy on the social, economic and cultural determinants of youth diets.

These regional findings highlight a few opportunities for policy priorities, including (1) strengthening and extending early childhood nutrition programmes in high-income countries to sustain healthy plant-based eating into adolescence; (2) improving access to diverse vegetables in South Asia through agricultural policy and market interventions; (3) leveraging existing cultural preferences for vegetables in East/Southeast Asia while addressing remaining gaps in fruit and vegetable consumption and (4) implementing equity-focused interventions targeting rural and lower-education households across most regions, while recognising reversed patterns in high-income settings.

Although many country-specific findings mirror their region, others diverge, such as high fruit and vegetable intake among youth in Mexico. These patterns underscore the need for both regionally and nationally targeted approaches to advance nutrition and health among youth.

Our subgroup analyses revealed that girls consumed slightly more fruits and non-starchy vegetables than boys, consistent with prior literature from individual countries.^58^ However, intakes of beans/legumes, nuts/seeds and other starchy vegetables were similar by sex, new findings not previously observed globally. In contrast, larger differences were seen by household education and urbanicity. Youth in higher-educated households generally consumed more healthful plant-based foods, suggesting a positive influence of education (and associated income and food access) on diet quality.^21^ An exception was beans/legumes, which were more common in lower-education households, suggesting a potential role for cultural tradition, affordability and nutrient-to-price advantages of this food group.^59^ Compared with rural youth, urban youth generally consumed more fruits and nuts/seeds, consistent with differences in income and market access. Interestingly, these education and urbanicity patterns were generally reversed in high-income countries, suggesting that other factors, like home production or more traditional diets, may promote healthful plant-based foods among children in rural or lower education households, an area ripe for research.

We found that over nearly three decades, youth consumption of non-starchy vegetables and nuts/seeds increased globally, which could relate to improved supply chains for access to more varied and non-seasonal produce.^60^ However, overall consumption of plant-based foods remained low in 2018, and absolute changes in intake were small in several regions and for several food groups (eg, fruits, beans/legumes), while consumption of starchy vegetables declined globally. These findings underscore the insufficient progress towards the SDGs and improving nutrition in youth, and the need for new and accelerated efforts to improve production, affordability, access, convenience and consumption.

In 2021, the Global Nutrition Report used GDD data to estimate that more than half the world’s children do not meet WHO–UNICEF dietary guidelines for fruits and vegetables, while two-thirds fall short on recommendations for beans/legumes and nuts/seeds.^61^ Our results expand on these findings by evaluating consumption across regions, nations and subgroups by age, sex, urbanicity and education. Other strengths of our investigation include the incorporation of 1248 mostly nationally representative, individual-level dietary surveys from countries representing 99% of the global population. This reduces misclassification, bias and ecologic fallacy from relying on national per capita food availability data, and further permits evaluation of intakes by age, sex, urbanicity and education. Model estimates were validated using fivefold cross-validation and model fit criteria; and heterogeneity and missingness were incorporated into uncertainty estimates.

Limitations include variability in the quality and completeness of input data, which were more limited for some countries, dietary exposures and time periods. Our findings should therefore be considered the best available, but still imperfect, estimates of these foods among youth; the reported uncertainty intervals should be considered in this regard. Standardisation and harmonisation of data inputs increased reliability and comparability but did not allow for more granular assessments of foods, such as subtypes of fruits or legumes. Given the time frame of GDD data collection, our results provide findings through 2018. Dietary patterns may have shifted since 2018 due to the COVID-19 pandemic, economic disruptions and climate events, highlighting the importance of continued surveillance and future GDD updates.

In conclusion, consumption of plant-based foods remains low among youth. The identified patterns and heterogeneity underscore opportunities for tailored interventions, such as age-specific, context-specific or country-specific policies, culturally relevant dietary guidance and systems-level strategies targeting diets in both early life and adolescence. These actions are critical to closing gaps in global nutrition, advancing child health and supporting food systems in line with the SDGs.

## Supplementary material

10.1136/bmjgh-2025-021543 Supplementary Figure 1

10.1136/bmjgh-2025-021543 Supplementary Figure 2

10.1136/bmjgh-2025-021543 Supplementary Figure 3

10.1136/bmjgh-2025-021543 Supplementary Figure 4

10.1136/bmjgh-2025-021543 Supplementary Figure 5

10.1136/bmjgh-2025-021543 Supplementary Figure 6

10.1136/bmjgh-2025-021543 Supplementary file 1

## Data Availability

Data are available in a public, open access repository. Data are available upon reasonable request.
