# Supplementary material for: Global, regional and national intake of plant-based foods among youth in 185 countries (1990–2018): findings from the Global Dietary Database
Source: BMJ Glob Health. 2026 Jul 8;11(7):e021543. doi: 10.1136/bmjgh-2025-021543 (PMC13358298; doi:10.1136/bmjgh-2025-021543)
Supplement: Supplementary Figure 3 [file bmjgh-11-7-s003.pdf]

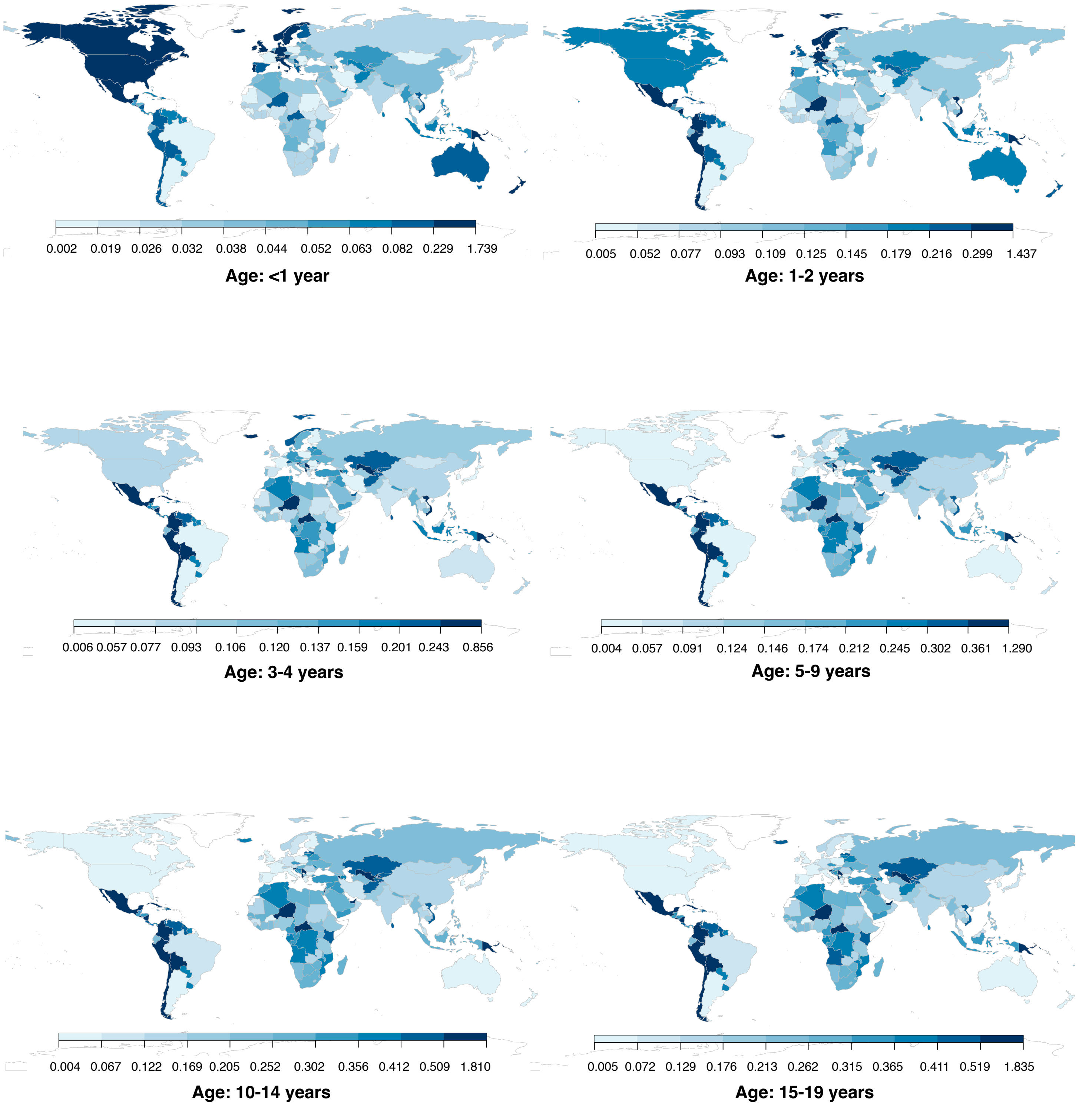

Supplemental Figure 3: National energy adjusted decile ranked mean other non-potato starchy vegetable consumption in youth 0-19 years of age in 2018. The colored countries are the decile ranked mean total non-starchy vegetable consumption (servings/day) by country and by age. Other non-potato starchy vegetable consumption is ranked into deciles with light blue indicating the lowest decile of total consumption and dark blue indicating the highest decile of consumption. One serving of other non-potato starchy vegetables is equal to 42 g (6-11 months), 47 g (12-24 months), and 160 g (3-19 years). All estimates are adjusted to age-specific daily energy intake levels as follows: 700 kcal/day for ages 0-0.9 years, 1,000 kcal/day for ages 1-1.9 years, 1,300 kcal/day for ages 2-5 years, 1,700 kcal/day for ages 6-10 years, 2,000 kcal/day for ages 11-74 years, and 1,700 kcal/day for ages ≥75 years.
