# Supplementary material for: Global, regional and national intake of plant-based foods among youth in 185 countries (1990–2018): findings from the Global Dietary Database
Source: BMJ Glob Health. 2026 Jul 8;11(7):e021543. doi: 10.1136/bmjgh-2025-021543 (PMC13358298; doi:10.1136/bmjgh-2025-021543)
Supplement: Supplementary Figure 4 [file bmjgh-11-7-s004.pdf]

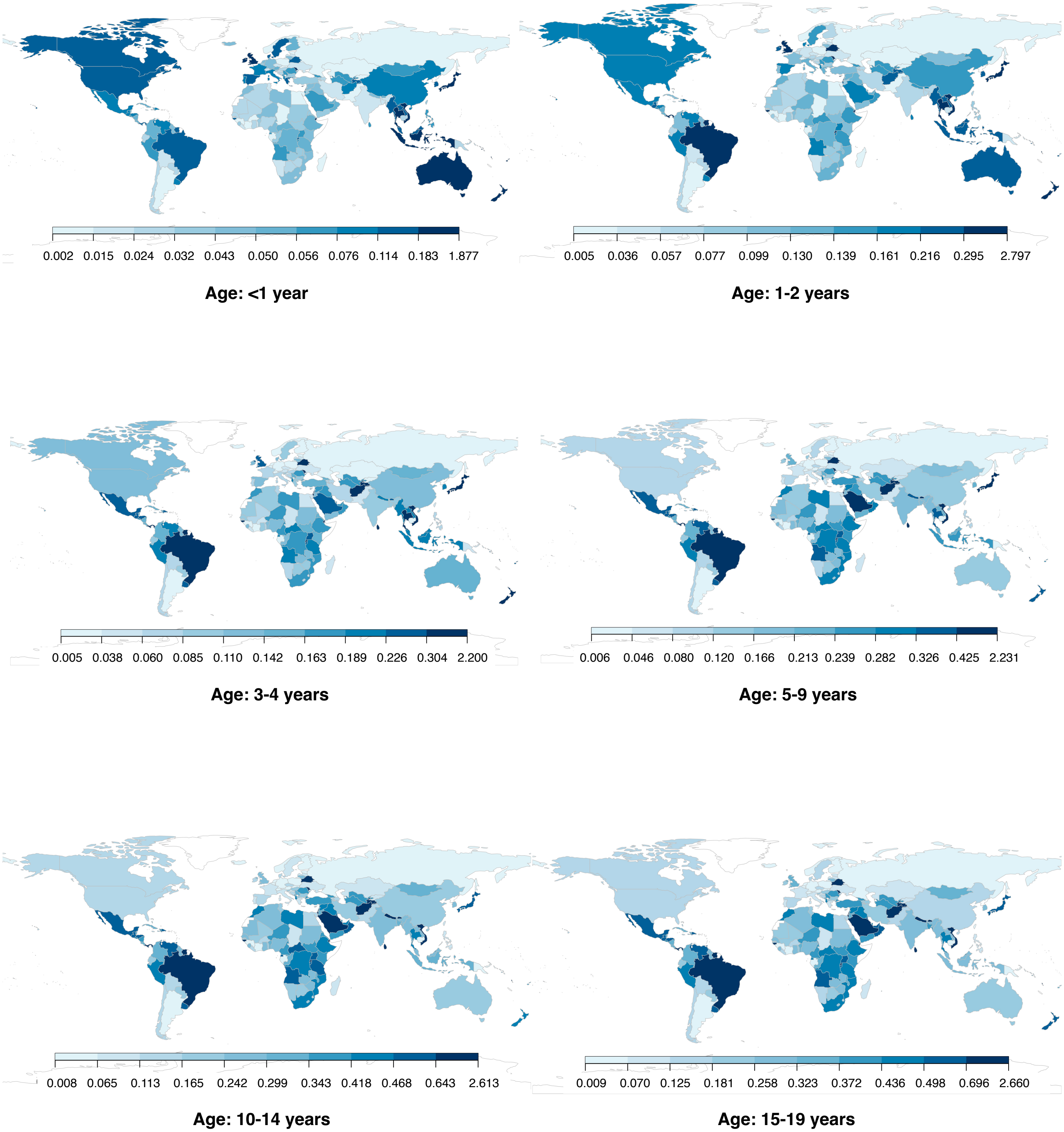

Supplemental Figure 4: National energy-adjusted decile ranked mean beans/legumes consumption in youth 0-19 years of age in 2018. The colored countries are the decile ranked mean total beans/legumes consumption (servings/day) by country and by age. Beans/legumes consumption is ranked into deciles with light blue indicating the lowest decile of total consumption and dark blue indicating the highest decile of consumption. One serving of beans/legumes is equal to 24 g (6-11 months), 32 g (12-24 months), and 100 g (3-19 years). All estimates are adjusted to age-specific daily energy intake levels as follows: 700 kcal/day for ages 0-0.9 years, 1,000 kcal/day for ages 1-1.9 years, 1,300 kcal/day for ages 2-5 years, 1,700 kcal/day for ages 6-10 years, 2,000 kcal/day for ages 11-74 years, and 1,700 kcal/day for ages ≥75 years.
