# Supplementary material for: Global, regional and national intake of plant-based foods among youth in 185 countries (1990–2018): findings from the Global Dietary Database
Source: BMJ Glob Health. 2026 Jul 8;11(7):e021543. doi: 10.1136/bmjgh-2025-021543 (PMC13358298; doi:10.1136/bmjgh-2025-021543)
Supplement: Supplementary Figure 6 [file bmjgh-11-7-s006.pdf]

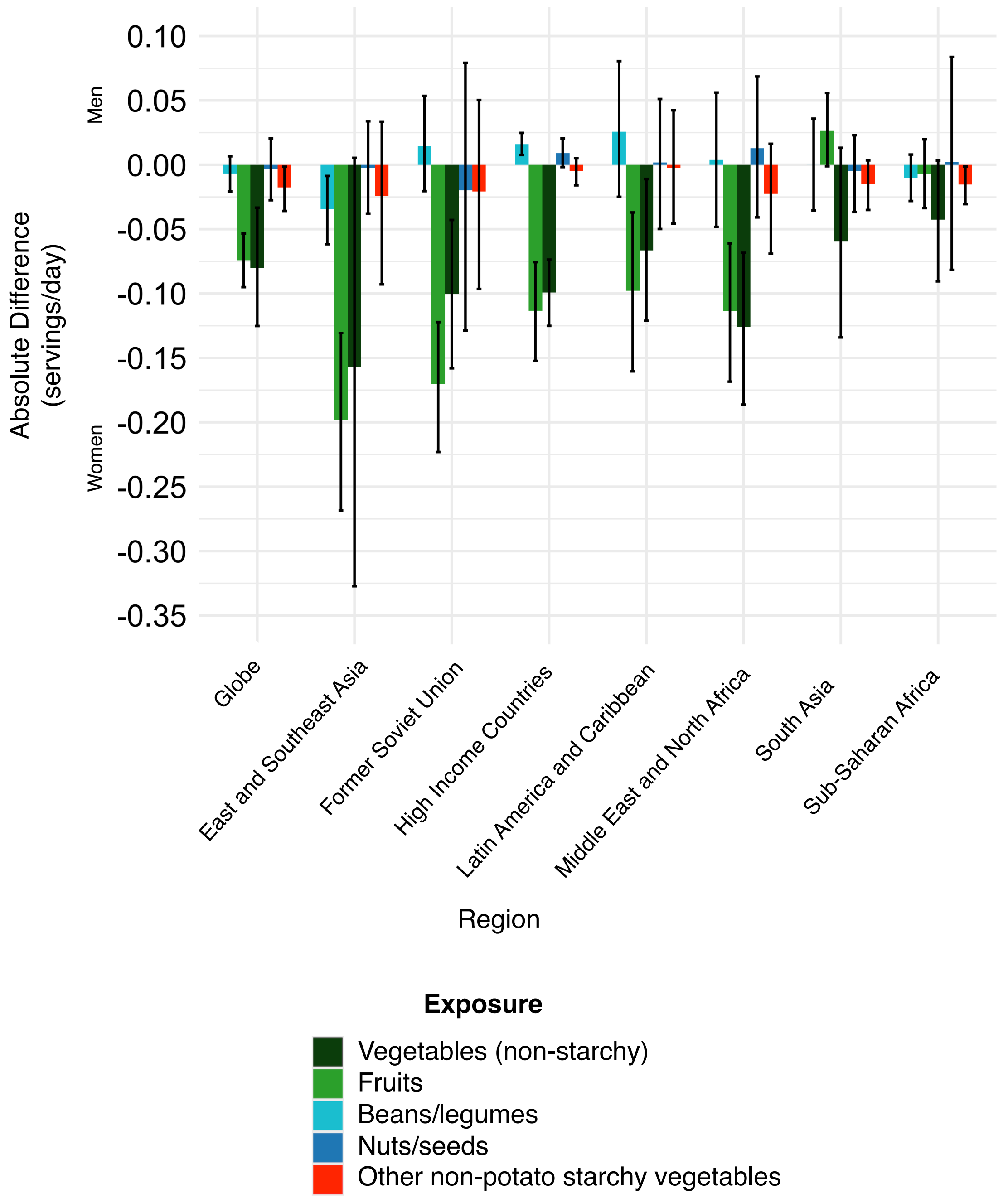


**Supplemental Figure 6: Mean absolute difference between men vs. women by region of plant-based food consumption in youth 0-19 years of age in 2018.** The filled bars represent the absolute mean difference in servings/day in consumption of fruits, non-starchy and other non-potato starchy vegetables, beans/legumes, and nuts/seeds, and the error bars the 95% uncertainty interval. Positive values indicate a greater absolute mean difference consumption in men compared to women. One serving of fruit is equal to 49 g (6-11 months), 75 g (12-24 months), and 100 g (3-19 years). One serving of non-starchy vegetables is equal to 44 g (6-11 months), 50 g (12-24 months), and 100 g (3-19 years). One serving of other starchy vegetables is equal to 42 g (6-11 months), 47 g (12-24 months), and 160 g (3-19 years). One serving of beans/legumes is equal to 24 g (6-11 months), 32 g (12-24 months), and 100 g (3-19 years). One serving of nuts/seeds consumption is equal to 24 g (6-11 months), 32 g (12-24 months), and 28.35 g (3-19 years). All estimates are adjusted to age-specific daily energy intake levels as follows: 700 kcal/day for ages 0-0.9 years, 1,000 kcal/day for ages 1-1.9 years, 1,300 kcal/day for ages 2-5 years, 1,700 kcal/day for ages 6-10 years, 2,000 kcal/day for ages 11-74 years, and 1,700 kcal/day for ages ≥75 years.
