# Supplementary material for: Global, regional and national intake of plant-based foods among youth in 185 countries (1990–2018): findings from the Global Dietary Database
Source: BMJ Glob Health. 2026 Jul 8;11(7):e021543. doi: 10.1136/bmjgh-2025-021543 (PMC13358298; doi:10.1136/bmjgh-2025-021543)
Supplement: Supplementary file 1 [file bmjgh-11-7-s007.docx]

Table S1: Definitions and units of plant-based foods. 7

Table S2: Countries, regions, and super-regions included in GDD 2018 (N=185) 8

Table S3: Survey availability in GDD for 2018. 22

Table S4: Spearman correlation coefficients in children 0-11 months of age in 2018. 23

Table S5: Spearman correlation coefficients in children 12-23 months of age in 2018. 23

Table S6: Spearman correlation coefficients in children 3-4 years of age in 2018. 23

Table S7: Spearman correlation coefficients in children 5-9 years of age in 2018. 24

Table S8: Spearman correlation coefficients in children 10-14 years of age in 2018. 24

Table S9: Spearman correlation coefficients in children 15-19 years of age in 2018. 24

Table S10: Global consumption (servings/day) in children less than 2 years in 2018. 24

Table S11: Global consumption (servings/day) in children 2-19 years in 2018. 25

Table S12: Global consumption (servings/day) in children 0 to 19 years in 2018. 25

Table S13: Regional consumption (servings/day) in children 0-19 years in 2018. 25

Table S14: National consumption (servings/day) in children <2 years in 2018. 27

Table S15: National consumption (servings/day) in children 2-19 years in 2018. 33

Table S16: National consumption (servings/day) in children <1 year in 2018. 39

Table S17: National consumption (servings/day) in children 1-2 years in 2018. 45

Table S18: National consumption (servings/day) in children 3-4 years in 2018. 51

Table S19: National consumption (servings/day) in children 5-9 years in 2018. 57

Table S20: National consumption (servings/day) in children 10-14 years in 2018. 63

Table S21: National consumption (servings/day) in children 15-19 years in 2018. 69

Table S22: Mean absolute difference (servings/day) by sex in youth 0-19 years in 2018. 75

Table S23: Mean percent difference (servings/day) by sex in youth 0-19 years in 2018. 75

Table S24: Mean absolute difference (servings/day) by urban/rural residence in youth 0-19 years in 2018. 75

Table S25: Mean percent difference (servings/day) by urban/rural residence in youth 0-19 years in 2018. 76

Table S26: Mean absolute difference (servings/day) by education in youth 0-19 years in 2018. 76

Table S27: Mean percent difference (servings/day) by education in youth 0-19 years in 2018. 77

Table S28: Mean absolute difference (servings/day) by year comparing 1990 to 2018 in youth 0-19. 77

Table S29: Mean percent differences (servings/day) by year comparing 1990 to 2018 in youth 0-19. 77

**Description of data assessment, standardization, and analysis**

**Data assessment**

Data received from ‘corresponding members’ or from publicly available surveys were checked to confirm survey-level characteristics; dietary intake variables were categorized into GDD dietary factors; necessary unit and format conversions were noted. Data-owners or survey directors were contacted extensively to resolve questions about data quality, categorization, or assessment methods to ensure accuracy and completeness of data prior to analysis.

**Preliminary data checks**

Biostatisticians generated survey description files for each survey including design characteristics, variable lists, and summary statistics for categorical and continuous variables. Research assistants used these description files to assess inclusion of survey level information and demographic variables. Discrepancies between author-reported characteristics and those ultimately included in the dataset were noted for further discussion with the data-owner.

**Categorization of variables into GDD dietary factors**

Reported dietary data were matched to GDD dietary factors. This involved categorizing foods, nutrients, mixed dishes, and regional items into the matched GDD dietary factor, noting cases where variables represented less than the optimal GDD definition. Unit conversions were included as necessary to transform variables into the optimal GDD units.

**Categorization of variables into GDD dietary factors – Food Frequency Questionnaires**

To transform food and beverage data reported from Food Frequency Questionnaires into optimal GDD units, most often grams per day, all categorical variables were standardized into single daily serving units. When ranges of frequencies were provided, the mean of each range was utilized to represent each frequency category. Variables reported in times per week were divided by 7 to calculate the average daily servings. Variables reported in times per month were divided by 30⋅42 (the average number of days in a month) to calculate the average daily servings. In cases where the upper range was open-ended (e.g., “5 or more times per week”), the ranges of the other frequency categories were used to calculate an upper limit. Servings per day were then converted into grams per day by multiplying the number of servings by the author-reported serving sizes or by the GDD standard serving sizes.

**Communication with data owners and creation of data key**

Any questions regarding the data, including those about region-specific diets (e.g., disaggregation of mixed dishes, classification of regional items), survey-level characteristics, and serving sizes for foods and beverages, were communicated to the data owner. After all data questions were answered, research assistants generated a data key outlining all available variables of interest, including demographic and dietary variables. Categorizations of dietary variables were turned into STATA code for clear identification and research assistants flagged the quality assurance checks.

**Converting household data to individual data**

Household-level data were transformed into individual-level data using the Adult Male Equivalent (AME) method. The AME method estimates individual-level intakes by assigning each household member a reference AME based on their age and sex. Household members’ reference AMEs are summed to find total household AME. Each individual’s reference AME is then divided by the total household AME to find individual-level AME. This individual-level AME represents the proportion each individual contributes to the overall household AME. This individual level proportion is multiplied by the household consumption of each food item to estimate individual-level intake.

**Data aggregation**

Using preliminary checking documents provided by research assistants, biostatisticians converted individual-level data into aggregated outputs for each dietary factor stratified by the available demographic variables. Stata version 12 was used to convert all demographic and dietary variables from raw data files to a single data file containing only relevant variables. Missing observations were excluded from the dataset and all variables were recoded to match the GDD demographic and dietary variable coding scheme. Data were then aggregated into demographic strata by age, sex, residence, education, and pregnancy/lactating status. In addition to the single, final data file, supporting files were generated including a summary report, minimum/maximum values for each dietary factor, and group level means, standard deviations, and percentiles of intake. All output files were stored in each survey’s specific folder on the Tufts GDD Box, accessible to all members of the research team.

**Energy adjustment**

We extracted both raw and energy-adjusted data when available. If energy adjustment of individual-level data had not been completed by the data owner, biostatisticians completed energy adjustment at the aggregation stage to age-specific levels using the residual method. This approach was considered the “gold standard.” We adjusted for total energy intake to mitigate the effects of measurement error in data collection, account for differences in energy requirements related to body size, metabolic efficiency and physical activity, and to facilitate comparisons between surveys, age groups, and sexes.

**Total daily energy values by age**

| Age (years) | Daily energy intake (kcal) |
| --- | --- |
| <1 | 700 |
| 1-2 | 1,000 |
| 2-5 | 1,300 |
| 6-10 | 1,700 |
| 11-74 | 2,000 |
| 75+ | 1,700 |

Child and older adult-specific daily energy values were selected using dietary recommendations and mean population ranges from the USA, United Kingdom, and India.

**Energy adjustment corrections**

We initially asked that all data be shared both unadjusted and energy-adjusted to 2,000 kcal, regardless of age category, but retrospectively changed this decision to reflect the age-specific levels. When possible, energy adjustment using the residual method was repeated to reflect these changes. In some cases, this approach was not possible, and thus alternative approaches for energy adjustment correction were taken.

*Energy adjustment correction of aggregate (“stratum-level”) data*

In some cases, data were provided or accessed at the stratum level (i.e., age group, sex, education level, etc.). In these cases, energy adjustment correction depended on whether 2,000 kcal/day-adjusted values had previously been provided by the data owner. If energy-adjusted data had been provided, a simple ratio of the age-specific level to 2,000 kcal was applied post-hoc to convert the value to the correct energy level. If stratum-level data were only provided in an unadjusted format but with corresponding total energy intake, intake was adjusted to the age-specific energy level using the energy density method, in which a simple ratio of reported calorie intake to age-specific level was applied to the unadjusted value. If stratum-level data were provided in age groups which traversed more than one level of age-specific energy intake, a weighted mean daily energy intake was calculated. This weighted mean daily energy level was then used to adjust intake using the ratio readjustment method. If only unadjusted intake was available, the energy density method was used.

*Energy adjustment of data without adjusted values or total energy intake*

In limited cases, individual-level data were not initially energy-adjusted or provided with mean caloric intake data, precluding the use of the gold standard and ratio readjustment methods. In these instances, daily per capita energy availability data from FAO Food Balance Sheets (FBS) were used to inform stratum-level caloric intake. In short, country-year-specific FBS energy data were adjusted using coefficients derived from a multivariate linear regression of GDD input data, FBS data, and both regional and survey-level covariates. Adjusted FBS energy was then corrected to the prescribed energy level by applying a factor of the energy level’s proportion of 2,000 kcal. Unadjusted food and nutrient intake values were then adjusted with this corrected energy intake via the energy density method.

**Quality control**

Data integrity and quality were assessed at each step during survey collection, processing, harmonization, and analyses. Duplicate reviews were performed of recorded survey characteristics, demographic variables, dietary definition classifications, and unit conversions. To assess for outliers and validity (errors) in reported intakes, plausibility thresholds were defined for each dietary factor, both at the individual level and stratum (e.g., group mean) level, based on dietary reference intakes, tolerable upper limits, toxicity ranges, and existing regional data on mean intakes in populations. Any value identified as potentially implausible was reviewed for extraction errors, followed by direct correspondence with the corresponding member or public survey data owners, to detect and correct potential errors. Data remaining implausible after such steps were excluded from final datasets. Results for each dietary factor were further graphed and visually inspected by country, age, sex, dietary assessment method, representativeness, and time, reviewing survey result plausibility and consistency within and across countries.

**Data finalization**

After data has been finalized for inclusion, it is stored within the Access database, which houses information on all surveys, corresponding authors, and survey checking statuses. Aggregated data is collated by dietary factor and prepared for input into the GDD prediction model.

**Protocol for converting FFQ frequency data into GDD servings**

1. Step 1- Standardize the categorical frequency variables to a single daily serving unit
   1. If a range of frequencies is given, take the mean (“Avg”) of the range
   2. If the frequency is presented in times/week, divide by 7 (for days in a week)
   3. If the frequency is presented in times/month, divide by 30·42 (average days in a month)
      1. *Note:* If the category is presented as days/week instead of times/week, assume one serving per day and treat as times/week
      2. Example A) 5-7 days/week = (6 days/week) / (7days/week) = 0·857 servings/day
      3. Example B) 1-3 times/month = (2 times/month) / (30.42 days/month) = 0·066 servings/day
   4. If the upper range is open ended, use the range of the other frequency categories in the survey to create an upper limit and then take the average of that range.
      1. Example: “5 or more times per week” where next lowest level is 2-4 times per week. Assume a range of 5-7 times per week, take the average (6 times per week)/(7 days/week) = 0·857 servings/day
2. Step 2- Convert servings to grams
   1. If available, survey-specific serving sizes were used for conversions.
   2. If survey-specific serving sizes are not available, ask the data owner for usual, country-specific serving sizes.
   3. If data owner does not provide country-specific serving sizes, utilize country-specific serving sizes identified from national agencies (e.g., USDA).
   4. If no country-specific serving sizes are identified, use the GDD standard serving size conversions.

**Common categories of intake and their servings per day conversions**

| Categorical Variable | Calculation | Daily Serving |
| --- | --- | --- |
| Never | 0 | 0 |
| Occasional-Few times/year* | Should capture the range of values between never and the next highest choice based on the data set | *Depends on next level categorization |
| Less than once a month (1-11 times per year) | 1+11=12/2=6 Avg servings/year  6/12 months=0.5 servings/month  0·5/30·42 days | 0·0164 |
| 1-3 times/month | 1+3=4/2=2 Avg servings/month  2/30·42 days | 0·066 |
| 1/week | 1 servings/7 days | 0·143 |
| 2-4 days/week | 2+4=6/2=3 Avg servings/week  3servings/7 days | 0·429 |
| 5-6 days/week | 5+6=11/2= 5.5 Avg servings/week  5·5/7 days | 0·786 |
| 5-7 days/week | 5+7=12/2=6 Avg servings/week  6/7 days | 0·857 |
| 1/day |  | 1 |
| 2-3/day | 2+3=5/2= Avg 2·5 servings | 2·5 |
| 4-5/day | 4+5=9/2= Avg 4·5 servings | 4·5 |

**Common weight conversions**

| **Provided weight** | **Grams** |
| --- | --- |
| 1 Kilogram | 1000 |
| 1 Ounce *Cannot use for fluid ounces | 28 |
| 1 Pound | 454 |

**Standard serving sizes for foods and beverages**

| Dietary Factor | | Reference serving sizes | “Usual” average serving sizes (g/serving) | | |
| --- | --- | --- | --- | --- | --- |
| Variable Code | Variable name | 2003-06 US NHANES (median) | Adults and children older than 2 years | 12-24 months | 6 to 11 months |
| v01 | Fruits^0^ | 110 g per serving | 100 | 75 | 49 |
| v02 | Non-starchy vegetables^0^ | 40 g per serving | 100 | 50 | 44 |
| v04 | Other non-potato starchy^*^ vegetables | - | 160 | 47 | 42 |
| v05 | Beans and legumes^0^ | 86.5 g per serving | 100 | 32 | 24 |
| v06 | Nuts and seeds | 29.75 g per serving | 28.35 | 32 | 24 |
| * Calculated using average of item-specific serving sizes from the USDA Nutrient Database.  ^0^ Calculated using average if both item-specific serving sizes from the USDA Nutrient Database and intake from NHANES 2003-2006 | | | | | |

Table S1: Definitions and units of plant-based foods.

| Dietary factor | Unit | Preferred definition | Alternative definition |
| --- | --- | --- | --- |
| Fruit | g/day | Total fruit intake, including fresh, frozen, cooked, canned, or dried fruit, excluding fruit juices and salted or pickled fruits. | Total fruit intake including fruit juices, nuts/seeds, vegetables, salted/pickled, preserved and processed fruits (jams). |
| Non-starchy vegetables | g/day | Total vegetable intake, including fresh, frozen, cooked, canned, or dried vegetables. This definition excludes salted or pickled vegetables, vegetable juices, starchy vegetables (e.g., potatoes, taro, cassava, manioc,, yucca, corn, peas), and legumes (beans and lentils) | Total vegetable intake including vegetable juices, starchy vegetables, nuts/legumes, nuts/beans, beans/legumes, salted/picked vegetables, and salted/pickled beans/legumes. |
| Other non-potato starchy vegetables | g/day | Total intake of non-potato starchy vegetables, including fresh, frozen, cooked, canned, or dehydrated starchy vegetables. Examples of starchy vegetables include green peas, corn (including corn flour/corn meal), yam, sweet potatoes, taro, plantain, cassava, manioc, tannier (yautia), jicama, and water chestnuts. This definition excludes white potatoes. | Includes starchy fruits or potatoes and starches refined from starchy vegetables. May include non starchy vegetables such as carrots and/or fruits such as mangos, sweet potatoes and hard squashes. |
| Beans and legumes | g/day | Total intake of beans and legumes (beans, lentils) including fresh, frozen, cooked, canned, or dried beans/legumes. This definition excludes peanuts and peanut butter. This definition includes soybeans but excludes soy milk and soy protein. | Includes nuts/seeds, soy protein, soy products, peanuts, and peas. |
| Nuts and seeds | g/day | Total intake of tree nuts (e.g. walnuts, almonds, hazelnuts, pecans, cashews, pistachios), seeds (e.g. sesame seeds, sunflower seeds, pumpkin seeds), and peanuts (including peanut butter). | Includes pulses, beans, legumes and foods primarily (>51%) from nuts or seeds. |

Table S2: Countries, regions, and super-regions included in GDD 2018 (N=185)

| Region | Countries |
| --- | --- |
| **Southeast and East Asia (super-region) (N=24)** | |
| East Asia (N=2) | China, Taiwan |
| Southeast Asia (N=9) | Cambodia, Indonesia, Lao People’s Democratic Republic, Malaysia, Myanmar, The Philippines, Thailand, Timor-Leste, Viet Nam |
| Asia-Pacific high income (N=4) | Brunei Darussalam, Japan, Republic of Korea, Singapore |
| Oceania (N=9) | Fiji, Kiribati, Marshall Islands, Micronesia, Papua New Guinea, Samoa, Solomon Islands, Tonga, Vanuatu |
| **Former Soviet Union (super-region) (N=29)** | |
| Central Asia (N=9) | Armenia, Azerbaijan, Georgia, Kazakhstan, Kyrgyzstan, Mongolia, Tajikistan, Turkmenistan, Uzbekistan |
| Central Europe (N=13) | Albania, Bosnia and Herzegovina, Bulgaria, Croatia, Czech Republic, Hungary, Montenegro, Poland, Romania, Serbia, Slovakia, Slovenia, The former Yugoslav Republic of Macedonia |
| Eastern Europe (N=7) | Belarus, Estonia, Latvia, Lithuania, Republic of Moldova, Russian Federation, Ukraine |
|  | **Latin America and Caribbean (super-region) (N=32)** |
| Caribbean (N=15) | Antigua and Barbuda, Bahamas, Barbados, Belize, Cuba, Dominica, Dominican Republic, Grenada, Guyana, Haiti, Jamaica, Saint Lucia, Saint Vincent and the Grenadines, Suriname, Trinidad and Tobago |
| Andean Latin America (N=3) | Bolivia (Plurinational State of), Ecuador, Peru |
| Central Latin America (N=9) | Colombia, Costa Rica, El Salvador, Guatemala, Honduras, Mexico, Nicaragua, Panama, Venezuela (Bolivarian Republic of) |
| Southern Latin America (N=3) | Argentina, Chile, Uruguay |
| Tropical Latin America (N=2) | Brazil, Paraguay |
| **Middle East and Northern Africa (super-region) (N=20)** | |
| Western Europe (N=1) | Israel |
| North Africa and Middle East (N=18) | Algeria, Bahrain, Egypt, Iran (Islamic Republic of), Iraq, Jordan, Kuwait, Lebanon, Morocco, Occupied Palestinian Territory, Oman, Qatar, Saudi Arabia, Syrian Arab Republic, Tunisia, Turkey, United Arab Emirates, Yemen |
|  | **South Asia (super-region) (N=8)** |
| South Asia (N=6) | Afghanistan, Bangladesh, Bhutan, India, Nepal, Pakistan |
| Southeast Asia (N=2) | Maldives, Sri Lanka |
| **Sub-Saharan Africa (super-region) (N=48)** | |
| Central Sub-Saharan Africa (N=6) | Angola, Central African Republic, Congo, Democratic Republic of Congo, Equatorial Guinea, Gabon |
| Eastern Sub-Saharan Africa (N=17) | Burundi, Comoros, Djibouti, Eritrea, Ethiopia, Kenya, Madagascar, Malawi, Mauritius, Mozambique, Rwanda, Seychelles, South Sudan, Sudan, Uganda, United Republic of Tanzania, Zambia |
| Southern Sub-Saharan Africa (N=6) | Botswana, Lesotho, Namibia, South Africa, Swaziland, Zimbabwe |
| Western Sub-Saharan Africa (N=19) | Benin, Burkina Faso, Cameroon, Cape Verde, Chad, Cȏte d’Ivoire, The Gambia, Ghana, Guinea, Guinea-Bissau, Liberia, Mali, Mauritania, Niger, Nigeria, Săo Tomé and Príncipe, Senegal, Sierra Leone, Togo |
| **High-Income Countries (super-region) (N=24)** | |
| Australasia (N=2) | Australia, New Zealand |
| Western Europe (N=20) | Austria, Belgium, Cyprus, Denmark, Finland, France, Germany, Greece, Iceland, Ireland, Italy, Luxembourg, Malta, Netherlands, Norway, Portugal, Spain, Sweden, Switzerland, United Kingdom |
| North America high income (N=2) | Canada, United States of America |
| ^We included countries: 1) classified as United Nations (UN) Member States, 2) included in the United Nations Food and Agriculture Food Balance Sheets database, or 3) included in the World Bank Gross Domestic Product database.^ | |
|  | |

# **Covariates**

**Covariate identification**

Country- and time-specific covariate data were identified from various sources to further inform model predictions. These data supplement individual-level dietary intake data, particularly in countries for which these inputs are limited. We consulted experts and conducted comprehensive searches of publicly available databases to identify >800 covariates. We prioritized approximately 400 covariates for testing:

| Data source | Year(s) |
| --- | --- |
| UN FAO food balance sheets | 1980 - 2018 |
| Harvard Global Expanded Nutrient Supply (GENuS) | 1980 - 2011 |
| Principal component analysis of FAO and GENuS data | 2013 |
| Euromonitor fat and oils sales data | 1998 - 2018 |
| World Bank Gross Domestic Product (GDP) | 1980 - 2018 |
| World Bank unemployment rate | 1980 - 2015 |
| World Bank gini coefficient | 1980 - 2015 |
| World Bank poverty rate | 1980 - 2015 |
| Barro Lee years of schooling | 1980 - 2010 |
| World Bank precipitation | 1982 - 2014 |
| CIA Factbook latitude | N/A |
| CIA Factbook land area | N/A |
| CIA Factbook coastline ratio | N/A |

We conducted principal component analysis (PCA) using the 'princomp' function in R separately for: 1) 23 grouped FAO food balance sheet (FBS) foods, beverages, and energy, 2) 142 GENuS foods, and beverages, and 3) 19 GENuS nutrients and energy. The first four components from each PCA were considered for covariate testing.

**Covariate imputation and truncation**

If covariate data were missing for some (but not all) years of a given country, we used linear interpolation to ﬁll in those years. Covariate data sources that ended before 2018 were imputed using a moving average of the three most recent values to obtain values for all covariates through the year 2018. Region-level means were assigned to countries for which entire covariates were missing. To assess validity of the imputations, we imputed non-missing values with the same model and visually compared observed versus imputed values via scatter plots.

The GDD prediction model operates on the natural log scale (except for dietary factors measured as proportions), including the covariate data. To reduce the risk of having very small values for covariates with a broad range of values on the log scale having an outsized influence on modeled estimates, we truncated covariate data on the non-transformed scale using the following rules:

For covariates with a 95th percentile value

1. >3·5: Truncate values <0·5 to 0·5
2. ≥1 and ≤3·5: Truncate values <0·1 to 0·1
3. <1: No truncation

**Covariate testing**

For each dietary factor, we calculated the correlations between covariates and original survey-level stratified mean dietary intakes, and we selected up to 10 covariates for model inclusion, favoring those with the highest correlations, a mix of food/nutrients and other covariates, and sensible links to the dietary factor.

Each of the covariates identified in the correlation stage (maximum 10 covariates) and the four PCA components were then included in a stepwise regression (entry point of p<·299 and exit point of p<·30) to test for inclusion in GDD models. These stepwise regressions resulted in three nested versions of the GDD model per diet factor:

1. **Base model:** Closest diet factor proxy from FAO or GENuS (1-2 covariates per model)
2. **Restricted model:** All covariates with p<0·1 from the results of the stepwise regression plus base model covariate(s).
3. **Inclusive model:** All covariates from the results of the stepwise regression plus base model covariate(s).

For each dietary factor, five-fold cross-validation was used to compare model fit for the three versions of the GDD model. Data were split into five partitions at the survey level: four partitions making up the training dataset, and the remaining segment as the testing data. The models were fit to the training set, and resulting outputs were compared to training set to assess model fit via calculating the expected log predictive density (ELPD)^1^. This was repeated five times so that each partition was used once as the training set.

Final model selection and included covariates by dietary factor.

| Dietary factor | Covariates | |  |
| --- | --- | --- | --- |
| Fruit | FAO fruits | |  |
| Non-starchy vegetables | FAO vegetables |  |  |
| Other non-potato starchy vegetables | FAO starchy roots, FAO vegetable oils, World bank Gini coefficient, Comp 3 | | |
| Beans and legumes | FAO pulses | | |
| Nuts and seeds | FAO nuts and seeds | | |

**References**

1. Vehtari, A., Gelman, A. & Gabry, J. Practical Bayesian model evaluation using leave-one-out cross-validation and WAIC. *Stat Comput* **27,**1413–1432 (2017). https://doi.org/10.1007/s11222-016-9696-4.

# **GDD Prediction Model**

1. **Overview**

The GDD prediction model aims to estimate mean intake of 53 dietary factors in 185 countries, by country/year/age/sex/urbanicity/education, by synthesizing survey mean intake data from sources of varying quality. The Bayesian multilevel framework has some advantageous properties that are appealing for our purposes. Namely,

- - “Shrinkage” of parameter estimates towards an overall mean. For example, mean estimates for data sparse countries are pulled towards the region mean, allowing for more reasonable estimates for countries with potentially unreliable data.
  - Intuitive framework for predicting means (with uncertainty bounds) for countries with no available data.
  - Ability to include prior knowledge about intake through priors
  - Allows for model flexibility and complexity often not granted in similar frequentist approaches due to difficulty in optimization.

1. **Hierarchical nature of the data**

Survey data collected across the globe have an inherently nested hierarchical structure which makes a multilevel approach to modeling the data appealing. The hierarchical structure of the data we assumed was as follows: countries were nested in super-regions, which are nested in the globe. Our model assumed that the super-region means were distributed log-normally around the global mean, and that country means were distributed log-normally around their respective super-region means. Using this structure allowed us to borrow strength across units, a concept commonly known as “partial pooling” in the Bayesian literature. In partial pooling, each country’s mean estimate borrows from the other countries’ data within the region, resulting in shrinkage of the country mean estimate towards the region mean. The less informative the data was for a particular country, the more pooling there is.

Our model used the following seven super-regions:

- 1. Southeast and East Asia
  2. FSU (Central/Eastern Europe and Central Asia)
  3. HIC (High Income Countries)
  4. LAC (Latin America and Caribbean)
  5. MENA (Middle East and North Africa)
  6. SAARC (South Asia)
  7. SSA (Sub-Saharan Africa)

1. **Description of model**

Fundamentally, our model was a Bayesian model on the log-means of intake with a nested hierarchical structure (clusters countries within super-regions and super-regions within the globe), assuming exchangeability between countries and super-regions after accounting for covariates. To this structure, we added sex, urban/rural, education, and non-linear age effects (also within a nested hierarchical structure), survey and country-level covariates, and overdispersion on study-level variance to account for non-sampling variation. It borrowed heavily from models presented by Finucane et al.^1^ and Flaxman et al.^2^. For dietary factors that were measured as proportions of energy intake, we used *−log*(*−log*(*y*)) as the link function instead of *log*(*y*).

Below we provide a full mathematical description of the model, with detailed descriptions for each component, but first, we present some notation:

- 1. Subscript notation:
     1. h: age/sex/educ/urbanicity group
     2. i: study
     3. j: country
     4. k: super-region
  2. Superscript notation:
     1. c: country
     2. s: super-region
     3. g: globe

**The model**

$$f\left( y_{h,i} \right)\sim N(a_{j}+ b_{1j}{sex}_{h,i}+b_{2j}u_{h,i}+b_{3j}{educ}_{h,i}+y_{j}\left( z_{h,i} \right)+X_{i}\beta, {SE}_{h,i}^{2}+\tau_{i}^{2})$$

Where,

## f (y) ⇐−log(−log(y)) for dietary factors measured as proportions, log(y) otherwise

## y_h,i_ ⇐ mean intake level for stratum h in study i

## $a_{j}$ ⇐ country-specific intercept

## $b_{1j}$ ⇐ country-specific difference between females and males

## sex_h,i_ ⇐ variable indicating whether the y_h,i_ corresponds to an all male group (0), all female group (1), or mixed (0.5)

## $b_{2j}$ ⇐ region-specific difference between urban and rural

## u_h,i_ ⇐ variable indicating whether the y_h,i_ corresponds to an all rural group (0), all urban group (1),or mixed (% urban)

## $b_{3j}$ ⇐ region-specific education effect

## u_i_ ⇐ two variables indicating whether y_h,i_ corresponds to low education (defined to be 6 years or less of schooling if mixed), proportion of low education, and high education

## $\gamma_{j}$ ⇐ non-linear age-trend for region j

## $z_{h,j}$ ⇐ midpoint age of stratum h in study

## X_i_β ⇐ study + country level covariate effects

## $\mathrm{SE}_{h,i}^{2}$⇐ standard error of f (y_h,i_) (estimated via delta method)

## $\tau_{i}^{2}$ ⇐ overdispersion parameter for study i

## **Intercept, sex differences, education differences, and urban/rural differences**

We fit a multi-level model with 3 levels (countries nested in super-regions nested in the globe) for intercepts and sex differences, and 2 levels (super-regions nested in the globe) for age pattern, education differences, and urban/rural differences. *a_j_* refers to the intercept for country *j*, *b*_1_*_j_* refers to the country specific sex effect, *b*_2_*_j_* refers to the country specific urban effect, and *b*_3_*_j_* refers to the country specific education effect. *a^g^* and *b^g^* correspond to global intercept and effects while *a^s^*, *b^s^* denote super-region specific random effects and *a^c^*, *b^c^* denote country specific random effects. *κ^c^* and *κ^s^* are the between-country and between-super-region variance, respectively, for their respective model components. Note that the model assumes between country variance is the same across all super-regions, and that education, urban/rural differences and age patterns are assumed to be the same for countries within a super-region.

Mathematically, this can be described as follows:

$a_{j}= a_{j}^{c}+ a_{k[j]}^{s}+a^{g}$

$$b_{1j}= b_{1j}^{c}+ b_{1k[j]}^{s}+b_{1}^{g}$$

$$b_{2j}= b_{2k[j]}^{s}+ b_{2}^{g}$$

$$b_{3j}= b_{3k[j]}^{s}+ b_{3}^{g}$$

$$a_{j}^{c} \sim N\left( 0, k_{a}^{c} \right), b_{1j}^{c} \sim N\left( 0, k_{1b}^{c} \right),$$

$$a_{k}^{s} \sim N\left( 0, k_{a}^{s} \right), b_{1k}^{s} \sim N\left( 0, k_{1b}^{s} \right), b_{2k}^{s} \sim N\left( 0, k_{2b}^{s} \right), b_{3k}^{s} \sim N(0, k_{3b}^{s})$$

Weakly informative priors are used for the hyperparameters: half-Normal (0, 0.5) for the $k$ parameters, Normal (0,1) for a^g^, $b_{1}^{g}, b_{2}^{g}$ , and $b_{3}^{g}$ (input data are standardize to the standard normal scale to ensure priors are sensible for all dietary factors and to increase computational stability).

## **Covariate effects**

There were two survey-level covariate effects included in the model to explain potential bias from a survey: survey type and food definition. There were four main types of diet surveys included as covariates in the model: short-term recalls (single or multiple); food frequency questionnaires (FFQs); household budget/intake surveys; and DHS (Demographic Health Survey) questionnaires. Only the recall was considered the “gold standard” with regards to estimating the mean unbiasedly. All surveys reported using the optimal definition for sugar-sweetened beverages (SSBs).We included country-year specific predictors in the model (e.g., food availability (FAO food balance sheets or Global Expanded Nutrient Supply (GENuS) model). We assumed their relationship to f (y) was linear, and that the relationships were independent of location (not super-region dependent, or country dependent).

$$X_{i}\beta=\beta_{1}I\{X_{i}^{\mathrm{diet}}=FFQ\}+$$

$$\beta_{2}I\{X_{i}^{\mathrm{diet}}=Household survey\}+$$

$$\beta_{2}I\{X_{i}^{\mathrm{diet}}=DHS\}+$$

$$\beta_{3}I\{X_{i}^{\mathrm{metric}}=alternative \}+$$

$$X_{i[j]}^{country-year predictions} \beta_{c}$$

For survey level-covariates, we used a prior of Normal(0, 0.35). The prior for βc parameters depended on the dietary factor. For many dietary factors, we only used 1 or 2 country-level covariates, all from FAO or GENuS. For these variables, we had a very strong prior belief that they should be strongly correlated with the outcome of interest (e.g., log(sugar and sweeteners availability from FAO) should be strongly positively correlated with log(SSBs intake)). In these cases, we used a highly informative prior of N(1, 0.1). For other dietary factors, either no such variable existed, or other country-year level predictors were also included and do not warrant such a high degree of certainty in a strong relationship. In these cases, we used a much weaker prior of N(0, 0.5).

## **Age trend**

For many surveys, intake was not linearly associated with age. We modelled age using restricted cubic splines with 4 knots at *k*_1_, *k*_2_, *k*_3_, *k*_4_, corresponding to ages 5, 20, 50 and 65, respectively, after standardization:

$$Y_{j\left[ i \right]}\left( zh \right)=y_{1j\left[ i \right]}z_{h}+y_{2j\left[ i \right]}S_{1}\mathbf{+}y_{3j\left[ i \right]}S_{2}$$

where,

$$S_{1}=(Z_{h}- k_{1})^{3}- \frac{k_{4}- k_{1}}{k_{4}- k_{3}}(z_{h}- k_{3})^{3}+ \frac{k_{3}- k_{1}}{k_{4}- k_{3}}(z_{h}- k_{4})^{3}$$

$$S_{2}=(Z_{h}- k_{2})^{3}- \frac{k_{4}- k_{2}}{k_{4}- k_{3}}(z_{h}- k_{3})^{3}+ \frac{k_{3}- k_{2}}{k_{4}- k_{3}}(z_{h}- k_{4})^{3}$$

As with the urban and education effect parameters, we used 2 levels of hierarchy for the age-trend:

$$Y_{1j\left[ i \right]}=y_{1k[j]}^{s}+ y_{1}^{g}$$

$$Y_{2j\left[ i \right]}=y_{2k[j]}^{s}+ y_{2}^{g}$$

$$Y_{3j\left[ i \right]}=y_{3k\left[ j \right]}^{s}+ y_{3}^{g}$$

$$y_{1k}^{s} \sim N\left( 0, k_{1y}^{s} \right), y_{2k}^{s} \sim N\left( 0, k_{2y}^{s} \right),y_{3k}^{s} \sim N\left( 0, k_{3y}^{s} \right)$$

Weakly informative distributions were used for hyper-prior parameters: Half-Normal(0, 0.5) for the

*κ* parameters and Normal(0, 0.35) for the *γ^g^* parameters.

## **Overdispersion**

An additional variance component was added to each study to allow the model to account for non- sampling variation due to survey-level error (from imperfect study design and quality). This additional variance component was modeled in such a way to reflect our expectation that surveys that are less likely to represent the true mean (but not necessarily biased) were more variable. Sources of this non-sampling variation accounted for included surveys not being nationally representative, surveys not being stratified by sex, urban/rural or education, and surveys that used large age groupings (>10 years). We also added an additional constraint to ensure local surveys were considered more variable than regional surveys.

Thus,

$$\tau_{i}^{2}=exp( \phi_{intercept}+ \phi_{regional}I\left( X_{i}^{rep}=regional \right)+ \phi_{local}I\left( X_{i}^{rep}=local \right)$$

$${+ \phi}_{agerange}I\left( X_{i}^{AgeRange}>10 \right)$$

${+ \phi}_{sex}I\left( X_{i}^{sex}=both \right) {+ \phi}_{urban/rural}I((X_{i}^{urban/rural}=both) or (X_{i}^{educ}=all))$

with the constraints $\phi_{regional}^{2}< \phi_{local}^{2}$, and all $\phi>0$except $\phi_{intercept}$. We use a prior of Normal (-2.5, 1) for $\phi_{intercept}$ to reflect our a priori belief than an “ideal” survey that is both fully stratified and nationally representative should have minimal overdispersion. For all other $\phi$ parameters, we used a prior of Normal (0, 0.5).

## **Computation**

We fit each model using STAN^3,4^ through rstan^5^, using the No-U-turn sampler (NUTS)^6^, a variant of Hamiltonian Monte Carlo^7^. We used 4 chains of 2000 iterations each, treating the first 1000 iterations of each chain as warm up, for a total of 4000 Monte Carlo iterations to define our posterior distributions.

## **Predictions**

The model described above was ultimately used to provide predictive distributions of mean intake for each dietary factor by country-year and subgroup. Note that the model specified *g*(*yh,i*[*j*]) of

subgroup *h* in survey *i* from country *j* as a linear combination of model parameters and survey- year-subgroup specific information:

$$a_{j}+ b_{1j}{sex}_{h,i}+ b_{2s}u_{h,i}+ b_{3s}{educ}_{h,i}+ \gamma_{s}{(z}_{h,i})+ X_{i}\beta$$

where we had posterior distributions for model parameters *a_j_*, *b_j_*, *b*_2_*_s_*, *b*_3_*_s_*, *γ_s_* and *β*. To obtain a predictive distribution for subgroup *h* in country *j*, we calculated:

$$\mu_{hj}= g^{-1}(a_{j}+ b_{1j}{sex}_{h,j}+ b_{2s}u_{h,j}+ b_{3s}{educ}_{h,j}+ \gamma_{s}{(z}_{h,j})+ X_{j}^{country-year predictors}\beta_{c})$$

for each draw of our posterior distributions. Because we are interested in country-specific means, we did not use survey specific parameters in our predictions. For countries with no survey data, we did not have a posterior distribution of *a_j_*. To get the predictive distributions for these countries in such a way that properly accounts for the variation of mean intake between countries within a region, we replaced *a_j_* and *_b1j_* with *a^*^_j_* and *b^*^1j* where *a^*^_j_ ∼ N* (*ak*[*j*]*, κ^c^_a_*) and *b^*^_1j_ ∼ N* (*b*1*k*[*j*]*, κ^c^_1b_*). Here, *ak*[*j*] and *b*1*k*[*j*] are super-region-level intercepts and sex effects corresponding to country *j*, and *κ^c^* and *κ b*1*k*[*j*] are super-region-level intercepts and sex effects corresponding to country *j*, and *κ^c^_a_* and *κ^c^_1b_* are the between-country variances for intercept and sex effects, respectively. In other words, each posterior draw for the super-region-level parameter and it’s corresponding between-country variance parameter generated a unique normal distribution for that draw, and we took a one sample draw from each of these distributions to generate the predictive distribution of that parameter for an unknown country in that region. Note that the uncertainty around the super-region level parameter and between country variance propagate into the predictive distribution for the mean.

For some dietary factors, there were entire super-regions with no data. For those super-regions, predictive distributions for *b*_2_*_s_*, *b*_3_*_s_*, and *γ_s_* were obtained in a similar way, generating a normal distribution for each draw from the global level parameter and between region variance parameter and sampling from that. For *a_j_* and *b*_1_*_j_*, we needed to account for between-super-region variance and the between country variance. Therefore, taking the intercept as an example, for each posterior draw, we sampled from *N* (*a^g^, κ^c^_a_* + *κ^s^_a_*). Note that this is equivalent to drawing a sample region mean from *N(a^g^, k^s^_a_*) then using that sample as mean and *k^c^_a_* as variance to form a normal distribution to sample country mean from.

**References**

1. Finucane MM, Stevens GA, Cowan MJ, et al. National, regional, and global trends in body-mass index since 1980: systematic analysis of health examination surveys and epidemiological studies with 960 country-years and 9•1 million participants. *Lancet* 2011; 377(9765):557–567.
2. Flaxman AD, Vos T, Murray C. An integrative metaregression framework for descriptive epidemiology. University of Washington Press, 2015.
3. Carpenter B, Gelman A, Hoffman MD, et al. Stan: A probabilistic programming language. *Journal of statistical software* 2017; 76(1).
4. Stan Development Team. Stan modeling language users guide and reference manual, version 2.27, 2021.
5. Stan Development Team. RStan: the R interface to Stan, 2019. R package version 2.19.2.
6. Hoffman M, Gelman A, et al. The no-u-turn sampler: adaptively setting path lengths in hamiltonian monte carlo. *Journal of Machine Learning Research* 2014; 15(1):1593–1623.
7. Neal, R. Mcmc using hamiltonian dynamics. In Brooks S, Gelman A, Jones GL, et al., Handbook of Markov Chain Monte Carlo, pages 116–162. Cambridge University Press, Cambridge, 2011.

## **Varying slopes modeling structure**

- - Our extensive work to identify surveys and model intakes led to recognition and the finding that, for certain dietary factors, the available global data and model were insufficient to accurately model differences in intakes by jointly stratified by country, age, sex, education level, and urban/rural status while also modeling differences in intakes over time.
  - For countries without multiple comparable dietary surveys over time (the majority of nations worldwide), trends over time are largely determined by the strength of the relationship between the best available covariates (often variables from FAO food balance sheets or associated GENuS variables) and the raw survey data. For certain dietary factors, this relationship was sufficiently robust to allow modeling of all joint demographic strata and time trends. By reviewing extensive time trends plots for individual dietary factors and nations, dietary factors with a model beta coefficient ≥0·4 with their corresponding FAO/GENuS covariate were identified as having a reasonable statistical relationship to capture both all demographic strata differences and time trends. For others (FAO/GENuS beta coefficient<0·4), time trends were modeled using a second, separate Bayesian model.
  - This second Bayesian model assessed the country-specific associations over time of the survey data for each dietary factor with its corresponding FAO/GENuS covariate. The model incorporated country-level intercepts and slopes, along with their correlation that is estimated across countries. Input data were the same stratified survey data as for the GDD Core model and including dietary assessment method as a covariate. This time component model did not separately estimate differences by age, sex, education, or urban/rural status, but focused on the relationships with FAO/GENuS over time. In sensitivity analyses, age and sex were included as main effects (not varying by country or region) but were found to not qualitatively alter the parameter estimates for the relationship of a country’s dietary intake data with its FAO/GENuS data. Thus, including these demographics did not largely affect the time-varying predictions. This model is commonly referred as a varying slopes model structure and leverages two-dimensional partial pooling between intercepts and slopes to regularize all parameters and minimize overfitting risk^1-3^. Predictions with the varying slopes model take into account a country-specific intercept and slope when the country has dietary factor data and use the global intercept and slope for countries where data are not available. Time effects were predicted separately for each year including 1990, 1995, 2000, 2005, 2010, 2015, and 2018.
  - For each country and dietary factor, the country-specific time-trend central predictions from the varying slopes models were used to generate a country-year specific adjustment scaling factor, one for each year of 1990, 1995, 2000, 2005, 2010, 2015, and 2018, compared to the reference of one of these years as determined by the median year of that country’s survey data (or 2005 if no country data). This scaling factor, determined by taking the ratio of the predicted dietary intake for that year as compared to the reference year, was multiplied by the country-year posterior predictions from the fully stratified, Core GDD model to determine a time-adjusted final estimate for each stratum.
  - To be conservative, this varying slopes adjustment (scaling factor) was only used for dietary factors and countries meeting all of the following criteria: at the model level, (a) FAO/GENuS beta coefficient<0.4 in the Core GDD model; and (b) availability of a closely corresponding FAO/GENuS covariate (e.g., dietary survey vitamin A intake vs. GENuS vitamin A); and at the country-level, (c) identification of a positive relationship (coefficient or slope) between the national survey data and FAO/GENuS covariate in the varying slopes model; and (d) to minimize implausible results at the country level, no more than a 3-fold difference between the ratio of the country’s range of predicted intake between 1990-2018 divided by the ratio of the country’s range of FAO/GENuS values over that same time period.
  - Among 53 evaluated dietary factors in the GDD, 29 were modeled and incorporated time adjustment using this Bayesian varying slopes model. The other dietary factors were not because (in order of criteria applied) 11 did not have any closely corresponding FAO/GENuS variable (e.g., dietary iodine), 8 had an FAO/GENuS beta coefficient in Core GDD Core Model of at least 0·4, and 4 were unable to complete sampling for the varying-slopes model (i.e., the MCMC chains did not finalize, independent of parameterization). One additional dietary factor, vitamin B9, with a borderline FAO/GENuS beta (0·34) was also not further scaled based on adequate qualitative characteristics of the observed time trends in the GDD Core Model.

A measurement error, varying slopes model that accounts for dietary assessment method, using standardized log-intakes for all dietary factors except those reported in percent energy:

| $Y_{obs,i} \sim Normal(Y_{true,i} , \mathrm{DE}_{SE,i})$ | [distribution for observed intake, Y_obs_, including measurement error associated with the stratum estimate, DE_SE,i_] |
| --- | --- |
| $Y_{true,i} \sim Normal(\mu_{i} , \sigma)$ | [distribution for true strata intake Y] |
| $\mu_{i}= \alpha_{country[i]}+\beta_{country[i]}*FAO+M_{method[i]}$ | [linear equation for the average intake; Each country receives its own intercept and slope while also accounting for dietary assessment method] |
| $\left[ \begin{matrix} \alpha_{\mathrm{country}} \\ \beta_{\mathrm{country}} \end{matrix} \right]\sim MVNormal \left( \left[ \begin{matrix} \alpha\\ \beta\end{matrix} \right] , S \right)$ | [population of varying effects] |
| $S= \left( \begin{matrix} \sigma_{\alpha} & 0 \\ 0 & \sigma_{\beta} \end{matrix} \right)R\left( \begin{matrix} \sigma_{\alpha} & 0 \\ 0 & \sigma_{\beta} \end{matrix} \right)$ | [construct covariance matrix] |

With hyperpriors that define the adaptive varying effects and effects for dietary assessment:

| $\alpha\sim Normal(0 , 1)$ | [prior for average intercept] |
| --- | --- |
| $\beta\sim Normal(1 , 0.11)$ | [prior for average slope] |
| $M[method] \sim Normal(0 , 0.2)$ | [prior for method effect] |
| $\sigma\sim Halfnormal (0 ,0.5)$ | [prior for stddev within countries] |
| $\sigma_{\alpha} \sim Halfnormal (0 ,0.5)$ | [prior for stddev among intercepts] |
| $\sigma_{\beta} \sim Halfnormal (0 ,0.5)$ | [prior for stddev among slopes] |
| $R \sim LKJcorr (2)$ | [prior for correlation matrix] |

**References**

1. Gelman, A. and L. Pardoe, *Bayesian measures of explained variance and pooling in multilevel (hierarchical) models.* Technometrics, 2006. **48**(2): p. 241-251.

2. McElreath, R., *Statistical Rethinking: A Bayesian Course with Examples in R and Stan.* Statistical Rethinking: A Bayesian Course with Examples in R and Stan, 2016: p. 1-464.

3. Wagner, T., et al., *Using Multilevel Models to Quantify Heterogeneity in Resource Selection.* Journal of Wildlife Management, 2011. **75**(8): p. 1788-1796.

Table S3: Survey availability in GDD for 2018.

| Region (Total no. of surveys) | Number of surveys (% nationally or sub-nationally representative) | Total sample size of surveyed subjects | No. of surveys, by dietary assessment method | | | |
| --- | --- | --- | --- | --- | --- | --- |
|  |  |  | 24-hour recall | FFQ | DHS | Household budget survey |
| **Fruit (N=806)** | | | | | | |
| Southeast and East Asia | 100 (93%) | 1,070,116 | 34 | 61 | 12 | 0 |
| Former Soviet Union | 140 (99%) | 626,952 | 18 | 84 | 9 | 30 |
| High Income Countries | 213 (97%) | 1,293,639 | 60 | 122 | 0 | 37 |
| Latin America and the Caribbean | 85 (92%) | 526,599 | 11 | 56 | 25 | 0 |
| Middle East and North Africa | 57 (89%) | 355,848 | 5 | 48 | 11 | 0 |
| South Asia | 26 (79%) | 639,364 | 8 | 14 | 11 | 0 |
| Sub-Saharan Africa | 144 (96%) | 697,376 | 12 | 56 | 82 | 0 |
| Overall | **765 (95%)** | **5,209,894** | **148** | **441** | **150** | **67** |
| **Vegetables (non-starchy) (N=792)** | | | | | | |
| Southeast and East Asia | 99 (94%) | 1,046,251 | 34 | 60 | 11 | 0 |
| Former Soviet Union | 138 (99%) | 627,484 | 19 | 84 | 6 | 30 |
| High Income Countries | 215 (97%) | 1,313,012 | 63 | 121 | 0 | 37 |
| Latin America and the Caribbean | 82 (93%) | 512,140 | 11 | 54 | 23 | 0 |
| Middle East and North Africa | 55 (89%) | 340,389 | 4 | 48 | 10 | 0 |
| South Asia | 26 (81%) | 622,434 | 8 | 13 | 11 | 0 |
| Sub-Saharan Africa | 142 (98%) | 673,679 | 10 | 56 | 79 | 0 |
| Overall | **757 (96%)** | **5,135,389** | **149** | **436** | **140** | **67** |
| **Other non-potato starchy vegetables (N=239)** | | | | | | |
| Southeast and East Asia | 31 (89%) | 371,925 | 7 | 16 | 12 | 0 |
| Former Soviet Union | 15 (100%) | 28,565 | 6 | 0 | 9 | 0 |
| High Income Countries | 25 (93%) | 149,616 | 23 | 4 | 0 | 0 |
| Latin America and the Caribbean | 37 (88%) | 343,701 | 7 | 8 | 27 | 0 |
| Middle East and North Africa | 11 (73%) | 123,587 | 1 | 4 | 10 | 0 |
| South Asia | 11 (92%) | 130,252 | 3 | 2 | 7 | 0 |
| Sub-Saharan Africa | 90 (97%) | 529,422 | 8 | 1 | 84 | 0 |
| Overall | **220 (92%)** | **1,677,068** | **55** | **35** | **149** | **0** |
| **Beans and legumes (N=380)** | | | | | | |
| Southeast and East Asia | 41 (85%) | 660,177 | 13 | 24 | 11 | 0 |
| Former Soviet Union | 46 (98%) | 286,986 | 11 | 1 | 5 | 30 |
| High Income Countries | 98 (94%) | 720,547 | 56 | 12 | 0 | 36 |
| Latin America and the Caribbean | 40 (91%) | 372,481 | 10 | 12 | 22 | 0 |
| Middle East and North Africa | 16 (73%) | 115,378 | 4 | 11 | 7 | 0 |
| South Asia | 18 (78%) | 529,100 | 8 | 4 | 11 | 0 |
| Sub-Saharan Africa | 88 (96%) | 504,890 | 12 | 4 | 76 | 0 |
| Overall | **347 (91%)** | **3,189,559** | **114** | **68** | **132** | **66** |
| **Nuts and seeds (N=267)** | | | | | | |
| Southeast and East Asia | 38 (88%) | 708,453 | 31 | 11 | 1 | 0 |
| Former Soviet Union | 51 (100%) | 288,919 | 12 | 9 | 1 | 29 |
| High Income Countries | 120 (98%) | 804,672 | 55 | 32 | 0 | 36 |
| Latin America and the Caribbean | 12 (80%) | 171,705 | 8 | 5 | 2 | 0 |
| Middle East and North Africa | 11 (69%) | 65,003 | 4 | 8 | 4 | 0 |
| South Asia | 4 (57%) | 143,464 | 6 | 0 | 1 | 0 |
| Sub-Saharan Africa | 9 (75%) | 27,488 | 8 | 1 | 3 | 0 |
| Overall | **245 (92%)** | **2,209,704** | **124** | **66** | **12** | **65** |

Table S4: Spearman correlation coefficients in children 0-11 months of age in 2018.

|  | Fruit | Vegetables (non-starchy) | Other non-potato starchy vegetables | Beans and legumes | Nuts and seeds |
| --- | --- | --- | --- | --- | --- |
| Fruit | 1.00 | 0.71 | 0.53 | 0.46 | -0.33 |
| Vegetables (non-starchy) | 0.71 | 1.00 | 0.33 | 0.39 | -0.31 |
| Other non-potato starchy vegetables | 0.53 | 0.33 | 1.00 | 0.33 | -0.26 |
| Beans/legumes | 0.46 | 0.39 | 0.33 | 1.00 | -0.21 |
| Nuts and seeds | -0.33 | -0.31 | -0.26 | -0.21 | 1.00 |

Table S5: Spearman correlation coefficients in children 12-23 months of age in 2018.

|  | Fruit | Vegetables (non-starchy) | Other non-potato starchy vegetables | Beans and legumes | Nuts and seeds |
| --- | --- | --- | --- | --- | --- |
| Fruit | 1.00 | 0.54 | 0.36 | 0.26 | -0.26 |
| Vegetables (non-starchy) | 0.54 | 1.00 | 0.09 | 0.22 | -0.26 |
| Other non-potato starchy vegetables | 0.36 | 0.09 | 1.00 | 0.13 | -0.13 |
| Beans and legumes | 0.26 | 0.22 | 0.13 | 1.00 | -0.07 |
| Nuts and seeds | -0.26 | -0.26 | -0.13 | -0.07 | 1.00 |

Table S6: Spearman correlation coefficients in children 3-4 years of age in 2018.

|  | Fruit | Vegetables (non-starchy) | Other non-potato starchy vegetables | Beans and legumes | Nuts and seeds |
| --- | --- | --- | --- | --- | --- |
| Fruit | 1 | 0.36 | 0.09 | 0.15 | -0.17 |
| Vegetables (non-starchy) | 0.36 | 1 | -0.10 | 0.16 | -0.17 |
| Other non-potato starchy vegetables | 0.09 | -0.1 | 1 | 0.12 | 0.05 |
| Beans and legumes | 0.15 | 0.16 | 0.12 | 1 | 0.01 |
| Nuts and seeds | -0.17 | -0.17 | 0.05 | 0.01 | 1 |

Table S7: Spearman correlation coefficients in children 5-9 years of age in 2018.

|  | Fruit | Vegetables (non-starchy) | Other non-potato starchy vegetables | Beans and legumes | Nuts and seeds |
| --- | --- | --- | --- | --- | --- |
| Fruit | 1 | 0.37 | 0.12 | 0.24 | -0.05 |
| Vegetables (non-starchy) | 0.37 | 1 | 0.06 | 0.27 | -0.08 |
| Other non-potato starchy vegetables | 0.12 | 0.06 | 1 | 0.3 | 0.17 |
| Beans and legumes | 0.24 | 0.27 | 0.3 | 1 | 0.07 |
| Nuts and seeds | -0.05 | -0.08 | 0.17 | 0.07 | 1 |

Table S8: Spearman correlation coefficients in children 10-14 years of age in 2018.

|  | Fruit | Vegetables (non-starchy) | Other non-potato starchy vegetables | Beans and legumes | Nuts and seeds |
| --- | --- | --- | --- | --- | --- |
| Fruit | 1 | 0.41 | 0.23 | 0.31 | 0.03 |
| Vegetables (non-starchy) | 0.41 | 1 | 0.11 | 0.30 | -0.03 |
| Other non-potato starchy vegetables | 0.23 | 0.11 | 1 | 0.35 | 0.20 |
| Beans and legumes | 0.31 | 0.30 | 0.35 | 1 | 0.10 |
| Nuts and seeds | 0.03 | -0.03 | 0.20 | 0.1 | 1 |

Table S9: Spearman correlation coefficients in children 15-19 years of age in 2018.

|  | Fruit | Vegetables (non-starchy) | Other non-potato starchy vegetables | Beans and legumes | Nuts and seeds |
| --- | --- | --- | --- | --- | --- |
| Fruit | 1 | 0.39 | 0.24 | 0.30 | 0.07 |
| Vegetables (non-starchy) | 0.39 | 1 | 0.10 | 0.28 | 0 |
| Other non-potato starchy vegetables | 0.24 | 0.10 | 1 | 0.34 | 0.20 |
| Beans and legumes | 0.30 | 0.28 | 0.34 | 1 | 0.11 |
| Nuts and seeds | 0.07 | 0 | 0.20 | 0.11 | 1 |

Table S10: Global consumption (servings/day) in children less than 2 years in 2018.

| Region | Fruit | Vegetables (Non-starchy) | Other non-potato starchy vegetables | Beans and legumes | Nuts and seeds | Total-Plant-Based Food Consumption |
| --- | --- | --- | --- | --- | --- | --- |
| Globe | 0.45 (0.43, 0.47) | 0.64 (0.61, 0.68) | 0.10 (0.09, 0.11) | 0.11 (0.10, 0.13) | 0.13 (0.10, 0.17) | 1.43 (1.33, 1.56) |
| South Asia | 0.15 (0.14, 0.17) | 0.25 (0.24, 0.28) | 0.06 (0.05, 0.07) | 0.05 (0.04, 0.06) | 0.05 (0.03, 0.1) | 0.56 (0.50, 0.68) |
| Sub-Saharan Africa | 0.2 (0.19, 0.22) | 0.43 (0.41, 0.46) | 0.07 (0.06, 0.08) | 0.08 (0.08, 0.09) | 0.23 (0.14, 0.39) | 1.01 (0.88, 1.24) |
| Former Soviet Union | 0.53 (0.47, 0.61) | 0.55 (0.46, 0.64) | 0.09 (0.07, 0.14) | 0.06 (0.04, 0.09) | 0.27 (0.17, 0.42) | 1.50 (1.21, 1.90) |
| Middle East and North Africa | 0.28 (0.25, 0.32) | 0.24 (0.21, 0.26) | 0.08 (0.05, 0.12) | 0.07 (0.05, 0.11) | 0.09 (0.06, 0.15) | 0.76 (0.62, 0.96) |
| Latin America and Caribbean | 0.51 (0.47, 0.55) | 0.47 (0.44, 0.51) | 0.21 (0.19, 0.24) | 0.17 (0.15, 0.19) | 0.06 (0.03, 0.11) | 1.42 (1.28, 1.60) |
| High Income Countries | 1.74 (1.57, 1.95) | 0.9 (0.83, 0.98) | 0.24 (0.19, 0.32) | 0.17 (0.15, 0.2) | 0.06 (0.06, 0.07) | 3.11 (2.80, 3.52) |
| East and Southeast Asia | 0.7 (0.63, 0.77) | 1.46 (1.34, 1.59) | 0.09 (0.07, 0.15) | 0.2 (0.17, 0.24) | 0.13 (0.1, 0.16) | 2.58 (2.31, 2.91) |

Table S11: Global consumption (servings/day) in children 2-19 years in 2018.

| Region | Fruit | Vegetables (Non-starchy) | Other non-potato starchy vegetables | Beans and legumes | Nuts and seeds | Total Plant-Based Food Consumption |
| --- | --- | --- | --- | --- | --- | --- |
| Globe | 0.70 (0.68, 0.73) | 1.38 (1.33-1.44) | 0.20 (0.18, 0.23) | 0.26 (0.24-0.28) | 0.26 (0.22-0.31) | 2.80 (2.65, 2.99) |
| South Asia | 0.39 (0.36, 0.42) | 1.34 (1.26, 1.43) | 0.13 (0.11, 0.16) | 0.25 (0.22, 0.29) | 0.12 (0.08, 0.18) | 2.23 (2.03, 2.48) |
| Sub-Saharan Africa | 0.63 (0.59, 0.67) | 1.25 (1.19, 1.32) | 0.22 (0.19, 0.25) | 0.28 (0.25, 0.31) | 0.39 (0.25, 0.61) | 2.77 (2.47, 3.16) |
| Former Soviet Union | 0.83 (0.78, 0.9) | 0.87 (0.82, 0.94) | 0.26 (0.19, 0.39) | 0.15 (0.11, 0.21) | 0.76 (0.59, 0.95) | 2.87 (2.49, 3.49) |
| Middle East and North Africa | 0.87 (0.81, 0.93) | 1.06 (1, 1.13) | 0.23 (0.16, 0.36) | 0.27 (0.2, 0.39) | 0.29 (0.21, 0.44) | 2.72 (2.38, 3.25) |
| Latin America and Caribbean | 1.12 (1.06, 1.19) | 1.09 (1.03, 1.14) | 0.54 (0.49, 0.6) | 0.49 (0.44, 0.54) | 0.17 (0.11, 0.25) | 3.41 (3.13, 3.72) |
| High Income Countries | 0.75 (0.72, 0.78) | 0.73 (0.7, 0.75) | 0.06 (0.05, 0.08) | 0.13 (0.12, 0.14) | 0.22 (0.2, 0.23) | 1.89 (1.79, 1.98) |
| East and Southeast Asia | 0.89 (0.83, 0.95) | 2.05 (1.92, 2.18) | 0.17 (0.12, 0.26) | 0.23 (0.2, 0.26) | 0.23 (0.2, 0.28) | 3.57 (3.27, 3.93) |

Table S12: Global consumption (servings/day) in children 0 to 19 years in 2018.

| Age | Fruit | Vegetables  (non-starchy) | Other non-potato starchy vegetables | Beans and legumes | Nuts and seeds |
| --- | --- | --- | --- | --- | --- |
| <1 | 0.47 (0.44-0.51) | 0.47 (0.45-0.5) | 0.06 (0.05-0.07) | 0.07 (0.07-0.08) | 0.12 (0.09-0.18) |
| 1-2 | 0.44 (0.42-0.46) | 0.8 (0.76-0.83) | 0.13 (0.11-0.16) | 0.14 (0.13-0.16) | 0.13 (0.1-0.18) |
| 3-4 | 0.58 (0.55-0.6) | 0.93 (0.89-0.97) | 0.13 (0.11-0.15) | 0.15 (0.14-0.16) | 0.22 (0.18-0.28) |
| 5-9 | 0.66 (0.63-0.69) | 1.18 (1.13-1.22) | 0.18 (0.16-0.21) | 0.22 (0.2-0.24) | 0.25 (0.21-0.31) |
| 10-14 | 0.79 (0.76-0.82) | 1.61 (1.55-1.67) | 0.25 (0.22-0.29) | 0.32 (0.29-0.35) | 0.27 (0.23-0.34) |
| 15-19 | 0.83 (0.8-0.86) | 1.84 (1.77-1.91) | 0.25 (0.22-0.3) | 0.34 (0.32-0.37) | 0.29 (0.24-0.35) |

Table S13: Regional consumption (servings/day) in children 0-19 years in 2018.

| Region | Age | Fruit | Vegetables (non-starchy) | Other non-potato starchy Vegetables | Beans and legumes | Nuts and seeds |
| --- | --- | --- | --- | --- | --- | --- |
| East and Southeast Asia | <1 | 0.72 (0.64-0.81) | 1.16 (1.05-1.28) | 0.05 (0.04-0.09) | 0.16 (0.13-0.19) | 0.12 (0.1-0.16) |
| East and Southeast Asia | 1-2 | 0.68 (0.63-0.74) | 1.73 (1.6-1.87) | 0.13 (0.09-0.22) | 0.24 (0.2-0.28) | 0.13 (0.1-0.16) |
| East and Southeast Asia | 3-4 | 0.85 (0.79-0.91) | 1.74 (1.62-1.86) | 0.11 (0.08-0.2) | 0.19 (0.16-0.22) | 0.2 (0.17-0.24) |
| East and Southeast Asia | 5-9 | 0.87 (0.81-0.93) | 1.86 (1.74-1.98) | 0.15 (0.1-0.25) | 0.21 (0.18-0.24) | 0.22 (0.18-0.27) |
| East and Southeast Asia | 10-14 | 0.96 (0.9-1.03) | 2.27 (2.13-2.42) | 0.2 (0.14-0.34) | 0.26 (0.23-0.3) | 0.25 (0.21-0.3) |
| East and Southeast Asia | 15-19 | 0.96 (0.9-1.02) | 2.49 (2.33-2.65) | 0.21 (0.15-0.36) | 0.27 (0.23-0.31) | 0.27 (0.22-0.32) |
| Former Soviet Union | <1 | 0.53 (0.45-0.63) | 0.43 (0.35-0.53) | 0.04 (0.03-0.07) | 0.03 (0.02-0.06) | 0.22 (0.12-0.39) |
| Former Soviet Union | 1-2 | 0.53 (0.48-0.59) | 0.64 (0.56-0.74) | 0.13 (0.1-0.21) | 0.08 (0.05-0.12) | 0.31 (0.2-0.46) |
| Former Soviet Union | 3-4 | 0.69 (0.64-0.75) | 0.65 (0.59-0.72) | 0.15 (0.11-0.24) | 0.08 (0.06-0.13) | 0.59 (0.42-0.78) |
| Former Soviet Union | 5-9 | 0.75 (0.7-0.81) | 0.71 (0.66-0.78) | 0.23 (0.17-0.37) | 0.12 (0.09-0.19) | 0.76 (0.58-0.98) |
| Former Soviet Union | 10-14 | 0.93 (0.86-1) | 0.98 (0.91-1.06) | 0.33 (0.24-0.53) | 0.18 (0.13-0.27) | 0.85 (0.65-1.08) |
| Former Soviet Union | 15-19 | 1.06 (0.98-1.14) | 1.26 (1.17-1.37) | 0.35 (0.25-0.54) | 0.21 (0.15-0.32) | 0.82 (0.64-1.04) |
| High Income Countries | <1 | 2.43 (2.14-2.77) | 0.87 (0.78-0.97) | 0.26 (0.21-0.36) | 0.16 (0.13-0.19) | 0.05 (0.04-0.06) |
| High Income Countries | 1-2 | 1.3 (1.19-1.41) | 0.94 (0.87-1) | 0.22 (0.17-0.3) | 0.18 (0.16-0.21) | 0.07 (0.06-0.08) |
| High Income Countries | 3-4 | 1.04 (0.98-1.1) | 0.74 (0.7-0.77) | 0.09 (0.07-0.12) | 0.12 (0.11-0.13) | 0.14 (0.13-0.15) |
| High Income Countries | 5-9 | 0.72 (0.69-0.76) | 0.63 (0.61-0.66) | 0.06 (0.05-0.08) | 0.11 (0.1-0.12) | 0.19 (0.17-0.2) |
| High Income Countries | 10-14 | 0.71 (0.68-0.74) | 0.75 (0.72-0.78) | 0.06 (0.05-0.08) | 0.13 (0.12-0.15) | 0.24 (0.22-0.26) |
| High Income Countries | 15-19 | 0.76 (0.73-0.79) | 0.89 (0.87-0.93) | 0.06 (0.05-0.08) | 0.16 (0.15-0.17) | 0.27 (0.25-0.29) |
| Latin America and Caribbean | <1 | 0.44 (0.4-0.49) | 0.31 (0.28-0.34) | 0.1 (0.09-0.12) | 0.09 (0.08-0.11) | 0.05 (0.03-0.1) |
| Latin America and Caribbean | 1-2 | 0.55 (0.51-0.59) | 0.61 (0.57-0.65) | 0.3 (0.27-0.34) | 0.23 (0.2-0.26) | 0.06 (0.04-0.12) |
| Latin America and Caribbean | 3-4 | 0.84 (0.8-0.9) | 0.75 (0.71-0.79) | 0.31 (0.28-0.36) | 0.26 (0.24-0.3) | 0.11 (0.06-0.2) |
| Latin America and Caribbean | 5-9 | 1.04 (0.98-1.11) | 0.95 (0.9-1) | 0.48 (0.43-0.54) | 0.4 (0.36-0.45) | 0.14 (0.08-0.23) |
| Latin America and Caribbean | 10-14 | 1.27 (1.2-1.36) | 1.25 (1.18-1.32) | 0.66 (0.6-0.74) | 0.59 (0.53-0.67) | 0.18 (0.11-0.29) |
| Latin America and Caribbean | 15-19 | 1.31 (1.24-1.39) | 1.36 (1.3-1.43) | 0.66 (0.6-0.74) | 0.65 (0.58-0.73) | 0.21 (0.12-0.33) |
| Middle East and North Africa | <1 | 0.22 (0.19-0.26) | 0.13 (0.11-0.14) | 0.04 (0.03-0.06) | 0.04 (0.03-0.06) | 0.08 (0.05-0.14) |
| Middle East and North Africa | 1-2 | 0.32 (0.29-0.36) | 0.34 (0.31-0.37) | 0.11 (0.08-0.19) | 0.1 (0.07-0.15) | 0.1 (0.07-0.17) |
| Middle East and North Africa | 3-4 | 0.56 (0.51-0.61) | 0.54 (0.5-0.58) | 0.13 (0.09-0.2) | 0.13 (0.09-0.19) | 0.19 (0.13-0.31) |
| Middle East and North Africa | 5-9 | 0.77 (0.71-0.83) | 0.86 (0.8-0.92) | 0.21 (0.14-0.33) | 0.22 (0.16-0.33) | 0.26 (0.18-0.41) |
| Middle East and North Africa | 10-14 | 1.03 (0.95-1.12) | 1.31 (1.22-1.4) | 0.3 (0.2-0.48) | 0.34 (0.25-0.51) | 0.33 (0.23-0.51) |
| Middle East and North Africa | 15-19 | 1.15 (1.07-1.24) | 1.58 (1.48-1.68) | 0.3 (0.21-0.48) | 0.38 (0.28-0.55) | 0.38 (0.27-0.58) |
| South Asia | <1 | 0.13 (0.11-0.14) | 0.13 (0.12-0.14) | 0.03 (0.02-0.04) | 0.02 (0.02-0.03) | 0.05 (0.03-0.1) |
| South Asia | 1-2 | 0.17 (0.16-0.18) | 0.37 (0.34-0.39) | 0.08 (0.07-0.11) | 0.07 (0.06-0.09) | 0.05 (0.03-0.1) |
| South Asia | 3-4 | 0.28 (0.25-0.3) | 0.61 (0.57-0.65) | 0.08 (0.07-0.11) | 0.1 (0.09-0.13) | 0.09 (0.06-0.17) |
| South Asia | 5-9 | 0.36 (0.33-0.39) | 1.03 (0.96-1.1) | 0.12 (0.1-0.15) | 0.19 (0.16-0.24) | 0.11 (0.07-0.18) |
| South Asia | 10-14 | 0.45 (0.41-0.49) | 1.6 (1.5-1.72) | 0.16 (0.13-0.2) | 0.3 (0.26-0.37) | 0.13 (0.09-0.2) |
| South Asia | 15-19 | 0.47 (0.44-0.51) | 1.91 (1.8-2.04) | 0.15 (0.13-0.19) | 0.35 (0.31-0.41) | 0.14 (0.09-0.21) |
| Sub-Saharan Africa | <1 | 0.16 (0.15-0.17) | 0.26 (0.25-0.28) | 0.03 (0.03-0.04) | 0.04 (0.04-0.05) | 0.23 (0.13-0.42) |
| Sub-Saharan Africa | 1-2 | 0.23 (0.22-0.25) | 0.58 (0.55-0.62) | 0.1 (0.09-0.12) | 0.12 (0.11-0.13) | 0.23 (0.14-0.38) |
| Sub-Saharan Africa | 3-4 | 0.41 (0.39-0.44) | 0.79 (0.75-0.84) | 0.12 (0.11-0.14) | 0.15 (0.13-0.16) | 0.35 (0.22-0.58) |
| Sub-Saharan Africa | 5-9 | 0.58 (0.54-0.61) | 1.11 (1.05-1.17) | 0.19 (0.17-0.23) | 0.25 (0.22-0.28) | 0.38 (0.24-0.61) |
| Sub-Saharan Africa | 10-14 | 0.76 (0.71-0.81) | 1.53 (1.45-1.62) | 0.28 (0.25-0.33) | 0.36 (0.33-0.41) | 0.41 (0.26-0.67) |
| Sub-Saharan Africa | 15-19 | 0.83 (0.78-0.88) | 1.71 (1.62-1.8) | 0.29 (0.25-0.33) | 0.38 (0.34-0.42) | 0.43 (0.27-0.68) |

Table S14: National consumption (servings/day) in children <2 years in 2018.

| Country | Fruit | Vegetables (non-Starchy) | Other non-potato starchy vegetables | Beans and Legumes | Nuts and Seeds | Total Plant-Based Food Consumption |
| --- | --- | --- | --- | --- | --- | --- |
| Afghanistan | 0.27 (0.19, 0.38) | 0.17 (0.11, 0.26) | 0.16 (0.06, 0.48) | 0.2 (0.07, 0.6) | 0.44 (0.14, 1.28) | 1.23 (0.57, 2.99) |
| Angola | 0.38 (0.31, 0.48) | 1.06 (0.83, 1.39) | 0.1 (0.05, 0.21) | 0.12 (0.07, 0.25) | 0.22 (0.1, 0.52) | 1.88 (1.36, 2.86) |
| Albania | 0.7 (0.59, 0.84) | 0.39 (0.32, 0.48) | 0.17 (0.13, 0.22) | 0.14 (0.11, 0.18) | 0.17 (0.08, 0.38) | 1.56 (1.22, 2.1) |
| United Arab Emirates | 0.29 (0.24, 0.35) | 0.19 (0.15, 0.22) | 0.14 (0.06, 0.38) | 0.1 (0.04, 0.27) | 0.28 (0.12, 0.66) | 1 (0.62, 1.87) |
| Argentina | 0.43 (0.37, 0.49) | 0.47 (0.41, 0.54) | 0.02 (0.01, 0.03) | 0.01 (0.01, 0.02) | 0.01 (0.01, 0.02) | 0.94 (0.81, 1.1) |
| Armenia | 0.46 (0.38, 0.56) | 0.29 (0.23, 0.37) | 0.22 (0.18, 0.28) | 0.05 (0.04, 0.07) | 0.26 (0.17, 0.42) | 1.29 (0.99, 1.71) |
| Antigua & Barbuda | 0.43 (0.3, 0.63) | 0.88 (0.63, 1.23) | 0.14 (0.07, 0.33) | 0.16 (0.08, 0.35) | 0.02 (0.01, 0.05) | 1.63 (1.08, 2.6) |
| Australia | 2.1 (1.7, 2.58) | 0.8 (0.67, 0.96) | 0.22 (0.09, 0.63) | 0.21 (0.15, 0.31) | 0.06 (0.04, 0.07) | 3.39 (2.65, 4.54) |
| Austria | 1.61 (1.38, 1.91) | 0.77 (0.68, 0.88) | 0.41 (0.19, 1) | 0.06 (0.04, 0.09) | 0.04 (0.03, 0.05) | 2.89 (2.32, 3.94) |
| Azerbaijan | 0.53 (0.44, 0.65) | 0.24 (0.19, 0.3) | 0.1 (0.08, 0.12) | 0.05 (0.04, 0.07) | 0.46 (0.21, 0.99) | 1.4 (0.97, 2.13) |
| Burundi | 0.23 (0.19, 0.29) | 0.6 (0.51, 0.71) | 0.07 (0.06, 0.09) | 0.13 (0.11, 0.16) | 0.12 (0.05, 0.33) | 1.17 (0.92, 1.59) |
| Belgium | 1.61 (1.38, 1.88) | 0.76 (0.66, 0.87) | 0.31 (0.13, 0.86) | 0.04 (0.03, 0.06) | 0.03 (0.02, 0.03) | 2.75 (2.21, 3.7) |
| Benin | 0.23 (0.21, 0.26) | 0.31 (0.28, 0.35) | 0.04 (0.04, 0.05) | 0.08 (0.07, 0.09) | 0.25 (0.11, 0.58) | 0.92 (0.71, 1.33) |
| Burkina Faso | 0.11 (0.1, 0.13) | 0.09 (0.08, 0.1) | 0.01 (0.01, 0.01) | 0.03 (0.02, 0.04) | 0.43 (0.14, 1.26) | 0.67 (0.35, 1.53) |
| Bangladesh | 0.22 (0.2, 0.24) | 0.36 (0.33, 0.39) | 0.04 (0.04, 0.05) | 0.05 (0.05, 0.06) | 0.01 (0.01, 0.01) | 0.68 (0.62, 0.75) |
| Bulgaria | 0.47 (0.38, 0.58) | 1.14 (0.95, 1.38) | 0.01 (0, 0.02) | 0.06 (0.04, 0.08) | 0.18 (0.13, 0.26) | 1.87 (1.51, 2.32) |
| Bahrain | 0.32 (0.24, 0.43) | 0.28 (0.2, 0.39) | 0.08 (0.03, 0.2) | 0.1 (0.04, 0.25) | 0.18 (0.08, 0.44) | 0.96 (0.59, 1.71) |
| Bahamas | 0.38 (0.25, 0.55) | 0.4 (0.28, 0.56) | 0.14 (0.06, 0.36) | 0.06 (0.03, 0.15) | 0.08 (0.04, 0.2) | 1.06 (0.67, 1.83) |
| Bosnia & Herzegovina | 2.99 (2.41, 3.69) | 1.41 (1.14, 1.74) | 0.09 (0.04, 0.2) | 0.05 (0.02, 0.1) | 0.13 (0.06, 0.28) | 4.66 (3.67, 6) |
| Belarus | 1.22 (0.66, 2.22) | 0.25 (0.14, 0.46) | 0.1 (0.04, 0.24) | 0.25 (0.12, 0.62) | 0.16 (0.07, 0.38) | 1.98 (1.03, 3.92) |
| Belize | 0.39 (0.27, 0.57) | 0.58 (0.41, 0.81) | 0.12 (0.05, 0.31) | 0.1 (0.05, 0.23) | 0.02 (0.01, 0.04) | 1.21 (0.79, 1.96) |
| Bolivia | 0.4 (0.35, 0.44) | 0.44 (0.4, 0.49) | 0.2 (0.18, 0.23) | 0.04 (0.04, 0.05) | 0.1 (0.05, 0.23) | 1.19 (1.01, 1.45) |
| Brazil | 0.38 (0.34, 0.43) | 0.35 (0.32, 0.39) | 0.02 (0.02, 0.03) | 0.31 (0.26, 0.36) | 0 (0, 0) | 1.07 (0.94, 1.23) |
| Barbados | 0.57 (0.47, 0.7) | 0.53 (0.44, 0.63) | 0.12 (0.06, 0.3) | 0.03 (0.02, 0.04) | 0.08 (0.05, 0.12) | 1.32 (1.03, 1.79) |
| Brunei | 0.69 (0.49, 0.98) | 0.99 (0.75, 1.32) | 0.06 (0.03, 0.14) | 0.29 (0.14, 0.62) | 0.06 (0.03, 0.14) | 2.09 (1.44, 3.21) |
| Bhutan | 0.38 (0.28, 0.51) | 0.47 (0.33, 0.66) | 0.07 (0.03, 0.2) | 0.12 (0.05, 0.35) | 0.08 (0.03, 0.24) | 1.13 (0.73, 1.97) |
| Botswana | 0.15 (0.12, 0.2) | 0.34 (0.28, 0.42) | 0.06 (0.03, 0.13) | 0.06 (0.03, 0.11) | 0.83 (0.4, 1.57) | 1.45 (0.86, 2.43) |
| Central African Republic | 0.31 (0.24, 0.39) | 0.33 (0.25, 0.43) | 0.18 (0.15, 0.22) | 0.11 (0.06, 0.24) | 0.15 (0.07, 0.35) | 1.08 (0.78, 1.63) |
| Canada | 2.12 (1.84, 2.44) | 0.87 (0.79, 0.98) | 0.23 (0.19, 0.28) | 0.17 (0.14, 0.21) | 0.13 (0.11, 0.15) | 3.52 (3.07, 4.06) |
| Switzerland | 2.12 (1.77, 2.54) | 0.75 (0.65, 0.89) | 0.57 (0.26, 1.42) | 0.02 (0.02, 0.03) | 0.05 (0.03, 0.1) | 3.52 (2.71, 4.98) |
| Chile | 0.49 (0.4, 0.6) | 0.52 (0.44, 0.63) | 0.25 (0.11, 0.63) | 0.05 (0.03, 0.08) | 0.15 (0.07, 0.37) | 1.46 (1.04, 2.32) |
| China | 0.73 (0.65, 0.82) | 1.82 (1.64, 2.02) | 0.07 (0.03, 0.16) | 0.13 (0.1, 0.16) | 0.14 (0.11, 0.17) | 2.88 (2.53, 3.33) |
| Côte d’lvoire | 0.19 (0.16, 0.22) | 0.29 (0.25, 0.34) | 0.06 (0.05, 0.07) | 0.01 (0.01, 0.02) | 0.18 (0.08, 0.43) | 0.73 (0.55, 1.08) |
| Cameroon | 0.26 (0.23, 0.29) | 0.4 (0.36, 0.44) | 0.07 (0.06, 0.09) | 0.1 (0.08, 0.11) | 0.29 (0.14, 0.66) | 1.12 (0.87, 1.6) |
| Congo - Kinshasa | 0.3 (0.27, 0.34) | 0.69 (0.63, 0.77) | 0.09 (0.08, 0.1) | 0.1 (0.09, 0.12) | 0.51 (0.21, 1.25) | 1.69 (1.27, 2.58) |
| Congo - Brazzaville | 0.28 (0.25, 0.32) | 0.45 (0.41, 0.49) | 0.08 (0.06, 0.09) | 0.07 (0.06, 0.09) | 0.19 (0.09, 0.43) | 1.07 (0.86, 1.43) |
| Colombia | 0.45 (0.41, 0.5) | 0.37 (0.33, 0.4) | 0.27 (0.24, 0.3) | 0.1 (0.09, 0.11) | 0.46 (0.22, 0.96) | 1.65 (1.3, 2.26) |
| Comoros | 0.26 (0.23, 0.31) | 0.3 (0.27, 0.35) | 0.08 (0.07, 0.09) | 0.03 (0.02, 0.04) | 0.15 (0.07, 0.34) | 0.83 (0.65, 1.14) |
| Cape Verde | 0.71 (0.51, 0.97) | 0.58 (0.44, 0.76) | 0.05 (0.02, 0.11) | 0.09 (0.05, 0.19) | 0.28 (0.13, 0.66) | 1.7 (1.15, 2.7) |
| Costa Rica | 0.59 (0.41, 0.86) | 0.63 (0.45, 0.86) | 0.14 (0.06, 0.33) | 0.1 (0.05, 0.22) | 0.07 (0.03, 0.16) | 1.53 (1, 2.44) |
| Cuba | 0.58 (0.46, 0.74) | 0.42 (0.33, 0.55) | 0.17 (0.08, 0.39) | 0.09 (0.05, 0.21) | 0 (0, 0.01) | 1.27 (0.91, 1.89) |
| Cyprus | 0.95 (0.59, 1.57) | 0.8 (0.49, 1.34) | 0.19 (0.09, 0.47) | 0.28 (0.11, 0.73) | 0.03 (0.01, 0.08) | 2.26 (1.29, 4.19) |
| Czechia | 0.37 (0.31, 0.44) | 0.3 (0.25, 0.37) | 0.09 (0.04, 0.23) | 0.04 (0.02, 0.09) | 0.05 (0.03, 0.08) | 0.85 (0.65, 1.21) |
| Germany | 2.13 (1.86, 2.44) | 1.25 (1.12, 1.4) | 0.43 (0.35, 0.52) | 0.05 (0.04, 0.06) | 0.02 (0.02, 0.03) | 3.88 (3.39, 4.44) |
| Djibouti | 0.44 (0.34, 0.57) | 0.31 (0.24, 0.43) | 0.04 (0.02, 0.11) | 0.68 (0.36, 1.52) | 0 (0, 0) | 1.47 (0.96, 2.64) |
| Dominica | 0.48 (0.38, 0.61) | 0.39 (0.32, 0.49) | 0.16 (0.07, 0.38) | 0.11 (0.06, 0.25) | 0.04 (0.02, 0.09) | 1.18 (0.85, 1.81) |
| Denmark | 1.4 (1.14, 1.7) | 0.81 (0.69, 0.96) | 0.36 (0.16, 1.01) | 0.2 (0.1, 0.44) | 0.06 (0.04, 0.09) | 2.83 (2.13, 4.2) |
| Dominican Republic | 0.65 (0.59, 0.72) | 0.4 (0.36, 0.45) | 0.16 (0.14, 0.18) | 0.17 (0.15, 0.19) | 0.01 (0.01, 0.03) | 1.4 (1.25, 1.57) |
| Algeria | 0.22 (0.17, 0.28) | 0.31 (0.25, 0.39) | 0.1 (0.04, 0.27) | 0.05 (0.02, 0.17) | 0.05 (0.02, 0.12) | 0.74 (0.51, 1.22) |
| Ecuador | 0.12 (0.1, 0.14) | 0.42 (0.37, 0.48) | 0.08 (0.04, 0.17) | 0.06 (0.03, 0.1) | 0.02 (0.01, 0.05) | 0.7 (0.55, 0.95) |
| Egypt | 0.25 (0.23, 0.27) | 0.24 (0.22, 0.27) | 0.07 (0.06, 0.08) | 0.02 (0.02, 0.03) | 0.02 (0.01, 0.03) | 0.61 (0.55, 0.67) |
| Eritrea | 0.18 (0.13, 0.24) | 0.22 (0.17, 0.28) | 0.05 (0.03, 0.11) | 0.08 (0.05, 0.17) | 0.19 (0.09, 0.44) | 0.72 (0.46, 1.24) |
| Spain | 0.98 (0.84, 1.14) | 0.67 (0.59, 0.75) | 0.18 (0.08, 0.45) | 0.19 (0.09, 0.41) | 0.06 (0.05, 0.08) | 2.08 (1.64, 2.84) |
| Estonia | 0.52 (0.44, 0.62) | 0.85 (0.71, 1.02) | 0.01 (0.01, 0.01) | 0.02 (0.01, 0.03) | 0.12 (0.08, 0.2) | 1.52 (1.25, 1.87) |
| Ethiopia | 0.12 (0.11, 0.13) | 0.28 (0.25, 0.31) | 0.04 (0.04, 0.05) | 0.11 (0.1, 0.12) | 0.03 (0.02, 0.05) | 0.58 (0.51, 0.66) |
| Finland | 2.31 (1.98, 2.68) | 0.83 (0.73, 0.94) | 0.12 (0.05, 0.29) | 0.06 (0.04, 0.07) | 0.02 (0.02, 0.03) | 3.34 (2.82, 4) |
| Fiji | 0.91 (0.61, 1.33) | 1.32 (0.96, 1.81) | 0.09 (0.04, 0.21) | 0.25 (0.13, 0.51) | 0.07 (0.03, 0.14) | 2.63 (1.77, 4) |
| France | 2.35 (2.04, 2.71) | 1.05 (0.94, 1.17) | 0.02 (0.01, 0.02) | 0.12 (0.1, 0.15) | 0.02 (0.02, 0.02) | 3.56 (3.1, 4.08) |
| Micronesia (Federated States of) | 1.2 (0.86, 1.68) | 0.87 (0.59, 1.31) | 0.09 (0.04, 0.21) | 0.24 (0.12, 0.53) | 0.04 (0.02, 0.1) | 2.43 (1.63, 3.82) |
| Gabon | 0.22 (0.19, 0.25) | 0.38 (0.34, 0.43) | 0.1 (0.08, 0.11) | 0.01 (0.01, 0.01) | 0.39 (0.17, 0.88) | 1.09 (0.78, 1.69) |
| United Kingdom | 1.43 (1.23, 1.65) | 0.79 (0.7, 0.89) | 0.26 (0.11, 0.68) | 0.37 (0.29, 0.47) | 0.04 (0.03, 0.05) | 2.89 (2.37, 3.74) |
| Georgia | 0.36 (0.3, 0.44) | 0.26 (0.21, 0.33) | 0.06 (0.03, 0.14) | 0.12 (0.06, 0.26) | 0.09 (0.04, 0.2) | 0.89 (0.63, 1.37) |
| Ghana | 0.27 (0.25, 0.3) | 0.43 (0.4, 0.46) | 0.06 (0.05, 0.07) | 0.06 (0.05, 0.07) | 0.56 (0.3, 1.05) | 1.38 (1.05, 1.94) |
| Guinea | 0.07 (0.06, 0.08) | 0.06 (0.05, 0.08) | 0.03 (0.02, 0.04) | 0.01 (0.01, 0.01) | 0.2 (0.09, 0.48) | 0.37 (0.23, 0.69) |
| Gambia | 0.1 (0.08, 0.12) | 0.15 (0.12, 0.19) | 0.03 (0.02, 0.03) | 0.02 (0.02, 0.03) | 0.22 (0.1, 0.57) | 0.52 (0.34, 0.94) |
| Guinea-Bissau | 0.21 (0.17, 0.27) | 0.44 (0.34, 0.57) | 0.06 (0.03, 0.13) | 0.29 (0.15, 0.61) | 0.24 (0.11, 0.55) | 1.23 (0.81, 2.13) |
| Equatorial Guinea | 0.28 (0.22, 0.36) | 0.46 (0.35, 0.62) | 0.08 (0.04, 0.19) | 0.12 (0.06, 0.24) | 0.21 (0.1, 0.49) | 1.15 (0.78, 1.89) |
| Greece | 1.17 (0.98, 1.39) | 0.59 (0.51, 0.69) | 0.21 (0.09, 0.53) | 0.12 (0.09, 0.17) | 0.05 (0.04, 0.07) | 2.14 (1.71, 2.85) |
| Grenada | 0.55 (0.38, 0.79) | 0.96 (0.68, 1.34) | 0.16 (0.08, 0.37) | 0.1 (0.05, 0.23) | 0 (0, 0) | 1.78 (1.19, 2.74) |
| Guatemala | 0.35 (0.31, 0.39) | 0.37 (0.33, 0.4) | 0.1 (0.09, 0.12) | 0.17 (0.15, 0.2) | 0.02 (0.01, 0.05) | 1.02 (0.89, 1.17) |
| Guyana | 0.92 (0.78, 1.09) | 0.56 (0.49, 0.65) | 0.13 (0.11, 0.16) | 0.08 (0.06, 0.1) | 0.07 (0.04, 0.16) | 1.77 (1.48, 2.16) |
| Honduras | 0.41 (0.36, 0.46) | 0.12 (0.1, 0.13) | 0.1 (0.08, 0.11) | 0.15 (0.13, 0.18) | 0.04 (0.03, 0.06) | 0.82 (0.71, 0.95) |
| Croatia | 0.29 (0.24, 0.36) | 2.13 (1.77, 2.59) | 0.1 (0.05, 0.23) | 0.03 (0.01, 0.07) | 0.14 (0.05, 0.33) | 2.69 (2.12, 3.59) |
| Haiti | 0.32 (0.28, 0.36) | 0.32 (0.28, 0.36) | 0.07 (0.06, 0.08) | 0.1 (0.09, 0.12) | 0.05 (0.03, 0.07) | 0.86 (0.74, 0.98) |
| Hungary | 0.3 (0.25, 0.35) | 0.18 (0.15, 0.21) | 0.08 (0.04, 0.19) | 0.06 (0.02, 0.17) | 0.03 (0.01, 0.04) | 0.63 (0.46, 0.96) |
| Indonesia | 0.56 (0.51, 0.61) | 0.99 (0.91, 1.07) | 0.14 (0.12, 0.16) | 0.24 (0.21, 0.27) | 0.08 (0.06, 0.1) | 2 (1.81, 2.21) |
| India | 0.14 (0.13, 0.16) | 0.27 (0.25, 0.29) | 0.05 (0.04, 0.06) | 0.05 (0.04, 0.05) | 0.04 (0.03, 0.05) | 0.54 (0.49, 0.61) |
| Ireland | 1.26 (1.02, 1.57) | 0.81 (0.67, 0.95) | 0.31 (0.14, 0.75) | 0.21 (0.09, 0.46) | 0.1 (0.05, 0.2) | 2.69 (1.96, 3.93) |
| Iran | 0.45 (0.39, 0.51) | 0.35 (0.3, 0.39) | 0.02 (0.02, 0.03) | 0.04 (0.03, 0.05) | 0.06 (0.05, 0.08) | 0.93 (0.8, 1.07) |
| Iraq | 0.29 (0.2, 0.43) | 0.2 (0.14, 0.27) | 0.08 (0.04, 0.22) | 0.1 (0.04, 0.24) | 0.12 (0.05, 0.29) | 0.8 (0.48, 1.45) |
| Iceland | 1.22 (1.03, 1.44) | 0.48 (0.42, 0.55) | 1.66 (0.69, 3.99) | 0.05 (0.04, 0.07) | 0.01 (0.01, 0.01) | 3.42 (2.19, 6.06) |
| Israel | 0.36 (0.31, 0.42) | 0.13 (0.11, 0.15) | 0.13 (0.1, 0.17) | 0.19 (0.15, 0.25) | 0.26 (0.21, 0.34) | 1.08 (0.88, 1.32) |
| Italy | 3 (2.62, 3.45) | 0.96 (0.85, 1.08) | 0.27 (0.21, 0.34) | 0.12 (0.1, 0.15) | 0.02 (0.02, 0.03) | 4.38 (3.81, 5.05) |
| Jamaica | 0.8 (0.62, 1.03) | 0.57 (0.46, 0.7) | 0.11 (0.05, 0.24) | 0.04 (0.03, 0.06) | 0.03 (0.02, 0.04) | 1.55 (1.18, 2.08) |
| Jordan | 0.48 (0.43, 0.53) | 0.19 (0.17, 0.21) | 0.05 (0.05, 0.06) | 0.07 (0.06, 0.08) | 0.1 (0.07, 0.14) | 0.89 (0.78, 1.02) |
| Japan | 0.83 (0.77, 0.9) | 1.63 (1.53, 1.74) | 0.03 (0.02, 0.05) | 0.46 (0.38, 0.56) | 0.03 (0.03, 0.04) | 2.99 (2.73, 3.29) |
| Kazakhstan | 0.31 (0.26, 0.37) | 0.51 (0.43, 0.62) | 0.13 (0.1, 0.16) | 0.02 (0.01, 0.05) | 1.54 (0.8, 2.45) | 2.51 (1.61, 3.64) |
| Kenya | 0.32 (0.29, 0.35) | 0.77 (0.71, 0.83) | 0.11 (0.1, 0.13) | 0.09 (0.08, 0.1) | 0.01 (0.01, 0.02) | 1.3 (1.18, 1.43) |
| Kyrgyzstan | 0.4 (0.34, 0.47) | 0.13 (0.1, 0.18) | 0.12 (0.11, 0.15) | 0.02 (0.02, 0.03) | 0.51 (0.24, 1.1) | 1.2 (0.81, 1.93) |
| Cambodia | 0.41 (0.36, 0.46) | 0.74 (0.68, 0.82) | 0.09 (0.08, 0.1) | 0.03 (0.03, 0.04) | 0.02 (0.02, 0.04) | 1.29 (1.16, 1.45) |
| Kiribati | 0.81 (0.54, 1.23) | 0.56 (0.39, 0.81) | 0.09 (0.04, 0.21) | 0.25 (0.13, 0.54) | 0.05 (0.03, 0.11) | 1.78 (1.13, 2.9) |
| South Korea | 0.77 (0.7, 0.84) | 0.8 (0.75, 0.87) | 0.02 (0.02, 0.03) | 0.23 (0.2, 0.26) | 0.02 (0.02, 0.03) | 1.85 (1.68, 2.03) |
| Kuwait | 0.15 (0.13, 0.19) | 0.17 (0.14, 0.2) | 0.05 (0.02, 0.14) | 0.12 (0.05, 0.32) | 0.22 (0.09, 0.53) | 0.7 (0.43, 1.38) |
| Laos | 0.99 (0.86, 1.14) | 0.89 (0.79, 0.99) | 0.04 (0.02, 0.05) | 0.22 (0.11, 0.48) | 0.01 (0.01, 0.01) | 2.14 (1.8, 2.67) |
| Lebanon | 0.6 (0.51, 0.72) | 0.54 (0.46, 0.63) | 0.04 (0.03, 0.06) | 0.07 (0.06, 0.09) | 0.4 (0.3, 0.53) | 1.66 (1.36, 2.02) |
| Liberia | 0.15 (0.13, 0.18) | 0.38 (0.33, 0.44) | 0.06 (0.05, 0.07) | 0.03 (0.02, 0.04) | 0.05 (0.02, 0.11) | 0.68 (0.56, 0.85) |
| Libya | 0.14 (0.11, 0.19) | 0.18 (0.14, 0.22) | 0.08 (0.03, 0.19) | 0.1 (0.04, 0.24) | 0.18 (0.08, 0.4) | 0.67 (0.41, 1.23) |
| St. Lucia | 0.34 (0.24, 0.5) | 0.34 (0.24, 0.47) | 0.2 (0.09, 0.47) | 0.2 (0.1, 0.46) | 0 (0, 0.01) | 1.09 (0.67, 1.92) |
| Sri Lanka | 0.31 (0.27, 0.36) | 0.31 (0.28, 0.35) | 0.15 (0.06, 0.41) | 0.15 (0.12, 0.18) | 0.11 (0.04, 0.35) | 1.05 (0.78, 1.66) |
| Lesotho | 0.21 (0.18, 0.24) | 0.58 (0.51, 0.65) | 0.04 (0.04, 0.05) | 0.05 (0.04, 0.06) | 0.03 (0.01, 0.08) | 0.91 (0.79, 1.07) |
| Lithuania | 0.34 (0.28, 0.42) | 0.38 (0.31, 0.46) | 0.1 (0.05, 0.23) | 0.09 (0.04, 0.19) | 0.14 (0.08, 0.25) | 1.05 (0.76, 1.55) |
| Luxembourg | 1.35 (1.02, 1.79) | 0.64 (0.5, 0.81) | 0.39 (0.17, 1.04) | 0.09 (0.03, 0.3) | 0 (0, 0) | 2.47 (1.71, 3.95) |
| Latvia | 0.29 (0.25, 0.35) | 0.67 (0.56, 0.81) | 0.11 (0.05, 0.29) | 0.03 (0.02, 0.08) | 0.11 (0.06, 0.18) | 1.22 (0.93, 1.7) |
| Morocco | 0.26 (0.21, 0.32) | 0.26 (0.21, 0.31) | 0.09 (0.04, 0.23) | 0.1 (0.04, 0.26) | 0.14 (0.06, 0.32) | 0.84 (0.56, 1.44) |
| Moldova | 0.49 (0.28, 0.86) | 0.26 (0.14, 0.48) | 0.1 (0.05, 0.25) | 0.39 (0.19, 0.87) | 0.23 (0.11, 0.52) | 1.47 (0.77, 2.98) |
| Madagascar | 0.28 (0.25, 0.31) | 0.52 (0.47, 0.58) | 0.07 (0.06, 0.08) | 0.03 (0.02, 0.03) | 0.02 (0.01, 0.04) | 0.91 (0.81, 1.03) |
| Maldives | 0.9 (0.76, 1.07) | 0.27 (0.23, 0.32) | 0.14 (0.12, 0.18) | 0.05 (0.04, 0.06) | 0.18 (0.06, 0.54) | 1.54 (1.21, 2.16) |
| Mexico | 0.68 (0.61, 0.75) | 0.74 (0.68, 0.81) | 0.56 (0.51, 0.62) | 0.15 (0.13, 0.17) | 0.02 (0.01, 0.02) | 2.16 (1.95, 2.38) |
| Marshall Islands | 0.92 (0.72, 1.18) | 1.16 (0.88, 1.57) | 0.08 (0.03, 0.18) | 0.34 (0.17, 0.75) | 0.05 (0.03, 0.12) | 2.54 (1.83, 3.8) |
| North Macedonia | 0.46 (0.36, 0.58) | 0.44 (0.35, 0.55) | 0.07 (0.03, 0.16) | 0.07 (0.04, 0.15) | 0.17 (0.08, 0.39) | 1.21 (0.86, 1.84) |
| Mali | 0.22 (0.2, 0.25) | 0.3 (0.27, 0.33) | 0.06 (0.05, 0.07) | 0.05 (0.04, 0.06) | 0.25 (0.08, 0.77) | 0.89 (0.64, 1.49) |
| Malta | 2.04 (1.68, 2.47) | 0.59 (0.5, 0.7) | 0.35 (0.15, 0.94) | 0.03 (0.02, 0.06) | 0.05 (0.02, 0.1) | 3.07 (2.38, 4.27) |
| Myanmar (Burma) | 0.65 (0.56, 0.77) | 1.07 (0.94, 1.22) | 0.1 (0.05, 0.27) | 0.21 (0.1, 0.5) | 0.07 (0.03, 0.15) | 2.11 (1.68, 2.9) |
| Montenegro | 1.23 (0.73, 2.09) | 0.4 (0.23, 0.72) | 0.09 (0.04, 0.2) | 0.11 (0.04, 0.28) | 0.16 (0.06, 0.42) | 1.99 (1.1, 3.71) |
| Mongolia | 0.26 (0.21, 0.32) | 0.58 (0.47, 0.72) | 0.04 (0.02, 0.1) | 0.1 (0.05, 0.23) | 0.03 (0.01, 0.06) | 1 (0.76, 1.43) |
| Mozambique | 0.32 (0.28, 0.37) | 0.44 (0.4, 0.5) | 0.09 (0.07, 0.11) | 0.1 (0.08, 0.12) | 0.21 (0.13, 0.33) | 1.16 (0.96, 1.43) |
| Mauritania | 0.11 (0.09, 0.13) | 0.7 (0.61, 0.81) | 0.04 (0.02, 0.08) | 0.06 (0.03, 0.13) | 0.03 (0.02, 0.08) | 0.94 (0.76, 1.23) |
| Mauritius | 0.22 (0.19, 0.26) | 0.52 (0.46, 0.58) | 0.06 (0.03, 0.13) | 0.11 (0.07, 0.16) | 0.16 (0.08, 0.38) | 1.08 (0.84, 1.51) |
| Malawi | 0.35 (0.32, 0.39) | 0.55 (0.51, 0.59) | 0.07 (0.06, 0.08) | 0.08 (0.07, 0.09) | 0.15 (0.07, 0.37) | 1.2 (1.02, 1.52) |
| Malaysia | 0.88 (0.78, 1.01) | 1 (0.9, 1.11) | 0.06 (0.04, 0.07) | 0.22 (0.18, 0.27) | 0.88 (0.65, 1.17) | 3.03 (2.55, 3.62) |
| Namibia | 0.79 (0.71, 0.87) | 0.47 (0.43, 0.51) | 0.06 (0.05, 0.07) | 0.04 (0.03, 0.05) | 0.16 (0.07, 0.35) | 1.51 (1.29, 1.85) |
| Niger | 0.21 (0.18, 0.24) | 0.33 (0.29, 0.37) | 0.39 (0.33, 0.47) | 0.1 (0.08, 0.11) | 0.31 (0.13, 0.81) | 1.34 (1.01, 2) |
| Nigeria | 0.11 (0.1, 0.13) | 0.3 (0.27, 0.33) | 0.05 (0.04, 0.05) | 0.06 (0.06, 0.07) | 0.21 (0.1, 0.49) | 0.73 (0.56, 1.06) |
| Nicaragua | 0.72 (0.57, 0.9) | 1.08 (0.85, 1.41) | 0.23 (0.11, 0.53) | 0.1 (0.05, 0.22) | 0.05 (0.02, 0.11) | 2.18 (1.6, 3.18) |
| Netherlands | 1.65 (1.45, 1.88) | 0.78 (0.71, 0.86) | 0.05 (0.04, 0.06) | 0.05 (0.04, 0.06) | 0.03 (0.03, 0.04) | 2.55 (2.25, 2.9) |
| Norway | 1.3 (1.1, 1.53) | 0.89 (0.78, 1.01) | 0.62 (0.26, 1.76) | 0.01 (0, 0.03) | 0.03 (0.02, 0.03) | 2.85 (2.17, 4.36) |
| Nepal | 0.15 (0.13, 0.17) | 0.31 (0.29, 0.34) | 0.1 (0.09, 0.12) | 0.11 (0.09, 0.13) | 0.06 (0.02, 0.2) | 0.73 (0.62, 0.96) |
| New Zealand | 2.44 (1.96, 3.03) | 0.88 (0.74, 1.06) | 0.27 (0.12, 0.76) | 0.56 (0.42, 0.72) | 0.01 (0.01, 0.02) | 4.16 (3.25, 5.59) |
| Oman | 0.33 (0.24, 0.46) | 0.24 (0.19, 0.32) | 0.04 (0.02, 0.12) | 0.11 (0.05, 0.29) | 0.15 (0.07, 0.35) | 0.88 (0.56, 1.53) |
| Pakistan | 0.14 (0.12, 0.15) | 0.15 (0.13, 0.17) | 0.06 (0.05, 0.08) | 0.02 (0.02, 0.03) | 0.04 (0.01, 0.12) | 0.41 (0.34, 0.55) |
| Panama | 0.6 (0.48, 0.75) | 0.58 (0.46, 0.74) | 0.12 (0.06, 0.26) | 0.24 (0.12, 0.51) | 0.14 (0.07, 0.3) | 1.67 (1.18, 2.57) |
| Peru | 1.04 (0.96, 1.13) | 0.37 (0.34, 0.4) | 0.24 (0.22, 0.27) | 0.13 (0.11, 0.14) | 0.06 (0.03, 0.14) | 1.84 (1.65, 2.08) |
| Philippines | 0.7 (0.63, 0.77) | 0.69 (0.64, 0.76) | 0.1 (0.09, 0.12) | 0.08 (0.07, 0.09) | 0.01 (0, 0.01) | 1.59 (1.44, 1.75) |
| Papua New Guinea | 0.03 (0.02, 0.05) | 0.53 (0.43, 0.64) | 0.45 (0.18, 1.23) | 0.04 (0.02, 0.1) | 0 (0, 0) | 1.05 (0.65, 2.03) |
| Poland | 1.16 (1, 1.35) | 0.52 (0.44, 0.61) | 0 (0, 0) | 0.01 (0.01, 0.01) | 0.05 (0.04, 0.08) | 1.74 (1.48, 2.07) |
| Portugal | 6.52 (5.73, 7.28) | 1.04 (0.94, 1.15) | 0.3 (0.26, 0.35) | 0.11 (0.09, 0.13) | 0.02 (0.02, 0.03) | 7.99 (7.04, 8.92) |
| Paraguay | 0.43 (0.36, 0.51) | 0.26 (0.23, 0.3) | 0.13 (0.06, 0.28) | 0.06 (0.03, 0.12) | 0.05 (0.03, 0.11) | 0.93 (0.71, 1.33) |
| Palestinian Territories | 0.3 (0.21, 0.44) | 0.24 (0.17, 0.33) | 0.09 (0.04, 0.24) | 0.1 (0.04, 0.25) | 0.18 (0.08, 0.43) | 0.92 (0.54, 1.69) |
| Qatar | 0.33 (0.22, 0.48) | 0.25 (0.18, 0.35) | 0.07 (0.03, 0.22) | 0.1 (0.04, 0.26) | 0.17 (0.07, 0.41) | 0.92 (0.54, 1.72) |
| Romania | 0.96 (0.78, 1.18) | 1.03 (0.85, 1.26) | 0.03 (0.02, 0.05) | 0.11 (0.08, 0.16) | 0.16 (0.09, 0.28) | 2.3 (1.82, 2.92) |
| Russia | 0.45 (0.38, 0.53) | 0.53 (0.44, 0.63) | 0.07 (0.03, 0.17) | 0 (0, 0.01) | 0.24 (0.15, 0.4) | 1.29 (1.01, 1.73) |
| Rwanda | 0.38 (0.33, 0.43) | 0.88 (0.79, 0.97) | 0.12 (0.1, 0.14) | 0.25 (0.22, 0.29) | 0.13 (0.06, 0.33) | 1.76 (1.51, 2.16) |
| Saudi Arabia | 0.4 (0.3, 0.52) | 0.23 (0.17, 0.31) | 0.07 (0.03, 0.19) | 0.15 (0.06, 0.37) | 0.23 (0.1, 0.52) | 1.07 (0.67, 1.91) |
| Sudan | 0.26 (0.18, 0.37) | 0.41 (0.29, 0.58) | 0.04 (0.02, 0.08) | 0.07 (0.04, 0.15) | 0.27 (0.13, 0.62) | 1.05 (0.65, 1.8) |
| Senegal | 0.16 (0.15, 0.18) | 0.23 (0.21, 0.26) | 0.07 (0.06, 0.08) | 0.06 (0.05, 0.07) | 0.14 (0.06, 0.34) | 0.67 (0.53, 0.93) |
| Singapore | 1.09 (0.9, 1.34) | 0.89 (0.75, 1.05) | 0.08 (0.03, 0.22) | 0.13 (0.1, 0.17) | 0.04 (0.03, 0.05) | 2.23 (1.8, 2.83) |
| Solomon Islands | 0.5 (0.39, 0.65) | 1.14 (0.88, 1.48) | 0.1 (0.04, 0.24) | 0.25 (0.13, 0.56) | 0.02 (0.01, 0.04) | 2.01 (1.45, 2.97) |
| Sierra Leone | 0.28 (0.24, 0.32) | 0.37 (0.34, 0.42) | 0.03 (0.03, 0.04) | 0.07 (0.06, 0.08) | 0.05 (0.02, 0.11) | 0.8 (0.69, 0.97) |
| El Salvador | 0.56 (0.38, 0.83) | 0.49 (0.35, 0.69) | 0.19 (0.09, 0.45) | 0.13 (0.06, 0.3) | 0.03 (0.01, 0.06) | 1.4 (0.9, 2.32) |
| Serbia | 0.73 (0.48, 1.15) | 1.72 (1.1, 2.63) | 0.18 (0.08, 0.41) | 0.11 (0.06, 0.22) | 0.06 (0.02, 0.15) | 2.8 (1.75, 4.56) |
| South Sudan | 0.2 (0.16, 0.26) | 0.46 (0.34, 0.62) | 0.06 (0.03, 0.13) | 0.09 (0.05, 0.19) | 0.15 (0.07, 0.34) | 0.95 (0.64, 1.55) |
| São Tomé and Príncipe | 0.4 (0.33, 0.49) | 0.11 (0.1, 0.14) | 0.11 (0.1, 0.14) | 0.03 (0.02, 0.04) | 0.17 (0.07, 0.42) | 0.82 (0.62, 1.22) |
| Suriname | 0.47 (0.32, 0.69) | 0.7 (0.5, 0.97) | 0.13 (0.06, 0.3) | 0.27 (0.14, 0.56) | 0.14 (0.07, 0.31) | 1.7 (1.09, 2.82) |
| Slovakia | 0.4 (0.33, 0.47) | 0.29 (0.24, 0.35) | 0.01 (0, 0.01) | 0.02 (0.01, 0.03) | 0.05 (0.03, 0.08) | 0.76 (0.62, 0.95) |
| Slovenia | 0.38 (0.31, 0.46) | 0.33 (0.27, 0.4) | 0.25 (0.11, 0.6) | 0.07 (0.03, 0.15) | 0.14 (0.06, 0.31) | 1.16 (0.79, 1.92) |
| Sweden | 1.79 (1.56, 2.07) | 1.02 (0.9, 1.13) | 0.35 (0.29, 0.43) | 0.14 (0.11, 0.17) | 0.04 (0.03, 0.05) | 3.34 (2.9, 3.85) |
| Eswatini | 0.28 (0.25, 0.32) | 0.51 (0.44, 0.59) | 0.05 (0.04, 0.06) | 0.03 (0.03, 0.04) | 0.13 (0.09, 0.2) | 1.01 (0.85, 1.21) |
| Seychelles | 0.32 (0.28, 0.38) | 0.63 (0.56, 0.72) | 0.02 (0.01, 0.02) | 0.06 (0.05, 0.07) | 0.17 (0.08, 0.41) | 1.21 (0.98, 1.6) |
| Syria | 0.28 (0.21, 0.38) | 0.21 (0.16, 0.27) | 0.08 (0.04, 0.22) | 0.1 (0.05, 0.25) | 0.18 (0.08, 0.41) | 0.86 (0.53, 1.52) |
| Chad | 0.14 (0.12, 0.15) | 0.12 (0.11, 0.13) | 0.06 (0.05, 0.07) | 0.02 (0.02, 0.03) | 0.18 (0.08, 0.42) | 0.51 (0.38, 0.81) |
| Togo | 0.17 (0.14, 0.21) | 0.47 (0.39, 0.57) | 0.05 (0.04, 0.06) | 0.04 (0.03, 0.06) | 0.18 (0.09, 0.4) | 0.91 (0.68, 1.3) |
| Thailand | 0.78 (0.58, 1.04) | 1.04 (0.81, 1.35) | 0.07 (0.03, 0.16) | 0.35 (0.14, 0.86) | 0.02 (0.01, 0.04) | 2.25 (1.57, 3.46) |
| Tajikistan | 0.5 (0.4, 0.62) | 0.2 (0.16, 0.25) | 0.13 (0.11, 0.16) | 0.22 (0.17, 0.28) | 0.06 (0.03, 0.14) | 1.11 (0.86, 1.46) |
| Turkmenistan | 0.36 (0.29, 0.47) | 0.37 (0.27, 0.49) | 0.06 (0.03, 0.15) | 0.11 (0.06, 0.26) | 0.01 (0, 0.02) | 0.92 (0.65, 1.39) |
| Timor-Leste | 0.43 (0.37, 0.51) | 0.91 (0.81, 1.03) | 0.1 (0.08, 0.12) | 0.08 (0.06, 0.1) | 0.02 (0.01, 0.05) | 1.54 (1.34, 1.81) |
| Tonga | 1.08 (0.85, 1.38) | 0.89 (0.74, 1.06) | 0.07 (0.03, 0.2) | 0.33 (0.15, 0.81) | 0.05 (0.02, 0.12) | 2.41 (1.8, 3.57) |
| Trinidad & Tobago | 0.31 (0.27, 0.36) | 0.33 (0.28, 0.37) | 0.09 (0.07, 0.11) | 0.14 (0.12, 0.17) | 0.08 (0.04, 0.18) | 0.95 (0.76, 1.2) |
| Tunisia | 0.17 (0.14, 0.2) | 0.3 (0.25, 0.35) | 0.1 (0.04, 0.27) | 0.04 (0.02, 0.07) | 0.06 (0.04, 0.09) | 0.67 (0.5, 0.97) |
| Turkey | 0.21 (0.18, 0.25) | 0.17 (0.15, 0.2) | 0.09 (0.03, 0.23) | 0.08 (0.05, 0.13) | 0.1 (0.06, 0.17) | 0.65 (0.47, 0.98) |
| Taiwan | 1.12 (0.9, 1.39) | 1.64 (1.38, 1.95) | 0.08 (0.04, 0.21) | 0.05 (0.04, 0.07) | 0.04 (0.03, 0.05) | 2.93 (2.39, 3.67) |
| Tanzania | 0.21 (0.18, 0.23) | 0.54 (0.49, 0.6) | 0.05 (0.04, 0.06) | 0.11 (0.1, 0.12) | 0.31 (0.15, 0.72) | 1.22 (0.96, 1.74) |
| Uganda | 0.18 (0.16, 0.2) | 0.36 (0.32, 0.39) | 0.05 (0.05, 0.06) | 0.15 (0.13, 0.17) | 0.15 (0.07, 0.35) | 0.88 (0.72, 1.17) |
| Ukraine | 0.46 (0.38, 0.56) | 0.53 (0.43, 0.65) | 0.08 (0.04, 0.21) | 0.03 (0.01, 0.07) | 0.19 (0.08, 0.47) | 1.29 (0.94, 1.95) |
| Uruguay | 0.43 (0.37, 0.5) | 0.61 (0.53, 0.68) | 0.12 (0.05, 0.33) | 0.16 (0.07, 0.39) | 0.23 (0.1, 0.54) | 1.55 (1.14, 2.44) |
| United States | 1.38 (1.24, 1.55) | 0.88 (0.81, 0.96) | 0.21 (0.18, 0.25) | 0.18 (0.15, 0.21) | 0.09 (0.08, 0.1) | 2.74 (2.47, 3.07) |
| Uzbekistan | 0.44 (0.35, 0.55) | 0.74 (0.55, 0.97) | 0.17 (0.14, 0.21) | 0.12 (0.06, 0.26) | 0.04 (0.02, 0.1) | 1.5 (1.11, 2.09) |
| St. Vincent & Grenadines | 0.65 (0.43, 0.96) | 0.39 (0.28, 0.54) | 0.11 (0.06, 0.26) | 0.17 (0.09, 0.36) | 0.05 (0.03, 0.11) | 1.38 (0.88, 2.23) |
| Venezuela | 0.49 (0.34, 0.71) | 0.35 (0.25, 0.48) | 0.16 (0.07, 0.42) | 0.14 (0.07, 0.32) | 0.01 (0, 0.02) | 1.16 (0.73, 1.95) |
| Vietnam | 0.68 (0.58, 0.79) | 1.08 (0.96, 1.21) | 0.22 (0.14, 0.34) | 0.86 (0.56, 1.3) | 0.31 (0.15, 0.66) | 3.15 (2.39, 4.32) |
| Vanuatu | 0.35 (0.27, 0.44) | 0.47 (0.36, 0.6) | 0.09 (0.04, 0.21) | 0.6 (0.28, 1.41) | 0.06 (0.03, 0.14) | 1.56 (0.98, 2.79) |
| Samoa | 1.17 (0.91, 1.53) | 2.19 (1.77, 2.67) | 0.07 (0.03, 0.18) | 2.48 (1.21, 5.17) | 0.43 (0.21, 0.92) | 6.35 (4.13, 10.47) |
| Yemen | 0.11 (0.09, 0.14) | 0.1 (0.08, 0.12) | 0.09 (0.07, 0.11) | 0.08 (0.07, 0.1) | 0.03 (0.01, 0.08) | 0.42 (0.33, 0.54) |
| South Africa | 0.1 (0.09, 0.11) | 0.39 (0.36, 0.43) | 0.07 (0.03, 0.15) | 0.1 (0.07, 0.13) | 0 (0, 0.01) | 0.66 (0.56, 0.83) |
| Zambia | 0.16 (0.15, 0.18) | 0.62 (0.58, 0.68) | 0.04 (0.03, 0.05) | 0.07 (0.06, 0.08) | 0.39 (0.19, 0.81) | 1.28 (1, 1.79) |
| Zimbabwe | 0.26 (0.24, 0.29) | 0.45 (0.41, 0.48) | 0.06 (0.05, 0.07) | 0.06 (0.05, 0.07) | 0.06 (0.04, 0.09) | 0.89 (0.79, 1) |

Table S15: National consumption (servings/day) in children 2-19 years in 2018.

| Country | Fruit | Vegetables (non-starchy) | Other non-potato starchy vegetables | Beans and legumes | Nuts and seeds | Total Plant-Based Food Consumption |
| --- | --- | --- | --- | --- | --- | --- |
| Afghanistan | 0.67 (0.5, 0.9) | 0.85 (0.58, 1.21) | 0.36 (0.15, 0.92) | 0.97 (0.42, 2.01) | 0.98 (0.35, 2.15) | 3.83 (2, 7.19) |
| Angola | 1.16 (0.97, 1.39) | 2.91 (2.43, 3.4) | 0.32 (0.2, 0.55) | 0.39 (0.24, 0.66) | 0.39 (0.21, 0.79) | 5.17 (4.05, 6.79) |
| Albania | 1.1 (0.93, 1.29) | 0.64 (0.54, 0.77) | 0.51 (0.39, 0.69) | 0.37 (0.29, 0.47) | 0.63 (0.35, 1.12) | 3.24 (2.49, 4.33) |
| United Arab Emirates | 0.89 (0.75, 1.05) | 0.84 (0.73, 0.96) | 0.43 (0.2, 0.9) | 0.38 (0.18, 0.81) | 0.83 (0.41, 1.52) | 3.37 (2.28, 5.24) |
| Argentina | 0.94 (0.82, 1.07) | 1.09 (0.95, 1.23) | 0.05 (0.04, 0.07) | 0.04 (0.03, 0.05) | 0.04 (0.03, 0.05) | 2.16 (1.87, 2.47) |
| Armenia | 0.69 (0.57, 0.85) | 0.45 (0.37, 0.56) | 0.65 (0.51, 0.83) | 0.13 (0.09, 0.19) | 0.96 (0.58, 1.64) | 2.88 (2.12, 4.06) |
| Antigua & Barbuda | 0.95 (0.66, 1.38) | 2.04 (1.46, 2.8) | 0.37 (0.19, 0.72) | 0.45 (0.24, 0.85) | 0.06 (0.03, 0.13) | 3.88 (2.59, 5.89) |
| Australia | 0.9 (0.75, 1.08) | 0.64 (0.54, 0.75) | 0.06 (0.03, 0.15) | 0.16 (0.11, 0.22) | 0.19 (0.16, 0.24) | 1.94 (1.58, 2.44) |
| Austria | 0.69 (0.61, 0.78) | 0.62 (0.56, 0.69) | 0.1 (0.05, 0.24) | 0.04 (0.03, 0.07) | 0.14 (0.11, 0.17) | 1.6 (1.36, 1.95) |
| Azerbaijan | 0.81 (0.68, 0.96) | 0.38 (0.31, 0.45) | 0.29 (0.24, 0.36) | 0.13 (0.1, 0.17) | 1.49 (0.9, 2.23) | 3.09 (2.23, 4.17) |
| Burundi | 0.71 (0.57, 0.88) | 1.72 (1.44, 2.03) | 0.23 (0.18, 0.28) | 0.42 (0.34, 0.53) | 0.22 (0.11, 0.49) | 3.3 (2.65, 4.2) |
| Belgium | 0.69 (0.62, 0.77) | 0.61 (0.54, 0.68) | 0.08 (0.04, 0.19) | 0.03 (0.02, 0.05) | 0.09 (0.07, 0.1) | 1.49 (1.28, 1.79) |
| Benin | 0.72 (0.64, 0.82) | 0.91 (0.82, 1.01) | 0.13 (0.11, 0.15) | 0.26 (0.22, 0.3) | 0.44 (0.23, 0.89) | 2.46 (2.02, 3.16) |
| Burkina Faso | 0.34 (0.29, 0.39) | 0.25 (0.22, 0.28) | 0.04 (0.03, 0.04) | 0.1 (0.08, 0.12) | 0.74 (0.26, 2.17) | 1.46 (0.88, 3) |
| Bangladesh | 0.56 (0.51, 0.61) | 1.88 (1.76, 2.02) | 0.1 (0.08, 0.11) | 0.27 (0.24, 0.3) | 0.02 (0.02, 0.02) | 2.82 (2.61, 3.07) |
| Bulgaria | 0.73 (0.6, 0.87) | 1.81 (1.57, 2.08) | 0.03 (0.01, 0.06) | 0.16 (0.11, 0.22) | 0.67 (0.46, 0.98) | 3.39 (2.75, 4.22) |
| Bahrain | 0.98 (0.79, 1.23) | 1.26 (0.96, 1.68) | 0.23 (0.11, 0.53) | 0.36 (0.18, 0.78) | 0.56 (0.27, 1.08) | 3.39 (2.31, 5.29) |
| Bahamas | 0.85 (0.57, 1.25) | 0.94 (0.68, 1.32) | 0.39 (0.19, 0.75) | 0.19 (0.1, 0.38) | 0.25 (0.13, 0.53) | 2.62 (1.67, 4.23) |
| Bosnia & Herzegovina | 4.46 (3.89, 4.87) | 2.3 (2.01, 2.64) | 0.27 (0.14, 0.55) | 0.12 (0.07, 0.25) | 0.49 (0.26, 0.86) | 7.65 (6.38, 9.16) |
| Belarus | 1.85 (1.01, 3.25) | 0.39 (0.22, 0.7) | 0.29 (0.15, 0.61) | 0.64 (0.35, 1.2) | 0.58 (0.31, 1.05) | 3.75 (2.03, 6.81) |
| Belize | 0.86 (0.6, 1.24) | 1.35 (0.96, 1.89) | 0.33 (0.16, 0.67) | 0.28 (0.15, 0.58) | 0.06 (0.03, 0.12) | 2.89 (1.9, 4.5) |
| Bolivia | 0.87 (0.78, 0.97) | 1.01 (0.92, 1.12) | 0.53 (0.46, 0.6) | 0.12 (0.1, 0.14) | 0.31 (0.16, 0.62) | 2.84 (2.42, 3.45) |
| Brazil | 0.86 (0.77, 0.95) | 0.83 (0.75, 0.91) | 0.07 (0.05, 0.09) | 0.88 (0.74, 1.02) | 0.01 (0, 0.01) | 2.63 (2.32, 2.97) |
| Barbados | 1.29 (1.07, 1.57) | 1.25 (1.05, 1.47) | 0.33 (0.17, 0.67) | 0.07 (0.05, 0.12) | 0.23 (0.15, 0.36) | 3.18 (2.48, 4.18) |
| Brunei | 0.88 (0.63, 1.23) | 1.4 (1.06, 1.85) | 0.11 (0.05, 0.24) | 0.33 (0.18, 0.63) | 0.12 (0.06, 0.24) | 2.83 (1.98, 4.18) |
| Bhutan | 0.99 (0.74, 1.33) | 2.48 (1.76, 3.48) | 0.17 (0.08, 0.42) | 0.64 (0.28, 1.37) | 0.19 (0.07, 0.51) | 4.47 (2.93, 7.12) |
| Botswana | 0.48 (0.37, 0.63) | 1.01 (0.82, 1.25) | 0.21 (0.13, 0.36) | 0.2 (0.12, 0.33) | 1.34 (0.76, 2.09) | 3.24 (2.2, 4.66) |
| Central African Republic | 0.96 (0.81, 1.16) | 0.97 (0.79, 1.21) | 0.58 (0.48, 0.71) | 0.39 (0.24, 0.67) | 0.27 (0.14, 0.54) | 3.18 (2.45, 4.29) |
| Canada | 0.91 (0.83, 0.99) | 0.7 (0.66, 0.75) | 0.06 (0.05, 0.07) | 0.13 (0.11, 0.15) | 0.45 (0.4, 0.5) | 2.24 (2.04, 2.46) |
| Switzerland | 0.91 (0.78, 1.06) | 0.61 (0.53, 0.7) | 0.15 (0.07, 0.33) | 0.02 (0.01, 0.02) | 0.18 (0.11, 0.33) | 1.85 (1.5, 2.43) |
| Chile | 1.08 (0.89, 1.31) | 1.22 (1.03, 1.45) | 0.66 (0.33, 1.23) | 0.14 (0.08, 0.24) | 0.46 (0.23, 0.9) | 3.56 (2.56, 5.13) |
| China | 0.92 (0.84, 1.01) | 2.55 (2.33, 2.77) | 0.13 (0.07, 0.26) | 0.14 (0.12, 0.18) | 0.25 (0.21, 0.31) | 3.99 (3.56, 4.54) |
| Côte d’Ivoire | 0.59 (0.5, 0.68) | 0.85 (0.74, 0.98) | 0.18 (0.14, 0.23) | 0.05 (0.03, 0.07) | 0.32 (0.17, 0.67) | 1.99 (1.58, 2.62) |
| Cameroon | 0.8 (0.71, 0.91) | 1.15 (1.03, 1.27) | 0.23 (0.2, 0.27) | 0.32 (0.27, 0.37) | 0.51 (0.27, 0.95) | 3.01 (2.48, 3.78) |
| Congo - Kinshasa | 0.92 (0.81, 1.04) | 1.97 (1.78, 2.19) | 0.27 (0.23, 0.32) | 0.32 (0.27, 0.38) | 0.89 (0.39, 2.02) | 4.38 (3.49, 5.95) |
| Congo - Brazzaville | 0.87 (0.77, 0.98) | 1.3 (1.18, 1.44) | 0.24 (0.2, 0.29) | 0.24 (0.2, 0.29) | 0.33 (0.17, 0.67) | 2.97 (2.51, 3.66) |
| Colombia | 1.01 (0.93, 1.1) | 0.86 (0.79, 0.94) | 0.71 (0.62, 0.8) | 0.27 (0.24, 0.31) | 1.27 (0.74, 2.01) | 4.13 (3.32, 5.16) |
| Comoros | 0.82 (0.7, 0.95) | 0.88 (0.78, 1.01) | 0.25 (0.21, 0.3) | 0.1 (0.07, 0.14) | 0.26 (0.14, 0.53) | 2.32 (1.9, 2.94) |
| Cape Verde | 2.25 (1.61, 3.05) | 1.74 (1.32, 2.29) | 0.16 (0.09, 0.3) | 0.32 (0.2, 0.56) | 0.5 (0.25, 1) | 4.95 (3.46, 7.2) |
| Costa Rica | 1.31 (0.92, 1.89) | 1.46 (1.05, 2) | 0.36 (0.19, 0.7) | 0.29 (0.16, 0.54) | 0.22 (0.11, 0.45) | 3.65 (2.44, 5.59) |
| Cuba | 1.29 (1.07, 1.57) | 0.99 (0.81, 1.24) | 0.45 (0.23, 0.85) | 0.28 (0.15, 0.53) | 0.01 (0, 0.02) | 3.02 (2.26, 4.2) |
| Cyprus | 0.41 (0.25, 0.66) | 0.65 (0.4, 1.08) | 0.05 (0.02, 0.11) | 0.21 (0.08, 0.53) | 0.12 (0.05, 0.27) | 1.43 (0.8, 2.65) |
| Czechia | 0.56 (0.5, 0.63) | 0.47 (0.43, 0.52) | 0.27 (0.14, 0.56) | 0.11 (0.06, 0.21) | 0.17 (0.12, 0.24) | 1.58 (1.24, 2.18) |
| Germany | 0.91 (0.83, 1) | 1.01 (0.94, 1.09) | 0.11 (0.09, 0.13) | 0.03 (0.03, 0.04) | 0.08 (0.07, 0.09) | 2.15 (1.96, 2.35) |
| Djibouti | 1.38 (1.14, 1.69) | 0.95 (0.77, 1.19) | 0.14 (0.07, 0.31) | 2.04 (1.36, 2.84) | 0 (0, 0.01) | 4.51 (3.33, 6.05) |
| Dominica | 1.06 (0.84, 1.33) | 0.91 (0.75, 1.12) | 0.42 (0.22, 0.81) | 0.32 (0.18, 0.6) | 0.12 (0.06, 0.23) | 2.82 (2.05, 4.1) |
| Denmark | 0.6 (0.5, 0.7) | 0.65 (0.57, 0.75) | 0.09 (0.04, 0.23) | 0.15 (0.08, 0.3) | 0.22 (0.16, 0.3) | 1.71 (1.36, 2.29) |
| Dominican Republic | 1.44 (1.31, 1.59) | 0.93 (0.84, 1.03) | 0.41 (0.36, 0.47) | 0.48 (0.42, 0.54) | 0.04 (0.02, 0.07) | 3.29 (2.94, 3.7) |
| Algeria | 0.65 (0.52, 0.83) | 1.35 (1.12, 1.62) | 0.3 (0.14, 0.66) | 0.18 (0.06, 0.56) | 0.15 (0.08, 0.32) | 2.63 (1.92, 3.99) |
| Ecuador | 0.26 (0.23, 0.3) | 0.97 (0.86, 1.1) | 0.2 (0.09, 0.44) | 0.16 (0.09, 0.28) | 0.07 (0.03, 0.16) | 1.67 (1.3, 2.29) |
| Egypt | 0.75 (0.68, 0.82) | 1.07 (0.98, 1.18) | 0.2 (0.18, 0.23) | 0.09 (0.07, 0.1) | 0.06 (0.04, 0.08) | 2.17 (1.95, 2.42) |
| Eritrea | 0.55 (0.4, 0.76) | 0.63 (0.5, 0.81) | 0.16 (0.09, 0.29) | 0.29 (0.18, 0.5) | 0.34 (0.18, 0.69) | 1.97 (1.35, 3.04) |
| Spain | 0.42 (0.37, 0.47) | 0.54 (0.48, 0.59) | 0.05 (0.02, 0.11) | 0.14 (0.06, 0.3) | 0.21 (0.16, 0.29) | 1.35 (1.1, 1.75) |
| Estonia | 0.8 (0.72, 0.88) | 1.33 (1.22, 1.45) | 0.02 (0.02, 0.03) | 0.05 (0.04, 0.07) | 0.45 (0.34, 0.6) | 2.65 (2.33, 3.04) |
| Ethiopia | 0.37 (0.33, 0.42) | 0.82 (0.74, 0.9) | 0.14 (0.12, 0.16) | 0.36 (0.31, 0.41) | 0.06 (0.04, 0.09) | 1.75 (1.55, 1.98) |
| Finland | 0.99 (0.88, 1.1) | 0.67 (0.61, 0.73) | 0.03 (0.01, 0.07) | 0.04 (0.03, 0.05) | 0.08 (0.07, 0.09) | 1.8 (1.6, 2.05) |
| Fiji | 1.15 (0.78, 1.67) | 1.82 (1.32, 2.48) | 0.16 (0.08, 0.34) | 0.28 (0.15, 0.52) | 0.13 (0.07, 0.25) | 3.53 (2.4, 5.25) |
| France | 1 (0.91, 1.1) | 0.84 (0.78, 0.91) | 0 (0, 0.01) | 0.09 (0.08, 0.11) | 0.06 (0.06, 0.07) | 2.01 (1.83, 2.2) |
| Micronesia (Federated States of) | 1.53 (1.1, 2.12) | 1.22 (0.83, 1.83) | 0.16 (0.08, 0.34) | 0.27 (0.14, 0.54) | 0.08 (0.05, 0.17) | 3.26 (2.2, 4.99) |
| Gabon | 0.66 (0.57, 0.76) | 1.09 (0.96, 1.24) | 0.29 (0.25, 0.35) | 0.03 (0.02, 0.03) | 0.67 (0.34, 1.28) | 2.74 (2.14, 3.66) |
| United Kingdom | 0.61 (0.56, 0.67) | 0.63 (0.58, 0.69) | 0.07 (0.03, 0.16) | 0.27 (0.22, 0.33) | 0.14 (0.12, 0.17) | 1.71 (1.5, 2.01) |
| Georgia | 0.55 (0.47, 0.64) | 0.41 (0.36, 0.47) | 0.17 (0.09, 0.36) | 0.3 (0.16, 0.6) | 0.32 (0.17, 0.62) | 1.75 (1.25, 2.68) |
| Ghana | 0.84 (0.77, 0.92) | 1.25 (1.16, 1.34) | 0.19 (0.16, 0.22) | 0.2 (0.18, 0.24) | 0.99 (0.55, 1.74) | 3.47 (2.82, 4.45) |
| Guinea | 0.21 (0.18, 0.26) | 0.19 (0.15, 0.23) | 0.09 (0.07, 0.11) | 0.03 (0.03, 0.04) | 0.36 (0.19, 0.71) | 0.89 (0.62, 1.35) |
| Gambia | 0.3 (0.23, 0.38) | 0.44 (0.35, 0.55) | 0.08 (0.06, 0.1) | 0.07 (0.06, 0.1) | 0.4 (0.2, 0.85) | 1.3 (0.9, 1.98) |
| Guinea-Bissau | 0.65 (0.54, 0.78) | 1.27 (1.03, 1.55) | 0.19 (0.11, 0.36) | 0.94 (0.59, 1.52) | 0.43 (0.23, 0.85) | 3.47 (2.51, 5.06) |
| Equatorial Guinea | 0.86 (0.72, 1.03) | 1.31 (1.05, 1.66) | 0.27 (0.16, 0.47) | 0.38 (0.24, 0.65) | 0.38 (0.19, 0.76) | 3.19 (2.36, 4.56) |
| Greece | 0.5 (0.43, 0.57) | 0.48 (0.42, 0.55) | 0.05 (0.03, 0.12) | 0.09 (0.07, 0.12) | 0.19 (0.15, 0.24) | 1.31 (1.1, 1.61) |
| Grenada | 1.2 (0.83, 1.75) | 2.19 (1.58, 3.05) | 0.41 (0.22, 0.77) | 0.29 (0.16, 0.55) | 0 (0, 0) | 4.1 (2.79, 6.12) |
| Guatemala | 0.76 (0.69, 0.85) | 0.85 (0.78, 0.93) | 0.27 (0.24, 0.31) | 0.48 (0.42, 0.56) | 0.07 (0.04, 0.14) | 2.44 (2.16, 2.79) |
| Guyana | 2.05 (1.75, 2.41) | 1.31 (1.14, 1.51) | 0.34 (0.29, 0.41) | 0.23 (0.18, 0.28) | 0.22 (0.11, 0.43) | 4.15 (3.48, 5.03) |
| Honduras | 0.9 (0.8, 1.02) | 0.27 (0.24, 0.31) | 0.26 (0.22, 0.3) | 0.43 (0.38, 0.5) | 0.12 (0.09, 0.18) | 1.99 (1.72, 2.31) |
| Croatia | 0.46 (0.4, 0.53) | 3.43 (3.06, 3.79) | 0.31 (0.16, 0.6) | 0.07 (0.03, 0.19) | 0.51 (0.22, 1.16) | 4.78 (3.86, 6.26) |
| Haiti | 0.71 (0.63, 0.8) | 0.73 (0.65, 0.82) | 0.17 (0.15, 0.2) | 0.28 (0.24, 0.33) | 0.14 (0.1, 0.2) | 2.03 (1.76, 2.35) |
| Hungary | 0.46 (0.41, 0.52) | 0.29 (0.26, 0.31) | 0.24 (0.13, 0.52) | 0.14 (0.05, 0.44) | 0.09 (0.07, 0.12) | 1.22 (0.91, 1.91) |
| Indonesia | 0.7 (0.65, 0.76) | 1.38 (1.28, 1.49) | 0.25 (0.22, 0.29) | 0.27 (0.24, 0.3) | 0.15 (0.12, 0.18) | 2.75 (2.51, 3.03) |
| India | 0.36 (0.33, 0.4) | 1.4 (1.3, 1.5) | 0.11 (0.1, 0.14) | 0.23 (0.2, 0.26) | 0.09 (0.07, 0.12) | 2.2 (2.01, 2.41) |
| Ireland | 0.54 (0.44, 0.65) | 0.64 (0.55, 0.74) | 0.08 (0.04, 0.18) | 0.15 (0.07, 0.33) | 0.34 (0.17, 0.68) | 1.75 (1.26, 2.58) |
| Iran | 1.38 (1.27, 1.5) | 1.56 (1.44, 1.69) | 0.07 (0.05, 0.09) | 0.15 (0.13, 0.18) | 0.19 (0.16, 0.23) | 3.35 (3.05, 3.69) |
| Iraq | 0.91 (0.63, 1.3) | 0.89 (0.65, 1.21) | 0.26 (0.12, 0.57) | 0.36 (0.18, 0.74) | 0.37 (0.18, 0.76) | 2.78 (1.76, 4.58) |
| Iceland | 0.52 (0.45, 0.6) | 0.38 (0.34, 0.43) | 0.43 (0.2, 0.96) | 0.04 (0.03, 0.05) | 0.04 (0.03, 0.05) | 1.41 (1.05, 2.08) |
| Israel | 1.1 (0.98, 1.23) | 0.58 (0.53, 0.63) | 0.4 (0.32, 0.5) | 0.69 (0.56, 0.85) | 0.8 (0.67, 0.95) | 3.56 (3.05, 4.17) |
| Italy | 1.28 (1.16, 1.41) | 0.77 (0.71, 0.85) | 0.07 (0.06, 0.08) | 0.09 (0.08, 0.11) | 0.09 (0.07, 0.1) | 2.3 (2.08, 2.55) |
| Jamaica | 1.77 (1.38, 2.27) | 1.32 (1.09, 1.61) | 0.3 (0.16, 0.56) | 0.12 (0.08, 0.18) | 0.08 (0.05, 0.12) | 3.59 (2.76, 4.74) |
| Jordan | 1.49 (1.34, 1.66) | 0.89 (0.81, 0.98) | 0.16 (0.14, 0.19) | 0.26 (0.22, 0.3) | 0.31 (0.23, 0.42) | 3.11 (2.73, 3.55) |
| Japan | 1.06 (1.02, 1.11) | 2.31 (2.24, 2.39) | 0.06 (0.04, 0.09) | 0.52 (0.45, 0.61) | 0.06 (0.06, 0.06) | 4.02 (3.8, 4.27) |
| Kazakhstan | 0.46 (0.39, 0.53) | 0.76 (0.68, 0.86) | 0.35 (0.28, 0.44) | 0.06 (0.03, 0.11) | 3.06 (2.35, 3.54) | 4.69 (3.73, 5.49) |
| Kenya | 1.01 (0.92, 1.12) | 2.27 (2.11, 2.45) | 0.36 (0.31, 0.41) | 0.3 (0.26, 0.34) | 0.02 (0.01, 0.03) | 3.96 (3.61, 4.35) |
| Kyrgyzstan | 0.6 (0.5, 0.72) | 0.2 (0.15, 0.27) | 0.35 (0.29, 0.43) | 0.06 (0.04, 0.08) | 1.61 (1, 2.36) | 2.82 (1.98, 3.86) |
| Cambodia | 0.51 (0.46, 0.57) | 1.03 (0.93, 1.12) | 0.16 (0.14, 0.18) | 0.04 (0.03, 0.04) | 0.04 (0.03, 0.07) | 1.78 (1.59, 1.99) |
| Kiribati | 1.02 (0.69, 1.53) | 0.77 (0.54, 1.11) | 0.17 (0.08, 0.35) | 0.28 (0.15, 0.54) | 0.09 (0.05, 0.18) | 2.34 (1.51, 3.7) |
| South Korea | 0.98 (0.93, 1.05) | 1.15 (1.1, 1.21) | 0.04 (0.04, 0.05) | 0.26 (0.24, 0.29) | 0.04 (0.04, 0.05) | 2.48 (2.33, 2.63) |
| Kuwait | 0.47 (0.39, 0.55) | 0.74 (0.64, 0.85) | 0.15 (0.07, 0.37) | 0.44 (0.2, 0.96) | 0.67 (0.32, 1.29) | 2.47 (1.62, 4.02) |
| Laos | 1.25 (1.11, 1.41) | 1.24 (1.13, 1.36) | 0.06 (0.04, 0.09) | 0.25 (0.13, 0.49) | 0.02 (0.02, 0.03) | 2.82 (2.43, 3.38) |
| Lebanon | 1.91 (1.67, 2.19) | 2.53 (2.24, 2.84) | 0.13 (0.1, 0.17) | 0.28 (0.22, 0.33) | 1.23 (1.01, 1.5) | 6.08 (5.24, 7.04) |
| Liberia | 0.48 (0.4, 0.57) | 1.12 (0.97, 1.28) | 0.2 (0.16, 0.24) | 0.1 (0.08, 0.12) | 0.08 (0.04, 0.18) | 1.98 (1.66, 2.39) |
| Libya | 0.44 (0.35, 0.56) | 0.81 (0.67, 0.98) | 0.24 (0.11, 0.51) | 0.36 (0.18, 0.76) | 0.54 (0.27, 1.05) | 2.39 (1.58, 3.86) |
| St. Lucia | 0.77 (0.54, 1.11) | 0.81 (0.59, 1.12) | 0.54 (0.27, 1) | 0.6 (0.33, 1.09) | 0.01 (0.01, 0.03) | 2.73 (1.74, 4.35) |
| Sri Lanka | 0.8 (0.71, 0.91) | 1.61 (1.46, 1.78) | 0.36 (0.16, 0.84) | 0.75 (0.62, 0.9) | 0.27 (0.1, 0.72) | 3.79 (3.04, 5.14) |
| Lesotho | 0.66 (0.57, 0.76) | 1.72 (1.53, 1.94) | 0.14 (0.12, 0.17) | 0.16 (0.13, 0.19) | 0.06 (0.03, 0.12) | 2.74 (2.38, 3.19) |
| Lithuania | 0.52 (0.45, 0.61) | 0.59 (0.52, 0.66) | 0.3 (0.15, 0.6) | 0.22 (0.12, 0.44) | 0.53 (0.36, 0.77) | 2.17 (1.61, 3.08) |
| Luxembourg | 0.58 (0.44, 0.75) | 0.51 (0.41, 0.64) | 0.1 (0.05, 0.24) | 0.07 (0.02, 0.22) | 0 (0, 0.01) | 1.26 (0.92, 1.87) |
| Latvia | 0.44 (0.39, 0.5) | 1.04 (0.94, 1.15) | 0.35 (0.18, 0.66) | 0.09 (0.04, 0.2) | 0.38 (0.26, 0.55) | 2.3 (1.82, 3.07) |
| Morocco | 0.8 (0.66, 0.98) | 1.18 (1.01, 1.37) | 0.26 (0.12, 0.6) | 0.37 (0.18, 0.81) | 0.43 (0.21, 0.85) | 3.04 (2.19, 4.6) |
| Moldova | 0.75 (0.43, 1.31) | 0.42 (0.23, 0.77) | 0.32 (0.16, 0.62) | 1 (0.56, 1.67) | 0.84 (0.48, 1.4) | 3.32 (1.86, 5.78) |
| Madagascar | 0.87 (0.78, 0.97) | 1.53 (1.39, 1.69) | 0.21 (0.18, 0.24) | 0.08 (0.07, 0.1) | 0.03 (0.01, 0.06) | 2.72 (2.43, 3.05) |
| Maldives | 2.27 (1.92, 2.66) | 1.35 (1.16, 1.58) | 0.32 (0.26, 0.4) | 0.24 (0.19, 0.29) | 0.42 (0.15, 1.04) | 4.6 (3.69, 5.97) |
| Mexico | 1.51 (1.38, 1.64) | 1.73 (1.61, 1.85) | 1.47 (1.34, 1.61) | 0.43 (0.38, 0.48) | 0.05 (0.04, 0.06) | 5.18 (4.75, 5.65) |
| Marshall Islands | 1.17 (0.96, 1.43) | 1.62 (1.27, 2.1) | 0.14 (0.07, 0.31) | 0.39 (0.21, 0.75) | 0.1 (0.05, 0.2) | 3.42 (2.56, 4.79) |
| North Macedonia | 0.71 (0.58, 0.85) | 0.71 (0.61, 0.84) | 0.22 (0.11, 0.43) | 0.19 (0.1, 0.36) | 0.63 (0.34, 1.1) | 2.46 (1.74, 3.58) |
| Mali | 0.68 (0.61, 0.77) | 0.85 (0.76, 0.94) | 0.19 (0.16, 0.23) | 0.16 (0.13, 0.19) | 0.44 (0.15, 1.27) | 2.33 (1.82, 3.39) |
| Malta | 0.87 (0.75, 1.03) | 0.47 (0.41, 0.54) | 0.09 (0.04, 0.21) | 0.02 (0.01, 0.05) | 0.17 (0.08, 0.34) | 1.63 (1.3, 2.17) |
| Myanmar (Burma) | 0.83 (0.72, 0.96) | 1.52 (1.35, 1.69) | 0.2 (0.1, 0.43) | 0.25 (0.13, 0.49) | 0.13 (0.07, 0.27) | 2.93 (2.37, 3.85) |
| Montenegro | 1.91 (1.14, 3.24) | 0.65 (0.38, 1.16) | 0.27 (0.14, 0.55) | 0.28 (0.11, 0.72) | 0.6 (0.24, 1.43) | 3.7 (2, 7.1) |
| Mongolia | 0.38 (0.32, 0.46) | 0.87 (0.75, 1) | 0.11 (0.06, 0.23) | 0.25 (0.13, 0.49) | 0.1 (0.05, 0.18) | 1.71 (1.32, 2.36) |
| Mozambique | 0.99 (0.87, 1.14) | 1.28 (1.15, 1.44) | 0.28 (0.23, 0.34) | 0.33 (0.27, 0.39) | 0.36 (0.24, 0.54) | 3.24 (2.76, 3.84) |
| Mauritania | 0.33 (0.28, 0.4) | 2.03 (1.78, 2.33) | 0.12 (0.06, 0.24) | 0.2 (0.12, 0.35) | 0.06 (0.03, 0.14) | 2.74 (2.27, 3.45) |
| Mauritius | 0.74 (0.65, 0.85) | 1.63 (1.46, 1.82) | 0.21 (0.12, 0.41) | 0.39 (0.27, 0.58) | 0.3 (0.16, 0.6) | 3.28 (2.64, 4.26) |
| Malawi | 1.1 (1, 1.21) | 1.61 (1.5, 1.74) | 0.21 (0.18, 0.24) | 0.26 (0.22, 0.3) | 0.26 (0.14, 0.57) | 3.45 (3.04, 4.07) |
| Malaysia | 1.12 (1.01, 1.25) | 1.41 (1.29, 1.54) | 0.1 (0.08, 0.13) | 0.25 (0.21, 0.3) | 1.66 (1.25, 2.18) | 4.54 (3.84, 5.39) |
| Namibia | 2.43 (2.22, 2.66) | 1.37 (1.26, 1.48) | 0.18 (0.15, 0.21) | 0.13 (0.11, 0.16) | 0.28 (0.14, 0.55) | 4.39 (3.89, 5.06) |
| Niger | 0.62 (0.54, 0.72) | 0.92 (0.82, 1.05) | 1.19 (0.99, 1.43) | 0.3 (0.25, 0.36) | 0.55 (0.27, 1.16) | 3.6 (2.87, 4.72) |
| Nigeria | 0.34 (0.3, 0.38) | 0.86 (0.78, 0.94) | 0.14 (0.12, 0.16) | 0.21 (0.18, 0.24) | 0.37 (0.19, 0.74) | 1.91 (1.57, 2.46) |
| Nicaragua | 1.57 (1.3, 1.91) | 2.45 (2.04, 2.98) | 0.59 (0.32, 1.08) | 0.29 (0.16, 0.55) | 0.15 (0.08, 0.29) | 5.06 (3.9, 6.81) |
| Netherlands | 0.7 (0.65, 0.76) | 0.63 (0.59, 0.68) | 0.01 (0.01, 0.01) | 0.04 (0.03, 0.04) | 0.11 (0.1, 0.13) | 1.5 (1.38, 1.63) |
| Norway | 0.55 (0.49, 0.63) | 0.72 (0.64, 0.79) | 0.16 (0.08, 0.39) | 0.01 (0, 0.02) | 0.09 (0.08, 0.11) | 1.53 (1.29, 1.94) |
| Nepal | 0.38 (0.34, 0.44) | 1.66 (1.52, 1.81) | 0.24 (0.21, 0.29) | 0.55 (0.48, 0.63) | 0.15 (0.05, 0.41) | 2.99 (2.6, 3.58) |
| New Zealand | 1.04 (0.86, 1.26) | 0.71 (0.61, 0.83) | 0.07 (0.03, 0.17) | 0.41 (0.32, 0.52) | 0.05 (0.04, 0.07) | 2.28 (1.86, 2.84) |
| Oman | 0.98 (0.73, 1.32) | 1.03 (0.81, 1.32) | 0.13 (0.06, 0.31) | 0.39 (0.19, 0.83) | 0.45 (0.22, 0.87) | 2.98 (2.01, 4.65) |
| Pakistan | 0.34 (0.31, 0.39) | 0.74 (0.67, 0.82) | 0.15 (0.12, 0.18) | 0.1 (0.08, 0.12) | 0.09 (0.03, 0.26) | 1.43 (1.21, 1.76) |
| Panama | 1.31 (1.09, 1.58) | 1.34 (1.12, 1.63) | 0.3 (0.16, 0.59) | 0.66 (0.38, 1.14) | 0.42 (0.21, 0.79) | 4.04 (2.96, 5.73) |
| Peru | 2.29 (2.09, 2.5) | 0.85 (0.77, 0.92) | 0.63 (0.56, 0.72) | 0.35 (0.31, 0.4) | 0.19 (0.1, 0.39) | 4.31 (3.84, 4.92) |
| Philippines | 0.89 (0.82, 0.96) | 0.97 (0.9, 1.04) | 0.19 (0.16, 0.21) | 0.09 (0.08, 0.1) | 0.01 (0.01, 0.01) | 2.15 (1.98, 2.33) |
| Papua New Guinea | 0.04 (0.03, 0.06) | 0.73 (0.6, 0.89) | 0.79 (0.36, 1.55) | 0.05 (0.02, 0.1) | 0 (0, 0.01) | 1.61 (1.01, 2.61) |
| Poland | 1.79 (1.65, 1.94) | 0.82 (0.77, 0.88) | 0.01 (0.01, 0.01) | 0.03 (0.02, 0.03) | 0.2 (0.16, 0.25) | 2.84 (2.61, 3.12) |
| Portugal | 2.8 (2.56, 3.07) | 0.85 (0.79, 0.91) | 0.07 (0.07, 0.08) | 0.08 (0.07, 0.09) | 0.08 (0.07, 0.09) | 3.88 (3.56, 4.24) |
| Paraguay | 0.94 (0.81, 1.11) | 0.61 (0.54, 0.69) | 0.34 (0.18, 0.64) | 0.17 (0.09, 0.31) | 0.16 (0.08, 0.31) | 2.23 (1.71, 3.07) |
| Palestinian Territories | 0.93 (0.65, 1.33) | 1.07 (0.78, 1.44) | 0.27 (0.13, 0.61) | 0.37 (0.18, 0.77) | 0.56 (0.29, 1.07) | 3.21 (2.02, 5.23) |
| Qatar | 1.02 (0.7, 1.48) | 1.15 (0.84, 1.56) | 0.23 (0.1, 0.55) | 0.35 (0.17, 0.79) | 0.52 (0.25, 1.06) | 3.27 (2.05, 5.45) |
| Romania | 1.49 (1.27, 1.75) | 1.66 (1.47, 1.87) | 0.1 (0.07, 0.14) | 0.3 (0.22, 0.39) | 0.61 (0.41, 0.89) | 4.15 (3.44, 5.05) |
| Russia | 0.68 (0.61, 0.75) | 0.81 (0.75, 0.89) | 0.2 (0.1, 0.43) | 0.01 (0.01, 0.02) | 0.88 (0.64, 1.23) | 2.58 (2.1, 3.31) |
| Rwanda | 1.18 (1.03, 1.34) | 2.57 (2.32, 2.85) | 0.38 (0.32, 0.44) | 0.84 (0.72, 0.97) | 0.23 (0.11, 0.53) | 5.19 (4.51, 6.13) |
| Saudi Arabia | 1.23 (0.99, 1.52) | 1.03 (0.82, 1.35) | 0.21 (0.1, 0.49) | 0.54 (0.26, 1.1) | 0.69 (0.35, 1.3) | 3.7 (2.52, 5.76) |
| Sudan | 0.8 (0.55, 1.16) | 1.2 (0.86, 1.68) | 0.12 (0.07, 0.23) | 0.24 (0.14, 0.42) | 0.49 (0.26, 0.94) | 2.84 (1.88, 4.42) |
| Senegal | 0.51 (0.45, 0.56) | 0.67 (0.61, 0.74) | 0.22 (0.19, 0.25) | 0.19 (0.16, 0.22) | 0.25 (0.13, 0.53) | 1.83 (1.54, 2.3) |
| Singapore | 1.39 (1.16, 1.68) | 1.27 (1.07, 1.49) | 0.16 (0.07, 0.37) | 0.15 (0.12, 0.19) | 0.07 (0.05, 0.09) | 3.04 (2.47, 3.82) |
| Solomon Islands | 0.63 (0.5, 0.79) | 1.55 (1.2, 1.99) | 0.17 (0.08, 0.38) | 0.29 (0.15, 0.55) | 0.03 (0.02, 0.07) | 2.68 (1.95, 3.78) |
| Sierra Leone | 0.86 (0.76, 0.98) | 1.09 (0.98, 1.22) | 0.1 (0.08, 0.12) | 0.23 (0.2, 0.27) | 0.08 (0.04, 0.18) | 2.37 (2.06, 2.78) |
| El Salvador | 1.24 (0.86, 1.83) | 1.14 (0.82, 1.61) | 0.5 (0.26, 0.95) | 0.36 (0.2, 0.71) | 0.08 (0.04, 0.17) | 3.34 (2.18, 5.27) |
| Serbia | 1.15 (0.76, 1.74) | 2.79 (1.87, 4.07) | 0.55 (0.29, 1) | 0.29 (0.15, 0.57) | 0.22 (0.1, 0.53) | 5 (3.16, 7.92) |
| South Sudan | 0.64 (0.53, 0.77) | 1.33 (1.06, 1.71) | 0.19 (0.11, 0.35) | 0.3 (0.18, 0.54) | 0.26 (0.14, 0.55) | 2.71 (2.01, 3.93) |
| São Tomé and Príncipe | 1.25 (1.04, 1.51) | 0.33 (0.28, 0.4) | 0.36 (0.3, 0.44) | 0.09 (0.07, 0.12) | 0.3 (0.15, 0.66) | 2.34 (1.85, 3.13) |
| Suriname | 1.04 (0.72, 1.5) | 1.61 (1.18, 2.22) | 0.34 (0.18, 0.65) | 0.74 (0.43, 1.29) | 0.4 (0.22, 0.76) | 4.14 (2.72, 6.42) |
| Slovakia | 0.61 (0.54, 0.69) | 0.46 (0.42, 0.51) | 0.02 (0.01, 0.04) | 0.04 (0.03, 0.06) | 0.18 (0.14, 0.24) | 1.32 (1.14, 1.54) |
| Slovenia | 0.58 (0.5, 0.68) | 0.52 (0.46, 0.58) | 0.73 (0.37, 1.26) | 0.17 (0.07, 0.37) | 0.51 (0.25, 1.06) | 2.5 (1.65, 3.94) |
| Sweden | 0.76 (0.7, 0.84) | 0.8 (0.75, 0.87) | 0.09 (0.08, 0.1) | 0.1 (0.09, 0.12) | 0.13 (0.12, 0.15) | 1.89 (1.72, 2.08) |
| Eswatini | 0.89 (0.8, 1) | 1.53 (1.32, 1.76) | 0.17 (0.14, 0.21) | 0.11 (0.1, 0.14) | 0.24 (0.17, 0.34) | 2.95 (2.52, 3.45) |
| Seychelles | 1.02 (0.87, 1.17) | 1.87 (1.65, 2.12) | 0.06 (0.04, 0.07) | 0.2 (0.16, 0.24) | 0.31 (0.15, 0.62) | 3.45 (2.88, 4.23) |
| Syria | 0.87 (0.66, 1.17) | 0.96 (0.76, 1.22) | 0.26 (0.13, 0.58) | 0.37 (0.19, 0.78) | 0.56 (0.28, 1.08) | 3.03 (2.01, 4.82) |
| Chad | 0.41 (0.36, 0.47) | 0.34 (0.31, 0.38) | 0.18 (0.15, 0.21) | 0.08 (0.06, 0.1) | 0.31 (0.17, 0.64) | 1.33 (1.04, 1.8) |
| Togo | 0.53 (0.42, 0.66) | 1.36 (1.14, 1.65) | 0.14 (0.12, 0.18) | 0.14 (0.11, 0.19) | 0.31 (0.16, 0.6) | 2.49 (1.95, 3.28) |
| Thailand | 1 (0.75, 1.33) | 1.47 (1.15, 1.91) | 0.13 (0.06, 0.27) | 0.4 (0.16, 0.98) | 0.04 (0.02, 0.07) | 3.04 (2.15, 4.56) |
| Tajikistan | 0.74 (0.59, 0.94) | 0.3 (0.24, 0.39) | 0.37 (0.29, 0.46) | 0.53 (0.39, 0.74) | 0.22 (0.12, 0.43) | 2.16 (1.62, 2.95) |
| Turkmenistan | 0.55 (0.45, 0.66) | 0.56 (0.45, 0.69) | 0.19 (0.1, 0.39) | 0.29 (0.16, 0.59) | 0.04 (0.02, 0.07) | 1.62 (1.17, 2.4) |
| Timor-Leste | 0.55 (0.47, 0.64) | 1.27 (1.14, 1.43) | 0.18 (0.15, 0.22) | 0.09 (0.07, 0.1) | 0.05 (0.03, 0.09) | 2.13 (1.85, 2.48) |
| Tonga | 1.36 (1.09, 1.71) | 1.24 (1.04, 1.47) | 0.13 (0.06, 0.3) | 0.38 (0.2, 0.78) | 0.1 (0.05, 0.2) | 3.2 (2.44, 4.47) |
| Trinidad & Tobago | 0.68 (0.59, 0.79) | 0.75 (0.66, 0.85) | 0.23 (0.17, 0.3) | 0.4 (0.32, 0.48) | 0.24 (0.11, 0.52) | 2.29 (1.86, 2.94) |
| Tunisia | 0.52 (0.45, 0.6) | 1.35 (1.19, 1.52) | 0.3 (0.14, 0.66) | 0.15 (0.09, 0.24) | 0.18 (0.13, 0.25) | 2.49 (2, 3.27) |
| Turkey | 0.67 (0.58, 0.76) | 0.8 (0.71, 0.89) | 0.27 (0.1, 0.7) | 0.31 (0.21, 0.47) | 0.32 (0.2, 0.51) | 2.36 (1.8, 3.33) |
| Taiwan | 1.43 (1.16, 1.76) | 2.34 (2, 2.76) | 0.16 (0.08, 0.36) | 0.06 (0.05, 0.08) | 0.07 (0.06, 0.09) | 4.06 (3.34, 5.05) |
| Tanzania | 0.64 (0.57, 0.72) | 1.56 (1.43, 1.72) | 0.16 (0.13, 0.18) | 0.36 (0.31, 0.41) | 0.56 (0.3, 1.05) | 3.27 (2.73, 4.08) |
| Uganda | 0.54 (0.48, 0.61) | 1.03 (0.94, 1.13) | 0.17 (0.14, 0.19) | 0.48 (0.42, 0.55) | 0.26 (0.14, 0.55) | 2.48 (2.12, 3.03) |
| Ukraine | 0.71 (0.61, 0.81) | 0.82 (0.74, 0.92) | 0.25 (0.12, 0.53) | 0.08 (0.04, 0.15) | 0.68 (0.37, 1.2) | 2.54 (1.89, 3.61) |
| Uruguay | 0.96 (0.83, 1.1) | 1.41 (1.26, 1.57) | 0.34 (0.17, 0.71) | 0.46 (0.24, 0.91) | 0.69 (0.35, 1.25) | 3.86 (2.86, 5.54) |
| United States | 0.59 (0.56, 0.63) | 0.71 (0.68, 0.74) | 0.05 (0.05, 0.06) | 0.13 (0.12, 0.14) | 0.3 (0.28, 0.33) | 1.79 (1.69, 1.9) |
| Uzbekistan | 0.67 (0.53, 0.84) | 1.14 (0.91, 1.41) | 0.49 (0.39, 0.63) | 0.3 (0.16, 0.59) | 0.14 (0.08, 0.28) | 2.74 (2.06, 3.74) |
| St. Vincent & Grenadines | 1.45 (0.97, 2.15) | 0.93 (0.66, 1.26) | 0.31 (0.16, 0.61) | 0.49 (0.27, 0.9) | 0.16 (0.09, 0.31) | 3.34 (2.15, 5.22) |
| Venezuela | 1.09 (0.75, 1.56) | 0.81 (0.58, 1.1) | 0.43 (0.21, 0.85) | 0.41 (0.22, 0.81) | 0.02 (0.01, 0.04) | 2.76 (1.78, 4.36) |
| Vietnam | 0.86 (0.75, 0.98) | 1.49 (1.35, 1.66) | 0.4 (0.26, 0.61) | 0.95 (0.63, 1.42) | 0.59 (0.31, 1.1) | 4.28 (3.29, 5.78) |
| Vanuatu | 0.43 (0.34, 0.54) | 0.64 (0.5, 0.81) | 0.15 (0.08, 0.33) | 0.66 (0.34, 1.25) | 0.12 (0.06, 0.23) | 2 (1.32, 3.17) |
| Samoa | 1.47 (1.15, 1.88) | 2.99 (2.43, 3.64) | 0.13 (0.06, 0.28) | 2.3 (1.4, 3.38) | 0.79 (0.41, 1.4) | 7.67 (5.46, 10.58) |
| Yemen | 0.35 (0.29, 0.42) | 0.46 (0.38, 0.55) | 0.26 (0.22, 0.32) | 0.29 (0.23, 0.36) | 0.1 (0.05, 0.23) | 1.45 (1.16, 1.87) |
| South Africa | 0.31 (0.28, 0.34) | 1.17 (1.07, 1.27) | 0.22 (0.12, 0.42) | 0.33 (0.25, 0.45) | 0.01 (0.01, 0.01) | 2.03 (1.72, 2.49) |
| Zambia | 0.5 (0.45, 0.55) | 1.82 (1.68, 1.96) | 0.13 (0.11, 0.15) | 0.22 (0.19, 0.26) | 0.67 (0.36, 1.21) | 3.33 (2.8, 4.12) |
| Zimbabwe | 0.81 (0.74, 0.88) | 1.3 (1.19, 1.41) | 0.2 (0.17, 0.22) | 0.2 (0.17, 0.23) | 0.11 (0.07, 0.15) | 2.61 (2.35, 2.9) |

Table S16: National consumption (servings/day) in children <1 year in 2018.

| Country | Fruits | Vegetables (non-starchy) | Other non-potato starchy vegetables | Beans and legumes | Nuts and seeds |
| --- | --- | --- | --- | --- | --- |
| Afghanistan | 0.22 (0.15-0.33) | 0.08 (0.05-0.14) | 0.08 (0.03-0.28) | 0.09 (0.03-0.29) | 0.4 (0.11-1.41) |
| Angola | 0.29 (0.22-0.39) | 0.64 (0.48-0.87) | 0.04 (0.02-0.11) | 0.06 (0.03-0.14) | 0.22 (0.09-0.57) |
| Albania | 0.7 (0.58-0.86) | 0.31 (0.25-0.39) | 0.08 (0.06-0.11) | 0.08 (0.06-0.11) | 0.13 (0.05-0.34) |
| United Arab Emirates | 0.23 (0.18-0.28) | 0.1 (0.08-0.12) | 0.07 (0.02-0.2) | 0.05 (0.02-0.14) | 0.23 (0.09-0.65) |
| Argentina | 0.37 (0.32-0.44) | 0.32 (0.27-0.37) | 0.01 (0.01-0.01) | 0.01 (0.01-0.01) | 0.01 (0.01-0.02) |
| Armenia | 0.46 (0.37-0.57) | 0.23 (0.18-0.3) | 0.11 (0.08-0.14) | 0.03 (0.02-0.04) | 0.21 (0.12-0.34) |
| Antigua & Barbuda | 0.38 (0.26-0.56) | 0.59 (0.42-0.83) | 0.07 (0.03-0.18) | 0.08 (0.04-0.22) | 0.02 (0.01-0.05) |
| Australia | 2.94 (2.34-3.63) | 0.77 (0.63-0.93) | 0.22 (0.08-0.79) | 0.2 (0.14-0.29) | 0.05 (0.04-0.06) |
| Austria | 2.25 (1.89-2.71) | 0.74 (0.65-0.86) | 0.44 (0.18-1.25) | 0.05 (0.03-0.08) | 0.03 (0.03-0.04) |
| Azerbaijan | 0.54 (0.43-0.66) | 0.19 (0.15-0.25) | 0.05 (0.04-0.06) | 0.03 (0.02-0.04) | 0.35 (0.13-0.95) |
| Burundi | 0.18 (0.15-0.23) | 0.37 (0.31-0.43) | 0.03 (0.03-0.04) | 0.07 (0.05-0.08) | 0.11 (0.04-0.37) |
| Belgium | 2.25 (1.89-2.67) | 0.72 (0.62-0.84) | 0.33 (0.11-1.06) | 0.04 (0.03-0.06) | 0.02 (0.02-0.03) |
| Benin | 0.18 (0.16-0.21) | 0.19 (0.17-0.21) | 0.02 (0.02-0.02) | 0.04 (0.03-0.05) | 0.24 (0.09-0.67) |
| Burkina Faso | 0.09 (0.07-0.1) | 0.05 (0.05-0.06) | 0.01 (0-0.01) | 0.01 (0.01-0.02) | 0.43 (0.14-1.29) |
| Bangladesh | 0.18 (0.16-0.2) | 0.18 (0.16-0.2) | 0.02 (0.02-0.03) | 0.02 (0.02-0.03) | 0.01 (0.01-0.01) |
| Bulgaria | 0.48 (0.38-0.6) | 0.91 (0.73-1.12) | 0 (0-0.01) | 0.04 (0.02-0.05) | 0.14 (0.09-0.22) |
| Bahrain | 0.25 (0.18-0.36) | 0.15 (0.1-0.22) | 0.04 (0.01-0.11) | 0.05 (0.02-0.14) | 0.15 (0.06-0.42) |
| Bahamas | 0.33 (0.22-0.49) | 0.27 (0.19-0.38) | 0.07 (0.03-0.19) | 0.03 (0.01-0.09) | 0.07 (0.03-0.19) |
| Bosnia & Herzegovina | 3 (2.35-3.8) | 1.12 (0.87-1.43) | 0.04 (0.02-0.11) | 0.03 (0.01-0.06) | 0.1 (0.04-0.26) |
| Belarus | 1.22 (0.65-2.24) | 0.2 (0.11-0.38) | 0.05 (0.02-0.13) | 0.14 (0.06-0.4) | 0.12 (0.04-0.35) |
| Belize | 0.34 (0.23-0.5) | 0.39 (0.27-0.55) | 0.06 (0.02-0.17) | 0.05 (0.02-0.14) | 0.02 (0.01-0.04) |
| Bolivia | 0.35 (0.3-0.39) | 0.29 (0.26-0.33) | 0.1 (0.09-0.12) | 0.02 (0.02-0.03) | 0.09 (0.04-0.24) |
| Brazil | 0.34 (0.29-0.38) | 0.24 (0.21-0.27) | 0.01 (0.01-0.02) | 0.17 (0.14-0.2) | 0 (0-0) |
| Barbados | 0.5 (0.41-0.62) | 0.35 (0.29-0.42) | 0.06 (0.02-0.16) | 0.01 (0.01-0.02) | 0.07 (0.04-0.11) |
| Brunei | 0.71 (0.51-1.02) | 0.79 (0.59-1.06) | 0.03 (0.01-0.09) | 0.22 (0.1-0.53) | 0.06 (0.03-0.15) |
| Bhutan | 0.32 (0.23-0.43) | 0.24 (0.17-0.33) | 0.04 (0.01-0.11) | 0.05 (0.02-0.15) | 0.07 (0.02-0.25) |
| Botswana | 0.12 (0.09-0.16) | 0.21 (0.17-0.26) | 0.03 (0.01-0.06) | 0.03 (0.01-0.06) | 0.83 (0.36-1.79) |
| Central African Republic | 0.24 (0.18-0.32) | 0.2 (0.14-0.29) | 0.08 (0.07-0.1) | 0.06 (0.03-0.14) | 0.15 (0.06-0.4) |
| Canada | 2.96 (2.52-3.47) | 0.84 (0.74-0.96) | 0.25 (0.2-0.31) | 0.16 (0.13-0.2) | 0.11 (0.09-0.13) |
| Switzerland | 2.96 (2.43-3.59) | 0.72 (0.61-0.86) | 0.6 (0.25-1.78) | 0.02 (0.02-0.03) | 0.04 (0.02-0.09) |
| Chile | 0.43 (0.34-0.53) | 0.35 (0.29-0.43) | 0.12 (0.05-0.36) | 0.03 (0.02-0.05) | 0.13 (0.05-0.37) |
| China | 0.75 (0.66-0.86) | 1.44 (1.29-1.63) | 0.04 (0.02-0.1) | 0.1 (0.08-0.13) | 0.13 (0.1-0.17) |
| Côte d’lvoire | 0.15 (0.12-0.17) | 0.18 (0.15-0.21) | 0.03 (0.02-0.03) | 0.01 (0.01-0.01) | 0.17 (0.07-0.49) |
| Cameroon | 0.2 (0.18-0.23) | 0.24 (0.22-0.27) | 0.03 (0.03-0.04) | 0.05 (0.04-0.06) | 0.28 (0.12-0.78) |
| Congo - Kinshasa | 0.23 (0.21-0.27) | 0.42 (0.38-0.47) | 0.04 (0.03-0.05) | 0.05 (0.04-0.06) | 0.51 (0.21-1.28) |
| Congo - Brazzaville | 0.22 (0.19-0.25) | 0.27 (0.25-0.3) | 0.04 (0.03-0.04) | 0.04 (0.03-0.04) | 0.18 (0.07-0.49) |
| Colombia | 0.4 (0.36-0.44) | 0.24 (0.22-0.27) | 0.14 (0.12-0.15) | 0.05 (0.05-0.06) | 0.4 (0.16-0.99) |
| Comoros | 0.21 (0.18-0.24) | 0.18 (0.16-0.21) | 0.04 (0.03-0.04) | 0.02 (0.01-0.02) | 0.14 (0.06-0.39) |
| Cape Verde | 0.55 (0.39-0.76) | 0.35 (0.27-0.46) | 0.02 (0.01-0.05) | 0.04 (0.02-0.11) | 0.27 (0.1-0.77) |
| Costa Rica | 0.52 (0.36-0.75) | 0.42 (0.3-0.58) | 0.07 (0.03-0.18) | 0.05 (0.02-0.13) | 0.06 (0.03-0.16) |
| Cuba | 0.5 (0.37-0.69) | 0.28 (0.21-0.39) | 0.08 (0.03-0.22) | 0.05 (0.02-0.13) | 0 (0-0.01) |
| Cyprus | 1.32 (0.81-2.21) | 0.77 (0.47-1.3) | 0.2 (0.08-0.59) | 0.26 (0.1-0.67) | 0.03 (0.01-0.07) |
| Czechia | 0.37 (0.3-0.45) | 0.24 (0.19-0.3) | 0.04 (0.02-0.12) | 0.02 (0.01-0.06) | 0.04 (0.02-0.07) |
| Germany | 2.97 (2.54-3.47) | 1.2 (1.05-1.37) | 0.47 (0.38-0.58) | 0.04 (0.03-0.05) | 0.02 (0.02-0.02) |
| Djibouti | 0.34 (0.25-0.47) | 0.19 (0.14-0.28) | 0.02 (0.01-0.06) | 0.33 (0.15-0.83) | 0 (0-0) |
| Dominica | 0.42 (0.33-0.54) | 0.26 (0.21-0.33) | 0.08 (0.03-0.21) | 0.06 (0.03-0.14) | 0.03 (0.01-0.09) |
| Denmark | 1.95 (1.57-2.41) | 0.78 (0.65-0.93) | 0.39 (0.14-1.29) | 0.18 (0.08-0.48) | 0.05 (0.04-0.07) |
| Dominican Republic | 0.57 (0.51-0.64) | 0.27 (0.24-0.3) | 0.08 (0.07-0.09) | 0.09 (0.08-0.11) | 0.01 (0-0.03) |
| Algeria | 0.17 (0.13-0.23) | 0.16 (0.13-0.21) | 0.05 (0.02-0.13) | 0.03 (0.01-0.08) | 0.04 (0.02-0.12) |
| Ecuador | 0.1 (0.09-0.12) | 0.28 (0.24-0.33) | 0.04 (0.02-0.09) | 0.03 (0.02-0.06) | 0.02 (0.01-0.05) |
| Egypt | 0.19 (0.17-0.22) | 0.13 (0.11-0.14) | 0.03 (0.03-0.04) | 0.01 (0.01-0.01) | 0.02 (0.01-0.02) |
| Eritrea | 0.14 (0.1-0.19) | 0.13 (0.1-0.17) | 0.02 (0.01-0.06) | 0.04 (0.02-0.1) | 0.19 (0.08-0.51) |
| Spain | 1.37 (1.15-1.62) | 0.64 (0.55-0.73) | 0.19 (0.07-0.55) | 0.17 (0.08-0.38) | 0.05 (0.04-0.07) |
| Estonia | 0.52 (0.43-0.64) | 0.67 (0.54-0.84) | 0 (0-0.01) | 0.01 (0.01-0.02) | 0.1 (0.05-0.17) |
| Ethiopia | 0.09 (0.08-0.1) | 0.17 (0.15-0.19) | 0.02 (0.02-0.02) | 0.05 (0.05-0.06) | 0.03 (0.02-0.05) |
| Finland | 3.22 (2.71-3.8) | 0.8 (0.69-0.91) | 0.13 (0.05-0.32) | 0.05 (0.04-0.07) | 0.02 (0.01-0.02) |
| Fiji | 0.94 (0.63-1.39) | 1.05 (0.75-1.44) | 0.05 (0.02-0.13) | 0.19 (0.08-0.45) | 0.06 (0.03-0.16) |
| France | 3.28 (2.8-3.85) | 1 (0.88-1.14) | 0.02 (0.01-0.02) | 0.11 (0.09-0.15) | 0.02 (0.01-0.02) |
| Micronesia (Federated States of) | 1.24 (0.89-1.75) | 0.69 (0.47-1.03) | 0.05 (0.02-0.14) | 0.18 (0.08-0.45) | 0.04 (0.02-0.11) |
| Gabon | 0.17 (0.14-0.2) | 0.23 (0.2-0.27) | 0.04 (0.04-0.05) | 0 (0-0.01) | 0.37 (0.14-1.06) |
| United Kingdom | 1.99 (1.69-2.34) | 0.76 (0.66-0.87) | 0.27 (0.1-0.84) | 0.34 (0.26-0.44) | 0.03 (0.03-0.04) |
| Georgia | 0.36 (0.29-0.46) | 0.21 (0.16-0.27) | 0.03 (0.01-0.07) | 0.06 (0.03-0.17) | 0.07 (0.02-0.19) |
| Ghana | 0.21 (0.19-0.23) | 0.26 (0.24-0.28) | 0.03 (0.02-0.03) | 0.03 (0.03-0.04) | 0.56 (0.3-1.08) |
| Guinea | 0.05 (0.04-0.07) | 0.04 (0.03-0.05) | 0.01 (0.01-0.02) | 0.01 (0-0.01) | 0.2 (0.08-0.54) |
| Gambia | 0.08 (0.06-0.1) | 0.09 (0.07-0.12) | 0.01 (0.01-0.02) | 0.01 (0.01-0.01) | 0.22 (0.08-0.66) |
| Guinea-Bissau | 0.16 (0.12-0.22) | 0.26 (0.19-0.37) | 0.03 (0.01-0.06) | 0.14 (0.06-0.34) | 0.24 (0.1-0.62) |
| Equatorial Guinea | 0.22 (0.16-0.29) | 0.28 (0.2-0.4) | 0.04 (0.02-0.1) | 0.06 (0.03-0.14) | 0.21 (0.08-0.56) |
| Greece | 1.63 (1.34-1.97) | 0.56 (0.48-0.67) | 0.22 (0.09-0.63) | 0.11 (0.08-0.16) | 0.05 (0.03-0.06) |
| Grenada | 0.48 (0.33-0.7) | 0.64 (0.46-0.9) | 0.08 (0.03-0.19) | 0.06 (0.03-0.14) | 0 (0-0) |
| Guatemala | 0.3 (0.27-0.35) | 0.25 (0.22-0.28) | 0.05 (0.05-0.06) | 0.1 (0.08-0.11) | 0.02 (0.01-0.05) |
| Guyana | 0.81 (0.68-0.97) | 0.38 (0.32-0.44) | 0.07 (0.06-0.08) | 0.04 (0.04-0.06) | 0.06 (0.03-0.16) |
| Honduras | 0.36 (0.31-0.41) | 0.08 (0.07-0.09) | 0.05 (0.04-0.06) | 0.08 (0.07-0.1) | 0.04 (0.02-0.05) |
| Croatia | 0.3 (0.24-0.37) | 1.69 (1.35-2.14) | 0.05 (0.02-0.12) | 0.02 (0.01-0.04) | 0.11 (0.04-0.27) |
| Haiti | 0.28 (0.25-0.32) | 0.21 (0.19-0.24) | 0.03 (0.03-0.04) | 0.06 (0.05-0.07) | 0.04 (0.03-0.06) |
| Hungary | 0.3 (0.24-0.37) | 0.14 (0.11-0.18) | 0.04 (0.02-0.1) | 0.03 (0.01-0.1) | 0.02 (0.01-0.04) |
| Indonesia | 0.58 (0.52-0.64) | 0.79 (0.72-0.86) | 0.08 (0.07-0.09) | 0.19 (0.16-0.22) | 0.08 (0.06-0.1) |
| India | 0.12 (0.1-0.13) | 0.13 (0.12-0.15) | 0.03 (0.02-0.03) | 0.02 (0.02-0.02) | 0.04 (0.03-0.05) |
| Ireland | 1.76 (1.4-2.21) | 0.77 (0.64-0.92) | 0.33 (0.13-0.92) | 0.19 (0.08-0.43) | 0.08 (0.04-0.17) |
| Iran | 0.35 (0.3-0.41) | 0.18 (0.15-0.21) | 0.01 (0.01-0.01) | 0.02 (0.02-0.03) | 0.06 (0.04-0.07) |
| Iraq | 0.23 (0.16-0.34) | 0.11 (0.07-0.15) | 0.04 (0.02-0.11) | 0.05 (0.02-0.13) | 0.1 (0.04-0.27) |
| Iceland | 1.71 (1.42-2.04) | 0.46 (0.39-0.53) | 1.74 (0.62-5.06) | 0.05 (0.03-0.06) | 0.01 (0.01-0.01) |
| Israel | 0.28 (0.23-0.34) | 0.07 (0.06-0.08) | 0.06 (0.05-0.08) | 0.1 (0.07-0.13) | 0.23 (0.17-0.31) |
| Italy | 4.18 (3.6-4.89) | 0.92 (0.8-1.06) | 0.3 (0.23-0.38) | 0.11 (0.09-0.14) | 0.02 (0.02-0.03) |
| Jamaica | 0.7 (0.54-0.91) | 0.38 (0.31-0.47) | 0.06 (0.02-0.14) | 0.02 (0.02-0.03) | 0.02 (0.02-0.04) |
| Jordan | 0.37 (0.33-0.43) | 0.1 (0.09-0.12) | 0.03 (0.02-0.03) | 0.04 (0.03-0.04) | 0.09 (0.06-0.12) |
| Japan | 0.86 (0.78-0.96) | 1.3 (1.19-1.41) | 0.02 (0.01-0.03) | 0.35 (0.29-0.44) | 0.03 (0.03-0.04) |
| Kazakhstan | 0.31 (0.26-0.38) | 0.41 (0.33-0.51) | 0.06 (0.05-0.08) | 0.01 (0.01-0.03) | 1.31 (0.53-2.63) |
| Kenya | 0.25 (0.23-0.28) | 0.47 (0.43-0.51) | 0.05 (0.04-0.06) | 0.04 (0.04-0.05) | 0.01 (0.01-0.02) |
| Kyrgyzstan | 0.4 (0.34-0.48) | 0.11 (0.08-0.14) | 0.06 (0.05-0.07) | 0.01 (0.01-0.02) | 0.4 (0.15-1.09) |
| Cambodia | 0.42 (0.37-0.48) | 0.59 (0.53-0.66) | 0.05 (0.04-0.06) | 0.03 (0.02-0.03) | 0.02 (0.02-0.04) |
| Kiribati | 0.84 (0.56-1.27) | 0.45 (0.31-0.65) | 0.05 (0.02-0.14) | 0.19 (0.08-0.47) | 0.05 (0.02-0.12) |
| South Korea | 0.8 (0.71-0.89) | 0.64 (0.58-0.7) | 0.01 (0.01-0.02) | 0.18 (0.15-0.21) | 0.02 (0.02-0.03) |
| Kuwait | 0.12 (0.1-0.15) | 0.09 (0.07-0.11) | 0.02 (0.01-0.07) | 0.06 (0.02-0.17) | 0.18 (0.06-0.53) |
| Laos | 1.03 (0.88-1.2) | 0.71 (0.62-0.8) | 0.02 (0.01-0.03) | 0.16 (0.07-0.43) | 0.01 (0.01-0.01) |
| Lebanon | 0.48 (0.39-0.58) | 0.28 (0.24-0.34) | 0.02 (0.02-0.03) | 0.04 (0.03-0.05) | 0.34 (0.25-0.48) |
| Liberia | 0.12 (0.1-0.14) | 0.23 (0.2-0.27) | 0.03 (0.02-0.03) | 0.02 (0.01-0.02) | 0.05 (0.02-0.13) |
| Libya | 0.11 (0.08-0.15) | 0.09 (0.07-0.12) | 0.03 (0.01-0.1) | 0.05 (0.02-0.13) | 0.15 (0.06-0.4) |
| St. Lucia | 0.3 (0.21-0.44) | 0.23 (0.16-0.32) | 0.1 (0.04-0.28) | 0.11 (0.05-0.27) | 0 (0-0.01) |
| Sri Lanka | 0.26 (0.22-0.31) | 0.16 (0.14-0.18) | 0.08 (0.03-0.24) | 0.07 (0.05-0.08) | 0.1 (0.03-0.36) |
| Lesotho | 0.16 (0.14-0.19) | 0.35 (0.31-0.4) | 0.02 (0.02-0.02) | 0.02 (0.02-0.03) | 0.03 (0.01-0.08) |
| Lithuania | 0.34 (0.27-0.43) | 0.3 (0.24-0.38) | 0.05 (0.02-0.12) | 0.05 (0.02-0.12) | 0.11 (0.06-0.21) |
| Luxembourg | 1.89 (1.41-2.52) | 0.61 (0.47-0.79) | 0.41 (0.15-1.31) | 0.08 (0.02-0.28) | 0 (0-0) |
| Latvia | 0.29 (0.24-0.36) | 0.53 (0.42-0.67) | 0.05 (0.02-0.14) | 0.02 (0.01-0.05) | 0.08 (0.04-0.15) |
| Morocco | 0.2 (0.16-0.26) | 0.14 (0.11-0.17) | 0.04 (0.01-0.12) | 0.05 (0.02-0.14) | 0.11 (0.05-0.32) |
| Moldova | 0.49 (0.28-0.87) | 0.21 (0.11-0.39) | 0.05 (0.02-0.13) | 0.22 (0.09-0.54) | 0.18 (0.07-0.49) |
| Madagascar | 0.22 (0.19-0.24) | 0.32 (0.29-0.35) | 0.03 (0.03-0.04) | 0.01 (0.01-0.02) | 0.02 (0.01-0.04) |
| Maldives | 0.75 (0.62-0.91) | 0.14 (0.11-0.16) | 0.08 (0.06-0.09) | 0.02 (0.02-0.03) | 0.16 (0.05-0.54) |
| Mexico | 0.6 (0.53-0.67) | 0.5 (0.45-0.55) | 0.28 (0.25-0.32) | 0.08 (0.07-0.1) | 0.01 (0.01-0.02) |
| Marshall Islands | 0.95 (0.69-1.31) | 0.91 (0.66-1.3) | 0.04 (0.02-0.12) | 0.26 (0.11-0.65) | 0.05 (0.02-0.13) |
| North Macedonia | 0.46 (0.35-0.59) | 0.35 (0.27-0.46) | 0.03 (0.01-0.09) | 0.04 (0.02-0.1) | 0.13 (0.05-0.34) |
| Mali | 0.17 (0.15-0.2) | 0.18 (0.16-0.2) | 0.03 (0.02-0.03) | 0.02 (0.02-0.03) | 0.25 (0.08-0.79) |
| Malta | 2.85 (2.31-3.51) | 0.57 (0.48-0.68) | 0.36 (0.14-1.18) | 0.03 (0.02-0.06) | 0.04 (0.02-0.08) |
| Myanmar (Burma) | 0.68 (0.57-0.81) | 0.85 (0.74-0.98) | 0.06 (0.02-0.17) | 0.16 (0.07-0.43) | 0.06 (0.03-0.17) |
| Montenegro | 1.24 (0.72-2.11) | 0.32 (0.18-0.57) | 0.04 (0.02-0.1) | 0.06 (0.02-0.16) | 0.13 (0.04-0.35) |
| Mongolia | 0.26 (0.2-0.33) | 0.46 (0.36-0.59) | 0.02 (0.01-0.05) | 0.06 (0.02-0.14) | 0.02 (0.01-0.06) |
| Mozambique | 0.25 (0.22-0.29) | 0.27 (0.24-0.3) | 0.04 (0.03-0.05) | 0.05 (0.04-0.06) | 0.21 (0.12-0.35) |
| Mauritania | 0.08 (0.07-0.1) | 0.43 (0.37-0.5) | 0.02 (0.01-0.04) | 0.03 (0.01-0.07) | 0.03 (0.01-0.09) |
| Mauritius | 0.17 (0.15-0.2) | 0.32 (0.28-0.36) | 0.03 (0.01-0.07) | 0.05 (0.04-0.08) | 0.16 (0.07-0.43) |
| Malawi | 0.28 (0.25-0.31) | 0.34 (0.31-0.36) | 0.03 (0.03-0.04) | 0.04 (0.03-0.04) | 0.14 (0.06-0.43) |
| Malaysia | 0.91 (0.79-1.06) | 0.79 (0.71-0.89) | 0.03 (0.02-0.04) | 0.17 (0.14-0.21) | 0.87 (0.63-1.18) |
| Namibia | 0.61 (0.54-0.68) | 0.29 (0.26-0.31) | 0.03 (0.02-0.03) | 0.02 (0.02-0.02) | 0.15 (0.07-0.39) |
| Niger | 0.16 (0.14-0.19) | 0.2 (0.18-0.23) | 0.18 (0.15-0.22) | 0.05 (0.04-0.06) | 0.3 (0.11-0.92) |
| Nigeria | 0.09 (0.08-0.1) | 0.18 (0.16-0.2) | 0.02 (0.02-0.02) | 0.03 (0.03-0.04) | 0.2 (0.08-0.57) |
| Nicaragua | 0.63 (0.47-0.83) | 0.73 (0.53-0.99) | 0.11 (0.05-0.29) | 0.06 (0.03-0.14) | 0.04 (0.02-0.11) |
| Netherlands | 2.29 (1.98-2.67) | 0.75 (0.66-0.84) | 0.05 (0.04-0.06) | 0.04 (0.04-0.06) | 0.03 (0.02-0.03) |
| Norway | 1.81 (1.51-2.17) | 0.85 (0.74-0.99) | 0.65 (0.23-2.19) | 0.01 (0-0.02) | 0.02 (0.02-0.03) |
| Nepal | 0.12 (0.11-0.14) | 0.16 (0.14-0.18) | 0.05 (0.05-0.07) | 0.05 (0.04-0.06) | 0.06 (0.02-0.2) |
| New Zealand | 3.4 (2.71-4.28) | 0.85 (0.7-1.02) | 0.28 (0.1-0.96) | 0.51 (0.38-0.68) | 0.01 (0.01-0.02) |
| Oman | 0.26 (0.19-0.36) | 0.13 (0.1-0.17) | 0.02 (0.01-0.06) | 0.05 (0.02-0.15) | 0.13 (0.05-0.34) |
| Pakistan | 0.11 (0.1-0.13) | 0.07 (0.07-0.09) | 0.03 (0.03-0.04) | 0.01 (0.01-0.01) | 0.04 (0.01-0.12) |
| Panama | 0.52 (0.39-0.69) | 0.39 (0.29-0.52) | 0.06 (0.02-0.14) | 0.13 (0.06-0.3) | 0.12 (0.05-0.3) |
| Peru | 0.91 (0.83-1) | 0.24 (0.22-0.27) | 0.12 (0.11-0.14) | 0.07 (0.06-0.08) | 0.05 (0.02-0.14) |
| Philippines | 0.73 (0.64-0.81) | 0.55 (0.5-0.61) | 0.06 (0.05-0.07) | 0.06 (0.05-0.07) | 0.01 (0-0.01) |
| Papua New Guinea | 0.03 (0.02-0.05) | 0.42 (0.34-0.51) | 0.25 (0.09-0.79) | 0.03 (0.01-0.1) | 0 (0-0) |
| Poland | 1.17 (0.97-1.4) | 0.41 (0.33-0.51) | 0 (0-0) | 0.01 (0-0.01) | 0.04 (0.02-0.07) |
| Portugal | 9.07 (7.88-10.08) | 1 (0.89-1.12) | 0.33 (0.28-0.39) | 0.1 (0.08-0.12) | 0.02 (0.01-0.02) |
| Paraguay | 0.37 (0.31-0.45) | 0.18 (0.15-0.2) | 0.06 (0.03-0.16) | 0.03 (0.02-0.07) | 0.05 (0.02-0.12) |
| Palestinian Territories | 0.24 (0.16-0.35) | 0.13 (0.09-0.17) | 0.04 (0.02-0.12) | 0.05 (0.02-0.14) | 0.16 (0.06-0.41) |
| Qatar | 0.26 (0.17-0.38) | 0.13 (0.09-0.19) | 0.03 (0.01-0.11) | 0.05 (0.02-0.15) | 0.14 (0.05-0.43) |
| Romania | 0.96 (0.76-1.21) | 0.82 (0.65-1.04) | 0.02 (0.01-0.02) | 0.06 (0.04-0.1) | 0.13 (0.07-0.24) |
| Russia | 0.45 (0.37-0.54) | 0.42 (0.34-0.52) | 0.03 (0.01-0.09) | 0 (0-0) | 0.19 (0.11-0.35) |
| Rwanda | 0.29 (0.26-0.34) | 0.54 (0.48-0.6) | 0.05 (0.05-0.06) | 0.13 (0.11-0.15) | 0.13 (0.05-0.38) |
| Saudi Arabia | 0.31 (0.22-0.43) | 0.12 (0.08-0.17) | 0.03 (0.01-0.1) | 0.07 (0.03-0.2) | 0.19 (0.07-0.51) |
| Sudan | 0.2 (0.14-0.29) | 0.25 (0.18-0.35) | 0.02 (0.01-0.04) | 0.03 (0.02-0.08) | 0.27 (0.11-0.75) |
| Senegal | 0.13 (0.11-0.14) | 0.14 (0.13-0.16) | 0.03 (0.03-0.04) | 0.03 (0.03-0.03) | 0.14 (0.05-0.39) |
| Singapore | 1.13 (0.92-1.4) | 0.71 (0.59-0.84) | 0.04 (0.02-0.14) | 0.1 (0.07-0.13) | 0.04 (0.03-0.05) |
| Solomon Islands | 0.52 (0.41-0.68) | 0.9 (0.7-1.18) | 0.06 (0.02-0.15) | 0.19 (0.08-0.47) | 0.02 (0.01-0.04) |
| Sierra Leone | 0.22 (0.19-0.25) | 0.23 (0.2-0.26) | 0.01 (0.01-0.02) | 0.03 (0.03-0.04) | 0.04 (0.02-0.13) |
| El Salvador | 0.49 (0.33-0.73) | 0.33 (0.24-0.46) | 0.09 (0.04-0.26) | 0.07 (0.03-0.18) | 0.02 (0.01-0.06) |
| Serbia | 0.74 (0.48-1.17) | 1.37 (0.86-2.11) | 0.08 (0.04-0.21) | 0.06 (0.03-0.13) | 0.05 (0.02-0.12) |
| South Sudan | 0.16 (0.12-0.22) | 0.28 (0.19-0.41) | 0.03 (0.01-0.07) | 0.04 (0.02-0.11) | 0.14 (0.06-0.39) |
| São Tomé and Príncipe | 0.31 (0.26-0.38) | 0.07 (0.06-0.08) | 0.05 (0.04-0.06) | 0.01 (0.01-0.02) | 0.16 (0.06-0.48) |
| Suriname | 0.41 (0.28-0.6) | 0.47 (0.33-0.65) | 0.06 (0.03-0.17) | 0.14 (0.07-0.35) | 0.12 (0.05-0.3) |
| Slovakia | 0.4 (0.33-0.49) | 0.23 (0.19-0.29) | 0 (0-0.01) | 0.01 (0.01-0.02) | 0.04 (0.02-0.07) |
| Slovenia | 0.38 (0.31-0.47) | 0.26 (0.21-0.33) | 0.11 (0.05-0.32) | 0.04 (0.02-0.09) | 0.11 (0.05-0.26) |
| Sweden | 2.49 (2.14-2.94) | 0.97 (0.85-1.1) | 0.39 (0.32-0.48) | 0.13 (0.1-0.16) | 0.03 (0.03-0.04) |
| Eswatini | 0.22 (0.19-0.25) | 0.31 (0.26-0.36) | 0.02 (0.02-0.03) | 0.02 (0.01-0.02) | 0.13 (0.09-0.2) |
| Seychelles | 0.25 (0.21-0.29) | 0.39 (0.34-0.44) | 0.01 (0.01-0.01) | 0.03 (0.02-0.04) | 0.17 (0.07-0.46) |
| Syria | 0.22 (0.16-0.3) | 0.11 (0.08-0.15) | 0.04 (0.02-0.11) | 0.05 (0.02-0.13) | 0.15 (0.06-0.39) |
| Chad | 0.11 (0.09-0.12) | 0.07 (0.06-0.08) | 0.03 (0.02-0.03) | 0.01 (0.01-0.02) | 0.17 (0.07-0.48) |
| Togo | 0.13 (0.11-0.17) | 0.28 (0.24-0.34) | 0.02 (0.02-0.03) | 0.02 (0.02-0.03) | 0.17 (0.07-0.45) |
| Thailand | 0.81 (0.6-1.09) | 0.82 (0.64-1.08) | 0.04 (0.02-0.11) | 0.27 (0.11-0.67) | 0.02 (0.01-0.04) |
| Tajikistan | 0.5 (0.4-0.62) | 0.16 (0.12-0.21) | 0.06 (0.05-0.08) | 0.13 (0.09-0.17) | 0.05 (0.02-0.13) |
| Turkmenistan | 0.36 (0.27-0.5) | 0.29 (0.2-0.41) | 0.03 (0.01-0.08) | 0.06 (0.03-0.17) | 0.01 (0-0.02) |
| Timor-Leste | 0.45 (0.38-0.53) | 0.72 (0.64-0.82) | 0.06 (0.05-0.07) | 0.06 (0.05-0.08) | 0.02 (0.01-0.06) |
| Tonga | 1.12 (0.87-1.43) | 0.71 (0.59-0.85) | 0.04 (0.01-0.13) | 0.24 (0.1-0.68) | 0.05 (0.02-0.14) |
| Trinidad & Tobago | 0.27 (0.23-0.32) | 0.22 (0.18-0.25) | 0.04 (0.03-0.06) | 0.08 (0.06-0.1) | 0.07 (0.03-0.16) |
| Tunisia | 0.13 (0.11-0.16) | 0.16 (0.13-0.19) | 0.05 (0.02-0.13) | 0.02 (0.01-0.03) | 0.05 (0.03-0.08) |
| Turkey | 0.16 (0.14-0.2) | 0.09 (0.07-0.11) | 0.04 (0.01-0.11) | 0.04 (0.03-0.07) | 0.09 (0.05-0.15) |
| Taiwan | 1.16 (0.92-1.45) | 1.3 (1.09-1.56) | 0.05 (0.02-0.14) | 0.04 (0.03-0.05) | 0.04 (0.03-0.05) |
| Tanzania | 0.16 (0.14-0.18) | 0.33 (0.3-0.37) | 0.02 (0.02-0.03) | 0.05 (0.05-0.06) | 0.31 (0.12-0.84) |
| Uganda | 0.14 (0.12-0.16) | 0.22 (0.2-0.24) | 0.02 (0.02-0.03) | 0.07 (0.06-0.08) | 0.14 (0.06-0.4) |
| Ukraine | 0.46 (0.37-0.58) | 0.42 (0.33-0.53) | 0.04 (0.02-0.11) | 0.02 (0.01-0.04) | 0.14 (0.05-0.41) |
| Uruguay | 0.38 (0.32-0.44) | 0.4 (0.35-0.46) | 0.06 (0.02-0.18) | 0.08 (0.04-0.23) | 0.2 (0.08-0.58) |
| United States | 1.93 (1.7-2.21) | 0.84 (0.76-0.94) | 0.23 (0.2-0.28) | 0.17 (0.14-0.2) | 0.07 (0.06-0.09) |
| Uzbekistan | 0.44 (0.35-0.56) | 0.57 (0.41-0.83) | 0.08 (0.07-0.1) | 0.07 (0.03-0.17) | 0.03 (0.01-0.08) |
| St. Vincent & Grenadines | 0.57 (0.38-0.84) | 0.26 (0.19-0.36) | 0.06 (0.02-0.14) | 0.09 (0.04-0.21) | 0.05 (0.02-0.11) |
| Venezuela | 0.43 (0.3-0.63) | 0.23 (0.17-0.33) | 0.08 (0.03-0.23) | 0.08 (0.03-0.2) | 0.01 (0-0.02) |
| Vietnam | 0.7 (0.59-0.83) | 0.85 (0.75-0.97) | 0.13 (0.08-0.2) | 0.66 (0.43-1.03) | 0.3 (0.13-0.74) |
| Vanuatu | 0.36 (0.28-0.46) | 0.37 (0.29-0.48) | 0.05 (0.02-0.13) | 0.44 (0.19-1.18) | 0.06 (0.03-0.15) |
| Samoa | 1.22 (0.93-1.59) | 1.74 (1.4-2.14) | 0.04 (0.02-0.11) | 1.88 (0.81-4.88) | 0.42 (0.18-1.06) |
| Yemen | 0.09 (0.07-0.11) | 0.05 (0.04-0.06) | 0.04 (0.03-0.05) | 0.04 (0.03-0.05) | 0.03 (0.01-0.08) |
| South Africa | 0.08 (0.07-0.08) | 0.24 (0.22-0.26) | 0.03 (0.01-0.08) | 0.05 (0.04-0.07) | 0 (0-0.01) |
| Zambia | 0.13 (0.11-0.14) | 0.38 (0.35-0.42) | 0.02 (0.02-0.02) | 0.03 (0.03-0.04) | 0.38 (0.17-0.92) |
| Zimbabwe | 0.2 (0.18-0.22) | 0.27 (0.25-0.3) | 0.03 (0.02-0.03) | 0.03 (0.03-0.04) | 0.06 (0.04-0.09) |

Table S17: National consumption (servings/day) in children 1-2 years in 2018.

| Country | Fruit | Vegetables (non-starchy) | Other non-potato starchy vegetables | Beans and legumes | Nuts and seeds |
| --- | --- | --- | --- | --- | --- |
| Afghanistan | 0.29 (0.2-0.44) | 0.24 (0.15-0.4) | 0.21 (0.07-0.75) | 0.27 (0.09-0.92) | 0.44 (0.13-1.45) |
| Angola | 0.43 (0.33-0.58) | 1.42 (1.05-1.97) | 0.15 (0.07-0.35) | 0.16 (0.08-0.37) | 0.21 (0.09-0.58) |
| Albania | 0.7 (0.59-0.83) | 0.46 (0.38-0.56) | 0.24 (0.19-0.33) | 0.18 (0.14-0.23) | 0.19 (0.08-0.46) |
| United Arab Emirates | 0.33 (0.27-0.4) | 0.26 (0.22-0.31) | 0.2 (0.08-0.6) | 0.14 (0.05-0.4) | 0.3 (0.12-0.78) |
| Argentina | 0.46 (0.4-0.53) | 0.61 (0.53-0.7) | 0.03 (0.02-0.04) | 0.02 (0.01-0.02) | 0.02 (0.01-0.02) |
| Armenia | 0.46 (0.38-0.56) | 0.35 (0.28-0.43) | 0.33 (0.26-0.4) | 0.07 (0.05-0.1) | 0.3 (0.19-0.48) |
| Antigua & Barbuda | 0.47 (0.32-0.68) | 1.14 (0.81-1.58) | 0.2 (0.09-0.53) | 0.21 (0.09-0.51) | 0.02 (0.01-0.05) |
| Australia | 1.56 (1.28-1.89) | 0.83 (0.7-0.98) | 0.18 (0.06-0.71) | 0.23 (0.16-0.32) | 0.06 (0.05-0.08) |
| Austria | 1.2 (1.04-1.39) | 0.8 (0.71-0.9) | 0.36 (0.15-1.03) | 0.06 (0.04-0.1) | 0.05 (0.04-0.06) |
| Azerbaijan | 0.53 (0.45-0.64) | 0.29 (0.23-0.35) | 0.15 (0.12-0.18) | 0.07 (0.05-0.09) | 0.52 (0.22-1.22) |
| Burundi | 0.27 (0.22-0.33) | 0.81 (0.68-0.95) | 0.11 (0.09-0.14) | 0.18 (0.15-0.22) | 0.12 (0.05-0.36) |
| Belgium | 1.2 (1.04-1.37) | 0.78 (0.69-0.89) | 0.27 (0.09-0.95) | 0.04 (0.03-0.07) | 0.03 (0.02-0.03) |
| Benin | 0.27 (0.24-0.3) | 0.42 (0.38-0.47) | 0.06 (0.05-0.07) | 0.11 (0.09-0.13) | 0.24 (0.1-0.63) |
| Burkina Faso | 0.13 (0.11-0.14) | 0.12 (0.1-0.13) | 0.02 (0.01-0.02) | 0.04 (0.03-0.05) | 0.43 (0.14-1.24) |
| Bangladesh | 0.24 (0.22-0.26) | 0.52 (0.48-0.56) | 0.06 (0.05-0.07) | 0.08 (0.07-0.08) | 0.01 (0.01-0.01) |
| Bulgaria | 0.47 (0.39-0.57) | 1.35 (1.14-1.6) | 0.01 (0.01-0.03) | 0.08 (0.06-0.11) | 0.21 (0.15-0.29) |
| Bahrain | 0.36 (0.26-0.5) | 0.39 (0.27-0.58) | 0.11 (0.04-0.33) | 0.13 (0.05-0.36) | 0.19 (0.08-0.54) |
| Bahamas | 0.41 (0.27-0.6) | 0.51 (0.37-0.72) | 0.2 (0.08-0.56) | 0.08 (0.03-0.23) | 0.09 (0.04-0.24) |
| Bosnia & Herzegovina | 2.98 (2.44-3.62) | 1.67 (1.37-2.02) | 0.13 (0.05-0.32) | 0.06 (0.03-0.14) | 0.14 (0.06-0.35) |
| Belarus | 1.22 (0.66-2.2) | 0.3 (0.16-0.54) | 0.14 (0.06-0.39) | 0.32 (0.14-0.87) | 0.18 (0.07-0.48) |
| Belize | 0.42 (0.29-0.61) | 0.75 (0.53-1.04) | 0.17 (0.07-0.51) | 0.13 (0.06-0.34) | 0.02 (0.01-0.05) |
| Bolivia | 0.43 (0.38-0.48) | 0.57 (0.51-0.63) | 0.29 (0.26-0.33) | 0.06 (0.05-0.07) | 0.11 (0.05-0.29) |
| Brazil | 0.41 (0.37-0.47) | 0.45 (0.41-0.5) | 0.04 (0.03-0.05) | 0.41 (0.35-0.48) | 0 (0-0) |
| Barbados | 0.62 (0.51-0.76) | 0.68 (0.57-0.81) | 0.17 (0.07-0.47) | 0.03 (0.02-0.06) | 0.08 (0.05-0.13) |
| Brunei | 0.68 (0.48-0.95) | 1.17 (0.89-1.56) | 0.08 (0.03-0.22) | 0.32 (0.14-0.81) | 0.06 (0.03-0.15) |
| Bhutan | 0.42 (0.31-0.57) | 0.67 (0.48-0.95) | 0.1 (0.04-0.31) | 0.17 (0.07-0.54) | 0.08 (0.03-0.28) |
| Botswana | 0.18 (0.14-0.23) | 0.46 (0.37-0.57) | 0.09 (0.05-0.21) | 0.08 (0.04-0.17) | 0.81 (0.35-1.62) |
| Central African Republic | 0.35 (0.26-0.47) | 0.44 (0.31-0.6) | 0.27 (0.22-0.33) | 0.15 (0.07-0.36) | 0.15 (0.06-0.38) |
| Canada | 1.57 (1.39-1.78) | 0.91 (0.83-1) | 0.21 (0.17-0.25) | 0.18 (0.15-0.22) | 0.15 (0.13-0.17) |
| Switzerland | 1.58 (1.33-1.86) | 0.78 (0.68-0.91) | 0.5 (0.2-1.39) | 0.03 (0.02-0.04) | 0.05 (0.03-0.13) |
| Chile | 0.53 (0.43-0.65) | 0.68 (0.57-0.81) | 0.35 (0.14-0.96) | 0.06 (0.04-0.11) | 0.16 (0.07-0.45) |
| China | 0.71 (0.64-0.79) | 2.15 (1.95-2.37) | 0.09 (0.04-0.24) | 0.15 (0.12-0.19) | 0.14 (0.11-0.17) |
| Côte d’lvoire | 0.22 (0.18-0.25) | 0.39 (0.34-0.45) | 0.08 (0.07-0.11) | 0.02 (0.01-0.03) | 0.17 (0.07-0.48) |
| Cameroon | 0.3 (0.26-0.34) | 0.53 (0.48-0.59) | 0.11 (0.1-0.13) | 0.13 (0.11-0.16) | 0.28 (0.12-0.69) |
| Congo - Kinshasa | 0.35 (0.31-0.39) | 0.93 (0.84-1.03) | 0.13 (0.11-0.15) | 0.14 (0.12-0.16) | 0.51 (0.22-1.23) |
| Congo - Brazzaville | 0.32 (0.28-0.36) | 0.6 (0.55-0.66) | 0.11 (0.09-0.14) | 0.1 (0.08-0.12) | 0.18 (0.08-0.49) |
| Colombia | 0.49 (0.45-0.53) | 0.47 (0.43-0.51) | 0.39 (0.35-0.44) | 0.13 (0.12-0.14) | 0.49 (0.22-1.12) |
| Comoros | 0.3 (0.26-0.35) | 0.41 (0.36-0.47) | 0.12 (0.1-0.14) | 0.04 (0.03-0.06) | 0.14 (0.06-0.38) |
| Cape Verde | 0.81 (0.58-1.11) | 0.78 (0.6-1.02) | 0.07 (0.03-0.18) | 0.12 (0.06-0.29) | 0.26 (0.11-0.73) |
| Costa Rica | 0.64 (0.45-0.93) | 0.81 (0.58-1.11) | 0.19 (0.08-0.51) | 0.13 (0.06-0.33) | 0.08 (0.03-0.19) |
| Cuba | 0.62 (0.47-0.83) | 0.54 (0.4-0.74) | 0.23 (0.1-0.61) | 0.12 (0.06-0.31) | 0 (0-0.01) |
| Cyprus | 0.71 (0.44-1.16) | 0.83 (0.51-1.38) | 0.16 (0.07-0.47) | 0.3 (0.11-0.77) | 0.04 (0.02-0.09) |
| Czechia | 0.37 (0.31-0.43) | 0.36 (0.3-0.42) | 0.13 (0.05-0.36) | 0.05 (0.02-0.13) | 0.05 (0.03-0.09) |
| Germany | 1.58 (1.41-1.78) | 1.3 (1.18-1.43) | 0.39 (0.33-0.46) | 0.05 (0.04-0.06) | 0.03 (0.02-0.03) |
| Djibouti | 0.5 (0.37-0.69) | 0.42 (0.3-0.61) | 0.06 (0.02-0.18) | 0.9 (0.42-2.31) | 0 (0-0) |
| Dominica | 0.52 (0.41-0.66) | 0.51 (0.41-0.62) | 0.22 (0.09-0.6) | 0.14 (0.07-0.36) | 0.04 (0.02-0.1) |
| Denmark | 1.04 (0.86-1.25) | 0.84 (0.72-0.98) | 0.32 (0.12-1.01) | 0.2 (0.09-0.52) | 0.07 (0.05-0.1) |
| Dominican Republic | 0.71 (0.64-0.77) | 0.52 (0.47-0.58) | 0.23 (0.2-0.26) | 0.23 (0.2-0.26) | 0.01 (0.01-0.03) |
| Algeria | 0.25 (0.2-0.32) | 0.44 (0.36-0.55) | 0.14 (0.06-0.42) | 0.07 (0.02-0.23) | 0.06 (0.02-0.14) |
| Ecuador | 0.13 (0.11-0.15) | 0.54 (0.48-0.62) | 0.11 (0.05-0.25) | 0.08 (0.04-0.14) | 0.03 (0.01-0.06) |
| Egypt | 0.28 (0.26-0.31) | 0.34 (0.32-0.37) | 0.1 (0.09-0.12) | 0.03 (0.03-0.04) | 0.02 (0.02-0.03) |
| Eritrea | 0.2 (0.15-0.28) | 0.29 (0.23-0.37) | 0.07 (0.03-0.18) | 0.11 (0.05-0.26) | 0.19 (0.08-0.46) |
| Spain | 0.73 (0.63-0.84) | 0.69 (0.61-0.77) | 0.16 (0.06-0.46) | 0.2 (0.09-0.43) | 0.07 (0.05-0.09) |
| Estonia | 0.52 (0.45-0.6) | 1 (0.86-1.17) | 0.01 (0.01-0.02) | 0.03 (0.02-0.04) | 0.14 (0.09-0.22) |
| Ethiopia | 0.14 (0.12-0.15) | 0.37 (0.34-0.41) | 0.07 (0.06-0.08) | 0.15 (0.13-0.17) | 0.03 (0.02-0.05) |
| Finland | 1.71 (1.49-1.95) | 0.86 (0.77-0.97) | 0.11 (0.04-0.26) | 0.06 (0.05-0.08) | 0.03 (0.02-0.03) |
| Fiji | 0.89 (0.6-1.3) | 1.56 (1.13-2.13) | 0.12 (0.05-0.31) | 0.28 (0.13-0.66) | 0.07 (0.03-0.15) |
| France | 1.75 (1.54-1.98) | 1.09 (0.98-1.2) | 0.02 (0.01-0.02) | 0.13 (0.11-0.16) | 0.02 (0.02-0.02) |
| Micronesia (Federated States of) | 1.17 (0.85-1.64) | 1.03 (0.7-1.55) | 0.11 (0.05-0.31) | 0.27 (0.12-0.69) | 0.04 (0.02-0.11) |
| Gabon | 0.25 (0.21-0.29) | 0.51 (0.45-0.58) | 0.14 (0.12-0.17) | 0.01 (0.01-0.01) | 0.38 (0.15-0.95) |
| United Kingdom | 1.06 (0.93-1.2) | 0.82 (0.74-0.91) | 0.22 (0.08-0.72) | 0.39 (0.31-0.49) | 0.05 (0.04-0.06) |
| Georgia | 0.36 (0.3-0.44) | 0.31 (0.26-0.38) | 0.08 (0.04-0.22) | 0.15 (0.07-0.37) | 0.1 (0.04-0.26) |
| Ghana | 0.31 (0.28-0.34) | 0.57 (0.53-0.61) | 0.09 (0.07-0.1) | 0.08 (0.07-0.1) | 0.56 (0.31-1.03) |
| Guinea | 0.08 (0.06-0.1) | 0.09 (0.07-0.1) | 0.04 (0.04-0.05) | 0.01 (0.01-0.02) | 0.2 (0.08-0.52) |
| Gambia | 0.11 (0.09-0.14) | 0.21 (0.16-0.26) | 0.04 (0.03-0.05) | 0.03 (0.02-0.04) | 0.21 (0.08-0.61) |
| Guinea-Bissau | 0.24 (0.18-0.32) | 0.58 (0.43-0.81) | 0.08 (0.04-0.21) | 0.38 (0.18-0.92) | 0.23 (0.1-0.63) |
| Equatorial Guinea | 0.32 (0.24-0.43) | 0.61 (0.44-0.86) | 0.12 (0.06-0.31) | 0.16 (0.07-0.36) | 0.2 (0.08-0.54) |
| Greece | 0.87 (0.74-1.02) | 0.61 (0.53-0.71) | 0.18 (0.07-0.53) | 0.13 (0.1-0.18) | 0.06 (0.05-0.08) |
| Grenada | 0.6 (0.41-0.86) | 1.24 (0.89-1.73) | 0.22 (0.1-0.59) | 0.14 (0.06-0.33) | 0 (0-0) |
| Guatemala | 0.38 (0.34-0.42) | 0.47 (0.43-0.52) | 0.15 (0.13-0.17) | 0.23 (0.2-0.27) | 0.02 (0.01-0.06) |
| Guyana | 1 (0.85-1.18) | 0.73 (0.63-0.84) | 0.19 (0.16-0.23) | 0.11 (0.09-0.13) | 0.08 (0.03-0.19) |
| Honduras | 0.44 (0.39-0.5) | 0.15 (0.13-0.17) | 0.14 (0.12-0.16) | 0.21 (0.18-0.24) | 0.05 (0.03-0.06) |
| Croatia | 0.29 (0.25-0.35) | 2.52 (2.13-3.01) | 0.14 (0.06-0.36) | 0.04 (0.01-0.1) | 0.16 (0.06-0.38) |
| Haiti | 0.35 (0.31-0.39) | 0.41 (0.37-0.46) | 0.1 (0.08-0.11) | 0.14 (0.12-0.16) | 0.05 (0.04-0.07) |
| Hungary | 0.29 (0.25-0.35) | 0.21 (0.18-0.25) | 0.11 (0.05-0.29) | 0.07 (0.02-0.22) | 0.03 (0.02-0.04) |
| Indonesia | 0.54 (0.5-0.59) | 1.17 (1.09-1.26) | 0.19 (0.16-0.21) | 0.28 (0.25-0.32) | 0.08 (0.06-0.1) |
| India | 0.16 (0.14-0.17) | 0.39 (0.36-0.42) | 0.07 (0.06-0.08) | 0.06 (0.06-0.07) | 0.04 (0.03-0.06) |
| Ireland | 0.94 (0.76-1.14) | 0.84 (0.7-0.97) | 0.27 (0.1-0.8) | 0.22 (0.09-0.49) | 0.11 (0.05-0.23) |
| Iran | 0.51 (0.45-0.58) | 0.49 (0.43-0.55) | 0.03 (0.03-0.04) | 0.06 (0.05-0.07) | 0.07 (0.06-0.09) |
| Iraq | 0.34 (0.23-0.49) | 0.28 (0.2-0.39) | 0.12 (0.05-0.35) | 0.13 (0.05-0.36) | 0.13 (0.05-0.34) |
| Iceland | 0.91 (0.78-1.06) | 0.5 (0.44-0.56) | 1.44 (0.52-4.17) | 0.05 (0.04-0.07) | 0.01 (0.01-0.02) |
| Israel | 0.41 (0.35-0.47) | 0.18 (0.16-0.21) | 0.2 (0.15-0.25) | 0.27 (0.21-0.34) | 0.29 (0.23-0.37) |
| Italy | 2.22 (1.98-2.52) | 0.99 (0.89-1.11) | 0.24 (0.2-0.3) | 0.13 (0.11-0.15) | 0.03 (0.02-0.03) |
| Jamaica | 0.86 (0.67-1.11) | 0.73 (0.6-0.9) | 0.16 (0.07-0.38) | 0.06 (0.04-0.08) | 0.03 (0.02-0.04) |
| Jordan | 0.54 (0.49-0.6) | 0.27 (0.25-0.3) | 0.08 (0.07-0.09) | 0.1 (0.09-0.11) | 0.11 (0.08-0.15) |
| Japan | 0.81 (0.76-0.87) | 1.93 (1.83-2.04) | 0.04 (0.03-0.07) | 0.54 (0.45-0.65) | 0.03 (0.03-0.04) |
| Kazakhstan | 0.31 (0.26-0.36) | 0.61 (0.52-0.72) | 0.19 (0.15-0.23) | 0.03 (0.01-0.07) | 1.67 (0.82-2.59) |
| Kenya | 0.37 (0.33-0.41) | 1.03 (0.95-1.11) | 0.17 (0.14-0.19) | 0.12 (0.11-0.14) | 0.01 (0.01-0.02) |
| Kyrgyzstan | 0.4 (0.34-0.47) | 0.16 (0.12-0.21) | 0.18 (0.15-0.22) | 0.03 (0.02-0.04) | 0.58 (0.25-1.3) |
| Cambodia | 0.4 (0.36-0.44) | 0.87 (0.8-0.96) | 0.12 (0.1-0.14) | 0.04 (0.03-0.05) | 0.02 (0.02-0.04) |
| Kiribati | 0.8 (0.53-1.19) | 0.67 (0.47-0.96) | 0.12 (0.05-0.32) | 0.29 (0.13-0.7) | 0.05 (0.02-0.11) |
| South Korea | 0.75 (0.69-0.82) | 0.95 (0.89-1.01) | 0.03 (0.03-0.04) | 0.27 (0.23-0.31) | 0.02 (0.02-0.02) |
| Kuwait | 0.17 (0.14-0.21) | 0.24 (0.2-0.28) | 0.07 (0.02-0.23) | 0.16 (0.06-0.49) | 0.23 (0.09-0.68) |
| Laos | 0.97 (0.85-1.1) | 1.05 (0.94-1.16) | 0.05 (0.03-0.07) | 0.25 (0.11-0.63) | 0.01 (0.01-0.01) |
| Lebanon | 0.69 (0.59-0.81) | 0.77 (0.66-0.89) | 0.06 (0.05-0.08) | 0.1 (0.08-0.13) | 0.44 (0.34-0.57) |
| Liberia | 0.18 (0.15-0.21) | 0.51 (0.45-0.59) | 0.09 (0.08-0.11) | 0.04 (0.03-0.05) | 0.05 (0.02-0.12) |
| Libya | 0.16 (0.13-0.21) | 0.25 (0.2-0.3) | 0.11 (0.04-0.31) | 0.13 (0.05-0.35) | 0.19 (0.08-0.48) |
| St. Lucia | 0.37 (0.26-0.54) | 0.44 (0.32-0.61) | 0.27 (0.11-0.75) | 0.27 (0.12-0.68) | 0 (0-0.01) |
| Sri Lanka | 0.35 (0.3-0.4) | 0.45 (0.4-0.5) | 0.21 (0.08-0.64) | 0.22 (0.18-0.26) | 0.12 (0.04-0.39) |
| Lesotho | 0.24 (0.21-0.28) | 0.78 (0.69-0.87) | 0.07 (0.06-0.08) | 0.06 (0.05-0.08) | 0.03 (0.01-0.08) |
| Lithuania | 0.34 (0.28-0.41) | 0.44 (0.37-0.53) | 0.14 (0.06-0.37) | 0.11 (0.05-0.27) | 0.17 (0.1-0.28) |
| Luxembourg | 1 (0.76-1.32) | 0.66 (0.52-0.83) | 0.34 (0.13-1.05) | 0.09 (0.03-0.32) | 0 (0-0) |
| Latvia | 0.29 (0.25-0.34) | 0.79 (0.67-0.93) | 0.16 (0.07-0.45) | 0.05 (0.02-0.11) | 0.12 (0.07-0.2) |
| Morocco | 0.29 (0.24-0.36) | 0.36 (0.31-0.43) | 0.12 (0.05-0.36) | 0.13 (0.05-0.39) | 0.15 (0.06-0.39) |
| Moldova | 0.48 (0.28-0.85) | 0.31 (0.17-0.57) | 0.15 (0.06-0.39) | 0.5 (0.22-1.23) | 0.26 (0.11-0.66) |
| Madagascar | 0.32 (0.29-0.36) | 0.7 (0.64-0.77) | 0.1 (0.08-0.11) | 0.04 (0.03-0.04) | 0.02 (0.01-0.04) |
| Maldives | 1 (0.85-1.18) | 0.39 (0.33-0.45) | 0.21 (0.17-0.25) | 0.07 (0.06-0.09) | 0.18 (0.06-0.6) |
| Mexico | 0.74 (0.67-0.81) | 0.96 (0.88-1.04) | 0.81 (0.74-0.89) | 0.2 (0.18-0.23) | 0.02 (0.01-0.02) |
| Marshall Islands | 0.89 (0.67-1.21) | 1.36 (0.98-1.97) | 0.1 (0.04-0.27) | 0.39 (0.17-0.96) | 0.05 (0.02-0.13) |
| North Macedonia | 0.46 (0.36-0.57) | 0.52 (0.42-0.64) | 0.1 (0.04-0.25) | 0.09 (0.04-0.21) | 0.19 (0.08-0.48) |
| Mali | 0.26 (0.23-0.29) | 0.4 (0.36-0.44) | 0.09 (0.08-0.11) | 0.07 (0.06-0.08) | 0.25 (0.08-0.77) |
| Malta | 1.52 (1.27-1.81) | 0.61 (0.53-0.71) | 0.31 (0.11-0.92) | 0.03 (0.02-0.07) | 0.06 (0.03-0.11) |
| Myanmar (Burma) | 0.64 (0.55-0.75) | 1.26 (1.12-1.43) | 0.14 (0.06-0.4) | 0.23 (0.1-0.65) | 0.07 (0.03-0.18) |
| Montenegro | 1.23 (0.73-2.08) | 0.48 (0.28-0.85) | 0.12 (0.05-0.32) | 0.14 (0.05-0.37) | 0.19 (0.07-0.48) |
| Mongolia | 0.26 (0.21-0.32) | 0.69 (0.57-0.83) | 0.06 (0.02-0.16) | 0.13 (0.06-0.33) | 0.03 (0.01-0.08) |
| Mozambique | 0.37 (0.32-0.42) | 0.6 (0.53-0.67) | 0.13 (0.11-0.16) | 0.14 (0.11-0.17) | 0.21 (0.13-0.32) |
| Mauritania | 0.12 (0.1-0.15) | 0.94 (0.82-1.09) | 0.05 (0.02-0.13) | 0.08 (0.04-0.19) | 0.03 (0.01-0.09) |
| Mauritius | 0.26 (0.22-0.29) | 0.7 (0.62-0.78) | 0.09 (0.04-0.21) | 0.15 (0.1-0.22) | 0.16 (0.07-0.4) |
| Malawi | 0.41 (0.37-0.45) | 0.74 (0.69-0.79) | 0.1 (0.08-0.11) | 0.11 (0.09-0.12) | 0.14 (0.06-0.41) |
| Malaysia | 0.86 (0.76-0.97) | 1.18 (1.07-1.3) | 0.08 (0.06-0.1) | 0.26 (0.21-0.31) | 0.88 (0.66-1.17) |
| Namibia | 0.9 (0.81-0.99) | 0.63 (0.58-0.69) | 0.09 (0.07-0.1) | 0.05 (0.05-0.06) | 0.15 (0.07-0.38) |
| Niger | 0.24 (0.2-0.27) | 0.44 (0.39-0.5) | 0.58 (0.49-0.69) | 0.13 (0.11-0.16) | 0.3 (0.12-0.96) |
| Nigeria | 0.13 (0.11-0.14) | 0.4 (0.36-0.44) | 0.07 (0.06-0.08) | 0.09 (0.08-0.1) | 0.2 (0.09-0.54) |
| Nicaragua | 0.77 (0.58-1.02) | 1.4 (1.03-1.92) | 0.32 (0.14-0.83) | 0.13 (0.06-0.33) | 0.05 (0.02-0.13) |
| Netherlands | 1.22 (1.09-1.37) | 0.81 (0.74-0.88) | 0.04 (0.03-0.05) | 0.05 (0.04-0.06) | 0.04 (0.03-0.04) |
| Norway | 0.96 (0.83-1.12) | 0.92 (0.82-1.04) | 0.53 (0.2-1.73) | 0.01 (0-0.03) | 0.03 (0.02-0.04) |
| Nepal | 0.16 (0.14-0.18) | 0.45 (0.41-0.49) | 0.15 (0.13-0.17) | 0.15 (0.13-0.18) | 0.06 (0.02-0.22) |
| New Zealand | 1.81 (1.47-2.22) | 0.92 (0.77-1.09) | 0.23 (0.09-0.78) | 0.59 (0.45-0.76) | 0.02 (0.01-0.02) |
| Oman | 0.38 (0.27-0.51) | 0.34 (0.26-0.45) | 0.06 (0.03-0.19) | 0.15 (0.06-0.43) | 0.16 (0.07-0.43) |
| Pakistan | 0.15 (0.13-0.17) | 0.21 (0.19-0.24) | 0.09 (0.08-0.11) | 0.03 (0.02-0.04) | 0.04 (0.01-0.13) |
| Panama | 0.64 (0.49-0.84) | 0.75 (0.56-1.01) | 0.16 (0.07-0.4) | 0.31 (0.14-0.75) | 0.15 (0.07-0.36) |
| Peru | 1.12 (1.04-1.21) | 0.47 (0.44-0.51) | 0.35 (0.31-0.39) | 0.17 (0.15-0.19) | 0.06 (0.03-0.18) |
| Philippines | 0.69 (0.63-0.75) | 0.82 (0.76-0.88) | 0.14 (0.12-0.16) | 0.09 (0.08-0.11) | 0.01 (0.01-0.01) |
| Papua New Guinea | 0.03 (0.02-0.05) | 0.62 (0.51-0.76) | 0.59 (0.21-1.89) | 0.05 (0.02-0.14) | 0 (0-0) |
| Poland | 1.16 (1.02-1.32) | 0.61 (0.53-0.71) | 0.01 (0-0.01) | 0.01 (0.01-0.02) | 0.06 (0.04-0.09) |
| Portugal | 4.87 (4.34-5.45) | 1.08 (0.99-1.17) | 0.27 (0.24-0.31) | 0.11 (0.1-0.13) | 0.02 (0.02-0.03) |
| Paraguay | 0.46 (0.4-0.55) | 0.34 (0.3-0.39) | 0.18 (0.08-0.45) | 0.08 (0.04-0.17) | 0.06 (0.03-0.14) |
| Palestinian Territories | 0.35 (0.24-0.5) | 0.34 (0.24-0.46) | 0.13 (0.05-0.38) | 0.14 (0.06-0.36) | 0.2 (0.08-0.51) |
| Qatar | 0.38 (0.25-0.55) | 0.36 (0.26-0.5) | 0.11 (0.04-0.34) | 0.13 (0.04-0.38) | 0.18 (0.07-0.51) |
| Romania | 0.96 (0.79-1.16) | 1.22 (1.03-1.46) | 0.05 (0.04-0.07) | 0.15 (0.11-0.21) | 0.19 (0.11-0.31) |
| Russia | 0.45 (0.39-0.52) | 0.63 (0.53-0.73) | 0.09 (0.04-0.27) | 0 (0-0.01) | 0.28 (0.18-0.44) |
| Rwanda | 0.43 (0.38-0.49) | 1.18 (1.07-1.3) | 0.18 (0.15-0.21) | 0.35 (0.3-0.4) | 0.13 (0.05-0.37) |
| Saudi Arabia | 0.45 (0.33-0.62) | 0.32 (0.23-0.46) | 0.1 (0.04-0.3) | 0.2 (0.08-0.54) | 0.24 (0.1-0.62) |
| Sudan | 0.29 (0.2-0.43) | 0.55 (0.4-0.77) | 0.05 (0.02-0.13) | 0.09 (0.04-0.22) | 0.26 (0.11-0.68) |
| Senegal | 0.19 (0.17-0.21) | 0.31 (0.28-0.34) | 0.1 (0.09-0.12) | 0.08 (0.07-0.09) | 0.14 (0.06-0.36) |
| Singapore | 1.07 (0.88-1.3) | 1.05 (0.89-1.24) | 0.1 (0.04-0.33) | 0.15 (0.12-0.2) | 0.04 (0.03-0.05) |
| Solomon Islands | 0.49 (0.39-0.63) | 1.35 (1.04-1.74) | 0.13 (0.05-0.36) | 0.29 (0.13-0.73) | 0.02 (0.01-0.05) |
| Sierra Leone | 0.32 (0.28-0.36) | 0.5 (0.45-0.56) | 0.05 (0.04-0.06) | 0.1 (0.08-0.11) | 0.04 (0.02-0.13) |
| El Salvador | 0.6 (0.41-0.89) | 0.63 (0.46-0.89) | 0.27 (0.11-0.72) | 0.16 (0.07-0.44) | 0.03 (0.01-0.08) |
| Serbia | 0.73 (0.48-1.14) | 2.03 (1.32-3.1) | 0.25 (0.11-0.64) | 0.14 (0.07-0.29) | 0.07 (0.03-0.17) |
| South Sudan | 0.23 (0.17-0.32) | 0.61 (0.43-0.88) | 0.08 (0.04-0.22) | 0.12 (0.05-0.3) | 0.14 (0.06-0.37) |
| São Tomé and Príncipe | 0.46 (0.38-0.56) | 0.15 (0.13-0.18) | 0.17 (0.14-0.2) | 0.04 (0.03-0.05) | 0.16 (0.06-0.47) |
| Suriname | 0.51 (0.35-0.74) | 0.9 (0.65-1.25) | 0.18 (0.08-0.46) | 0.35 (0.16-0.82) | 0.14 (0.06-0.35) |
| Slovakia | 0.4 (0.34-0.47) | 0.34 (0.29-0.41) | 0.01 (0-0.02) | 0.02 (0.01-0.03) | 0.06 (0.04-0.09) |
| Slovenia | 0.38 (0.32-0.45) | 0.39 (0.32-0.47) | 0.35 (0.15-0.95) | 0.09 (0.04-0.2) | 0.16 (0.08-0.35) |
| Sweden | 1.33 (1.18-1.51) | 1.05 (0.95-1.16) | 0.32 (0.27-0.38) | 0.15 (0.12-0.18) | 0.04 (0.04-0.05) |
| Eswatini | 0.32 (0.29-0.36) | 0.69 (0.59-0.79) | 0.08 (0.07-0.1) | 0.05 (0.04-0.06) | 0.13 (0.09-0.19) |
| Seychelles | 0.37 (0.32-0.43) | 0.85 (0.75-0.96) | 0.03 (0.02-0.03) | 0.08 (0.07-0.1) | 0.17 (0.07-0.44) |
| Syria | 0.32 (0.23-0.43) | 0.3 (0.23-0.38) | 0.12 (0.05-0.35) | 0.13 (0.05-0.36) | 0.19 (0.08-0.5) |
| Chad | 0.15 (0.14-0.18) | 0.16 (0.14-0.18) | 0.09 (0.07-0.1) | 0.03 (0.03-0.04) | 0.17 (0.07-0.45) |
| Togo | 0.2 (0.16-0.24) | 0.63 (0.53-0.76) | 0.07 (0.05-0.09) | 0.06 (0.05-0.08) | 0.17 (0.08-0.43) |
| Thailand | 0.76 (0.57-1.02) | 1.22 (0.95-1.59) | 0.09 (0.04-0.24) | 0.41 (0.17-1.01) | 0.02 (0.01-0.04) |
| Tajikistan | 0.5 (0.4-0.62) | 0.24 (0.19-0.3) | 0.19 (0.16-0.23) | 0.29 (0.22-0.37) | 0.06 (0.03-0.18) |
| Turkmenistan | 0.36 (0.27-0.49) | 0.43 (0.31-0.59) | 0.09 (0.04-0.24) | 0.15 (0.06-0.37) | 0.01 (0-0.03) |
| Timor-Leste | 0.42 (0.36-0.49) | 1.08 (0.96-1.22) | 0.14 (0.11-0.17) | 0.09 (0.07-0.11) | 0.02 (0.01-0.06) |
| Tonga | 1.05 (0.84-1.34) | 1.05 (0.88-1.25) | 0.09 (0.03-0.31) | 0.36 (0.15-1.09) | 0.05 (0.02-0.13) |
| Trinidad & Tobago | 0.33 (0.29-0.39) | 0.42 (0.36-0.48) | 0.13 (0.09-0.16) | 0.19 (0.15-0.23) | 0.09 (0.04-0.2) |
| Tunisia | 0.19 (0.16-0.23) | 0.42 (0.36-0.49) | 0.14 (0.06-0.42) | 0.06 (0.03-0.1) | 0.07 (0.05-0.1) |
| Turkey | 0.24 (0.2-0.28) | 0.24 (0.21-0.28) | 0.13 (0.05-0.34) | 0.12 (0.08-0.18) | 0.11 (0.07-0.19) |
| Taiwan | 1.1 (0.88-1.36) | 1.93 (1.64-2.3) | 0.11 (0.05-0.3) | 0.06 (0.05-0.08) | 0.04 (0.03-0.05) |
| Tanzania | 0.24 (0.21-0.27) | 0.73 (0.66-0.8) | 0.07 (0.06-0.09) | 0.15 (0.13-0.17) | 0.3 (0.13-0.77) |
| Uganda | 0.2 (0.18-0.23) | 0.48 (0.44-0.53) | 0.08 (0.07-0.09) | 0.2 (0.18-0.23) | 0.14 (0.06-0.39) |
| Ukraine | 0.46 (0.39-0.55) | 0.62 (0.52-0.75) | 0.12 (0.05-0.33) | 0.04 (0.02-0.1) | 0.21 (0.09-0.6) |
| Uruguay | 0.47 (0.4-0.54) | 0.78 (0.69-0.87) | 0.17 (0.07-0.53) | 0.2 (0.08-0.58) | 0.24 (0.1-0.66) |
| United States | 1.03 (0.94-1.12) | 0.91 (0.85-0.98) | 0.19 (0.17-0.22) | 0.19 (0.17-0.21) | 0.1 (0.09-0.11) |
| Uzbekistan | 0.44 (0.35-0.54) | 0.87 (0.63-1.19) | 0.25 (0.2-0.31) | 0.15 (0.07-0.37) | 0.04 (0.02-0.12) |
| St. Vincent & Grenadines | 0.7 (0.47-1.04) | 0.51 (0.36-0.69) | 0.16 (0.07-0.41) | 0.22 (0.1-0.53) | 0.06 (0.03-0.14) |
| Venezuela | 0.53 (0.37-0.77) | 0.45 (0.32-0.62) | 0.22 (0.09-0.66) | 0.19 (0.08-0.47) | 0.01 (0-0.02) |
| Vietnam | 0.66 (0.57-0.77) | 1.27 (1.14-1.42) | 0.3 (0.2-0.47) | 1 (0.65-1.52) | 0.3 (0.13-0.74) |
| Vanuatu | 0.34 (0.26-0.43) | 0.55 (0.43-0.7) | 0.11 (0.05-0.32) | 0.68 (0.29-1.76) | 0.06 (0.03-0.15) |
| Samoa | 1.15 (0.89-1.48) | 2.58 (2.1-3.16) | 0.1 (0.04-0.27) | 2.8 (1.25-6.57) | 0.42 (0.19-1.02) |
| Yemen | 0.13 (0.11-0.15) | 0.14 (0.12-0.17) | 0.13 (0.11-0.16) | 0.11 (0.09-0.13) | 0.03 (0.01-0.1) |
| South Africa | 0.11 (0.1-0.12) | 0.53 (0.48-0.58) | 0.1 (0.05-0.25) | 0.14 (0.1-0.18) | 0 (0-0.01) |
| Zambia | 0.18 (0.17-0.2) | 0.84 (0.78-0.9) | 0.06 (0.05-0.07) | 0.09 (0.08-0.11) | 0.38 (0.17-0.86) |
| Zimbabwe | 0.3 (0.27-0.33) | 0.6 (0.55-0.65) | 0.09 (0.08-0.11) | 0.08 (0.07-0.1) | 0.06 (0.04-0.09) |

Table S18: National consumption (servings/day) in children 3-4 years in 2018.

| Country | Fruit | Vegetables (non-starchy) | Other non-potato starchy vegetables | | Beans and legumes | Nuts and seeds |
| --- | --- | --- | --- | --- | --- | --- |
| Afghanistan | 0.48 (0.32-0.71) | 0.4 (0.25-0.65) | | 0.22 (0.07-0.77) | 0.39 (0.13-1.31) | 0.74 (0.22-2.3) |
| Angola | 0.76 (0.58-1.01) | 1.93 (1.43-2.63) | | 0.17 (0.08-0.4) | 0.2 (0.1-0.44) | 0.33 (0.14-0.84) |
| Albania | 0.9 (0.77-1.07) | 0.47 (0.39-0.56) | | 0.27 (0.21-0.36) | 0.2 (0.16-0.25) | 0.4 (0.18-0.97) |
| United Arab Emirates | 0.57 (0.48-0.68) | 0.42 (0.36-0.48) | | 0.23 (0.08-0.7) | 0.17 (0.07-0.49) | 0.55 (0.22-1.39) |
| Argentina | 0.71 (0.62-0.81) | 0.75 (0.65-0.85) | | 0.03 (0.02-0.04) | 0.02 (0.02-0.03) | 0.03 (0.02-0.04) |
| Armenia | 0.59 (0.49-0.72) | 0.35 (0.29-0.43) | | 0.36 (0.29-0.45) | 0.07 (0.05-0.11) | 0.64 (0.41-1.01) |
| Antigua & Barbuda | 0.72 (0.49-1.05) | 1.4 (1-1.94) | | 0.21 (0.09-0.55) | 0.24 (0.1-0.58) | 0.04 (0.02-0.1) |
| Australia | 1.25 (1.03-1.51) | 0.65 (0.55-0.77) | | 0.08 (0.03-0.27) | 0.15 (0.11-0.21) | 0.12 (0.1-0.15) |
| Austria | 0.96 (0.84-1.1) | 0.63 (0.57-0.7) | | 0.15 (0.06-0.41) | 0.04 (0.03-0.06) | 0.09 (0.07-0.11) |
| Azerbaijan | 0.69 (0.59-0.82) | 0.29 (0.24-0.35) | | 0.16 (0.13-0.2) | 0.07 (0.06-0.1) | 1.06 (0.47-2.1) |
| Burundi | 0.47 (0.38-0.58) | 1.11 (0.93-1.29) | | 0.13 (0.1-0.16) | 0.23 (0.19-0.28) | 0.18 (0.07-0.57) |
| Belgium | 0.96 (0.85-1.08) | 0.62 (0.55-0.69) | | 0.11 (0.04-0.36) | 0.03 (0.02-0.04) | 0.06 (0.05-0.07) |
| Benin | 0.47 (0.42-0.53) | 0.58 (0.52-0.64) | | 0.07 (0.06-0.08) | 0.14 (0.12-0.16) | 0.36 (0.16-0.96) |
| Burkina Faso | 0.22 (0.19-0.25) | 0.16 (0.14-0.18) | | 0.02 (0.02-0.02) | 0.05 (0.04-0.06) | 0.66 (0.22-1.92) |
| Bangladesh | 0.39 (0.36-0.42) | 0.86 (0.81-0.92) | | 0.06 (0.05-0.07) | 0.11 (0.1-0.12) | 0.01 (0.01-0.02) |
| Bulgaria | 0.61 (0.51-0.73) | 1.36 (1.18-1.57) | | 0.02 (0.01-0.03) | 0.09 (0.07-0.12) | 0.44 (0.32-0.6) |
| Bahrain | 0.62 (0.45-0.88) | 0.62 (0.43-0.94) | | 0.12 (0.05-0.38) | 0.17 (0.07-0.47) | 0.36 (0.14-0.96) |
| Bahamas | 0.63 (0.42-0.92) | 0.63 (0.45-0.88) | | 0.21 (0.09-0.58) | 0.1 (0.04-0.24) | 0.16 (0.07-0.42) |
| Bosnia & Herzegovina | 3.86 (3.22-4.62) | 1.68 (1.44-1.96) | | 0.14 (0.06-0.34) | 0.07 (0.03-0.15) | 0.3 (0.14-0.7) |
| Belarus | 1.59 (0.87-2.83) | 0.3 (0.16-0.54) | | 0.15 (0.06-0.43) | 0.35 (0.15-0.96) | 0.36 (0.16-0.95) |
| Belize | 0.65 (0.45-0.94) | 0.92 (0.66-1.29) | | 0.18 (0.07-0.52) | 0.15 (0.06-0.38) | 0.04 (0.02-0.1) |
| Bolivia | 0.66 (0.59-0.73) | 0.7 (0.64-0.77) | | 0.31 (0.27-0.35) | 0.07 (0.06-0.08) | 0.2 (0.09-0.5) |
| Brazil | 0.64 (0.57-0.71) | 0.56 (0.51-0.61) | | 0.04 (0.03-0.05) | 0.47 (0.4-0.55) | 0 (0-0) |
| Barbados | 0.95 (0.79-1.16) | 0.84 (0.7-0.99) | | 0.18 (0.07-0.49) | 0.04 (0.02-0.06) | 0.15 (0.1-0.23) |
| Brunei | 0.84 (0.61-1.18) | 1.18 (0.9-1.57) | | 0.07 (0.03-0.19) | 0.26 (0.12-0.65) | 0.09 (0.04-0.23) |
| Bhutan | 0.69 (0.51-0.92) | 1.12 (0.81-1.59) | | 0.1 (0.04-0.32) | 0.24 (0.09-0.68) | 0.14 (0.04-0.43) |
| Botswana | 0.31 (0.24-0.4) | 0.62 (0.51-0.77) | | 0.11 (0.05-0.24) | 0.1 (0.05-0.21) | 1.2 (0.56-2.24) |
| Central African Republic | 0.62 (0.47-0.82) | 0.6 (0.43-0.83) | | 0.31 (0.26-0.38) | 0.19 (0.09-0.46) | 0.22 (0.09-0.59) |
| Canada | 1.26 (1.14-1.39) | 0.71 (0.66-0.77) | | 0.08 (0.07-0.1) | 0.12 (0.1-0.14) | 0.29 (0.25-0.32) |
| Switzerland | 1.26 (1.08-1.48) | 0.62 (0.54-0.71) | | 0.2 (0.08-0.58) | 0.02 (0.01-0.02) | 0.11 (0.05-0.25) |
| Chile | 0.81 (0.67-0.98) | 0.84 (0.7-0.99) | | 0.36 (0.14-1.02) | 0.07 (0.04-0.13) | 0.29 (0.12-0.81) |
| China | 0.89 (0.8-0.98) | 2.16 (1.98-2.37) | | 0.08 (0.04-0.22) | 0.12 (0.1-0.15) | 0.21 (0.17-0.26) |
| Côte d’lvoire | 0.38 (0.33-0.45) | 0.54 (0.46-0.62) | | 0.1 (0.08-0.12) | 0.03 (0.02-0.04) | 0.27 (0.11-0.71) |
| Cameroon | 0.52 (0.46-0.59) | 0.72 (0.65-0.8) | | 0.13 (0.11-0.15) | 0.17 (0.14-0.2) | 0.43 (0.18-1.04) |
| Congo - Kinshasa | 0.61 (0.54-0.69) | 1.27 (1.15-1.4) | | 0.15 (0.13-0.18) | 0.17 (0.15-0.2) | 0.79 (0.34-1.84) |
| Congo - Brazzaville | 0.57 (0.5-0.64) | 0.82 (0.75-0.91) | | 0.13 (0.11-0.16) | 0.13 (0.11-0.15) | 0.28 (0.12-0.73) |
| Colombia | 0.75 (0.69-0.82) | 0.58 (0.53-0.63) | | 0.41 (0.36-0.46) | 0.15 (0.13-0.16) | 0.86 (0.38-1.83) |
| Comoros | 0.53 (0.46-0.62) | 0.56 (0.49-0.63) | | 0.13 (0.11-0.16) | 0.05 (0.04-0.07) | 0.22 (0.1-0.55) |
| Cape Verde | 1.42 (1.02-1.95) | 1.06 (0.81-1.39) | | 0.08 (0.03-0.2) | 0.15 (0.07-0.37) | 0.41 (0.16-1.16) |
| Costa Rica | 0.98 (0.69-1.42) | 1 (0.71-1.36) | | 0.2 (0.09-0.53) | 0.15 (0.07-0.39) | 0.14 (0.06-0.36) |
| Cuba | 0.96 (0.72-1.29) | 0.67 (0.49-0.92) | | 0.24 (0.1-0.66) | 0.14 (0.06-0.35) | 0.01 (0-0.01) |
| Cyprus | 0.57 (0.35-0.93) | 0.66 (0.4-1.09) | | 0.07 (0.03-0.19) | 0.19 (0.07-0.5) | 0.07 (0.03-0.17) |
| Czechia | 0.47 (0.42-0.54) | 0.36 (0.32-0.41) | | 0.14 (0.06-0.39) | 0.06 (0.03-0.15) | 0.11 (0.08-0.17) |
| Germany | 1.27 (1.14-1.4) | 1.02 (0.94-1.11) | | 0.16 (0.13-0.18) | 0.03 (0.03-0.04) | 0.05 (0.04-0.06) |
| Djibouti | 0.87 (0.64-1.2) | 0.58 (0.41-0.84) | | 0.07 (0.03-0.21) | 1.16 (0.55-2.79) | 0 (0-0.01) |
| Dominica | 0.8 (0.63-1.01) | 0.62 (0.51-0.77) | | 0.24 (0.1-0.63) | 0.16 (0.08-0.4) | 0.07 (0.03-0.18) |
| Denmark | 0.83 (0.7-0.99) | 0.66 (0.57-0.76) | | 0.13 (0.05-0.4) | 0.13 (0.06-0.35) | 0.14 (0.1-0.19) |
| Dominican Republic | 1.08 (0.99-1.19) | 0.64 (0.58-0.7) | | 0.24 (0.21-0.27) | 0.26 (0.23-0.3) | 0.02 (0.01-0.06) |
| Algeria | 0.43 (0.34-0.55) | 0.7 (0.58-0.85) | | 0.16 (0.06-0.48) | 0.09 (0.03-0.29) | 0.1 (0.04-0.26) |
| Ecuador | 0.2 (0.17-0.23) | 0.67 (0.59-0.76) | | 0.12 (0.05-0.26) | 0.09 (0.05-0.16) | 0.05 (0.02-0.11) |
| Egypt | 0.49 (0.45-0.53) | 0.55 (0.51-0.59) | | 0.12 (0.1-0.13) | 0.04 (0.04-0.05) | 0.04 (0.03-0.06) |
| Eritrea | 0.36 (0.26-0.49) | 0.4 (0.31-0.5) | | 0.08 (0.04-0.2) | 0.14 (0.07-0.33) | 0.28 (0.13-0.72) |
| Spain | 0.58 (0.51-0.66) | 0.54 (0.49-0.6) | | 0.06 (0.03-0.18) | 0.13 (0.06-0.28) | 0.14 (0.1-0.18) |
| Estonia | 0.67 (0.6-0.76) | 1.01 (0.9-1.14) | | 0.01 (0.01-0.02) | 0.03 (0.02-0.04) | 0.3 (0.21-0.41) |
| Ethiopia | 0.24 (0.22-0.27) | 0.51 (0.46-0.56) | | 0.08 (0.07-0.09) | 0.19 (0.17-0.21) | 0.05 (0.04-0.07) |
| Finland | 1.38 (1.22-1.54) | 0.68 (0.61-0.75) | | 0.04 (0.02-0.1) | 0.04 (0.03-0.05) | 0.05 (0.04-0.06) |
| Fiji | 1.11 (0.75-1.61) | 1.57 (1.14-2.14) | | 0.11 (0.05-0.28) | 0.23 (0.1-0.52) | 0.1 (0.05-0.24) |
| France | 1.4 (1.27-1.55) | 0.86 (0.79-0.94) | | 0.01 (0-0.01) | 0.08 (0.07-0.1) | 0.04 (0.04-0.05) |
| Micronesia (Federated States of) | 1.46 (1.06-2.04) | 1.04 (0.7-1.56) | | 0.1 (0.04-0.28) | 0.21 (0.09-0.57) | 0.07 (0.03-0.17) |
| Gabon | 0.44 (0.38-0.5) | 0.7 (0.61-0.79) | | 0.16 (0.14-0.19) | 0.01 (0.01-0.02) | 0.56 (0.23-1.45) |
| United Kingdom | 0.85 (0.77-0.94) | 0.64 (0.59-0.71) | | 0.09 (0.03-0.28) | 0.25 (0.21-0.31) | 0.09 (0.07-0.11) |
| Georgia | 0.47 (0.4-0.56) | 0.31 (0.27-0.37) | | 0.09 (0.04-0.25) | 0.16 (0.07-0.42) | 0.2 (0.09-0.53) |
| Ghana | 0.54 (0.5-0.59) | 0.78 (0.73-0.84) | | 0.1 (0.09-0.12) | 0.11 (0.09-0.12) | 0.86 (0.47-1.55) |
| Guinea | 0.14 (0.11-0.17) | 0.12 (0.1-0.14) | | 0.05 (0.04-0.06) | 0.02 (0.01-0.02) | 0.3 (0.13-0.77) |
| Gambia | 0.2 (0.15-0.25) | 0.28 (0.22-0.35) | | 0.04 (0.04-0.06) | 0.04 (0.03-0.05) | 0.33 (0.13-1) |
| Guinea-Bissau | 0.42 (0.32-0.57) | 0.79 (0.58-1.12) | | 0.1 (0.05-0.24) | 0.48 (0.23-1.18) | 0.36 (0.16-0.96) |
| Equatorial Guinea | 0.56 (0.43-0.74) | 0.83 (0.6-1.2) | | 0.14 (0.07-0.34) | 0.19 (0.09-0.48) | 0.31 (0.14-0.82) |
| Greece | 0.69 (0.6-0.8) | 0.48 (0.42-0.55) | | 0.07 (0.03-0.22) | 0.09 (0.06-0.11) | 0.12 (0.09-0.15) |
| Grenada | 0.92 (0.63-1.33) | 1.52 (1.1-2.13) | | 0.24 (0.1-0.59) | 0.16 (0.07-0.38) | 0 (0-0) |
| Guatemala | 0.58 (0.52-0.64) | 0.58 (0.54-0.64) | | 0.16 (0.14-0.18) | 0.27 (0.23-0.31) | 0.04 (0.02-0.11) |
| Guyana | 1.54 (1.32-1.81) | 0.89 (0.78-1.03) | | 0.2 (0.17-0.24) | 0.12 (0.1-0.15) | 0.14 (0.06-0.35) |
| Honduras | 0.68 (0.6-0.76) | 0.18 (0.16-0.21) | | 0.15 (0.13-0.17) | 0.24 (0.21-0.27) | 0.08 (0.06-0.12) |
| Croatia | 0.38 (0.33-0.45) | 2.54 (2.21-2.92) | | 0.16 (0.07-0.42) | 0.04 (0.02-0.11) | 0.33 (0.14-0.77) |
| Haiti | 0.54 (0.48-0.6) | 0.5 (0.45-0.56) | | 0.1 (0.09-0.12) | 0.16 (0.14-0.18) | 0.09 (0.07-0.13) |
| Hungary | 0.38 (0.33-0.44) | 0.21 (0.19-0.24) | | 0.13 (0.05-0.33) | 0.08 (0.02-0.24) | 0.06 (0.04-0.08) |
| Indonesia | 0.68 (0.63-0.73) | 1.18 (1.1-1.26) | | 0.17 (0.15-0.19) | 0.22 (0.2-0.25) | 0.12 (0.1-0.15) |
| India | 0.25 (0.23-0.28) | 0.64 (0.6-0.69) | | 0.07 (0.06-0.08) | 0.09 (0.08-0.1) | 0.07 (0.06-0.09) |
| Ireland | 0.75 (0.62-0.91) | 0.66 (0.56-0.76) | | 0.11 (0.04-0.32) | 0.14 (0.06-0.31) | 0.22 (0.11-0.44) |
| Iran | 0.89 (0.81-0.98) | 0.78 (0.71-0.86) | | 0.04 (0.03-0.05) | 0.07 (0.06-0.09) | 0.13 (0.11-0.16) |
| Iraq | 0.58 (0.4-0.85) | 0.45 (0.33-0.61) | | 0.14 (0.05-0.38) | 0.17 (0.07-0.45) | 0.24 (0.1-0.65) |
| Iceland | 0.73 (0.63-0.84) | 0.39 (0.35-0.44) | | 0.59 (0.21-1.77) | 0.03 (0.03-0.04) | 0.02 (0.02-0.03) |
| Israel | 0.7 (0.62-0.8) | 0.29 (0.26-0.32) | | 0.22 (0.17-0.28) | 0.34 (0.28-0.42) | 0.54 (0.45-0.66) |
| Italy | 1.78 (1.62-1.99) | 0.78 (0.71-0.86) | | 0.1 (0.08-0.12) | 0.08 (0.07-0.1) | 0.05 (0.05-0.06) |
| Jamaica | 1.33 (1.03-1.7) | 0.9 (0.74-1.1) | | 0.17 (0.07-0.4) | 0.07 (0.04-0.1) | 0.05 (0.04-0.08) |
| Jordan | 0.94 (0.85-1.03) | 0.43 (0.4-0.47) | | 0.09 (0.08-0.1) | 0.13 (0.11-0.14) | 0.21 (0.15-0.28) |
| Japan | 1.02 (0.97-1.07) | 1.94 (1.87-2.02) | | 0.04 (0.02-0.06) | 0.43 (0.36-0.51) | 0.05 (0.05-0.05) |
| Kazakhstan | 0.4 (0.34-0.47) | 0.61 (0.54-0.7) | | 0.21 (0.17-0.25) | 0.03 (0.02-0.08) | 2.82 (1.7-3.74) |
| Kenya | 0.65 (0.59-0.71) | 1.4 (1.3-1.51) | | 0.19 (0.17-0.22) | 0.15 (0.13-0.18) | 0.02 (0.01-0.03) |
| Kyrgyzstan | 0.52 (0.44-0.61) | 0.16 (0.12-0.21) | | 0.2 (0.17-0.24) | 0.03 (0.02-0.05) | 1.19 (0.54-2.24) |
| Cambodia | 0.49 (0.44-0.55) | 0.88 (0.81-0.96) | | 0.11 (0.09-0.12) | 0.03 (0.03-0.04) | 0.04 (0.03-0.06) |
| Kiribati | 0.99 (0.67-1.48) | 0.67 (0.47-0.96) | | 0.11 (0.05-0.27) | 0.23 (0.1-0.56) | 0.08 (0.03-0.18) |
| South Korea | 0.94 (0.88-1.01) | 0.96 (0.91-1.01) | | 0.03 (0.02-0.03) | 0.21 (0.19-0.24) | 0.03 (0.03-0.04) |
| Kuwait | 0.3 (0.25-0.36) | 0.37 (0.32-0.43) | | 0.08 (0.03-0.25) | 0.21 (0.07-0.62) | 0.43 (0.16-1.18) |
| Laos | 1.21 (1.07-1.36) | 1.06 (0.96-1.16) | | 0.04 (0.03-0.06) | 0.2 (0.08-0.51) | 0.02 (0.01-0.02) |
| Lebanon | 1.2 (1.04-1.39) | 1.22 (1.08-1.39) | | 0.07 (0.05-0.09) | 0.13 (0.11-0.16) | 0.82 (0.66-1.02) |
| Liberia | 0.31 (0.26-0.37) | 0.7 (0.61-0.8) | | 0.11 (0.09-0.13) | 0.05 (0.04-0.06) | 0.07 (0.03-0.19) |
| Libya | 0.28 (0.22-0.36) | 0.4 (0.33-0.48) | | 0.12 (0.05-0.35) | 0.17 (0.07-0.46) | 0.35 (0.15-0.87) |
| St. Lucia | 0.57 (0.4-0.82) | 0.54 (0.39-0.74) | | 0.29 (0.12-0.77) | 0.3 (0.13-0.77) | 0.01 (0-0.02) |
| Sri Lanka | 0.56 (0.5-0.64) | 0.75 (0.68-0.83) | | 0.21 (0.08-0.65) | 0.31 (0.25-0.37) | 0.2 (0.06-0.64) |
| Lesotho | 0.42 (0.36-0.48) | 1.06 (0.94-1.19) | | 0.08 (0.06-0.09) | 0.08 (0.07-0.1) | 0.05 (0.02-0.12) |
| Lithuania | 0.44 (0.38-0.52) | 0.45 (0.39-0.51) | | 0.15 (0.06-0.44) | 0.12 (0.05-0.31) | 0.35 (0.23-0.53) |
| Luxembourg | 0.81 (0.61-1.05) | 0.52 (0.42-0.65) | | 0.14 (0.05-0.43) | 0.06 (0.02-0.21) | 0 (0-0.01) |
| Latvia | 0.38 (0.33-0.43) | 0.8 (0.71-0.91) | | 0.18 (0.08-0.48) | 0.05 (0.02-0.11) | 0.25 (0.17-0.38) |
| Morocco | 0.51 (0.42-0.62) | 0.58 (0.49-0.68) | | 0.14 (0.05-0.42) | 0.17 (0.07-0.48) | 0.28 (0.12-0.71) |
| Moldova | 0.63 (0.36-1.1) | 0.31 (0.17-0.58) | | 0.16 (0.07-0.43) | 0.54 (0.26-1.26) | 0.54 (0.24-1.25) |
| Madagascar | 0.56 (0.5-0.63) | 0.96 (0.87-1.05) | | 0.11 (0.1-0.13) | 0.04 (0.04-0.05) | 0.02 (0.01-0.06) |
| Maldives | 1.62 (1.38-1.91) | 0.65 (0.56-0.75) | | 0.21 (0.17-0.26) | 0.1 (0.08-0.12) | 0.31 (0.1-0.93) |
| Mexico | 1.13 (1.03-1.24) | 1.18 (1.1-1.28) | | 0.86 (0.78-0.94) | 0.23 (0.21-0.26) | 0.03 (0.03-0.04) |
| Marshall Islands | 1.11 (0.84-1.52) | 1.36 (0.98-1.94) | | 0.09 (0.04-0.25) | 0.31 (0.13-0.77) | 0.08 (0.03-0.2) |
| North Macedonia | 0.59 (0.48-0.72) | 0.53 (0.44-0.63) | | 0.11 (0.05-0.28) | 0.1 (0.05-0.24) | 0.4 (0.18-0.94) |
| Mali | 0.45 (0.4-0.51) | 0.54 (0.49-0.6) | | 0.11 (0.09-0.12) | 0.09 (0.07-0.1) | 0.39 (0.13-1.15) |
| Malta | 1.22 (1.03-1.44) | 0.48 (0.42-0.55) | | 0.12 (0.04-0.39) | 0.02 (0.01-0.04) | 0.11 (0.05-0.22) |
| Myanmar (Burma) | 0.8 (0.69-0.92) | 1.27 (1.14-1.43) | | 0.12 (0.05-0.35) | 0.19 (0.08-0.51) | 0.1 (0.04-0.28) |
| Montenegro | 1.59 (0.95-2.69) | 0.48 (0.28-0.85) | | 0.14 (0.06-0.37) | 0.15 (0.06-0.4) | 0.39 (0.15-0.97) |
| Mongolia | 0.33 (0.28-0.4) | 0.69 (0.59-0.81) | | 0.06 (0.03-0.16) | 0.14 (0.06-0.35) | 0.06 (0.03-0.16) |
| Mozambique | 0.65 (0.57-0.74) | 0.81 (0.73-0.91) | | 0.15 (0.12-0.18) | 0.17 (0.14-0.21) | 0.32 (0.21-0.49) |
| Mauritania | 0.22 (0.18-0.26) | 1.28 (1.12-1.48) | | 0.06 (0.03-0.16) | 0.1 (0.05-0.24) | 0.05 (0.02-0.14) |
| Mauritius | 0.45 (0.39-0.52) | 0.95 (0.85-1.06) | | 0.1 (0.05-0.25) | 0.19 (0.13-0.28) | 0.24 (0.11-0.6) |
| Malawi | 0.71 (0.65-0.78) | 1.01 (0.94-1.08) | | 0.11 (0.1-0.13) | 0.14 (0.12-0.16) | 0.22 (0.09-0.62) |
| Malaysia | 1.07 (0.96-1.2) | 1.19 (1.09-1.31) | | 0.07 (0.05-0.09) | 0.21 (0.17-0.25) | 1.39 (1.04-1.81) |
| Namibia | 1.58 (1.43-1.73) | 0.86 (0.79-0.93) | | 0.1 (0.08-0.11) | 0.07 (0.06-0.08) | 0.23 (0.1-0.57) |
| Niger | 0.41 (0.36-0.48) | 0.6 (0.53-0.68) | | 0.67 (0.56-0.8) | 0.17 (0.14-0.2) | 0.46 (0.17-1.41) |
| Nigeria | 0.22 (0.2-0.25) | 0.54 (0.5-0.6) | | 0.08 (0.07-0.09) | 0.11 (0.1-0.13) | 0.31 (0.13-0.82) |
| Nicaragua | 1.18 (0.9-1.58) | 1.73 (1.29-2.35) | | 0.34 (0.14-0.89) | 0.15 (0.07-0.38) | 0.1 (0.04-0.24) |
| Netherlands | 0.98 (0.89-1.07) | 0.64 (0.59-0.68) | | 0.02 (0.01-0.02) | 0.03 (0.03-0.04) | 0.07 (0.06-0.08) |
| Norway | 0.77 (0.67-0.89) | 0.73 (0.65-0.81) | | 0.22 (0.08-0.7) | 0.01 (0-0.02) | 0.06 (0.05-0.07) |
| Nepal | 0.27 (0.24-0.3) | 0.75 (0.69-0.81) | | 0.15 (0.13-0.17) | 0.22 (0.19-0.25) | 0.11 (0.03-0.37) |
| New Zealand | 1.45 (1.19-1.76) | 0.72 (0.61-0.85) | | 0.09 (0.03-0.31) | 0.38 (0.3-0.48) | 0.03 (0.03-0.04) |
| Oman | 0.65 (0.48-0.88) | 0.54 (0.43-0.7) | | 0.07 (0.03-0.21) | 0.19 (0.08-0.52) | 0.3 (0.12-0.78) |
| Pakistan | 0.25 (0.22-0.28) | 0.36 (0.32-0.39) | | 0.09 (0.08-0.11) | 0.04 (0.04-0.05) | 0.07 (0.02-0.22) |
| Panama | 0.98 (0.75-1.3) | 0.93 (0.7-1.23) | | 0.17 (0.08-0.41) | 0.36 (0.17-0.82) | 0.27 (0.12-0.66) |
| Peru | 1.72 (1.59-1.86) | 0.58 (0.54-0.63) | | 0.37 (0.33-0.42) | 0.19 (0.18-0.22) | 0.12 (0.05-0.31) |
| Philippines | 0.86 (0.79-0.93) | 0.82 (0.77-0.88) | | 0.13 (0.11-0.14) | 0.08 (0.07-0.09) | 0.01 (0.01-0.01) |
| Papua New Guinea | 0.04 (0.03-0.06) | 0.62 (0.51-0.76) | | 0.53 (0.18-1.65) | 0.04 (0.02-0.12) | 0 (0-0.01) |
| Poland | 1.5 (1.36-1.66) | 0.61 (0.56-0.68) | | 0.01 (0-0.01) | 0.01 (0.01-0.02) | 0.13 (0.1-0.18) |
| Portugal | 3.91 (3.56-4.31) | 0.85 (0.79-0.91) | | 0.11 (0.1-0.12) | 0.07 (0.06-0.08) | 0.05 (0.04-0.06) |
| Paraguay | 0.71 (0.61-0.84) | 0.42 (0.37-0.48) | | 0.19 (0.08-0.46) | 0.09 (0.04-0.21) | 0.1 (0.05-0.24) |
| Palestinian Territories | 0.6 (0.42-0.87) | 0.54 (0.39-0.72) | | 0.14 (0.06-0.4) | 0.18 (0.07-0.48) | 0.37 (0.16-0.93) |
| Qatar | 0.65 (0.44-0.94) | 0.57 (0.41-0.77) | | 0.12 (0.04-0.38) | 0.16 (0.06-0.5) | 0.33 (0.12-0.93) |
| Romania | 1.24 (1.04-1.47) | 1.23 (1.07-1.43) | | 0.05 (0.04-0.08) | 0.16 (0.12-0.22) | 0.39 (0.26-0.61) |
| Russia | 0.58 (0.52-0.65) | 0.63 (0.56-0.7) | | 0.1 (0.04-0.32) | 0.01 (0-0.01) | 0.59 (0.41-0.85) |
| Rwanda | 0.76 (0.67-0.87) | 1.61 (1.46-1.78) | | 0.2 (0.17-0.24) | 0.44 (0.39-0.51) | 0.19 (0.08-0.57) |
| Saudi Arabia | 0.78 (0.58-1.07) | 0.52 (0.37-0.74) | | 0.11 (0.04-0.35) | 0.25 (0.1-0.73) | 0.46 (0.19-1.15) |
| Sudan | 0.51 (0.35-0.74) | 0.75 (0.54-1.05) | | 0.06 (0.03-0.16) | 0.12 (0.05-0.29) | 0.41 (0.17-1.04) |
| Senegal | 0.33 (0.3-0.37) | 0.43 (0.39-0.47) | | 0.12 (0.1-0.13) | 0.1 (0.09-0.12) | 0.21 (0.09-0.59) |
| Singapore | 1.33 (1.11-1.61) | 1.06 (0.9-1.24) | | 0.09 (0.03-0.3) | 0.12 (0.09-0.16) | 0.06 (0.04-0.07) |
| Solomon Islands | 0.61 (0.49-0.78) | 1.36 (1.05-1.75) | | 0.11 (0.04-0.32) | 0.23 (0.1-0.58) | 0.03 (0.01-0.07) |
| Sierra Leone | 0.56 (0.49-0.64) | 0.68 (0.62-0.76) | | 0.05 (0.04-0.06) | 0.12 (0.1-0.14) | 0.07 (0.03-0.18) |
| El Salvador | 0.93 (0.64-1.37) | 0.78 (0.56-1.09) | | 0.28 (0.12-0.76) | 0.19 (0.08-0.49) | 0.05 (0.02-0.13) |
| Serbia | 0.95 (0.63-1.45) | 2.05 (1.36-3.12) | | 0.28 (0.12-0.72) | 0.16 (0.08-0.32) | 0.14 (0.06-0.34) |
| South Sudan | 0.41 (0.3-0.56) | 0.82 (0.58-1.22) | | 0.09 (0.04-0.26) | 0.15 (0.07-0.37) | 0.21 (0.09-0.59) |
| São Tomé and Príncipe | 0.81 (0.67-0.98) | 0.21 (0.18-0.25) | | 0.2 (0.16-0.24) | 0.05 (0.04-0.06) | 0.24 (0.1-0.73) |
| Suriname | 0.78 (0.54-1.13) | 1.11 (0.81-1.53) | | 0.19 (0.08-0.49) | 0.39 (0.19-0.95) | 0.26 (0.12-0.64) |
| Slovakia | 0.51 (0.45-0.59) | 0.35 (0.31-0.4) | | 0.01 (0-0.02) | 0.02 (0.01-0.04) | 0.12 (0.09-0.17) |
| Slovenia | 0.49 (0.42-0.58) | 0.39 (0.34-0.45) | | 0.39 (0.15-1.02) | 0.09 (0.04-0.21) | 0.34 (0.17-0.71) |
| Sweden | 1.06 (0.96-1.18) | 0.83 (0.76-0.9) | | 0.13 (0.11-0.15) | 0.1 (0.08-0.11) | 0.09 (0.07-0.1) |
| Eswatini | 0.57 (0.51-0.64) | 0.94 (0.8-1.08) | | 0.09 (0.08-0.11) | 0.06 (0.05-0.07) | 0.2 (0.14-0.29) |
| Seychelles | 0.65 (0.56-0.75) | 1.16 (1.03-1.32) | | 0.03 (0.02-0.04) | 0.1 (0.08-0.13) | 0.25 (0.11-0.65) |
| Syria | 0.55 (0.41-0.74) | 0.47 (0.37-0.6) | | 0.13 (0.05-0.39) | 0.17 (0.07-0.47) | 0.37 (0.15-0.9) |
| Chad | 0.27 (0.24-0.31) | 0.22 (0.19-0.24) | | 0.1 (0.08-0.12) | 0.04 (0.03-0.05) | 0.26 (0.11-0.69) |
| Togo | 0.34 (0.27-0.43) | 0.86 (0.72-1.04) | | 0.08 (0.06-0.1) | 0.08 (0.06-0.1) | 0.27 (0.12-0.65) |
| Thailand | 0.95 (0.71-1.26) | 1.23 (0.96-1.6) | | 0.08 (0.03-0.21) | 0.33 (0.13-0.8) | 0.03 (0.01-0.07) |
| Tajikistan | 0.65 (0.52-0.8) | 0.24 (0.19-0.3) | | 0.21 (0.17-0.26) | 0.32 (0.24-0.42) | 0.14 (0.06-0.37) |
| Turkmenistan | 0.47 (0.36-0.63) | 0.43 (0.32-0.59) | | 0.1 (0.04-0.28) | 0.17 (0.07-0.41) | 0.02 (0.01-0.06) |
| Timor-Leste | 0.53 (0.45-0.61) | 1.08 (0.97-1.22) | | 0.12 (0.1-0.15) | 0.07 (0.06-0.09) | 0.04 (0.02-0.09) |
| Tonga | 1.31 (1.05-1.66) | 1.06 (0.89-1.26) | | 0.08 (0.03-0.26) | 0.29 (0.12-0.85) | 0.07 (0.03-0.21) |
| Trinidad & Tobago | 0.51 (0.45-0.59) | 0.52 (0.45-0.59) | | 0.13 (0.1-0.17) | 0.22 (0.18-0.27) | 0.16 (0.07-0.35) |
| Tunisia | 0.33 (0.29-0.39) | 0.68 (0.59-0.77) | | 0.16 (0.06-0.48) | 0.07 (0.04-0.12) | 0.12 (0.09-0.17) |
| Turkey | 0.42 (0.36-0.48) | 0.38 (0.34-0.43) | | 0.14 (0.05-0.38) | 0.15 (0.1-0.23) | 0.21 (0.13-0.35) |
| Taiwan | 1.37 (1.11-1.68) | 1.95 (1.66-2.31) | | 0.1 (0.04-0.29) | 0.05 (0.04-0.06) | 0.06 (0.05-0.07) |
| Tanzania | 0.42 (0.37-0.47) | 0.99 (0.9-1.09) | | 0.09 (0.07-0.1) | 0.19 (0.17-0.22) | 0.47 (0.21-1.14) |
| Uganda | 0.36 (0.32-0.4) | 0.66 (0.6-0.72) | | 0.09 (0.08-0.11) | 0.26 (0.22-0.29) | 0.22 (0.09-0.6) |
| Ukraine | 0.6 (0.51-0.7) | 0.63 (0.55-0.72) | | 0.13 (0.05-0.38) | 0.04 (0.02-0.11) | 0.44 (0.18-1.14) |
| Uruguay | 0.72 (0.62-0.83) | 0.96 (0.86-1.07) | | 0.18 (0.07-0.55) | 0.24 (0.1-0.65) | 0.43 (0.17-1.18) |
| United States | 0.82 (0.77-0.88) | 0.72 (0.68-0.76) | | 0.08 (0.07-0.09) | 0.12 (0.11-0.13) | 0.19 (0.18-0.21) |
| Uzbekistan | 0.57 (0.46-0.71) | 0.87 (0.63-1.18) | | 0.28 (0.22-0.34) | 0.16 (0.07-0.4) | 0.09 (0.04-0.23) |
| St. Vincent & Grenadines | 1.08 (0.72-1.6) | 0.63 (0.44-0.85) | | 0.17 (0.07-0.41) | 0.25 (0.12-0.58) | 0.1 (0.05-0.24) |
| Venezuela | 0.82 (0.57-1.18) | 0.56 (0.4-0.76) | | 0.24 (0.1-0.66) | 0.21 (0.09-0.58) | 0.01 (0.01-0.04) |
| Vietnam | 0.83 (0.72-0.95) | 1.28 (1.15-1.43) | | 0.27 (0.18-0.42) | 0.8 (0.53-1.2) | 0.48 (0.21-1.15) |
| Vanuatu | 0.42 (0.33-0.53) | 0.55 (0.43-0.7) | | 0.1 (0.04-0.27) | 0.54 (0.23-1.34) | 0.09 (0.04-0.23) |
| Samoa | 1.43 (1.12-1.84) | 2.6 (2.12-3.17) | | 0.09 (0.03-0.25) | 2.2 (0.97-4.27) | 0.65 (0.28-1.48) |
| Yemen | 0.22 (0.19-0.26) | 0.23 (0.19-0.27) | | 0.15 (0.12-0.17) | 0.14 (0.12-0.17) | 0.06 (0.02-0.19) |
| South Africa | 0.2 (0.18-0.22) | 0.72 (0.66-0.78) | | 0.11 (0.05-0.27) | 0.17 (0.13-0.23) | 0.01 (0.01-0.01) |
| Zambia | 0.32 (0.29-0.36) | 1.15 (1.06-1.23) | | 0.07 (0.06-0.08) | 0.12 (0.1-0.14) | 0.58 (0.27-1.33) |
| Zimbabwe | 0.52 (0.48-0.57) | 0.82 (0.75-0.88) | | 0.11 (0.09-0.12) | 0.11 (0.09-0.12) | 0.09 (0.07-0.13) |

Table S19: National consumption (servings/day) in children 5-9 years in 2018.

| Country | Fruit | Vegetables (non-starchy) | Other non-potato starchy vegetables | Beans and legumes | Nuts and seeds |
| --- | --- | --- | --- | --- | --- |
| Afghanistan | 0.61 (0.42-0.91) | 0.67 (0.42-1.08) | 0.31 (0.1-1.14) | 0.7 (0.24-2.25) | 0.88 (0.27-2.4) |
| Angola | 1.06 (0.82-1.41) | 2.67 (1.98-3.53) | 0.27 (0.13-0.64) | 0.34 (0.17-0.75) | 0.37 (0.16-0.92) |
| Albania | 0.97 (0.82-1.14) | 0.5 (0.42-0.6) | 0.42 (0.32-0.57) | 0.29 (0.23-0.37) | 0.57 (0.26-1.2) |
| United Arab Emirates | 0.79 (0.66-0.93) | 0.67 (0.58-0.77) | 0.36 (0.13-0.99) | 0.3 (0.12-0.83) | 0.72 (0.29-1.65) |
| Argentina | 0.87 (0.77-1) | 0.96 (0.84-1.08) | 0.04 (0.03-0.06) | 0.03 (0.02-0.04) | 0.04 (0.03-0.05) |
| Armenia | 0.63 (0.52-0.77) | 0.37 (0.31-0.46) | 0.56 (0.45-0.72) | 0.11 (0.08-0.16) | 0.92 (0.56-1.54) |
| Antigua & Barbuda | 0.89 (0.61-1.28) | 1.78 (1.28-2.46) | 0.31 (0.13-0.81) | 0.36 (0.16-0.89) | 0.05 (0.02-0.13) |
| Australia | 0.87 (0.72-1.05) | 0.56 (0.48-0.66) | 0.05 (0.02-0.18) | 0.14 (0.1-0.19) | 0.17 (0.14-0.21) |
| Austria | 0.67 (0.59-0.76) | 0.55 (0.49-0.6) | 0.1 (0.04-0.27) | 0.04 (0.02-0.06) | 0.12 (0.1-0.15) |
| Azerbaijan | 0.74 (0.62-0.88) | 0.31 (0.26-0.38) | 0.26 (0.21-0.32) | 0.11 (0.08-0.15) | 1.45 (0.67-2.52) |
| Burundi | 0.66 (0.53-0.81) | 1.54 (1.3-1.82) | 0.21 (0.17-0.26) | 0.38 (0.31-0.48) | 0.2 (0.08-0.62) |
| Belgium | 0.67 (0.6-0.75) | 0.53 (0.47-0.6) | 0.07 (0.02-0.23) | 0.03 (0.02-0.04) | 0.08 (0.06-0.09) |
| Benin | 0.66 (0.58-0.74) | 0.8 (0.72-0.89) | 0.12 (0.1-0.14) | 0.23 (0.2-0.27) | 0.4 (0.17-1.06) |
| Burkina Faso | 0.31 (0.27-0.35) | 0.22 (0.2-0.25) | 0.03 (0.03-0.04) | 0.09 (0.07-0.1) | 0.72 (0.25-2.12) |
| Bangladesh | 0.5 (0.46-0.55) | 1.44 (1.34-1.55) | 0.09 (0.08-0.1) | 0.2 (0.18-0.22) | 0.02 (0.01-0.02) |
| Bulgaria | 0.66 (0.54-0.79) | 1.47 (1.27-1.69) | 0.02 (0.01-0.05) | 0.13 (0.09-0.18) | 0.63 (0.44-0.92) |
| Bahrain | 0.86 (0.62-1.2) | 1 (0.7-1.46) | 0.2 (0.07-0.6) | 0.29 (0.11-0.78) | 0.48 (0.19-1.2) |
| Bahamas | 0.77 (0.52-1.14) | 0.8 (0.58-1.13) | 0.31 (0.13-0.82) | 0.15 (0.06-0.38) | 0.2 (0.09-0.51) |
| Bosnia & Herzegovina | 4.12 (3.45-4.79) | 1.81 (1.57-2.08) | 0.22 (0.09-0.53) | 0.1 (0.05-0.23) | 0.44 (0.2-0.94) |
| Belarus | 1.69 (0.93-2.98) | 0.33 (0.18-0.58) | 0.24 (0.1-0.71) | 0.52 (0.23-1.31) | 0.52 (0.22-1.27) |
| Belize | 0.8 (0.55-1.16) | 1.17 (0.84-1.64) | 0.28 (0.11-0.78) | 0.22 (0.1-0.59) | 0.05 (0.02-0.12) |
| Bolivia | 0.81 (0.73-0.9) | 0.89 (0.8-0.99) | 0.47 (0.41-0.53) | 0.1 (0.09-0.12) | 0.26 (0.11-0.63) |
| Brazil | 0.79 (0.7-0.88) | 0.71 (0.65-0.78) | 0.06 (0.04-0.08) | 0.73 (0.61-0.84) | 0.01 (0-0.01) |
| Barbados | 1.18 (0.97-1.43) | 1.07 (0.9-1.26) | 0.27 (0.11-0.73) | 0.06 (0.04-0.1) | 0.19 (0.12-0.3) |
| Brunei | 0.86 (0.62-1.21) | 1.26 (0.96-1.67) | 0.09 (0.04-0.25) | 0.29 (0.13-0.69) | 0.1 (0.05-0.27) |
| Bhutan | 0.89 (0.66-1.2) | 1.88 (1.34-2.67) | 0.15 (0.05-0.47) | 0.46 (0.17-1.31) | 0.17 (0.05-0.53) |
| Botswana | 0.43 (0.33-0.56) | 0.87 (0.71-1.08) | 0.17 (0.09-0.39) | 0.16 (0.08-0.35) | 1.29 (0.61-2.27) |
| Central African Republic | 0.86 (0.65-1.13) | 0.83 (0.61-1.17) | 0.51 (0.42-0.62) | 0.32 (0.15-0.77) | 0.24 (0.1-0.61) |
| Canada | 0.88 (0.8-0.97) | 0.62 (0.58-0.66) | 0.05 (0.05-0.06) | 0.11 (0.09-0.13) | 0.39 (0.35-0.44) |
| Switzerland | 0.88 (0.76-1.03) | 0.53 (0.46-0.61) | 0.13 (0.05-0.38) | 0.02 (0.01-0.02) | 0.15 (0.07-0.34) |
| Chile | 1 (0.82-1.21) | 1.06 (0.9-1.26) | 0.55 (0.22-1.33) | 0.11 (0.07-0.2) | 0.37 (0.15-0.97) |
| China | 0.91 (0.82-1) | 2.31 (2.12-2.52) | 0.11 (0.05-0.28) | 0.13 (0.11-0.16) | 0.24 (0.19-0.29) |
| Côte d’lvoire | 0.53 (0.46-0.62) | 0.75 (0.65-0.86) | 0.16 (0.13-0.2) | 0.04 (0.03-0.06) | 0.29 (0.13-0.78) |
| Cameroon | 0.73 (0.64-0.83) | 1.01 (0.91-1.12) | 0.21 (0.18-0.24) | 0.28 (0.24-0.33) | 0.47 (0.2-1.09) |
| Congo - Kinshasa | 0.85 (0.75-0.96) | 1.76 (1.59-1.96) | 0.25 (0.21-0.29) | 0.29 (0.25-0.34) | 0.87 (0.38-1.98) |
| Congo - Brazzaville | 0.79 (0.7-0.89) | 1.14 (1.04-1.27) | 0.22 (0.18-0.26) | 0.21 (0.17-0.25) | 0.3 (0.13-0.76) |
| Colombia | 0.93 (0.85-1.01) | 0.74 (0.67-0.81) | 0.61 (0.54-0.69) | 0.23 (0.2-0.25) | 1.08 (0.5-2.08) |
| Comoros | 0.74 (0.64-0.86) | 0.77 (0.68-0.89) | 0.22 (0.18-0.26) | 0.09 (0.06-0.12) | 0.24 (0.1-0.61) |
| Cape Verde | 1.98 (1.42-2.72) | 1.48 (1.12-1.96) | 0.13 (0.05-0.34) | 0.25 (0.12-0.61) | 0.45 (0.19-1.19) |
| Costa Rica | 1.21 (0.85-1.75) | 1.27 (0.91-1.73) | 0.3 (0.13-0.79) | 0.23 (0.11-0.56) | 0.18 (0.08-0.48) |
| Cuba | 1.18 (0.89-1.59) | 0.85 (0.62-1.16) | 0.37 (0.16-0.96) | 0.22 (0.1-0.54) | 0.01 (0-0.02) |
| Cyprus | 0.4 (0.24-0.65) | 0.57 (0.35-0.94) | 0.04 (0.02-0.13) | 0.18 (0.07-0.46) | 0.1 (0.04-0.23) |
| Czechia | 0.51 (0.45-0.58) | 0.39 (0.35-0.43) | 0.22 (0.09-0.61) | 0.08 (0.04-0.21) | 0.16 (0.11-0.23) |
| Germany | 0.88 (0.8-0.97) | 0.88 (0.82-0.95) | 0.1 (0.09-0.12) | 0.03 (0.03-0.03) | 0.07 (0.06-0.08) |
| Djibouti | 1.21 (0.9-1.69) | 0.8 (0.56-1.18) | 0.11 (0.05-0.34) | 1.79 (0.88-3.34) | 0 (0-0.01) |
| Dominica | 0.98 (0.78-1.24) | 0.79 (0.65-0.98) | 0.35 (0.15-0.86) | 0.25 (0.12-0.61) | 0.09 (0.04-0.24) |
| Denmark | 0.58 (0.49-0.69) | 0.57 (0.5-0.66) | 0.08 (0.03-0.28) | 0.12 (0.05-0.33) | 0.19 (0.13-0.26) |
| Dominican Republic | 1.34 (1.21-1.47) | 0.81 (0.73-0.9) | 0.36 (0.32-0.41) | 0.4 (0.35-0.46) | 0.03 (0.01-0.08) |
| Algeria | 0.6 (0.47-0.76) | 1.12 (0.93-1.36) | 0.26 (0.1-0.74) | 0.16 (0.05-0.48) | 0.13 (0.06-0.34) |
| Ecuador | 0.24 (0.21-0.28) | 0.85 (0.75-0.97) | 0.18 (0.08-0.39) | 0.14 (0.08-0.24) | 0.06 (0.03-0.14) |
| Egypt | 0.67 (0.61-0.74) | 0.88 (0.8-0.96) | 0.19 (0.16-0.21) | 0.07 (0.06-0.09) | 0.05 (0.04-0.07) |
| Eritrea | 0.5 (0.36-0.69) | 0.55 (0.44-0.7) | 0.13 (0.06-0.33) | 0.24 (0.12-0.56) | 0.31 (0.13-0.78) |
| Spain | 0.41 (0.36-0.46) | 0.47 (0.42-0.52) | 0.04 (0.02-0.12) | 0.12 (0.06-0.26) | 0.19 (0.14-0.25) |
| Estonia | 0.72 (0.65-0.8) | 1.09 (1-1.2) | 0.02 (0.01-0.03) | 0.04 (0.03-0.06) | 0.43 (0.32-0.58) |
| Ethiopia | 0.33 (0.3-0.38) | 0.71 (0.64-0.78) | 0.12 (0.11-0.14) | 0.31 (0.27-0.36) | 0.06 (0.04-0.08) |
| Finland | 0.96 (0.86-1.07) | 0.58 (0.53-0.64) | 0.03 (0.01-0.07) | 0.04 (0.03-0.05) | 0.07 (0.06-0.08) |
| Fiji | 1.13 (0.77-1.65) | 1.67 (1.21-2.27) | 0.14 (0.06-0.36) | 0.25 (0.12-0.59) | 0.11 (0.05-0.28) |
| France | 0.98 (0.89-1.08) | 0.74 (0.68-0.8) | 0 (0-0.01) | 0.08 (0.07-0.09) | 0.06 (0.05-0.07) |
| Micronesia (Federated States of) | 1.5 (1.09-2.09) | 1.11 (0.75-1.65) | 0.13 (0.06-0.38) | 0.24 (0.11-0.59) | 0.08 (0.03-0.18) |
| Gabon | 0.61 (0.52-0.7) | 0.97 (0.86-1.11) | 0.27 (0.22-0.32) | 0.02 (0.02-0.03) | 0.62 (0.26-1.5) |
| United Kingdom | 0.59 (0.54-0.65) | 0.56 (0.51-0.61) | 0.06 (0.02-0.18) | 0.23 (0.19-0.29) | 0.12 (0.1-0.15) |
| Georgia | 0.5 (0.43-0.59) | 0.34 (0.29-0.39) | 0.14 (0.06-0.38) | 0.24 (0.1-0.61) | 0.29 (0.13-0.73) |
| Ghana | 0.76 (0.69-0.83) | 1.09 (1.01-1.17) | 0.16 (0.14-0.19) | 0.18 (0.15-0.21) | 0.95 (0.52-1.69) |
| Guinea | 0.19 (0.16-0.23) | 0.16 (0.13-0.2) | 0.08 (0.07-0.1) | 0.03 (0.02-0.04) | 0.33 (0.14-0.84) |
| Gambia | 0.27 (0.21-0.35) | 0.39 (0.31-0.49) | 0.07 (0.06-0.09) | 0.07 (0.05-0.09) | 0.36 (0.14-1.11) |
| Guinea-Bissau | 0.59 (0.45-0.79) | 1.1 (0.8-1.54) | 0.15 (0.07-0.4) | 0.81 (0.38-1.75) | 0.4 (0.17-1.01) |
| Equatorial Guinea | 0.78 (0.6-1.04) | 1.15 (0.84-1.61) | 0.23 (0.11-0.56) | 0.33 (0.16-0.74) | 0.35 (0.14-0.93) |
| Greece | 0.48 (0.42-0.56) | 0.42 (0.36-0.48) | 0.05 (0.02-0.14) | 0.08 (0.06-0.11) | 0.16 (0.13-0.21) |
| Grenada | 1.13 (0.78-1.64) | 1.94 (1.4-2.7) | 0.35 (0.16-0.83) | 0.24 (0.11-0.58) | 0 (0-0) |
| Guatemala | 0.71 (0.64-0.79) | 0.74 (0.68-0.81) | 0.24 (0.21-0.28) | 0.41 (0.35-0.47) | 0.06 (0.03-0.14) |
| Guyana | 1.89 (1.62-2.23) | 1.14 (0.99-1.31) | 0.3 (0.25-0.36) | 0.19 (0.15-0.23) | 0.18 (0.08-0.43) |
| Honduras | 0.83 (0.74-0.94) | 0.23 (0.21-0.27) | 0.22 (0.19-0.26) | 0.36 (0.31-0.42) | 0.11 (0.07-0.15) |
| Croatia | 0.41 (0.35-0.47) | 2.74 (2.43-3.08) | 0.25 (0.11-0.64) | 0.06 (0.02-0.15) | 0.48 (0.2-1.1) |
| Haiti | 0.66 (0.58-0.75) | 0.64 (0.57-0.72) | 0.15 (0.13-0.18) | 0.24 (0.21-0.28) | 0.12 (0.08-0.17) |
| Hungary | 0.41 (0.36-0.46) | 0.23 (0.2-0.25) | 0.2 (0.08-0.53) | 0.12 (0.04-0.36) | 0.09 (0.07-0.11) |
| Indonesia | 0.69 (0.64-0.75) | 1.26 (1.17-1.35) | 0.22 (0.19-0.25) | 0.25 (0.22-0.28) | 0.14 (0.11-0.17) |
| India | 0.33 (0.3-0.36) | 1.07 (1-1.15) | 0.1 (0.09-0.12) | 0.17 (0.15-0.19) | 0.09 (0.07-0.11) |
| Ireland | 0.52 (0.43-0.63) | 0.57 (0.49-0.66) | 0.07 (0.03-0.2) | 0.13 (0.06-0.29) | 0.3 (0.15-0.6) |
| Iran | 1.22 (1.12-1.34) | 1.26 (1.15-1.37) | 0.06 (0.05-0.08) | 0.13 (0.1-0.15) | 0.17 (0.14-0.21) |
| Iraq | 0.8 (0.56-1.16) | 0.72 (0.53-0.97) | 0.22 (0.08-0.6) | 0.28 (0.11-0.76) | 0.32 (0.13-0.81) |
| Iceland | 0.51 (0.44-0.58) | 0.34 (0.3-0.38) | 0.38 (0.14-1.13) | 0.03 (0.02-0.04) | 0.03 (0.02-0.04) |
| Israel | 0.97 (0.86-1.1) | 0.46 (0.42-0.51) | 0.35 (0.28-0.44) | 0.57 (0.46-0.71) | 0.71 (0.59-0.86) |
| Italy | 1.25 (1.13-1.38) | 0.68 (0.62-0.74) | 0.06 (0.05-0.08) | 0.08 (0.07-0.09) | 0.07 (0.06-0.09) |
| Jamaica | 1.64 (1.27-2.1) | 1.14 (0.94-1.4) | 0.25 (0.11-0.61) | 0.1 (0.07-0.15) | 0.07 (0.05-0.1) |
| Jordan | 1.3 (1.16-1.44) | 0.69 (0.63-0.77) | 0.14 (0.12-0.17) | 0.21 (0.18-0.25) | 0.27 (0.2-0.37) |
| Japan | 1.04 (0.99-1.09) | 2.07 (2-2.14) | 0.05 (0.03-0.08) | 0.47 (0.41-0.56) | 0.06 (0.05-0.06) |
| Kazakhstan | 0.43 (0.37-0.5) | 0.66 (0.58-0.74) | 0.32 (0.26-0.4) | 0.05 (0.02-0.12) | 3.1 (2.15-3.72) |
| Kenya | 0.9 (0.82-0.99) | 1.96 (1.82-2.11) | 0.31 (0.27-0.36) | 0.26 (0.22-0.29) | 0.02 (0.01-0.03) |
| Kyrgyzstan | 0.56 (0.47-0.67) | 0.17 (0.13-0.23) | 0.32 (0.26-0.39) | 0.05 (0.03-0.07) | 1.58 (0.78-2.64) |
| Cambodia | 0.51 (0.45-0.57) | 0.94 (0.85-1.03) | 0.14 (0.12-0.16) | 0.03 (0.03-0.04) | 0.04 (0.03-0.06) |
| Kiribati | 1.02 (0.68-1.52) | 0.72 (0.5-1.02) | 0.14 (0.06-0.38) | 0.26 (0.11-0.62) | 0.08 (0.04-0.2) |
| South Korea | 0.96 (0.9-1.03) | 1.02 (0.97-1.07) | 0.04 (0.03-0.04) | 0.24 (0.21-0.26) | 0.04 (0.03-0.04) |
| Kuwait | 0.42 (0.35-0.49) | 0.6 (0.52-0.69) | 0.13 (0.04-0.42) | 0.34 (0.12-1.03) | 0.57 (0.21-1.48) |
| Laos | 1.23 (1.1-1.39) | 1.13 (1.02-1.24) | 0.06 (0.04-0.08) | 0.22 (0.09-0.55) | 0.02 (0.02-0.02) |
| Lebanon | 1.65 (1.44-1.9) | 1.95 (1.73-2.2) | 0.11 (0.09-0.15) | 0.22 (0.18-0.27) | 1.08 (0.88-1.33) |
| Liberia | 0.43 (0.36-0.52) | 0.97 (0.85-1.11) | 0.17 (0.14-0.21) | 0.09 (0.07-0.11) | 0.08 (0.03-0.21) |
| Libya | 0.39 (0.3-0.49) | 0.64 (0.53-0.77) | 0.19 (0.08-0.55) | 0.28 (0.11-0.78) | 0.46 (0.19-1.09) |
| St. Lucia | 0.7 (0.49-1.02) | 0.69 (0.5-0.95) | 0.43 (0.19-1.1) | 0.47 (0.21-1.13) | 0.01 (0-0.03) |
| Sri Lanka | 0.73 (0.64-0.83) | 1.25 (1.13-1.39) | 0.31 (0.11-0.94) | 0.57 (0.47-0.68) | 0.24 (0.08-0.76) |
| Lesotho | 0.58 (0.5-0.68) | 1.47 (1.31-1.66) | 0.13 (0.1-0.15) | 0.13 (0.11-0.16) | 0.05 (0.02-0.14) |
| Lithuania | 0.47 (0.41-0.55) | 0.48 (0.42-0.54) | 0.24 (0.1-0.66) | 0.18 (0.08-0.46) | 0.51 (0.34-0.75) |
| Luxembourg | 0.56 (0.43-0.73) | 0.45 (0.36-0.56) | 0.09 (0.03-0.28) | 0.06 (0.02-0.19) | 0 (0-0.01) |
| Latvia | 0.41 (0.36-0.46) | 0.86 (0.78-0.95) | 0.28 (0.12-0.75) | 0.07 (0.03-0.17) | 0.37 (0.25-0.54) |
| Morocco | 0.7 (0.58-0.86) | 0.93 (0.8-1.08) | 0.22 (0.08-0.66) | 0.29 (0.11-0.81) | 0.36 (0.15-0.94) |
| Moldova | 0.67 (0.39-1.18) | 0.34 (0.18-0.62) | 0.26 (0.11-0.67) | 0.79 (0.35-1.75) | 0.78 (0.35-1.57) |
| Madagascar | 0.78 (0.7-0.87) | 1.33 (1.21-1.47) | 0.18 (0.16-0.21) | 0.07 (0.06-0.09) | 0.03 (0.01-0.07) |
| Maldives | 2.1 (1.79-2.49) | 1.08 (0.93-1.26) | 0.31 (0.25-0.38) | 0.19 (0.15-0.23) | 0.38 (0.12-1.12) |
| Mexico | 1.4 (1.27-1.53) | 1.51 (1.4-1.62) | 1.29 (1.17-1.42) | 0.36 (0.32-0.4) | 0.04 (0.04-0.05) |
| Marshall Islands | 1.14 (0.86-1.54) | 1.46 (1.06-2.03) | 0.12 (0.05-0.33) | 0.34 (0.15-0.86) | 0.09 (0.04-0.22) |
| North Macedonia | 0.63 (0.52-0.76) | 0.57 (0.48-0.67) | 0.17 (0.07-0.45) | 0.15 (0.07-0.35) | 0.58 (0.25-1.24) |
| Mali | 0.63 (0.56-0.71) | 0.76 (0.68-0.84) | 0.17 (0.15-0.2) | 0.14 (0.12-0.17) | 0.43 (0.14-1.24) |
| Malta | 0.85 (0.72-1) | 0.42 (0.36-0.48) | 0.08 (0.03-0.25) | 0.02 (0.01-0.04) | 0.15 (0.07-0.3) |
| Myanmar (Burma) | 0.82 (0.71-0.95) | 1.36 (1.21-1.52) | 0.16 (0.06-0.48) | 0.21 (0.09-0.56) | 0.12 (0.05-0.31) |
| Montenegro | 1.7 (1.02-2.89) | 0.52 (0.3-0.92) | 0.22 (0.09-0.58) | 0.22 (0.08-0.59) | 0.56 (0.22-1.36) |
| Mongolia | 0.36 (0.3-0.43) | 0.74 (0.64-0.86) | 0.1 (0.04-0.26) | 0.21 (0.09-0.52) | 0.09 (0.04-0.22) |
| Mozambique | 0.9 (0.79-1.03) | 1.13 (1.01-1.26) | 0.25 (0.2-0.3) | 0.29 (0.24-0.35) | 0.35 (0.23-0.53) |
| Mauritania | 0.3 (0.25-0.36) | 1.79 (1.57-2.05) | 0.1 (0.04-0.27) | 0.17 (0.08-0.39) | 0.06 (0.02-0.16) |
| Mauritius | 0.62 (0.54-0.72) | 1.32 (1.18-1.48) | 0.17 (0.08-0.42) | 0.32 (0.21-0.46) | 0.27 (0.12-0.66) |
| Malawi | 0.99 (0.9-1.09) | 1.41 (1.3-1.52) | 0.18 (0.16-0.21) | 0.23 (0.2-0.26) | 0.24 (0.1-0.68) |
| Malaysia | 1.1 (0.99-1.23) | 1.27 (1.16-1.39) | 0.09 (0.07-0.11) | 0.23 (0.19-0.27) | 1.55 (1.16-2.04) |
| Namibia | 2.2 (1.99-2.41) | 1.2 (1.1-1.3) | 0.16 (0.14-0.19) | 0.11 (0.1-0.14) | 0.26 (0.11-0.61) |
| Niger | 0.58 (0.5-0.67) | 0.84 (0.74-0.95) | 1.09 (0.91-1.31) | 0.28 (0.23-0.33) | 0.49 (0.19-1.46) |
| Nigeria | 0.31 (0.28-0.35) | 0.76 (0.69-0.84) | 0.13 (0.11-0.15) | 0.19 (0.16-0.21) | 0.34 (0.14-0.87) |
| Nicaragua | 1.45 (1.1-1.94) | 2.17 (1.65-2.93) | 0.5 (0.22-1.22) | 0.24 (0.11-0.58) | 0.13 (0.06-0.3) |
| Netherlands | 0.68 (0.63-0.74) | 0.55 (0.52-0.59) | 0.01 (0.01-0.01) | 0.03 (0.03-0.04) | 0.1 (0.09-0.11) |
| Norway | 0.54 (0.48-0.61) | 0.63 (0.56-0.7) | 0.14 (0.05-0.48) | 0.01 (0-0.02) | 0.08 (0.07-0.1) |
| Nepal | 0.34 (0.3-0.39) | 1.25 (1.15-1.36) | 0.22 (0.19-0.26) | 0.4 (0.35-0.47) | 0.13 (0.04-0.44) |
| New Zealand | 1.01 (0.83-1.23) | 0.62 (0.53-0.73) | 0.06 (0.02-0.2) | 0.35 (0.28-0.45) | 0.04 (0.03-0.06) |
| Oman | 0.9 (0.67-1.22) | 0.87 (0.69-1.12) | 0.12 (0.05-0.33) | 0.33 (0.13-0.87) | 0.4 (0.17-1) |
| Pakistan | 0.32 (0.28-0.36) | 0.59 (0.54-0.66) | 0.14 (0.11-0.17) | 0.08 (0.06-0.1) | 0.08 (0.03-0.27) |
| Panama | 1.22 (0.93-1.59) | 1.19 (0.9-1.61) | 0.26 (0.12-0.65) | 0.55 (0.26-1.2) | 0.35 (0.15-0.81) |
| Peru | 2.13 (1.95-2.32) | 0.74 (0.68-0.8) | 0.56 (0.49-0.63) | 0.3 (0.27-0.33) | 0.15 (0.06-0.4) |
| Philippines | 0.88 (0.81-0.95) | 0.88 (0.82-0.94) | 0.17 (0.14-0.19) | 0.08 (0.07-0.1) | 0.01 (0.01-0.01) |
| Papua New Guinea | 0.04 (0.03-0.06) | 0.67 (0.54-0.81) | 0.67 (0.25-1.77) | 0.04 (0.02-0.12) | 0 (0-0.01) |
| Poland | 1.61 (1.48-1.76) | 0.66 (0.62-0.71) | 0.01 (0.01-0.01) | 0.02 (0.02-0.03) | 0.19 (0.15-0.25) |
| Portugal | 2.73 (2.49-3) | 0.73 (0.68-0.79) | 0.07 (0.06-0.08) | 0.07 (0.06-0.08) | 0.07 (0.06-0.08) |
| Paraguay | 0.88 (0.75-1.03) | 0.53 (0.47-0.61) | 0.29 (0.12-0.67) | 0.14 (0.06-0.32) | 0.13 (0.06-0.31) |
| Palestinian Territories | 0.83 (0.58-1.19) | 0.86 (0.62-1.16) | 0.23 (0.09-0.67) | 0.29 (0.12-0.77) | 0.48 (0.2-1.18) |
| Qatar | 0.9 (0.61-1.3) | 0.91 (0.66-1.24) | 0.19 (0.06-0.61) | 0.27 (0.1-0.84) | 0.43 (0.17-1.18) |
| Romania | 1.33 (1.13-1.57) | 1.33 (1.17-1.5) | 0.09 (0.06-0.12) | 0.24 (0.18-0.32) | 0.57 (0.38-0.85) |
| Russia | 0.62 (0.56-0.69) | 0.68 (0.62-0.74) | 0.16 (0.07-0.5) | 0.01 (0-0.01) | 0.85 (0.61-1.2) |
| Rwanda | 1.06 (0.93-1.21) | 2.24 (2.02-2.49) | 0.33 (0.28-0.39) | 0.73 (0.64-0.85) | 0.21 (0.08-0.62) |
| Saudi Arabia | 1.08 (0.8-1.48) | 0.81 (0.6-1.16) | 0.18 (0.07-0.55) | 0.43 (0.17-1.1) | 0.6 (0.24-1.4) |
| Sudan | 0.72 (0.49-1.04) | 1.05 (0.75-1.46) | 0.1 (0.04-0.25) | 0.19 (0.09-0.46) | 0.44 (0.19-1.1) |
| Senegal | 0.46 (0.41-0.51) | 0.59 (0.54-0.65) | 0.19 (0.17-0.22) | 0.17 (0.15-0.2) | 0.23 (0.1-0.66) |
| Singapore | 1.36 (1.13-1.64) | 1.13 (0.95-1.32) | 0.12 (0.04-0.38) | 0.13 (0.1-0.17) | 0.06 (0.05-0.08) |
| Solomon Islands | 0.63 (0.5-0.79) | 1.45 (1.12-1.85) | 0.15 (0.06-0.42) | 0.26 (0.11-0.67) | 0.03 (0.01-0.07) |
| Sierra Leone | 0.77 (0.68-0.89) | 0.95 (0.85-1.06) | 0.09 (0.07-0.11) | 0.2 (0.17-0.24) | 0.08 (0.03-0.21) |
| El Salvador | 1.15 (0.79-1.68) | 0.99 (0.71-1.39) | 0.43 (0.17-1.06) | 0.29 (0.14-0.74) | 0.07 (0.03-0.17) |
| Serbia | 1.02 (0.67-1.54) | 2.2 (1.47-3.35) | 0.44 (0.19-1.02) | 0.23 (0.12-0.46) | 0.21 (0.09-0.49) |
| South Sudan | 0.57 (0.42-0.78) | 1.15 (0.81-1.71) | 0.15 (0.07-0.41) | 0.25 (0.11-0.63) | 0.24 (0.11-0.63) |
| São Tomé and Príncipe | 1.12 (0.94-1.36) | 0.29 (0.25-0.35) | 0.32 (0.27-0.39) | 0.08 (0.06-0.1) | 0.27 (0.11-0.78) |
| Suriname | 0.97 (0.67-1.4) | 1.41 (1.03-1.94) | 0.29 (0.13-0.71) | 0.61 (0.29-1.37) | 0.33 (0.15-0.79) |
| Slovakia | 0.55 (0.49-0.63) | 0.38 (0.34-0.42) | 0.02 (0.01-0.03) | 0.03 (0.02-0.05) | 0.17 (0.13-0.23) |
| Slovenia | 0.53 (0.45-0.61) | 0.42 (0.37-0.48) | 0.61 (0.25-1.36) | 0.14 (0.06-0.31) | 0.49 (0.24-1.01) |
| Sweden | 0.74 (0.68-0.82) | 0.71 (0.66-0.77) | 0.08 (0.07-0.1) | 0.09 (0.08-0.1) | 0.12 (0.1-0.13) |
| Eswatini | 0.79 (0.7-0.88) | 1.31 (1.13-1.5) | 0.15 (0.12-0.18) | 0.1 (0.08-0.12) | 0.23 (0.16-0.32) |
| Seychelles | 0.91 (0.78-1.05) | 1.62 (1.43-1.84) | 0.05 (0.04-0.06) | 0.17 (0.14-0.21) | 0.28 (0.12-0.74) |
| Syria | 0.76 (0.57-1.02) | 0.75 (0.6-0.95) | 0.22 (0.08-0.62) | 0.29 (0.12-0.79) | 0.48 (0.2-1.15) |
| Chad | 0.38 (0.33-0.43) | 0.3 (0.27-0.34) | 0.16 (0.14-0.19) | 0.07 (0.05-0.09) | 0.29 (0.13-0.75) |
| Togo | 0.48 (0.38-0.6) | 1.19 (1-1.45) | 0.13 (0.1-0.16) | 0.13 (0.1-0.16) | 0.29 (0.13-0.7) |
| Thailand | 0.98 (0.73-1.3) | 1.31 (1.02-1.7) | 0.1 (0.04-0.29) | 0.36 (0.15-0.89) | 0.03 (0.01-0.08) |
| Tajikistan | 0.69 (0.55-0.87) | 0.26 (0.2-0.33) | 0.33 (0.26-0.42) | 0.46 (0.34-0.64) | 0.2 (0.09-0.53) |
| Turkmenistan | 0.5 (0.38-0.66) | 0.47 (0.34-0.64) | 0.16 (0.07-0.42) | 0.24 (0.11-0.64) | 0.03 (0.01-0.08) |
| Timor-Leste | 0.54 (0.46-0.63) | 1.16 (1.03-1.31) | 0.16 (0.14-0.19) | 0.08 (0.07-0.1) | 0.04 (0.02-0.1) |
| Tonga | 1.34 (1.07-1.69) | 1.13 (0.95-1.34) | 0.11 (0.04-0.35) | 0.33 (0.13-0.89) | 0.08 (0.03-0.24) |
| Trinidad & Tobago | 0.63 (0.55-0.73) | 0.66 (0.58-0.75) | 0.2 (0.15-0.26) | 0.34 (0.27-0.41) | 0.21 (0.09-0.45) |
| Tunisia | 0.46 (0.4-0.53) | 1.08 (0.95-1.23) | 0.25 (0.1-0.69) | 0.12 (0.07-0.2) | 0.16 (0.11-0.23) |
| Turkey | 0.57 (0.5-0.66) | 0.62 (0.55-0.69) | 0.23 (0.09-0.6) | 0.25 (0.16-0.38) | 0.28 (0.17-0.45) |
| Taiwan | 1.4 (1.14-1.73) | 2.08 (1.78-2.46) | 0.13 (0.05-0.38) | 0.05 (0.04-0.07) | 0.07 (0.05-0.08) |
| Tanzania | 0.58 (0.52-0.65) | 1.38 (1.26-1.52) | 0.14 (0.12-0.16) | 0.32 (0.27-0.36) | 0.52 (0.22-1.21) |
| Uganda | 0.49 (0.44-0.55) | 0.91 (0.83-1) | 0.15 (0.13-0.17) | 0.42 (0.37-0.49) | 0.24 (0.1-0.63) |
| Ukraine | 0.64 (0.55-0.74) | 0.67 (0.6-0.76) | 0.2 (0.08-0.56) | 0.06 (0.03-0.15) | 0.62 (0.26-1.48) |
| Uruguay | 0.88 (0.77-1.02) | 1.22 (1.09-1.37) | 0.28 (0.11-0.82) | 0.36 (0.15-0.99) | 0.56 (0.23-1.36) |
| United States | 0.58 (0.54-0.61) | 0.62 (0.59-0.65) | 0.05 (0.05-0.06) | 0.11 (0.1-0.12) | 0.26 (0.24-0.28) |
| Uzbekistan | 0.61 (0.48-0.76) | 0.94 (0.7-1.29) | 0.43 (0.34-0.55) | 0.24 (0.11-0.64) | 0.13 (0.06-0.33) |
| St. Vincent & Grenadines | 1.33 (0.89-1.97) | 0.8 (0.57-1.09) | 0.26 (0.11-0.63) | 0.39 (0.18-0.88) | 0.13 (0.06-0.31) |
| Venezuela | 1.01 (0.7-1.45) | 0.71 (0.51-0.97) | 0.36 (0.14-1) | 0.33 (0.14-0.86) | 0.02 (0.01-0.05) |
| Vietnam | 0.85 (0.74-0.97) | 1.37 (1.23-1.52) | 0.35 (0.23-0.55) | 0.89 (0.59-1.33) | 0.54 (0.23-1.25) |
| Vanuatu | 0.43 (0.34-0.54) | 0.59 (0.46-0.75) | 0.13 (0.05-0.35) | 0.6 (0.25-1.43) | 0.11 (0.05-0.27) |
| Samoa | 1.46 (1.15-1.87) | 2.78 (2.26-3.38) | 0.11 (0.04-0.3) | 2.23 (1.06-3.77) | 0.72 (0.31-1.59) |
| Yemen | 0.31 (0.26-0.37) | 0.37 (0.3-0.44) | 0.23 (0.19-0.28) | 0.24 (0.19-0.3) | 0.08 (0.03-0.26) |
| South Africa | 0.27 (0.25-0.3) | 1 (0.91-1.09) | 0.18 (0.09-0.44) | 0.29 (0.21-0.38) | 0.01 (0.01-0.01) |
| Zambia | 0.45 (0.41-0.5) | 1.6 (1.47-1.72) | 0.11 (0.1-0.13) | 0.19 (0.16-0.23) | 0.63 (0.29-1.33) |
| Zimbabwe | 0.73 (0.66-0.8) | 1.14 (1.05-1.24) | 0.17 (0.15-0.2) | 0.17 (0.15-0.2) | 0.1 (0.07-0.15) |

Table S20: National consumption (servings/day) in children 10-14 years in 2018.

| Country | Fruit | Vegetables (non-starchy) | Other non-potato starchy vegetables | Beans and legumes | Nuts and seeds |
| --- | --- | --- | --- | --- | --- |
| Afghanistan | 0.77 (0.53-1.16) | 1.04 (0.65-1.71) | 0.42 (0.14-1.46) | 1.15 (0.39-3.02) | 1.02 (0.32-2.65) |
| Angola | 1.41 (1.07-1.86) | 3.55 (2.73-4.41) | 0.4 (0.19-0.92) | 0.49 (0.24-1.14) | 0.4 (0.17-1) |
| Albania | 1.17 (0.99-1.38) | 0.67 (0.56-0.81) | 0.62 (0.47-0.84) | 0.43 (0.33-0.55) | 0.7 (0.32-1.45) |
| United Arab Emirates | 1.06 (0.89-1.24) | 1.03 (0.9-1.18) | 0.52 (0.2-1.34) | 0.45 (0.17-1.3) | 0.88 (0.37-1.96) |
| Argentina | 1.07 (0.94-1.22) | 1.27 (1.11-1.43) | 0.06 (0.04-0.09) | 0.05 (0.03-0.06) | 0.05 (0.03-0.06) |
| Armenia | 0.76 (0.63-0.94) | 0.5 (0.41-0.63) | 0.82 (0.65-1.06) | 0.16 (0.11-0.24) | 1.11 (0.66-1.93) |
| Antigua & Barbuda | 1.09 (0.75-1.57) | 2.36 (1.7-3.25) | 0.44 (0.19-1.08) | 0.53 (0.24-1.28) | 0.07 (0.03-0.17) |
| Australia | 0.86 (0.71-1.03) | 0.66 (0.56-0.78) | 0.05 (0.02-0.19) | 0.17 (0.12-0.24) | 0.22 (0.17-0.27) |
| Austria | 0.66 (0.58-0.74) | 0.64 (0.58-0.71) | 0.1 (0.04-0.28) | 0.05 (0.03-0.07) | 0.15 (0.12-0.19) |
| Azerbaijan | 0.89 (0.75-1.06) | 0.42 (0.35-0.51) | 0.37 (0.3-0.46) | 0.16 (0.12-0.21) | 1.69 (0.81-2.72) |
| Burundi | 0.87 (0.7-1.08) | 2.15 (1.8-2.53) | 0.31 (0.25-0.38) | 0.57 (0.45-0.71) | 0.22 (0.09-0.66) |
| Belgium | 0.65 (0.59-0.73) | 0.63 (0.56-0.71) | 0.07 (0.02-0.25) | 0.03 (0.02-0.05) | 0.1 (0.08-0.12) |
| Benin | 0.87 (0.77-0.99) | 1.11 (1-1.24) | 0.17 (0.14-0.2) | 0.34 (0.29-0.39) | 0.44 (0.19-1.14) |
| Burkina Faso | 0.41 (0.36-0.47) | 0.31 (0.27-0.34) | 0.05 (0.04-0.06) | 0.13 (0.1-0.15) | 0.8 (0.28-2.34) |
| Bangladesh | 0.63 (0.57-0.7) | 2.23 (2.08-2.4) | 0.12 (0.1-0.14) | 0.33 (0.29-0.37) | 0.02 (0.02-0.03) |
| Bulgaria | 0.79 (0.66-0.95) | 1.97 (1.7-2.27) | 0.04 (0.02-0.07) | 0.19 (0.14-0.27) | 0.76 (0.51-1.15) |
| Bahrain | 1.16 (0.84-1.62) | 1.55 (1.09-2.25) | 0.28 (0.11-0.81) | 0.44 (0.17-1.19) | 0.61 (0.24-1.46) |
| Bahamas | 0.95 (0.64-1.4) | 1.06 (0.77-1.49) | 0.44 (0.18-1.14) | 0.21 (0.09-0.57) | 0.26 (0.11-0.68) |
| Bosnia & Herzegovina | 4.81 (4.17-5.19) | 2.43 (2.12-2.79) | 0.32 (0.14-0.76) | 0.14 (0.06-0.33) | 0.53 (0.24-1.15) |
| Belarus | 2.04 (1.12-3.6) | 0.44 (0.24-0.78) | 0.35 (0.14-0.96) | 0.76 (0.34-1.81) | 0.64 (0.27-1.46) |
| Belize | 0.98 (0.68-1.41) | 1.55 (1.1-2.17) | 0.37 (0.15-1.01) | 0.32 (0.14-0.88) | 0.06 (0.03-0.16) |
| Bolivia | 0.99 (0.89-1.11) | 1.18 (1.06-1.31) | 0.65 (0.57-0.75) | 0.14 (0.12-0.17) | 0.33 (0.14-0.79) |
| Brazil | 0.96 (0.86-1.07) | 0.94 (0.86-1.03) | 0.08 (0.06-0.11) | 1.06 (0.9-1.23) | 0.01 (0.01-0.01) |
| Barbados | 1.44 (1.19-1.75) | 1.41 (1.19-1.67) | 0.37 (0.16-0.97) | 0.09 (0.05-0.14) | 0.25 (0.16-0.38) |
| Brunei | 0.95 (0.68-1.34) | 1.54 (1.17-2.05) | 0.12 (0.05-0.33) | 0.36 (0.16-0.88) | 0.12 (0.05-0.3) |
| Bhutan | 1.13 (0.83-1.52) | 2.91 (2.07-4.13) | 0.2 (0.08-0.62) | 0.74 (0.28-1.98) | 0.19 (0.06-0.63) |
| Botswana | 0.57 (0.44-0.74) | 1.21 (0.99-1.5) | 0.26 (0.12-0.57) | 0.24 (0.12-0.52) | 1.42 (0.66-2.4) |
| Central African Republic | 1.14 (0.86-1.52) | 1.16 (0.84-1.64) | 0.75 (0.62-0.91) | 0.47 (0.23-1.12) | 0.27 (0.12-0.68) |
| Canada | 0.86 (0.79-0.95) | 0.73 (0.68-0.78) | 0.06 (0.05-0.07) | 0.13 (0.12-0.16) | 0.5 (0.45-0.56) |
| Switzerland | 0.86 (0.74-1) | 0.63 (0.55-0.72) | 0.14 (0.05-0.38) | 0.02 (0.01-0.03) | 0.19 (0.09-0.45) |
| Chile | 1.22 (1.01-1.48) | 1.4 (1.19-1.66) | 0.77 (0.31-1.76) | 0.17 (0.09-0.29) | 0.47 (0.19-1.15) |
| China | 1 (0.91-1.1) | 2.82 (2.59-3.08) | 0.15 (0.06-0.37) | 0.17 (0.13-0.21) | 0.27 (0.22-0.33) |
| Côte d’lvoire | 0.7 (0.6-0.82) | 1.04 (0.9-1.19) | 0.23 (0.18-0.29) | 0.06 (0.04-0.09) | 0.32 (0.14-0.87) |
| Cameroon | 0.97 (0.85-1.1) | 1.41 (1.26-1.56) | 0.31 (0.26-0.36) | 0.41 (0.35-0.49) | 0.52 (0.24-1.24) |
| Congo - Kinshasa | 1.12 (0.99-1.27) | 2.45 (2.21-2.73) | 0.37 (0.31-0.43) | 0.43 (0.36-0.5) | 0.97 (0.43-2.16) |
| Congo - Brazzaville | 1.04 (0.92-1.18) | 1.59 (1.44-1.76) | 0.32 (0.26-0.39) | 0.31 (0.26-0.38) | 0.34 (0.15-0.86) |
| Colombia | 1.14 (1.04-1.24) | 0.98 (0.89-1.07) | 0.86 (0.76-0.98) | 0.33 (0.29-0.37) | 1.33 (0.63-2.44) |
| Comoros | 0.98 (0.84-1.14) | 1.08 (0.95-1.24) | 0.32 (0.27-0.39) | 0.13 (0.09-0.18) | 0.27 (0.12-0.69) |
| Cape Verde | 2.63 (1.88-3.55) | 2.05 (1.56-2.72) | 0.19 (0.08-0.49) | 0.38 (0.18-0.92) | 0.5 (0.21-1.28) |
| Costa Rica | 1.49 (1.04-2.14) | 1.68 (1.21-2.3) | 0.42 (0.18-1.04) | 0.34 (0.15-0.84) | 0.23 (0.1-0.59) |
| Cuba | 1.45 (1.09-1.95) | 1.12 (0.83-1.54) | 0.52 (0.22-1.21) | 0.31 (0.14-0.78) | 0.01 (0-0.02) |
| Cyprus | 0.39 (0.24-0.63) | 0.67 (0.41-1.11) | 0.04 (0.02-0.12) | 0.22 (0.09-0.57) | 0.13 (0.06-0.3) |
| Czechia | 0.61 (0.54-0.7) | 0.52 (0.47-0.58) | 0.32 (0.14-0.87) | 0.12 (0.06-0.31) | 0.2 (0.14-0.28) |
| Germany | 0.86 (0.78-0.95) | 1.04 (0.96-1.12) | 0.11 (0.09-0.12) | 0.04 (0.03-0.04) | 0.09 (0.08-0.1) |
| Djibouti | 1.61 (1.18-2.24) | 1.1 (0.79-1.62) | 0.17 (0.07-0.5) | 2.45 (1.26-4.07) | 0 (0-0.01) |
| Dominica | 1.21 (0.95-1.52) | 1.05 (0.86-1.3) | 0.5 (0.21-1.15) | 0.37 (0.17-0.88) | 0.12 (0.05-0.3) |
| Denmark | 0.57 (0.48-0.67) | 0.67 (0.58-0.77) | 0.09 (0.03-0.28) | 0.15 (0.07-0.41) | 0.24 (0.17-0.33) |
| Dominican Republic | 1.64 (1.48-1.82) | 1.08 (0.97-1.19) | 0.51 (0.44-0.58) | 0.59 (0.51-0.67) | 0.04 (0.02-0.09) |
| Algeria | 0.8 (0.63-1.01) | 1.73 (1.44-2.08) | 0.37 (0.14-1.04) | 0.24 (0.08-0.75) | 0.17 (0.07-0.44) |
| Ecuador | 0.3 (0.26-0.35) | 1.13 (1-1.28) | 0.25 (0.11-0.55) | 0.2 (0.11-0.35) | 0.08 (0.04-0.18) |
| Egypt | 0.9 (0.81-1) | 1.35 (1.23-1.49) | 0.27 (0.23-0.31) | 0.11 (0.1-0.13) | 0.07 (0.05-0.09) |
| Eritrea | 0.66 (0.48-0.91) | 0.77 (0.61-0.98) | 0.19 (0.09-0.48) | 0.35 (0.17-0.84) | 0.35 (0.15-0.87) |
| Spain | 0.4 (0.35-0.45) | 0.55 (0.5-0.61) | 0.04 (0.02-0.13) | 0.15 (0.07-0.32) | 0.24 (0.18-0.32) |
| Estonia | 0.87 (0.78-0.97) | 1.47 (1.35-1.6) | 0.03 (0.02-0.04) | 0.06 (0.05-0.09) | 0.52 (0.39-0.7) |
| Ethiopia | 0.44 (0.4-0.5) | 0.99 (0.89-1.09) | 0.18 (0.16-0.21) | 0.46 (0.41-0.53) | 0.06 (0.04-0.09) |
| Finland | 0.94 (0.84-1.05) | 0.69 (0.63-0.76) | 0.03 (0.01-0.07) | 0.04 (0.03-0.06) | 0.09 (0.07-0.1) |
| Fiji | 1.25 (0.85-1.83) | 2.04 (1.49-2.78) | 0.19 (0.08-0.48) | 0.31 (0.14-0.72) | 0.13 (0.06-0.3) |
| France | 0.96 (0.87-1.05) | 0.87 (0.8-0.94) | 0 (0-0.01) | 0.1 (0.08-0.12) | 0.07 (0.06-0.08) |
| Micronesia (Federated States of) | 1.66 (1.2-2.31) | 1.35 (0.92-2.03) | 0.18 (0.08-0.46) | 0.29 (0.13-0.74) | 0.09 (0.04-0.22) |
| Gabon | 0.81 (0.69-0.93) | 1.36 (1.19-1.54) | 0.39 (0.33-0.46) | 0.04 (0.03-0.04) | 0.69 (0.29-1.65) |
| United Kingdom | 0.58 (0.53-0.64) | 0.66 (0.6-0.72) | 0.06 (0.02-0.2) | 0.29 (0.24-0.36) | 0.16 (0.13-0.19) |
| Georgia | 0.6 (0.52-0.71) | 0.45 (0.4-0.52) | 0.21 (0.09-0.55) | 0.35 (0.16-0.94) | 0.35 (0.16-0.9) |
| Ghana | 1.01 (0.92-1.1) | 1.51 (1.4-1.62) | 0.24 (0.21-0.28) | 0.26 (0.23-0.31) | 1.06 (0.58-1.88) |
| Guinea | 0.26 (0.21-0.31) | 0.23 (0.19-0.28) | 0.12 (0.1-0.14) | 0.04 (0.03-0.06) | 0.37 (0.16-0.92) |
| Gambia | 0.36 (0.28-0.46) | 0.55 (0.43-0.68) | 0.11 (0.08-0.14) | 0.1 (0.08-0.13) | 0.41 (0.16-1.14) |
| Guinea-Bissau | 0.78 (0.59-1.02) | 1.53 (1.12-2.16) | 0.23 (0.1-0.6) | 1.17 (0.57-2.37) | 0.44 (0.19-1.09) |
| Equatorial Guinea | 1.03 (0.79-1.39) | 1.61 (1.18-2.26) | 0.33 (0.16-0.79) | 0.48 (0.24-1.09) | 0.39 (0.16-0.98) |
| Greece | 0.47 (0.41-0.55) | 0.49 (0.43-0.56) | 0.05 (0.02-0.14) | 0.1 (0.07-0.13) | 0.21 (0.17-0.27) |
| Grenada | 1.38 (0.96-2) | 2.57 (1.85-3.56) | 0.5 (0.21-1.11) | 0.35 (0.16-0.84) | 0 (0-0) |
| Guatemala | 0.87 (0.78-0.97) | 0.98 (0.9-1.08) | 0.34 (0.29-0.39) | 0.59 (0.51-0.69) | 0.07 (0.03-0.18) |
| Guyana | 2.32 (1.98-2.73) | 1.51 (1.31-1.74) | 0.42 (0.36-0.51) | 0.27 (0.22-0.34) | 0.22 (0.1-0.54) |
| Honduras | 1.02 (0.9-1.16) | 0.31 (0.27-0.36) | 0.32 (0.27-0.37) | 0.53 (0.46-0.61) | 0.13 (0.09-0.19) |
| Croatia | 0.49 (0.43-0.57) | 3.68 (3.27-4.13) | 0.36 (0.15-0.85) | 0.09 (0.03-0.23) | 0.58 (0.25-1.33) |
| Haiti | 0.81 (0.71-0.92) | 0.85 (0.76-0.96) | 0.22 (0.19-0.25) | 0.35 (0.3-0.4) | 0.15 (0.11-0.22) |
| Hungary | 0.49 (0.44-0.56) | 0.31 (0.28-0.34) | 0.29 (0.13-0.74) | 0.17 (0.05-0.53) | 0.11 (0.08-0.14) |
| Indonesia | 0.76 (0.7-0.83) | 1.54 (1.42-1.66) | 0.3 (0.26-0.34) | 0.31 (0.27-0.35) | 0.16 (0.13-0.19) |
| India | 0.41 (0.38-0.46) | 1.66 (1.54-1.78) | 0.14 (0.12-0.17) | 0.28 (0.25-0.32) | 0.1 (0.08-0.13) |
| Ireland | 0.51 (0.42-0.62) | 0.67 (0.57-0.77) | 0.07 (0.03-0.21) | 0.16 (0.07-0.36) | 0.38 (0.19-0.77) |
| Iran | 1.64 (1.51-1.79) | 1.93 (1.77-2.1) | 0.09 (0.07-0.11) | 0.19 (0.16-0.23) | 0.22 (0.18-0.26) |
| Iraq | 1.08 (0.74-1.55) | 1.1 (0.81-1.49) | 0.31 (0.12-0.91) | 0.43 (0.18-1.17) | 0.4 (0.17-0.99) |
| Iceland | 0.5 (0.43-0.57) | 0.4 (0.35-0.45) | 0.39 (0.14-1.15) | 0.04 (0.03-0.05) | 0.04 (0.03-0.05) |
| Israel | 1.3 (1.16-1.46) | 0.71 (0.65-0.78) | 0.51 (0.41-0.64) | 0.89 (0.72-1.1) | 0.9 (0.75-1.07) |
| Italy | 1.22 (1.11-1.35) | 0.8 (0.72-0.87) | 0.07 (0.05-0.08) | 0.1 (0.08-0.11) | 0.09 (0.08-0.11) |
| Jamaica | 2.01 (1.56-2.58) | 1.51 (1.25-1.84) | 0.36 (0.15-0.82) | 0.15 (0.1-0.21) | 0.09 (0.06-0.13) |
| Jordan | 1.74 (1.55-1.94) | 1.07 (0.97-1.19) | 0.21 (0.17-0.24) | 0.32 (0.27-0.38) | 0.34 (0.25-0.47) |
| Japan | 1.15 (1.09-1.2) | 2.54 (2.46-2.63) | 0.07 (0.04-0.11) | 0.59 (0.51-0.7) | 0.06 (0.06-0.07) |
| Kazakhstan | 0.52 (0.44-0.61) | 0.89 (0.78-1) | 0.47 (0.38-0.59) | 0.07 (0.03-0.18) | 3.28 (2.36-3.79) |
| Kenya | 1.2 (1.08-1.32) | 2.72 (2.52-2.93) | 0.46 (0.4-0.53) | 0.38 (0.33-0.44) | 0.02 (0.01-0.03) |
| Kyrgyzstan | 0.67 (0.56-0.81) | 0.23 (0.17-0.31) | 0.46 (0.37-0.58) | 0.07 (0.05-0.11) | 1.84 (0.93-2.87) |
| Cambodia | 0.56 (0.5-0.63) | 1.15 (1.04-1.26) | 0.19 (0.16-0.22) | 0.04 (0.04-0.05) | 0.05 (0.03-0.07) |
| Kiribati | 1.12 (0.75-1.67) | 0.88 (0.62-1.26) | 0.2 (0.08-0.51) | 0.32 (0.14-0.75) | 0.1 (0.04-0.23) |
| South Korea | 1.06 (0.99-1.13) | 1.25 (1.19-1.31) | 0.05 (0.04-0.06) | 0.29 (0.27-0.32) | 0.04 (0.04-0.05) |
| Kuwait | 0.56 (0.46-0.66) | 0.92 (0.79-1.05) | 0.18 (0.06-0.59) | 0.53 (0.18-1.57) | 0.71 (0.26-1.73) |
| Laos | 1.36 (1.21-1.54) | 1.38 (1.26-1.52) | 0.08 (0.05-0.11) | 0.27 (0.12-0.69) | 0.02 (0.02-0.03) |
| Lebanon | 2.21 (1.93-2.54) | 3 (2.66-3.38) | 0.16 (0.13-0.21) | 0.34 (0.28-0.42) | 1.36 (1.11-1.66) |
| Liberia | 0.57 (0.47-0.68) | 1.35 (1.18-1.55) | 0.26 (0.21-0.31) | 0.13 (0.1-0.16) | 0.08 (0.04-0.22) |
| Libya | 0.52 (0.41-0.66) | 0.98 (0.81-1.19) | 0.28 (0.11-0.75) | 0.43 (0.18-1.12) | 0.58 (0.24-1.38) |
| St. Lucia | 0.86 (0.6-1.24) | 0.91 (0.66-1.25) | 0.61 (0.26-1.39) | 0.67 (0.29-1.63) | 0.01 (0.01-0.03) |
| Sri Lanka | 0.92 (0.81-1.05) | 1.94 (1.76-2.15) | 0.42 (0.15-1.14) | 0.93 (0.76-1.13) | 0.29 (0.09-0.89) |
| Lesotho | 0.77 (0.67-0.9) | 2.05 (1.82-2.32) | 0.18 (0.15-0.22) | 0.2 (0.17-0.24) | 0.06 (0.03-0.16) |
| Lithuania | 0.57 (0.49-0.66) | 0.65 (0.57-0.73) | 0.36 (0.15-0.89) | 0.26 (0.12-0.64) | 0.62 (0.42-0.9) |
| Luxembourg | 0.55 (0.42-0.72) | 0.53 (0.42-0.66) | 0.09 (0.03-0.29) | 0.07 (0.02-0.23) | 0 (0-0.01) |
| Latvia | 0.49 (0.44-0.55) | 1.16 (1.05-1.28) | 0.41 (0.17-0.99) | 0.11 (0.05-0.25) | 0.44 (0.31-0.65) |
| Morocco | 0.94 (0.77-1.15) | 1.43 (1.23-1.67) | 0.32 (0.12-0.93) | 0.44 (0.18-1.24) | 0.45 (0.18-1.16) |
| Moldova | 0.81 (0.46-1.42) | 0.45 (0.24-0.83) | 0.37 (0.16-0.92) | 1.16 (0.54-2.32) | 0.95 (0.43-1.83) |
| Madagascar | 1.03 (0.93-1.16) | 1.85 (1.68-2.04) | 0.27 (0.23-0.31) | 0.11 (0.09-0.13) | 0.03 (0.01-0.08) |
| Maldives | 2.65 (2.25-3.11) | 1.67 (1.43-1.96) | 0.41 (0.33-0.51) | 0.31 (0.25-0.38) | 0.45 (0.15-1.25) |
| Mexico | 1.71 (1.56-1.87) | 1.99 (1.86-2.14) | 1.81 (1.64-2) | 0.52 (0.46-0.59) | 0.05 (0.04-0.06) |
| Marshall Islands | 1.26 (0.93-1.72) | 1.79 (1.28-2.53) | 0.16 (0.06-0.44) | 0.43 (0.18-1.08) | 0.1 (0.04-0.27) |
| North Macedonia | 0.77 (0.63-0.92) | 0.76 (0.65-0.9) | 0.25 (0.11-0.64) | 0.21 (0.1-0.5) | 0.7 (0.31-1.42) |
| Mali | 0.83 (0.74-0.94) | 1.05 (0.94-1.17) | 0.26 (0.22-0.3) | 0.21 (0.18-0.25) | 0.48 (0.16-1.37) |
| Malta | 0.83 (0.71-0.98) | 0.49 (0.43-0.56) | 0.08 (0.03-0.26) | 0.03 (0.01-0.05) | 0.19 (0.09-0.38) |
| Myanmar (Burma) | 0.9 (0.78-1.04) | 1.66 (1.48-1.86) | 0.22 (0.09-0.63) | 0.26 (0.11-0.71) | 0.14 (0.06-0.34) |
| Montenegro | 2.05 (1.23-3.49) | 0.69 (0.4-1.24) | 0.32 (0.13-0.8) | 0.33 (0.12-0.87) | 0.68 (0.27-1.65) |
| Mongolia | 0.43 (0.36-0.52) | 1 (0.87-1.15) | 0.14 (0.06-0.39) | 0.31 (0.14-0.77) | 0.11 (0.05-0.27) |
| Mozambique | 1.2 (1.04-1.37) | 1.57 (1.41-1.76) | 0.36 (0.3-0.44) | 0.43 (0.35-0.52) | 0.39 (0.25-0.59) |
| Mauritania | 0.4 (0.34-0.48) | 2.49 (2.18-2.86) | 0.14 (0.06-0.38) | 0.25 (0.12-0.59) | 0.06 (0.03-0.18) |
| Mauritius | 0.83 (0.72-0.95) | 1.84 (1.64-2.06) | 0.24 (0.11-0.6) | 0.47 (0.32-0.69) | 0.3 (0.14-0.71) |
| Malawi | 1.32 (1.19-1.45) | 1.95 (1.81-2.11) | 0.27 (0.23-0.31) | 0.33 (0.29-0.39) | 0.26 (0.11-0.74) |
| Malaysia | 1.21 (1.09-1.35) | 1.55 (1.42-1.7) | 0.12 (0.09-0.15) | 0.28 (0.24-0.34) | 1.76 (1.32-2.32) |
| Namibia | 2.91 (2.65-3.19) | 1.66 (1.53-1.8) | 0.24 (0.2-0.28) | 0.17 (0.14-0.2) | 0.29 (0.13-0.67) |
| Niger | 0.77 (0.66-0.89) | 1.16 (1.03-1.32) | 1.61 (1.33-1.93) | 0.41 (0.34-0.49) | 0.55 (0.22-1.66) |
| Nigeria | 0.41 (0.37-0.46) | 1.05 (0.96-1.16) | 0.19 (0.16-0.22) | 0.28 (0.24-0.32) | 0.37 (0.16-0.95) |
| Nicaragua | 1.79 (1.36-2.36) | 2.83 (2.16-3.68) | 0.7 (0.3-1.57) | 0.34 (0.15-0.85) | 0.16 (0.07-0.37) |
| Netherlands | 0.67 (0.61-0.73) | 0.65 (0.61-0.69) | 0.01 (0.01-0.01) | 0.04 (0.03-0.05) | 0.13 (0.11-0.14) |
| Norway | 0.53 (0.47-0.6) | 0.74 (0.67-0.82) | 0.15 (0.05-0.46) | 0.01 (0-0.02) | 0.1 (0.08-0.12) |
| Nepal | 0.43 (0.38-0.49) | 1.94 (1.77-2.12) | 0.3 (0.25-0.35) | 0.67 (0.57-0.77) | 0.15 (0.05-0.52) |
| New Zealand | 0.99 (0.82-1.2) | 0.73 (0.63-0.86) | 0.06 (0.02-0.21) | 0.44 (0.35-0.56) | 0.06 (0.04-0.07) |
| Oman | 1.21 (0.9-1.63) | 1.35 (1.06-1.72) | 0.17 (0.06-0.51) | 0.49 (0.21-1.42) | 0.49 (0.2-1.29) |
| Pakistan | 0.4 (0.36-0.45) | 0.92 (0.83-1.02) | 0.18 (0.15-0.23) | 0.13 (0.11-0.16) | 0.1 (0.03-0.32) |
| Panama | 1.5 (1.13-1.96) | 1.56 (1.17-2.07) | 0.36 (0.16-0.85) | 0.79 (0.37-1.65) | 0.44 (0.19-1.01) |
| Peru | 2.61 (2.38-2.86) | 0.98 (0.9-1.07) | 0.78 (0.69-0.89) | 0.43 (0.39-0.49) | 0.19 (0.08-0.51) |
| Philippines | 0.97 (0.89-1.05) | 1.08 (1-1.15) | 0.22 (0.2-0.26) | 0.1 (0.09-0.12) | 0.01 (0.01-0.02) |
| Papua New Guinea | 0.04 (0.03-0.07) | 0.82 (0.67-1) | 0.89 (0.33-2.18) | 0.05 (0.02-0.16) | 0 (0-0.01) |
| Poland | 1.94 (1.79-2.11) | 0.89 (0.83-0.95) | 0.01 (0.01-0.02) | 0.03 (0.02-0.04) | 0.23 (0.18-0.3) |
| Portugal | 2.67 (2.44-2.93) | 0.86 (0.8-0.92) | 0.07 (0.07-0.08) | 0.08 (0.07-0.09) | 0.08 (0.07-0.1) |
| Paraguay | 1.08 (0.92-1.26) | 0.71 (0.62-0.8) | 0.41 (0.18-0.93) | 0.2 (0.09-0.48) | 0.17 (0.08-0.4) |
| Palestinian Territories | 1.11 (0.77-1.58) | 1.33 (0.96-1.78) | 0.33 (0.13-0.91) | 0.44 (0.18-1.21) | 0.61 (0.26-1.42) |
| Qatar | 1.21 (0.82-1.75) | 1.4 (1.02-1.91) | 0.27 (0.1-0.87) | 0.42 (0.15-1.28) | 0.54 (0.2-1.43) |
| Romania | 1.6 (1.36-1.89) | 1.78 (1.58-2.02) | 0.12 (0.09-0.17) | 0.35 (0.27-0.47) | 0.69 (0.46-1.02) |
| Russia | 0.75 (0.68-0.83) | 0.91 (0.84-1) | 0.24 (0.1-0.7) | 0.01 (0.01-0.02) | 1.04 (0.75-1.44) |
| Rwanda | 1.41 (1.23-1.61) | 3.12 (2.81-3.47) | 0.49 (0.41-0.57) | 1.09 (0.94-1.26) | 0.23 (0.09-0.66) |
| Saudi Arabia | 1.45 (1.07-1.96) | 1.26 (0.91-1.83) | 0.26 (0.09-0.77) | 0.65 (0.27-1.68) | 0.75 (0.31-1.67) |
| Sudan | 0.95 (0.65-1.38) | 1.45 (1.04-2.02) | 0.15 (0.07-0.37) | 0.29 (0.14-0.68) | 0.5 (0.22-1.2) |
| Senegal | 0.61 (0.55-0.68) | 0.82 (0.75-0.91) | 0.28 (0.24-0.33) | 0.25 (0.22-0.29) | 0.25 (0.11-0.73) |
| Singapore | 1.51 (1.25-1.81) | 1.38 (1.17-1.63) | 0.17 (0.06-0.56) | 0.17 (0.13-0.21) | 0.07 (0.06-0.09) |
| Solomon Islands | 0.69 (0.55-0.88) | 1.77 (1.37-2.26) | 0.2 (0.08-0.57) | 0.32 (0.14-0.81) | 0.03 (0.01-0.09) |
| Sierra Leone | 1.03 (0.91-1.17) | 1.32 (1.19-1.48) | 0.13 (0.11-0.16) | 0.3 (0.26-0.35) | 0.08 (0.04-0.24) |
| El Salvador | 1.4 (0.97-2.06) | 1.31 (0.94-1.85) | 0.58 (0.24-1.4) | 0.42 (0.18-1.05) | 0.09 (0.04-0.21) |
| Serbia | 1.23 (0.81-1.87) | 2.96 (1.98-4.47) | 0.64 (0.28-1.37) | 0.34 (0.17-0.68) | 0.25 (0.11-0.6) |
| South Sudan | 0.76 (0.57-1.04) | 1.6 (1.11-2.41) | 0.23 (0.1-0.61) | 0.36 (0.16-0.94) | 0.26 (0.11-0.7) |
| São Tomé and Príncipe | 1.49 (1.24-1.8) | 0.4 (0.34-0.48) | 0.47 (0.39-0.57) | 0.12 (0.09-0.15) | 0.3 (0.12-0.85) |
| Suriname | 1.19 (0.82-1.71) | 1.86 (1.37-2.56) | 0.41 (0.18-0.97) | 0.89 (0.41-1.83) | 0.42 (0.19-1.02) |
| Slovakia | 0.66 (0.59-0.75) | 0.5 (0.46-0.56) | 0.02 (0.01-0.04) | 0.05 (0.03-0.08) | 0.21 (0.16-0.28) |
| Slovenia | 0.63 (0.54-0.74) | 0.57 (0.5-0.64) | 0.88 (0.36-1.81) | 0.2 (0.09-0.46) | 0.59 (0.29-1.23) |
| Sweden | 0.73 (0.66-0.8) | 0.84 (0.78-0.91) | 0.09 (0.07-0.1) | 0.11 (0.09-0.13) | 0.15 (0.13-0.17) |
| Eswatini | 1.04 (0.93-1.17) | 1.81 (1.56-2.09) | 0.22 (0.18-0.26) | 0.14 (0.12-0.18) | 0.25 (0.17-0.36) |
| Seychelles | 1.21 (1.03-1.4) | 2.25 (1.99-2.56) | 0.07 (0.05-0.09) | 0.25 (0.21-0.31) | 0.31 (0.13-0.8) |
| Syria | 1.02 (0.77-1.36) | 1.16 (0.92-1.47) | 0.31 (0.12-0.85) | 0.44 (0.18-1.12) | 0.6 (0.26-1.38) |
| Chad | 0.5 (0.44-0.58) | 0.42 (0.38-0.47) | 0.24 (0.2-0.28) | 0.1 (0.08-0.14) | 0.32 (0.14-0.82) |
| Togo | 0.63 (0.5-0.79) | 1.66 (1.39-2) | 0.19 (0.15-0.24) | 0.19 (0.14-0.24) | 0.32 (0.15-0.75) |
| Thailand | 1.08 (0.81-1.43) | 1.61 (1.25-2.09) | 0.14 (0.06-0.38) | 0.45 (0.18-1.11) | 0.04 (0.02-0.09) |
| Tajikistan | 0.84 (0.66-1.06) | 0.34 (0.27-0.44) | 0.48 (0.38-0.61) | 0.68 (0.49-0.96) | 0.24 (0.11-0.65) |
| Turkmenistan | 0.6 (0.46-0.8) | 0.63 (0.46-0.85) | 0.23 (0.1-0.62) | 0.36 (0.16-0.88) | 0.04 (0.02-0.1) |
| Timor-Leste | 0.59 (0.51-0.69) | 1.42 (1.26-1.6) | 0.22 (0.18-0.26) | 0.1 (0.08-0.12) | 0.05 (0.02-0.12) |
| Tonga | 1.47 (1.19-1.87) | 1.38 (1.16-1.64) | 0.15 (0.06-0.45) | 0.4 (0.16-1.12) | 0.09 (0.04-0.27) |
| Trinidad & Tobago | 0.78 (0.67-0.9) | 0.87 (0.76-0.99) | 0.28 (0.21-0.37) | 0.49 (0.4-0.59) | 0.26 (0.12-0.58) |
| Tunisia | 0.61 (0.53-0.71) | 1.66 (1.47-1.88) | 0.37 (0.14-1.02) | 0.19 (0.12-0.31) | 0.2 (0.14-0.28) |
| Turkey | 0.77 (0.67-0.88) | 0.95 (0.84-1.05) | 0.33 (0.12-0.87) | 0.39 (0.25-0.58) | 0.35 (0.22-0.57) |
| Taiwan | 1.54 (1.25-1.9) | 2.54 (2.18-3) | 0.18 (0.07-0.49) | 0.07 (0.05-0.09) | 0.08 (0.06-0.09) |
| Tanzania | 0.77 (0.68-0.87) | 1.92 (1.75-2.12) | 0.2 (0.18-0.24) | 0.47 (0.41-0.54) | 0.57 (0.25-1.28) |
| Uganda | 0.66 (0.58-0.74) | 1.27 (1.15-1.39) | 0.22 (0.19-0.25) | 0.63 (0.55-0.72) | 0.27 (0.11-0.71) |
| Ukraine | 0.77 (0.67-0.89) | 0.91 (0.81-1.02) | 0.3 (0.12-0.83) | 0.09 (0.04-0.22) | 0.75 (0.33-1.63) |
| Uruguay | 1.09 (0.94-1.25) | 1.62 (1.44-1.81) | 0.39 (0.15-1.1) | 0.52 (0.21-1.39) | 0.7 (0.28-1.6) |
| United States | 0.56 (0.53-0.6) | 0.73 (0.7-0.76) | 0.05 (0.05-0.06) | 0.14 (0.13-0.16) | 0.34 (0.31-0.36) |
| Uzbekistan | 0.74 (0.58-0.93) | 1.26 (0.94-1.72) | 0.63 (0.49-0.81) | 0.36 (0.15-0.9) | 0.16 (0.07-0.39) |
| St. Vincent & Grenadines | 1.63 (1.09-2.41) | 1.06 (0.75-1.44) | 0.36 (0.16-0.87) | 0.56 (0.26-1.3) | 0.17 (0.08-0.39) |
| Venezuela | 1.24 (0.85-1.77) | 0.94 (0.68-1.28) | 0.5 (0.2-1.28) | 0.47 (0.2-1.24) | 0.02 (0.01-0.06) |
| Vietnam | 0.93 (0.81-1.07) | 1.67 (1.51-1.87) | 0.48 (0.31-0.74) | 1.11 (0.74-1.66) | 0.6 (0.26-1.36) |
| Vanuatu | 0.48 (0.38-0.59) | 0.72 (0.57-0.92) | 0.18 (0.07-0.48) | 0.74 (0.32-1.73) | 0.12 (0.05-0.31) |
| Samoa | 1.62 (1.26-2.07) | 3.4 (2.77-4.15) | 0.15 (0.06-0.42) | 2.61 (1.28-4.11) | 0.82 (0.37-1.73) |
| Yemen | 0.41 (0.34-0.5) | 0.56 (0.47-0.67) | 0.34 (0.28-0.41) | 0.37 (0.29-0.46) | 0.1 (0.04-0.32) |
| South Africa | 0.36 (0.33-0.4) | 1.4 (1.27-1.52) | 0.26 (0.12-0.68) | 0.42 (0.31-0.57) | 0.01 (0.01-0.01) |
| Zambia | 0.6 (0.54-0.66) | 2.22 (2.05-2.39) | 0.16 (0.14-0.19) | 0.28 (0.24-0.34) | 0.7 (0.33-1.45) |
| Zimbabwe | 0.97 (0.88-1.06) | 1.58 (1.45-1.72) | 0.26 (0.22-0.29) | 0.26 (0.22-0.3) | 0.11 (0.08-0.17) |

Table S21: National consumption (servings/day) in children 15-19 years in 2018.

| Country | Fruits | Vegetables (non-starchy) | Other non-potato starchy vegetables | Beans and legumes | Nuts and seeds |
| --- | --- | --- | --- | --- | --- |
| Afghanistan | 0.81 (0.56-1.24) | 1.24 (0.74-2.04) | 0.39 (0.13-1.35) | 1.33 (0.46-3.27) | 1.11 (0.34-2.78) |
| Angola | 1.54 (1.17-2) | 3.89 (3.01-4.69) | 0.41 (0.2-0.94) | 0.52 (0.25-1.12) | 0.43 (0.19-1.04) |
| Albania | 1.31 (1.11-1.56) | 0.86 (0.72-1.04) | 0.65 (0.49-0.87) | 0.48 (0.38-0.61) | 0.69 (0.31-1.46) |
| United Arab Emirates | 1.17 (0.99-1.37) | 1.24 (1.09-1.41) | 0.53 (0.2-1.34) | 0.5 (0.2-1.38) | 1.01 (0.43-2.16) |
| Argentina | 1.11 (0.98-1.26) | 1.39 (1.22-1.57) | 0.06 (0.04-0.09) | 0.05 (0.04-0.07) | 0.05 (0.04-0.07) |
| Armenia | 0.86 (0.71-1.05) | 0.64 (0.52-0.8) | 0.86 (0.67-1.11) | 0.18 (0.12-0.27) | 1.12 (0.66-1.95) |
| Antigua & Barbuda | 1.13 (0.77-1.63) | 2.59 (1.87-3.56) | 0.45 (0.18-1.05) | 0.56 (0.25-1.35) | 0.08 (0.04-0.2) |
| Australia | 0.91 (0.75-1.09) | 0.79 (0.67-0.93) | 0.05 (0.02-0.2) | 0.2 (0.14-0.28) | 0.25 (0.2-0.31) |
| Austria | 0.7 (0.62-0.79) | 0.77 (0.69-0.85) | 0.11 (0.04-0.29) | 0.05 (0.03-0.08) | 0.17 (0.14-0.22) |
| Azerbaijan | 1 (0.85-1.2) | 0.54 (0.45-0.65) | 0.39 (0.32-0.48) | 0.18 (0.14-0.24) | 1.71 (0.82-2.76) |
| Burundi | 0.95 (0.77-1.18) | 2.41 (2.02-2.84) | 0.32 (0.25-0.39) | 0.59 (0.47-0.73) | 0.23 (0.09-0.68) |
| Belgium | 0.7 (0.62-0.77) | 0.75 (0.66-0.84) | 0.08 (0.03-0.26) | 0.04 (0.03-0.06) | 0.11 (0.09-0.13) |
| Benin | 0.95 (0.85-1.07) | 1.25 (1.13-1.39) | 0.18 (0.15-0.21) | 0.35 (0.3-0.41) | 0.47 (0.2-1.2) |
| Burkina Faso | 0.44 (0.39-0.51) | 0.35 (0.31-0.39) | 0.05 (0.04-0.06) | 0.13 (0.11-0.16) | 0.84 (0.29-2.41) |
| Bangladesh | 0.67 (0.61-0.73) | 2.65 (2.49-2.84) | 0.11 (0.1-0.13) | 0.38 (0.34-0.42) | 0.02 (0.02-0.03) |
| Bulgaria | 0.89 (0.74-1.07) | 2.53 (2.18-2.91) | 0.04 (0.02-0.08) | 0.22 (0.15-0.3) | 0.76 (0.51-1.16) |
| Bahrain | 1.28 (0.95-1.8) | 1.87 (1.27-2.7) | 0.28 (0.11-0.84) | 0.49 (0.2-1.34) | 0.69 (0.27-1.61) |
| Bahamas | 0.98 (0.67-1.45) | 1.17 (0.84-1.64) | 0.45 (0.18-1.11) | 0.23 (0.1-0.59) | 0.31 (0.13-0.82) |
| Bosnia & Herzegovina | 5.1 (4.64-5.2) | 3.12 (2.73-3.56) | 0.33 (0.14-0.82) | 0.16 (0.07-0.38) | 0.53 (0.25-1.12) |
| Belarus | 2.3 (1.25-4.05) | 0.56 (0.31-1) | 0.37 (0.15-1.01) | 0.86 (0.36-1.98) | 0.63 (0.27-1.47) |
| Belize | 1.02 (0.7-1.47) | 1.71 (1.21-2.39) | 0.39 (0.15-1.06) | 0.35 (0.15-0.94) | 0.07 (0.03-0.19) |
| Bolivia | 1.03 (0.92-1.15) | 1.29 (1.17-1.43) | 0.66 (0.58-0.76) | 0.16 (0.13-0.18) | 0.39 (0.17-0.92) |
| Brazil | 1 (0.9-1.11) | 1.04 (0.95-1.13) | 0.08 (0.06-0.11) | 1.13 (0.96-1.31) | 0.01 (0.01-0.01) |
| Barbados | 1.5 (1.24-1.81) | 1.55 (1.31-1.83) | 0.39 (0.17-1.02) | 0.09 (0.06-0.15) | 0.3 (0.19-0.45) |
| Brunei | 0.94 (0.68-1.32) | 1.69 (1.29-2.24) | 0.13 (0.05-0.36) | 0.37 (0.17-0.92) | 0.13 (0.06-0.31) |
| Bhutan | 1.18 (0.88-1.59) | 3.46 (2.47-4.83) | 0.19 (0.07-0.57) | 0.86 (0.32-2.12) | 0.21 (0.07-0.66) |
| Botswana | 0.63 (0.48-0.81) | 1.36 (1.11-1.69) | 0.26 (0.13-0.6) | 0.25 (0.13-0.53) | 1.46 (0.71-2.47) |
| Central African Republic | 1.24 (0.95-1.66) | 1.3 (0.93-1.84) | 0.77 (0.63-0.94) | 0.49 (0.24-1.17) | 0.28 (0.13-0.73) |
| Canada | 0.91 (0.84-1) | 0.87 (0.81-0.93) | 0.06 (0.05-0.07) | 0.16 (0.14-0.19) | 0.57 (0.51-0.63) |
| Switzerland | 0.92 (0.79-1.06) | 0.75 (0.65-0.86) | 0.15 (0.06-0.41) | 0.02 (0.02-0.03) | 0.21 (0.1-0.5) |
| Chile | 1.27 (1.04-1.54) | 1.54 (1.3-1.83) | 0.79 (0.31-1.76) | 0.18 (0.1-0.31) | 0.56 (0.23-1.34) |
| China | 0.99 (0.9-1.09) | 3.09 (2.84-3.36) | 0.16 (0.07-0.41) | 0.17 (0.14-0.21) | 0.29 (0.24-0.35) |
| Côte d’lvoire | 0.77 (0.66-0.9) | 1.17 (1.01-1.34) | 0.24 (0.19-0.3) | 0.06 (0.05-0.09) | 0.34 (0.14-0.91) |
| Cameroon | 1.06 (0.93-1.19) | 1.58 (1.42-1.75) | 0.32 (0.27-0.37) | 0.43 (0.37-0.51) | 0.55 (0.24-1.25) |
| Congo - Kinshasa | 1.22 (1.08-1.39) | 2.75 (2.48-3.07) | 0.38 (0.32-0.44) | 0.45 (0.38-0.52) | 1.02 (0.45-2.25) |
| Congo - Brazzaville | 1.14 (1.01-1.28) | 1.78 (1.62-1.98) | 0.33 (0.27-0.4) | 0.32 (0.27-0.39) | 0.35 (0.16-0.88) |
| Colombia | 1.18 (1.08-1.29) | 1.08 (0.98-1.17) | 0.87 (0.77-0.99) | 0.35 (0.31-0.4) | 1.54 (0.71-2.64) |
| Comoros | 1.07 (0.92-1.24) | 1.21 (1.06-1.38) | 0.33 (0.27-0.4) | 0.14 (0.1-0.19) | 0.28 (0.12-0.69) |
| Cape Verde | 2.86 (2.06-3.85) | 2.3 (1.76-3.04) | 0.19 (0.09-0.49) | 0.39 (0.18-0.98) | 0.52 (0.22-1.34) |
| Costa Rica | 1.54 (1.08-2.22) | 1.85 (1.32-2.53) | 0.43 (0.18-1.06) | 0.36 (0.16-0.85) | 0.27 (0.11-0.67) |
| Cuba | 1.51 (1.14-2.02) | 1.24 (0.91-1.71) | 0.53 (0.22-1.25) | 0.34 (0.15-0.84) | 0.01 (0-0.03) |
| Cyprus | 0.41 (0.25-0.67) | 0.79 (0.48-1.32) | 0.05 (0.02-0.13) | 0.26 (0.1-0.67) | 0.15 (0.07-0.34) |
| Czechia | 0.69 (0.61-0.78) | 0.67 (0.6-0.74) | 0.34 (0.13-0.91) | 0.14 (0.06-0.37) | 0.2 (0.14-0.28) |
| Germany | 0.92 (0.84-1) | 1.24 (1.15-1.33) | 0.11 (0.1-0.13) | 0.04 (0.04-0.05) | 0.1 (0.09-0.12) |
| Djibouti | 1.74 (1.3-2.43) | 1.24 (0.89-1.8) | 0.17 (0.07-0.5) | 2.55 (1.31-4.09) | 0 (0-0.01) |
| Dominica | 1.25 (0.99-1.57) | 1.15 (0.95-1.43) | 0.5 (0.21-1.17) | 0.39 (0.18-0.95) | 0.14 (0.06-0.34) |
| Denmark | 0.61 (0.51-0.71) | 0.8 (0.7-0.92) | 0.09 (0.03-0.29) | 0.18 (0.08-0.49) | 0.27 (0.19-0.38) |
| Dominican Republic | 1.7 (1.54-1.88) | 1.18 (1.06-1.31) | 0.52 (0.45-0.59) | 0.63 (0.55-0.72) | 0.04 (0.02-0.12) |
| Algeria | 0.89 (0.71-1.12) | 2.09 (1.75-2.51) | 0.38 (0.15-1.05) | 0.27 (0.09-0.83) | 0.19 (0.08-0.49) |
| Ecuador | 0.31 (0.27-0.36) | 1.24 (1.1-1.41) | 0.25 (0.11-0.55) | 0.21 (0.12-0.38) | 0.09 (0.04-0.21) |
| Egypt | 1.01 (0.91-1.12) | 1.63 (1.49-1.81) | 0.28 (0.24-0.32) | 0.13 (0.11-0.15) | 0.08 (0.05-0.11) |
| Eritrea | 0.72 (0.52-0.99) | 0.86 (0.69-1.09) | 0.2 (0.09-0.47) | 0.36 (0.18-0.85) | 0.36 (0.16-0.9) |
| Spain | 0.42 (0.37-0.48) | 0.66 (0.6-0.72) | 0.05 (0.02-0.13) | 0.17 (0.08-0.38) | 0.27 (0.2-0.36) |
| Estonia | 0.98 (0.88-1.08) | 1.88 (1.73-2.06) | 0.03 (0.02-0.04) | 0.07 (0.05-0.1) | 0.52 (0.4-0.69) |
| Ethiopia | 0.48 (0.43-0.54) | 1.11 (1.01-1.22) | 0.19 (0.16-0.22) | 0.48 (0.42-0.55) | 0.07 (0.05-0.1) |
| Finland | 1 (0.89-1.11) | 0.82 (0.75-0.9) | 0.03 (0.01-0.08) | 0.05 (0.04-0.07) | 0.1 (0.08-0.12) |
| Fiji | 1.24 (0.84-1.8) | 2.24 (1.62-3.04) | 0.2 (0.08-0.52) | 0.32 (0.14-0.76) | 0.14 (0.06-0.33) |
| France | 1.02 (0.92-1.11) | 1.04 (0.96-1.12) | 0 (0-0.01) | 0.12 (0.1-0.14) | 0.08 (0.07-0.09) |
| Micronesia (Federated States of) | 1.64 (1.18-2.28) | 1.48 (1.01-2.2) | 0.19 (0.08-0.52) | 0.31 (0.13-0.79) | 0.09 (0.04-0.23) |
| Gabon | 0.88 (0.76-1.02) | 1.52 (1.33-1.73) | 0.4 (0.34-0.48) | 0.04 (0.03-0.05) | 0.73 (0.3-1.65) |
| United Kingdom | 0.62 (0.56-0.67) | 0.78 (0.72-0.85) | 0.07 (0.02-0.2) | 0.34 (0.28-0.42) | 0.18 (0.15-0.21) |
| Georgia | 0.68 (0.58-0.79) | 0.58 (0.51-0.67) | 0.22 (0.09-0.59) | 0.4 (0.17-1) | 0.36 (0.16-0.87) |
| Ghana | 1.1 (1.01-1.19) | 1.7 (1.58-1.82) | 0.25 (0.21-0.29) | 0.27 (0.24-0.32) | 1.12 (0.62-1.96) |
| Guinea | 0.28 (0.23-0.34) | 0.26 (0.21-0.31) | 0.12 (0.1-0.15) | 0.05 (0.04-0.06) | 0.39 (0.18-0.94) |
| Gambia | 0.39 (0.31-0.5) | 0.61 (0.48-0.76) | 0.11 (0.09-0.14) | 0.1 (0.08-0.13) | 0.42 (0.17-1.18) |
| Guinea-Bissau | 0.85 (0.65-1.12) | 1.73 (1.26-2.38) | 0.24 (0.11-0.62) | 1.21 (0.6-2.45) | 0.46 (0.2-1.13) |
| Equatorial Guinea | 1.13 (0.86-1.51) | 1.8 (1.32-2.53) | 0.34 (0.16-0.79) | 0.5 (0.24-1.15) | 0.41 (0.17-1.05) |
| Greece | 0.5 (0.44-0.58) | 0.59 (0.51-0.67) | 0.05 (0.02-0.17) | 0.12 (0.09-0.15) | 0.24 (0.19-0.3) |
| Grenada | 1.43 (0.99-2.09) | 2.83 (2.04-3.91) | 0.51 (0.22-1.12) | 0.37 (0.17-0.89) | 0 (0-0) |
| Guatemala | 0.9 (0.82-1) | 1.08 (0.99-1.18) | 0.34 (0.3-0.39) | 0.63 (0.55-0.73) | 0.09 (0.04-0.21) |
| Guyana | 2.41 (2.06-2.83) | 1.66 (1.45-1.9) | 0.43 (0.36-0.51) | 0.29 (0.24-0.36) | 0.27 (0.12-0.67) |
| Honduras | 1.06 (0.94-1.2) | 0.34 (0.3-0.39) | 0.32 (0.27-0.37) | 0.56 (0.49-0.65) | 0.16 (0.11-0.23) |
| Croatia | 0.55 (0.48-0.64) | 4.69 (4.21-5.09) | 0.38 (0.16-0.92) | 0.1 (0.04-0.25) | 0.58 (0.25-1.32) |
| Haiti | 0.84 (0.74-0.95) | 0.93 (0.83-1.05) | 0.22 (0.19-0.25) | 0.37 (0.32-0.43) | 0.18 (0.13-0.26) |
| Hungary | 0.55 (0.49-0.62) | 0.39 (0.35-0.43) | 0.3 (0.13-0.77) | 0.19 (0.06-0.59) | 0.11 (0.08-0.14) |
| Indonesia | 0.75 (0.69-0.82) | 1.68 (1.56-1.82) | 0.32 (0.27-0.36) | 0.32 (0.28-0.37) | 0.17 (0.14-0.21) |
| India | 0.44 (0.4-0.47) | 1.97 (1.84-2.11) | 0.13 (0.11-0.16) | 0.32 (0.29-0.37) | 0.11 (0.09-0.14) |
| Ireland | 0.54 (0.45-0.66) | 0.8 (0.68-0.92) | 0.08 (0.03-0.24) | 0.19 (0.08-0.43) | 0.43 (0.21-0.87) |
| Iran | 1.83 (1.69-1.98) | 2.34 (2.17-2.52) | 0.09 (0.07-0.11) | 0.22 (0.18-0.25) | 0.25 (0.21-0.29) |
| Iraq | 1.2 (0.83-1.71) | 1.33 (0.97-1.8) | 0.32 (0.13-0.91) | 0.49 (0.2-1.25) | 0.46 (0.19-1.15) |
| Iceland | 0.53 (0.46-0.61) | 0.47 (0.42-0.53) | 0.42 (0.15-1.27) | 0.05 (0.04-0.06) | 0.05 (0.03-0.06) |
| Israel | 1.45 (1.3-1.63) | 0.86 (0.79-0.94) | 0.52 (0.42-0.65) | 0.98 (0.81-1.2) | 1.03 (0.87-1.21) |
| Italy | 1.29 (1.18-1.43) | 0.95 (0.87-1.04) | 0.07 (0.06-0.09) | 0.12 (0.1-0.13) | 0.11 (0.09-0.12) |
| Jamaica | 2.08 (1.62-2.67) | 1.66 (1.37-2.03) | 0.36 (0.16-0.82) | 0.16 (0.11-0.23) | 0.11 (0.07-0.15) |
| Jordan | 1.94 (1.73-2.16) | 1.29 (1.17-1.43) | 0.21 (0.18-0.25) | 0.36 (0.31-0.42) | 0.39 (0.28-0.54) |
| Japan | 1.13 (1.09-1.18) | 2.78 (2.69-2.87) | 0.07 (0.05-0.12) | 0.61 (0.53-0.72) | 0.07 (0.07-0.07) |
| Kazakhstan | 0.58 (0.5-0.68) | 1.14 (1.01-1.28) | 0.49 (0.39-0.62) | 0.08 (0.04-0.2) | 3.29 (2.41-3.76) |
| Kenya | 1.31 (1.18-1.44) | 3.05 (2.84-3.29) | 0.47 (0.41-0.55) | 0.39 (0.34-0.45) | 0.02 (0.01-0.04) |
| Kyrgyzstan | 0.76 (0.62-0.91) | 0.29 (0.22-0.39) | 0.49 (0.39-0.6) | 0.08 (0.05-0.12) | 1.84 (0.93-2.89) |
| Cambodia | 0.55 (0.49-0.62) | 1.26 (1.14-1.39) | 0.2 (0.17-0.23) | 0.04 (0.04-0.05) | 0.05 (0.03-0.08) |
| Kiribati | 1.11 (0.75-1.65) | 0.96 (0.68-1.37) | 0.2 (0.08-0.53) | 0.33 (0.15-0.81) | 0.1 (0.05-0.25) |
| South Korea | 1.05 (0.99-1.11) | 1.37 (1.3-1.43) | 0.05 (0.05-0.06) | 0.3 (0.28-0.33) | 0.05 (0.04-0.05) |
| Kuwait | 0.62 (0.52-0.73) | 1.11 (0.96-1.27) | 0.19 (0.06-0.6) | 0.58 (0.21-1.66) | 0.83 (0.3-1.91) |
| Laos | 1.35 (1.2-1.52) | 1.51 (1.38-1.66) | 0.08 (0.06-0.12) | 0.28 (0.12-0.71) | 0.02 (0.02-0.03) |
| Lebanon | 2.47 (2.16-2.82) | 3.63 (3.23-4.07) | 0.17 (0.13-0.22) | 0.38 (0.31-0.46) | 1.55 (1.28-1.88) |
| Liberia | 0.63 (0.52-0.74) | 1.52 (1.33-1.74) | 0.26 (0.22-0.32) | 0.13 (0.11-0.16) | 0.09 (0.04-0.23) |
| Libya | 0.58 (0.46-0.74) | 1.18 (0.99-1.43) | 0.29 (0.11-0.8) | 0.49 (0.2-1.27) | 0.66 (0.27-1.53) |
| St. Lucia | 0.89 (0.62-1.29) | 1 (0.73-1.38) | 0.62 (0.25-1.45) | 0.72 (0.32-1.7) | 0.02 (0.01-0.04) |
| Sri Lanka | 0.97 (0.86-1.09) | 2.3 (2.1-2.53) | 0.41 (0.15-1.14) | 1.07 (0.9-1.29) | 0.31 (0.1-0.97) |
| Lesotho | 0.84 (0.73-0.98) | 2.3 (2.04-2.6) | 0.19 (0.16-0.23) | 0.21 (0.17-0.25) | 0.06 (0.03-0.16) |
| Lithuania | 0.64 (0.55-0.75) | 0.83 (0.73-0.93) | 0.37 (0.15-0.96) | 0.29 (0.13-0.75) | 0.62 (0.42-0.9) |
| Luxembourg | 0.58 (0.45-0.76) | 0.63 (0.51-0.79) | 0.1 (0.04-0.32) | 0.08 (0.03-0.28) | 0 (0-0.01) |
| Latvia | 0.55 (0.49-0.62) | 1.49 (1.35-1.64) | 0.43 (0.18-1.06) | 0.12 (0.05-0.27) | 0.45 (0.31-0.64) |
| Morocco | 1.05 (0.86-1.28) | 1.72 (1.49-2.01) | 0.32 (0.12-0.89) | 0.49 (0.2-1.37) | 0.52 (0.22-1.26) |
| Moldova | 0.91 (0.52-1.58) | 0.58 (0.31-1.07) | 0.39 (0.17-0.95) | 1.3 (0.6-2.52) | 0.95 (0.43-1.8) |
| Madagascar | 1.13 (1.01-1.26) | 2.08 (1.89-2.29) | 0.28 (0.24-0.32) | 0.11 (0.1-0.13) | 0.03 (0.01-0.08) |
| Maldives | 2.78 (2.36-3.24) | 2 (1.71-2.34) | 0.39 (0.32-0.48) | 0.36 (0.29-0.44) | 0.49 (0.16-1.37) |
| Mexico | 1.78 (1.63-1.93) | 2.19 (2.05-2.34) | 1.84 (1.67-2.01) | 0.56 (0.5-0.63) | 0.06 (0.05-0.08) |
| Marshall Islands | 1.25 (0.94-1.67) | 1.95 (1.41-2.78) | 0.17 (0.07-0.46) | 0.44 (0.19-1.14) | 0.11 (0.05-0.28) |
| North Macedonia | 0.86 (0.71-1.04) | 0.98 (0.83-1.15) | 0.26 (0.11-0.66) | 0.24 (0.11-0.59) | 0.7 (0.32-1.44) |
| Mali | 0.91 (0.81-1.02) | 1.18 (1.06-1.31) | 0.26 (0.22-0.31) | 0.22 (0.18-0.26) | 0.5 (0.17-1.43) |
| Malta | 0.88 (0.75-1.03) | 0.59 (0.51-0.67) | 0.09 (0.03-0.29) | 0.03 (0.02-0.06) | 0.21 (0.11-0.44) |
| Myanmar (Burma) | 0.89 (0.77-1.03) | 1.82 (1.63-2.04) | 0.23 (0.09-0.67) | 0.27 (0.11-0.74) | 0.14 (0.06-0.38) |
| Montenegro | 2.31 (1.38-3.93) | 0.89 (0.51-1.59) | 0.33 (0.15-0.83) | 0.37 (0.14-0.96) | 0.69 (0.27-1.65) |
| Mongolia | 0.49 (0.41-0.58) | 1.28 (1.11-1.47) | 0.15 (0.06-0.4) | 0.34 (0.15-0.88) | 0.11 (0.05-0.26) |
| Mozambique | 1.3 (1.14-1.49) | 1.76 (1.59-1.97) | 0.37 (0.31-0.45) | 0.44 (0.36-0.54) | 0.41 (0.27-0.62) |
| Mauritania | 0.44 (0.37-0.53) | 2.8 (2.45-3.2) | 0.14 (0.06-0.39) | 0.25 (0.12-0.6) | 0.06 (0.03-0.18) |
| Mauritius | 0.9 (0.79-1.04) | 2.07 (1.85-2.31) | 0.25 (0.12-0.6) | 0.49 (0.33-0.71) | 0.32 (0.14-0.72) |
| Malawi | 1.44 (1.3-1.58) | 2.19 (2.04-2.37) | 0.28 (0.24-0.32) | 0.35 (0.3-0.4) | 0.28 (0.11-0.76) |
| Malaysia | 1.2 (1.08-1.33) | 1.7 (1.56-1.85) | 0.13 (0.1-0.16) | 0.29 (0.25-0.35) | 1.89 (1.42-2.49) |
| Namibia | 3.17 (2.9-3.44) | 1.87 (1.72-2.02) | 0.24 (0.21-0.28) | 0.18 (0.15-0.21) | 0.3 (0.14-0.7) |
| Niger | 0.84 (0.72-0.97) | 1.3 (1.16-1.48) | 1.66 (1.37-1.98) | 0.42 (0.35-0.51) | 0.59 (0.23-1.81) |
| Nigeria | 0.45 (0.4-0.5) | 1.18 (1.08-1.31) | 0.19 (0.16-0.22) | 0.29 (0.25-0.33) | 0.4 (0.17-1.03) |
| Nicaragua | 1.84 (1.41-2.47) | 3.11 (2.35-3.99) | 0.72 (0.31-1.54) | 0.37 (0.17-0.88) | 0.19 (0.09-0.45) |
| Netherlands | 0.71 (0.65-0.77) | 0.77 (0.72-0.83) | 0.01 (0.01-0.02) | 0.05 (0.04-0.05) | 0.14 (0.13-0.16) |
| Norway | 0.56 (0.5-0.64) | 0.88 (0.79-0.97) | 0.16 (0.06-0.51) | 0.01 (0-0.02) | 0.12 (0.1-0.14) |
| Nepal | 0.46 (0.4-0.52) | 2.3 (2.12-2.51) | 0.28 (0.24-0.33) | 0.77 (0.67-0.88) | 0.17 (0.05-0.57) |
| New Zealand | 1.05 (0.87-1.27) | 0.87 (0.75-1.02) | 0.07 (0.03-0.22) | 0.52 (0.41-0.66) | 0.06 (0.05-0.08) |
| Oman | 1.35 (1.01-1.82) | 1.63 (1.28-2.11) | 0.17 (0.07-0.5) | 0.56 (0.22-1.49) | 0.56 (0.23-1.35) |
| Pakistan | 0.42 (0.38-0.47) | 1.09 (0.99-1.2) | 0.18 (0.14-0.22) | 0.15 (0.12-0.18) | 0.11 (0.03-0.36) |
| Panama | 1.54 (1.18-2.03) | 1.71 (1.3-2.28) | 0.36 (0.17-0.84) | 0.85 (0.4-1.77) | 0.52 (0.23-1.17) |
| Peru | 2.71 (2.47-2.97) | 1.08 (0.99-1.18) | 0.8 (0.7-0.91) | 0.46 (0.41-0.52) | 0.23 (0.1-0.61) |
| Philippines | 0.96 (0.88-1.04) | 1.18 (1.1-1.26) | 0.24 (0.21-0.27) | 0.11 (0.09-0.12) | 0.01 (0.01-0.02) |
| Papua New Guinea | 0.04 (0.03-0.06) | 0.9 (0.73-1.09) | 0.93 (0.34-2.25) | 0.05 (0.02-0.16) | 0 (0-0.01) |
| Poland | 2.18 (2.02-2.36) | 1.14 (1.07-1.22) | 0.01 (0.01-0.02) | 0.03 (0.03-0.04) | 0.23 (0.19-0.29) |
| Portugal | 2.84 (2.59-3.11) | 1.03 (0.96-1.1) | 0.08 (0.07-0.09) | 0.1 (0.09-0.11) | 0.1 (0.08-0.11) |
| Paraguay | 1.12 (0.96-1.31) | 0.78 (0.68-0.88) | 0.41 (0.18-0.94) | 0.21 (0.1-0.49) | 0.2 (0.09-0.46) |
| Palestinian Territories | 1.24 (0.86-1.77) | 1.6 (1.17-2.15) | 0.34 (0.13-0.89) | 0.5 (0.2-1.35) | 0.7 (0.3-1.54) |
| Qatar | 1.32 (0.9-1.92) | 1.67 (1.22-2.27) | 0.28 (0.1-0.85) | 0.46 (0.17-1.39) | 0.61 (0.24-1.67) |
| Romania | 1.8 (1.54-2.11) | 2.28 (2.03-2.58) | 0.13 (0.1-0.18) | 0.39 (0.3-0.52) | 0.69 (0.47-1) |
| Russia | 0.85 (0.76-0.93) | 1.17 (1.08-1.27) | 0.25 (0.1-0.71) | 0.01 (0.01-0.02) | 1.04 (0.76-1.42) |
| Rwanda | 1.54 (1.35-1.75) | 3.5 (3.16-3.89) | 0.5 (0.43-0.59) | 1.13 (0.98-1.31) | 0.25 (0.1-0.7) |
| Saudi Arabia | 1.61 (1.18-2.18) | 1.53 (1.1-2.15) | 0.27 (0.11-0.76) | 0.73 (0.29-1.79) | 0.85 (0.36-1.83) |
| Sudan | 1.03 (0.71-1.5) | 1.63 (1.17-2.28) | 0.15 (0.07-0.38) | 0.3 (0.14-0.71) | 0.52 (0.23-1.23) |
| Senegal | 0.67 (0.6-0.74) | 0.93 (0.84-1.01) | 0.29 (0.25-0.33) | 0.26 (0.22-0.3) | 0.27 (0.11-0.77) |
| Singapore | 1.48 (1.23-1.78) | 1.51 (1.27-1.77) | 0.18 (0.06-0.57) | 0.17 (0.14-0.22) | 0.08 (0.06-0.1) |
| Solomon Islands | 0.69 (0.54-0.86) | 1.94 (1.49-2.48) | 0.21 (0.09-0.59) | 0.33 (0.15-0.81) | 0.04 (0.02-0.09) |
| Sierra Leone | 1.12 (0.99-1.28) | 1.49 (1.33-1.65) | 0.13 (0.11-0.16) | 0.31 (0.27-0.36) | 0.09 (0.04-0.24) |
| El Salvador | 1.46 (1.01-2.13) | 1.44 (1.03-2.03) | 0.59 (0.25-1.41) | 0.44 (0.2-1.11) | 0.1 (0.05-0.26) |
| Serbia | 1.38 (0.91-2.1) | 3.79 (2.54-5.19) | 0.67 (0.29-1.43) | 0.38 (0.2-0.75) | 0.25 (0.11-0.6) |
| South Sudan | 0.82 (0.61-1.13) | 1.8 (1.24-2.62) | 0.23 (0.1-0.64) | 0.38 (0.17-0.96) | 0.28 (0.12-0.73) |
| São Tomé and Príncipe | 1.63 (1.36-1.96) | 0.45 (0.38-0.54) | 0.48 (0.4-0.58) | 0.12 (0.09-0.16) | 0.31 (0.13-0.91) |
| Suriname | 1.23 (0.85-1.78) | 2.05 (1.5-2.82) | 0.41 (0.18-0.99) | 0.94 (0.45-1.96) | 0.51 (0.23-1.13) |
| Slovakia | 0.74 (0.66-0.84) | 0.65 (0.59-0.71) | 0.02 (0.01-0.05) | 0.06 (0.04-0.09) | 0.21 (0.17-0.28) |
| Slovenia | 0.71 (0.61-0.83) | 0.73 (0.64-0.82) | 0.89 (0.38-1.83) | 0.23 (0.1-0.51) | 0.59 (0.29-1.22) |
| Sweden | 0.77 (0.71-0.85) | 1 (0.93-1.08) | 0.09 (0.08-0.11) | 0.13 (0.11-0.15) | 0.17 (0.15-0.19) |
| Eswatini | 1.14 (1.02-1.28) | 2.04 (1.75-2.35) | 0.23 (0.19-0.27) | 0.15 (0.12-0.18) | 0.26 (0.18-0.38) |
| Seychelles | 1.31 (1.13-1.52) | 2.53 (2.24-2.87) | 0.07 (0.06-0.09) | 0.26 (0.22-0.32) | 0.33 (0.14-0.86) |
| Syria | 1.14 (0.86-1.51) | 1.4 (1.11-1.77) | 0.32 (0.13-0.87) | 0.49 (0.2-1.29) | 0.68 (0.29-1.52) |
| Chad | 0.55 (0.48-0.63) | 0.47 (0.42-0.53) | 0.24 (0.21-0.29) | 0.11 (0.08-0.14) | 0.34 (0.14-0.89) |
| Togo | 0.69 (0.55-0.87) | 1.86 (1.57-2.25) | 0.19 (0.15-0.24) | 0.19 (0.15-0.25) | 0.34 (0.15-0.82) |
| Thailand | 1.07 (0.8-1.42) | 1.76 (1.37-2.29) | 0.15 (0.06-0.4) | 0.47 (0.19-1.15) | 0.04 (0.02-0.09) |
| Tajikistan | 0.94 (0.74-1.2) | 0.44 (0.34-0.57) | 0.51 (0.4-0.65) | 0.76 (0.55-1.08) | 0.24 (0.1-0.63) |
| Turkmenistan | 0.68 (0.52-0.9) | 0.81 (0.59-1.1) | 0.25 (0.1-0.63) | 0.39 (0.18-1.04) | 0.04 (0.02-0.1) |
| Timor-Leste | 0.59 (0.5-0.69) | 1.55 (1.38-1.75) | 0.23 (0.2-0.27) | 0.1 (0.09-0.13) | 0.05 (0.02-0.13) |
| Tonga | 1.46 (1.17-1.84) | 1.51 (1.27-1.8) | 0.16 (0.06-0.48) | 0.42 (0.17-1.22) | 0.1 (0.04-0.29) |
| Trinidad & Tobago | 0.81 (0.7-0.93) | 0.96 (0.84-1.08) | 0.28 (0.21-0.37) | 0.52 (0.43-0.63) | 0.31 (0.14-0.68) |
| Tunisia | 0.69 (0.59-0.79) | 2.01 (1.78-2.26) | 0.37 (0.15-0.99) | 0.21 (0.13-0.35) | 0.23 (0.16-0.33) |
| Turkey | 0.85 (0.75-0.98) | 1.14 (1.02-1.27) | 0.34 (0.13-0.89) | 0.43 (0.28-0.64) | 0.4 (0.25-0.65) |
| Taiwan | 1.53 (1.24-1.88) | 2.79 (2.38-3.29) | 0.19 (0.07-0.52) | 0.07 (0.05-0.09) | 0.08 (0.07-0.1) |
| Tanzania | 0.84 (0.75-0.94) | 2.16 (1.97-2.37) | 0.21 (0.18-0.25) | 0.49 (0.42-0.56) | 0.6 (0.26-1.37) |
| Uganda | 0.71 (0.64-0.8) | 1.42 (1.29-1.56) | 0.22 (0.19-0.26) | 0.65 (0.57-0.75) | 0.28 (0.12-0.74) |
| Ukraine | 0.87 (0.75-1) | 1.16 (1.04-1.3) | 0.31 (0.13-0.86) | 0.1 (0.04-0.25) | 0.75 (0.33-1.66) |
| Uruguay | 1.13 (0.98-1.3) | 1.78 (1.59-1.98) | 0.4 (0.15-1.11) | 0.56 (0.23-1.53) | 0.84 (0.34-1.87) |
| United States | 0.6 (0.56-0.63) | 0.87 (0.83-0.91) | 0.06 (0.05-0.06) | 0.17 (0.15-0.18) | 0.38 (0.35-0.41) |
| Uzbekistan | 0.83 (0.65-1.05) | 1.61 (1.17-2.26) | 0.67 (0.52-0.85) | 0.4 (0.18-0.97) | 0.16 (0.07-0.39) |
| St. Vincent & Grenadines | 1.69 (1.13-2.5) | 1.16 (0.83-1.57) | 0.37 (0.16-0.87) | 0.6 (0.29-1.33) | 0.2 (0.09-0.47) |
| Venezuela | 1.28 (0.89-1.84) | 1.03 (0.74-1.41) | 0.51 (0.2-1.33) | 0.5 (0.21-1.35) | 0.03 (0.01-0.07) |
| Vietnam | 0.93 (0.81-1.06) | 1.83 (1.66-2.04) | 0.51 (0.33-0.78) | 1.15 (0.77-1.72) | 0.65 (0.28-1.45) |
| Vanuatu | 0.47 (0.37-0.59) | 0.79 (0.62-1.01) | 0.19 (0.08-0.52) | 0.76 (0.33-1.79) | 0.13 (0.06-0.32) |
| Samoa | 1.6 (1.25-2.05) | 3.72 (3.03-4.54) | 0.16 (0.06-0.44) | 2.66 (1.39-4.15) | 0.87 (0.38-1.82) |
| Yemen | 0.46 (0.38-0.55) | 0.68 (0.56-0.81) | 0.35 (0.28-0.42) | 0.41 (0.33-0.51) | 0.12 (0.04-0.36) |
| South Africa | 0.4 (0.36-0.44) | 1.56 (1.43-1.7) | 0.27 (0.12-0.67) | 0.44 (0.33-0.59) | 0.01 (0.01-0.01) |
| Zambia | 0.65 (0.59-0.72) | 2.49 (2.3-2.68) | 0.17 (0.15-0.2) | 0.3 (0.25-0.35) | 0.73 (0.34-1.51) |
| Zimbabwe | 1.05 (0.96-1.15) | 1.78 (1.63-1.93) | 0.26 (0.23-0.3) | 0.27 (0.23-0.31) | 0.12 (0.08-0.18) |

Table S22: Mean absolute difference (servings/day) by sex in youth 0-19 years in 2018.

| Region | Fruit | Vegetables (non-starchy) | Other non-potato starchy vegetables | Beans and legumes | Nuts and seeds | Total Plant-Based Food Consumption |
| --- | --- | --- | --- | --- | --- | --- |
| Globe | -0.07 (-0.1--0.05) | -0.08 (-0.13--0.03) | -0.02 (-0.04-0) | -0.01 (-0.02-0.01) | 0 (-0.03-0.02) | -0.18, (-0.32--0.05) |
| South Asia | 0.03 (0-0.06) | -0.06 (-0.13-0.01) | -0.02 (-0.04-0) | 0 (-0.04-0.04) | -0.01 (-0.04-0.02) | -0.06, (-0.25,0.13) |
| Sub-Saharan Africa | -0.01 (-0.03-0.02) | -0.04 (-0.09-0) | -0.02 (-0.03-0) | -0.01 (-0.03-0.01) | 0 (-0.08-0.08) | -0.08 (-0.26-0.09) |
| Former Soviet Union | -0.17 (-0.22--0.12) | -0.1 (-0.16--0.04) | -0.02 (-0.1-0.05) | 0.01 (-0.02-0.05) | -0.02 (-0.13-0.08) | -0.30 (-0.63-0.02) |
| Middle East and North Africa | -0.11 (-0.17--0.06) | -0.13 (-0.19--0.07) | -0.02 (-0.07-0.02) | 0 (-0.05-0.06) | 0.01 (-0.04-0.07) | -0.25 (-0.52-0.02) |
| Latin America and Caribbean | -0.1 (-0.16--0.04) | -0.07 (-0.12--0.01) | 0 (-0.05-0.04) | 0.03 (-0.02-0.08) | 0 (-0.05-0.05) | -0.14 (-0.40-0.12) |
| High Income Countries | -0.11 (-0.15--0.08) | -0.1 (-0.13--0.07) | -0.01 (-0.02-0.01) | 0.02 (0.01-0.02) | 0.01 (0-0.02) | -0.19 (-0.29--0.10) |
| East and Southeast Asia | -0.2 (-0.27--0.13) | -0.16 (-0.33-0.01) | -0.02 (-0.09-0.03) | -0.03 (-0.06--0.01) | 0 (-0.04-0.03) | -0.41 (-0.79--0.07) |

Table S23: Mean percent difference (servings/day) by sex in youth 0-19 years in 2018.

| Region | Fruit | Vegetables (non-starchy) | Other non-potato starchy vegetables | Beans and legumes | Nuts and Seeds |
| --- | --- | --- | --- | --- | --- |
| Globe | -10.26 (-12.96, -7.51) | -5.91 (-9.10, -2.54) | -8.71 (-16.71, -0.49) | -2.69 (-8.05, 2.88) | -1.01 (-10.30, 8.51) |
| South Asia | 7.51 (-0.29-16.27) | -4.64 (-10.26-1.07) | -11.22 (-23.8-3.12) | 0.27 (-14.31-16.83) | -3.56 (-24.38-20.44) |
| Sub-Saharan Africa | -1.2 (-5.62-3.44) | -3.62 (-7.51-0.29) | -7.35 (-13.96--0.54) | -3.86 (-10.59-3.2) | 1.05 (-17.84-23.27) |
| Former Soviet Union | -19.11 (-23.96--14.09) | -11.22 (-17.14--4.84) | -7.08 (-29.52-21.06) | 11.92 (-13.26-43.11) | -2.49 (-16.32-11.73) |
| Middle East and North Africa | -13.2 (-18.64--7.28) | -12.12 (-17.45--6.68) | -9.37 (-24.38-7.89) | 2.03 (-16.98-24.29) | 5.29 (-12.82-26.54) |
| Latin America and Caribbean | -8.78 (-14.03--3.43) | -6.27 (-11.12--1.08) | -0.41 (-8.66-8.66) | 5.96 (-5.38-18.92) | 2.48 (-27.26-38.67) |
| High Income Countries | -12.41 (-16.32--8.41) | -12.49 (-15.5--9.44) | -6.06 (-17.54-7.47) | 13.28 (6.03-20.71) | 4.64 (-0.9-10.62) |
| East and Southeast Asia | -20.28 (-26.1--14.04) | -7.46 (-15.18-0.31) | -12.13 (-38.67-21.99) | -13.98 (-23.74--3.83) | -0.8 (-15.41-16.17) |

Table S24: Mean absolute difference (servings/day) by urban/rural residence in youth 0-19 years in 2018.

| Region | Fruit | Vegetables (Non-starchy) | Other non-potato starchy vegetables | Beans and legumes | Nuts and seeds | Total Plant-Based Food Consumption |
| --- | --- | --- | --- | --- | --- | --- |
| Globe | 0.24 (0.22-0.26) | -0.01 (-0.04-0.02) | 0.03 (0.02-0.05) | 0.01 (0-0.03) | 0.1 (0.07-0.13) | 0.37 (0.27-0.49) |
| South Asia | 0.12 (0.09-0.15) | 0.04 (-0.03-0.11) | 0.02 (0-0.04) | -0.02 (-0.05-0.01) | 0.07 (0.02-0.12) | 0.23 (0.03-0.43) |
| Sub-Saharan Africa | 0.12 (0.09-0.15) | 0 (-0.05-0.04) | 0 (-0.01-0.02) | 0 (-0.02-0.02) | 0.25 (0.11-0.44) | 0.37 (0.12-0.67) |
| Former Soviet Union | -0.04 (-0.1-0.02) | -0.02 (-0.09-0.04) | -0.01 (-0.07-0.07) | -0.06 (-0.1--0.02) | 0.15 (0.03-0.28) | 0.02 (-0.33-0.39) |
| Middle East and North Africa | 0.26 (0.21-0.33) | 0.12 (0.04-0.19) | 0.01 (-0.03-0.07) | 0.07 (0.02-0.14) | 0.1 (0.05-0.17) | 0.56 (0.29-0.90) |
| Latin America and Caribbean | 0.12 (0.06-0.17) | 0.01 (-0.03-0.06) | -0.02 (-0.05-0.01) | 0.01 (-0.02-0.05) | 0.02 (-0.03-0.06) | 0.14 (-0.07-0.35) |
| High Income Countries | -0.21 (-0.27--0.16) | -0.01 (-0.04-0.02) | 0 (-0.01-0.01) | 0.03 (0.02-0.04) | -0.05 (-0.06--0.03) | -0.24 (-0.36--0.12) |
| East and Southeast Asia | 0.16 (0.13-0.2) | 0.09 (0.04-0.15) | -0.01 (-0.07-0.05) | 0.01 (-0.01-0.03) | 0.05 (0.02-0.07) | 0.30 (0.11-0.50) |

Table S25: Mean percent difference (servings/day) by urban/rural residence in youth 0-19 years in 2018.

| Region | | Fruit | Vegetables (non-starchy) | Other non-potato starchy vegetables | Beans and legumes | Nuts and seeds |
| --- | --- | --- | --- | --- | --- | --- |
| Globe | 42.03 (38.48, 45.57) | | -1.09 (-3.27, 1.07) | 18.46 (8.04, 30.24) | 4.54 (-2.01,11.24) | 49.97 (30.79, 69.56) |
| South Asia | 35.84 (26.39-45.46) | | 3.08 (-2.03-8.8) | 15.35 (-0.51-31.81) | -6.32 (-17.98-5.63) | 72.44 (19.5-140.04) |
| Sub-Saharan Africa | 21.89 (16.65-27.47) | | -0.15 (-4.21-3.85) | 0.39 (-7.15-10.48) | -1.77 (-8.67-6.41) | 88.94 (41.67-146.07) |
| Former Soviet Union | -4.73 (-11.62-2.42) | | -2.17 (-9.78-5.08) | -5.04 (-26.2-26.97) | -31.41 (-47.48--10.89) | 24.99 (4.73-48.4) |
| Middle East and North Africa | 41.89 (31.74-53.11) | | 13.25 (4.79-22) | 4.15 (-14.93-27.25) | 34.52 (10.58-62.89) | 49.84 (21.11-81.65) |
| Latin America and Caribbean | 11.93 (6.03-18.21) | | 1.31 (-3.13-5.96) | -3.35 (-9.17-2.62) | 2.67 (-5.08-10.68) | 13.25 (-17.49-50.97) |
| High Income Countries | -20.91 (-25.56--16.35) | | -1.65 (-5.86-2.43) | 4.66 (-8.13-19.39) | 23.8 (13.42-35.05) | -20.33 (-26.07--14.02) |
| East and Southeast Asia | 20.72 (16.29-25.21) | | 4.91 (2.14-7.86) | -7.15 (-32.92-29.66) | 6.01 (-3.82-15.98) | 23.68 (6.76-38.38) |

Table S26: Mean absolute difference (servings/day) by education in youth 0-19 years in 2018.

| Region | Fruit | Vegetables (Non-starchy) | Other non-potato starchy vegetables | | Beans/Legumes | Nuts and seeds | Total Plant-Based Food Consumption |
| --- | --- | --- | --- | --- | --- | --- | --- |
| Globe | 0.41 (0.38-0.44) | 0.13 (0.09-0.17) | | 0.04 (0.01-0.06) | -0.02 (-0.04--0.01) | 0.13 (0.08-0.18) | 0.69 (0.52-0.84) |
| South Asia | 0.3 (0.26-0.35) | 0.18 (0.1-0.27) | | 0.07 (0.04-0.12) | 0.1 (0.05-0.14) | 0.04 (-0.01-0.11) | 0.69 (0.44-0.99) |
| Sub-Saharan Africa | 0.39 (0.33-0.44) | 0.38 (0.31-0.46) | | 0.08 (0.05-0.12) | 0.1 (0.07-0.14) | 0.33 (0.13-0.59) | 1.28 (0.89-1.75) |
| Former Soviet Union | -0.01 (-0.08-0.06) | -0.09 (-0.17--0.02) | | 0.05 (-0.03-0.14) | 0 (-0.04-0.05) | 0.22 (0.05-0.4) | 0.17 (-0.27-0.63) |
| Middle East and North Africa | 0.31 (0.23-0.39) | 0.2 (0.12-0.28) | | 0.03 (-0.02-0.08) | 0 (-0.06-0.06) | 0.08 (0.01-0.15) | 0.62 (0.28-0.96) |
| Latin America and Caribbean | 0.44 (0.36-0.53) | 0.39 (0.32-0.47) | | 0.18 (0.13-0.24) | -0.09 (-0.14--0.04) | 0.15 (0.07-0.25) | 1.07 (0.74-1.45) |
| High Income Countries | -0.16 (-0.23--0.1) | 0.05 (0.01-0.09) | | 0 (-0.01-0.02) | -0.08 (-0.11--0.05) | 0.16 (0.14-0.17) | -0.03 (-0.20-0.13) |
| East and Southeast Asia | 0.43 (0.37-0.49) | 0.3 (0.22-0.38) | | -0.01 (-0.07-0.07) | -0.02 (-0.05-0) | 0.04 (0.01-0.07) | 0.74 (0.48-1.01) |

Table S27: Mean percent difference (servings/day) by education in youth 0-19 years in 2018.

| Region | Fruit | Vegetables (non-starchy) | Other non-potato starchy vegetables | Beans and legumes | Nuts and seeds |  |
| --- | --- | --- | --- | --- | --- | --- |
| Globe | 73.32 (67.74, 78.99) | 9.52 (6.12, 13.00) | 17.67 (5.61, 34.05) | -10.84 (-17.14, -4.32) | 63.45 (28.60, 99.75) |  |
| South Asia | 100.13 (83.06-119) | 15.59 (8.19-23.6) | 61.64 (34.42-95.72) | 46.37 (22.28-70.29) | 33.05 (-7.2-86.24) |  |
| Sub-Saharan Africa | 74.12 (63.9-85.12) | 35.04 (28.01-42.35) | 44.13 (28.83-63.31) | 42.59 (29.23-58.03) | 98.35 (37.5-179.23) |  |
| Former Soviet Union | -0.82 (-9.7-8.56) | -10.47 (-18.38--2.61) | 23.61 (-11.21-65.22) | 3.84 (-24.91-40.95) | 38.36 (7.24-79.7) |  |
| Middle East and North Africa | 44.59 (32.17-57.32) | 21.52 (12.52-31.04) | 14.65 (-6.99-39.78) | 2.97 (-18.91-27.27) | 34.4 (4.4-68.43) |  |
| Latin America and Caribbean | 48.55 (38.61-59.26) | 44.09 (34.97-54.15) | 40.92 (28.61-54.42) | -18.36 (-26.45--9.59) | 149.18 (56.16-272.87) |  |
| High Income Countries | -16.67 (-22.54--10.29) | 7.7 (1.77-13.85) | 7.36 (-15.38-27.35) | -37.9 (-47.43--29.17) | 162.07 (135.12-190.02) | |
| East and Southeast Asia | 61.99 (53.49-71.07) | 16.51 (11.82-21.07) | -3.48 (-34.63-44.85) | -9.57 (-20.69-1.64) | 21.47 (4.49-37.34) |  |

Table S28: Mean absolute difference (servings/day) by year comparing 1990 to 2018 in youth 0-19.

| Region | Fruit | Vegetables (Non-starchy) | Other non-potato starchy vegetables | Beans and legumes | Nuts and seeds | Total Plant-Based Food Consumption |
| --- | --- | --- | --- | --- | --- | --- |
| Globe | 0.02 (0-0.05) | 0.44 (0.41-0.47) | -0.04 (-0.08--0.02) | 0.01 (0-0.02) | 0.15 (0.12-0.19) | 0.58 (0.45-0.71) |
| South Asia | 0.03 (0.02-0.05) | 0.03 (0.02-0.05) | -0.04 (-0.05--0.02) | 0.01 (-0.01-0.03) | 0.04 (0.02-0.08) | 0.07 (0-0.19) |
| Sub-Saharan Africa | 0.01 (0-0.02) | 0.1 (0.09-0.12) | -0.08 (-0.1--0.06) | -0.01 (-0.02-0) | 0.18 (0.1-0.3) | 0.20 (0.07-0.38) |
| Former Soviet Union | 0.12 (0.09-0.16) | 0.17 (0.14-0.2) | -0.04 (-0.11-0.01) | 0.01 (-0.02-0.03) | 0.55 (0.41-0.7) | 0.81 (0.51-1.10) |
| Middle East and North Africa | 0.04 (0.02-0.06) | 0.05 (0.03-0.06) | 0.02 (-0.01-0.05) | -0.02 (-0.06-0.02) | 0.09 (0.05-0.16) | 0.18 (0.03-0.35) |
| Latin America and Caribbean | 0.23 (0.21-0.25) | 0.28 (0.26-0.3) | -0.01 (-0.03-0.02) | 0.01 (-0.01-0.03) | 0.13 (0.08-0.2) | 0.64 (0.51-0.80) |
| High Income Countries | 0.05 (0.04-0.06) | 0.06 (0.05-0.06) | -0.02 (-0.02--0.01) | 0 (0-0) | 0.12 (0.11-0.13) | 0.21 (0.18-0.24) |
| East and Southeast Asia | 0.05 (0-0.11) | 1.34 (1.23-1.45) | -0.06 (-0.15--0.01) | 0.01 (0-0.02) | 0.17 (0.14-0.21) | 2.52 (1.22-1.78) |

Table S29: Mean percent differences (servings/day) by year comparing 1990 to 2018 in youth 0-19.

| Region | Fruit | Vegetables (non-starchy) | Other non-potato starchy vegetables | Beans and legumes | Nuts and seeds |
| --- | --- | --- | --- | --- | --- |
| Globe | 3.61 (0.02-7.2) | 50.25 (47.27-53.44) | -18.08 (-27.56--10.76) | 2.83 (-0.56-6.37) | 166.8 (141.36-197.32) |
| South Asia | 10.37 (6.78-14.02) | 2.83 (1.43-4.26) | -23.48 (-30.02--15.37) | 3.18 (-3.23-12.61) | 51.62 (23.79-91.15) |
| Sub-Saharan Africa | 1.78 (0.3-3.28) | 9.77 (8.22-11.29) | -28.02 (-33.41--22.24) | -3.86 (-8.29-0.93) | 90.98 (55.39-134.81) |
| Former Soviet Union | 18.25 (13.73-23.15) | 25.32 (21.13-29.8) | -13.38 (-29.55-4.86) | 5.37 (-11.32-24.28) | 353.33 (289.09-421.76) |
| Middle East and North Africa | 5.16 (2.53-7.95) | 5.19 (3.39-6.88) | 9.42 (-3.97-23.94) | -6.69 (-18.45-6.41) | 49.18 (28-73.93) |
| Latin America and Caribbean | 27.95 (25.41-30.42) | 38.33 (36.09-40.62) | -1.04 (-5.36-3.48) | 2.43 (-1.25-6.11) | 421.58 (306.34-539.19) |
| High Income Countries | 6.56 (5.56-7.57) | 8.4 (7.89-8.9) | -17 (-23.35--9.44) | -1.34 (-3.09-0.12) | 137.76 (124.78-151.46) |
| East and Southeast Asia | 6.75 (-0.16-13.86) | 204.56 (191.27-219.11) | -25.25 (-44.68--3.26) | 3.74 (-0.03-8.02) | 350.92 (290.2-417.69) |
